# Antigen–Antibody Complex-Guided Exploration of the Hotspots Conferring the Immune-Escaping Ability of the SARS-CoV-2 RBD

**DOI:** 10.3389/fmolb.2022.797132

**Published:** 2022-03-22

**Authors:** Kit-Man Fung, Shu-Jung Lai, Tzu-Lu Lin, Tien-Sheng Tseng

**Affiliations:** ^1^ Academia Sinica, Institute of Biological Chemistry, Taipei, Taiwan; ^2^ Graduate Institute of Biomedical Sciences, China Medical University, Taichung, Taiwan; ^3^ Institute of Molecular Biology, National Chung Hsing University, Taichung, Taiwan

**Keywords:** SARS-CoV-2, COVID-19, binding stability, hotspots, neutralization, convalescent antibody, immunity

## Abstract

The COVID-19 pandemic resulting from the spread of SARS-CoV-2 spurred devastating health and economic crises around the world. Neutralizing antibodies and licensed vaccines were developed to combat COVID-19, but progress was slow. In addition, variants of the receptor-binding domain (RBD) of the spike protein confer resistance of SARS-CoV-2 to neutralizing antibodies, nullifying the possibility of human immunity. Therefore, investigations into the RBD mutations that disrupt neutralization through convalescent antibodies are urgently required. In this study, we comprehensively and systematically investigated the binding stability of RBD variants targeting convalescent antibodies and revealed that the RBD residues F456, F490, L452, L455, and K417 are immune-escaping hotspots, and E484, F486, and N501 are destabilizing residues. Our study also explored the possible modes of actions of emerging SARS-CoV-2 variants. All results are consistent with experimental observations of attenuated antibody neutralization and clinically emerging SARS-CoV-2 variants. We identified possible immune-escaping hotspots that could further promote resistance to convalescent antibodies. The results provide valuable information for developing and designing novel monoclonal antibody drugs to combat emerging SARS-CoV-2 variants.

## Introduction

The coronavirus disease 2019 (COVID-19) pandemic resulting from the spread of severe acute respiratory syndrome-coronavirus 2 (SARS-CoV-2) led to social, health, and economic crises worldwide (Lai et al., 2020). By early June 2021, SARS-CoV-2 had already infected 176.4 million people and caused 3.8 million deaths. SARS-CoV-2 infections presents through common symptoms such as a hacking cough, sore throat, nasal stuffiness, diarrhea, respiratory illness, and fever (Baj et al., 2020; Pal et al., 2020). Additionally, severe acute respiratory syndrome, pneumonia, and kidney failure were observed in severe cases and were fatal (Lai et al., 2020). Coronaviruses are a family of RNA virus with a genome comprising approximately 30 kilobases and can be divided into four genera (Fehr and Perlman, 2015). SARS-CoV-1 and SARS-CoV-2 belong to the *β*-genus. In SARS-CoV-2, the structural proteins, spike (S), nucleocapsid (N), envelope (E), and membrane (M), are encoded by its RNA genome (Malik, 2020; Mousavizadeh and Ghasemi, 2021; V'Kovski et al., 2021). The S protein consists of N-terminal signal peptide S1 and S2 subunits (Lan et al., 2020; Tai et al., 2020); the S1 subunit contains the receptor-binding domain (RBD) and N-terminal domain, and the S2 subunit is composed of a cytoplasm domain, TM domain, heptapeptide repeat sequence 1 (HR1), heptapeptide repeat sequence 2 (HR2), and fusion peptide (FP; (Xia et al., 2020). Notably, the FP of the S2 subunit is essential for facilitating viral membrane fusion with the host cell membrane (Millet and Whittaker, 2018). In addition, the HR1 and HR2 of the S2 subunit form the six-helix bundle (6-HB), which is critical for membrane fusion and viral entry (Chambers et al., 1990; Xia et al., 2020). In the native state, the S protein exists as an inactive precursor. During viral infection, a host cell protease (TMPRSS2) cleaves the S protein into the S1 and S2 subunits (Bertram et al., 2013; Hoffmann et al., 2020), after which the angiotensin-converting enzyme 2 (ACE2) receptor of the host cell is recognized and bound by the RBD of the S1 subunit (Wrapp et al., 2020). Following binding of the RBD to ACE2, the S2 subunit undergoes conformational changes. The FP enters the host cell membrane, and the prehairpin coiled-coil of the HR1 domain is exposed (Huang et al., 2020). Consequently, the 6-HB triggers HR1 and HR2, drawing the viral and host cellular membrane close enough for fusion (Xia et al., 2018). Remarkably, as a surface-exposed protein essential for entry into the host cell, the RBD is regarded as a first-line therapeutic target for developing vaccines and antiviral agents.

Rapid action and the development of vaccines protecting against COVID-19 are urgently required. Some vaccine candidates are being evaluated in preclinical models, and others are being investigated through human clinical trials (World Health (Organization, 2021). Currently, only four vaccines are licensed (Gomez et al., 2021). The pharmaceutical companies Janssen and Oxford University/AstraZeneca developed the Ad26.COV2.S (Sadoff et al., 2021) and AZD1222 (Voysey et al., 2021) vaccines, respectively, based on nonreplicating adenoviruses. The Janssen vaccine was developed from modified adenovirus serotype 26, which generates prefusion S protein to elicit immunity and has an efficacy of 66.9%. The monovalent AstraZeneca vaccine contains a replication-deficient chimpanzee adenovirus (ChAdOx1) that encodes the S protein. When administrated, the expressed S protein stimulates cellular immune response with an efficacy of 63.09%. Moderna (mRNA-1273; (Baden et al., 2021; Oronsky et al., 2021); and Pfizer/BioNTech (BNT162b2; (Polack et al., 2020); are two other vaccines that were developed based on mRNA. The genetic fragment encoding the prefusion form of the S protein enhancing uptake in the immune cells of the host are packed in these vaccines. Thus, the S protein can be produced through the transcription and translation machinery of the host cell, further eliciting adaptive immunity against COVID-19. Two regimens of BNT162b2 and mRNA-1273 exerted 95% (Polack et al., 2020) and 94.1% (Baden et al., 2021; Oronsky et al., 2021) protective efficacy, respectively. Although all of these vaccines can provide a level of protection against SARS-CoV-2, the rates of mutations evident in emerging variants that confer resistance to and immune-escaping ability toward neutralizing antibodies continue to grow (Geers et al., 2021; Gomez et al., 2021).

The devastating effects of the COVID-19 pandemic have increased the urgency to disrupt the spread and replication of SARS-CoV-2. Natural infection or vaccination can elicit neutralizing antibodies of adaptive immunity against viruses (Rydyznski Moderbacher et al., 2020; Bettini and Locci, 2021; Sette and Crotty, 2021). Alternatively, passive immunity can be conferred when antibodies are administrated in the form of recombinant proteins or convalescent plasma (Casadevall, 2002). SARS-CoV-2 infection can reportedly elicit neutralizing antibodies that potently recognize the RBD (Barnes et al., 2020; Gavor et al., 2020; Liu et al., 2020). Nevertheless, COVID-19 convalescents exhibit low levels of plasma neutralizing activity, indicating that B-cells’ generation of high-titer neutralizing antibodies through natural infection is insufficient (Weisblum et al., 2020b). The RBD of the SARS-CoV-2 S protein binds to ACE2 with high affinity, with neutralizing antibodies targeting the RBD exerting protective effects against infection in both animal models and humans (Dong et al., 2020). However, antigenic evolution has been observed eroding the immunity of neutralizing antibodies (Greaney A. J. et al., 2021; Eguia et al., 2021). The positive selection for mutations of RBD drives the antigenic evolution (Jaroszewski et al., 2020; Velazquez-Salinas et al., 2020). Furthermore, several researchers have demonstrated that mutations of the SARS-CoV-2 S protein contribute to its immune-escaping ability from monoclonal antibodies as well as polyclonal human sera (Weisblum et al., 2020a; Greaney A. J. et al., 2021; Chen et al., 2021; Harvey et al., 2021). Moreover, RBD mutants are emerging and were observed in SARS-CoV-2 pandemic isolates, presenting a considerable challenge for the combat against COVID-19. Thus, to understand the viral evolution, a comprehensive examination of the hotspots of the SARS-CoV-2 RBD that confers the ability to escape convalescent antibodies’ recognition is timely and necessary.

In this study, we comprehensively and systematically investigated the RBD variants that markedly destabilize binding to six convalescent neutralizing antibodies, namely CV07-270, B38, CT-P59, CA1-B12, CA1-B3, and 47D1. We utilized the complex structures of the RBD antibody in convalescents to conduct in-depth mutational scanning to estimate their binding stability. During calculation, each residue of the RBD contributing to antibody interaction was replaced with distinct amino acids to examine the hotspots that impair binding. Our results demonstrated that mutations at the residues of F456, F490, G416, G502, K417, L452, L455, N487, R403, Y449, and Y489 were unfavorable for RBD binding to most of the convalescent antibodies. Consistently, the K417 T/N, L452R, and E484K/Q mutants were identified in emerging SARS-CoV-2 variants. In addition, the emerging N501Y variant was identified in several viral lineages (P.1, C.37, B1.1.7, and B.1.351). Moreover, mutations at residues F456, F486, F490, G485, and L455 located near the RBD’s receptor-binding ridge reportedly have considerable antigenic effects. All of the aforementioned experimental and clinical evidence corroborates our results. Evidently, the RBD hotspots (F456, F490, G416, G502, K417, L452, L455, N487, R403, Y449, and Y489) explored in this study would benefit from the development of potent therapeutics to combat against emerging SARS-CoV-2 variants.

## Materials and Methods

### Preparation of Protein Structures

The complex structure of the RBD antibody was obtained using Protein Data Bank (PDB IDs: 6XKP, 7BZ5, 7CM4, 7KFV, 7KFW, and 7MF1). In addition, the CHARMm polar H forcefield was applied to all structures in advance for computation.

### Calculation of Mutational Binding Stability

The binding stability of the RBD variants in complex with convalescent antibodies was determined using MutaBind2 (Weisblum et al., 2020b), mCSM-PPI2 (Rodrigues et al., 2019), FoldX (Schymkowitz et al., 2005), and Discovery Studio (DS) 3.5 (Accelrys, San Diego, CA, United States). The calculations in mCSM-PPI2 and MutaBind2 were performed according to the online instructions. In the FoldX prediction, the binding stability was estimated using the method previously reported (Teng et al., 2021). The Calculate Mutation Energy (Binding) protocol of DS 3.5 was employed to evaluate the changes in binding stability upon mutation. The complex structure of the RBD antibody and RBD moiety were selected as the “input typed molecule” and “ligand chain” parameters, respectively. Additionally, the “mutation sites” parameter was set to a single mutation with all other 20 amino acids. Moreover, the solvent dielectric constant, maximum number of mutants, dielectric constant, and maximum structures to save were set to 80, 25, 10, and 10, respectively; the remaining parameters were default settings. The values of mutational energies denoted destabilized (positive) and stabilized (negative) binding in the predictions of DS, FoldX, and MutaBind2 but were reversed in mCSM-PPI2.

### Generation of the Heatmap of Mutational Binding Energy

Excel (Microsoft Office 2013; Microsoft, Redmond, WA, United States) was used to create the heatmap based on the output binding energy values from single mutations. In the heatmap, the *x*-axis represented the types of amino acids for single mutations, and the *y*-axis represented the mutated residues of the RBD. Conditional formatting was applied on the obtained post mutation binding energy values**.** Green and purple were employed to create the two-color gradient scales that designate the values of the binding energies.

### LIGPLOT Analyses

The LIGPLOT program (Wallace et al., 1995; Laskowski and Swindells, 2011) was used to analyze the molecular interactions within the complex structure of the RBD antibody. During the analysis, the “antibody” LIGPLOT module was employed to explore domain–domain and protein–protein interactions.

### Molecular Dynamic Simulation and Binding Affinity Prediction

The complex structures of RBD-CA1-B3 (PDB ID: 7KFW), RBD-CA1-B12 (PDB ID: 7KFV), RBD-CT-P59 (PDB ID:7CM4) and RBD-B38 (PDB ID: 7BZ5) were employed to perform molecular dynamics (MD) simulations. The L455 and F456 variants of RBD complexed with convalescent antibodies were generated by Build and Edit Protein module of Discovery Studio (DS) 3.5 (Accelrys, San Diego, CA, United States). Additionally, the covalently attached carbohydrates of glycosylated residues of RBD were removed before solvation step. The generated structures were firstly subjected to Solvation (Discovery Studio 3.5) with orthorhombic cell shape under CHARMm forcefield (Supplementary Table S1). Subsequently, the complex structures of RBD-convalescent antibodies were solvated with waters molecules (14,170–15,280), sodium atoms (40–41), and chloride atoms (49–52). Consequently, the Standard Dynamics Cascade (Discovery Studio 3.5) were conducted for each solvated complex structure for 5 ns simulation times with 2 ps as save results interval (detailed parameter setting was shown in Supplementary Table S2). Furthermore, the Analyze Trajectory tool (Discovery Studio 3.5) was employed to plot the total energy changes as functions of simulation time. After MD simulations, the binding affinities of RBD variants to convalescent antibodies were determined by CSM-AB, a machine learning method capable of predicting antibody–antigen binding affinity by modelling interaction interfaces as graph-based signatures (Myung et al., 2021).

## Results

### Deviations in the Binding Stability of RBD Variants Targeting Convalescent Antibody CV07-270

**FIGURE 1:**
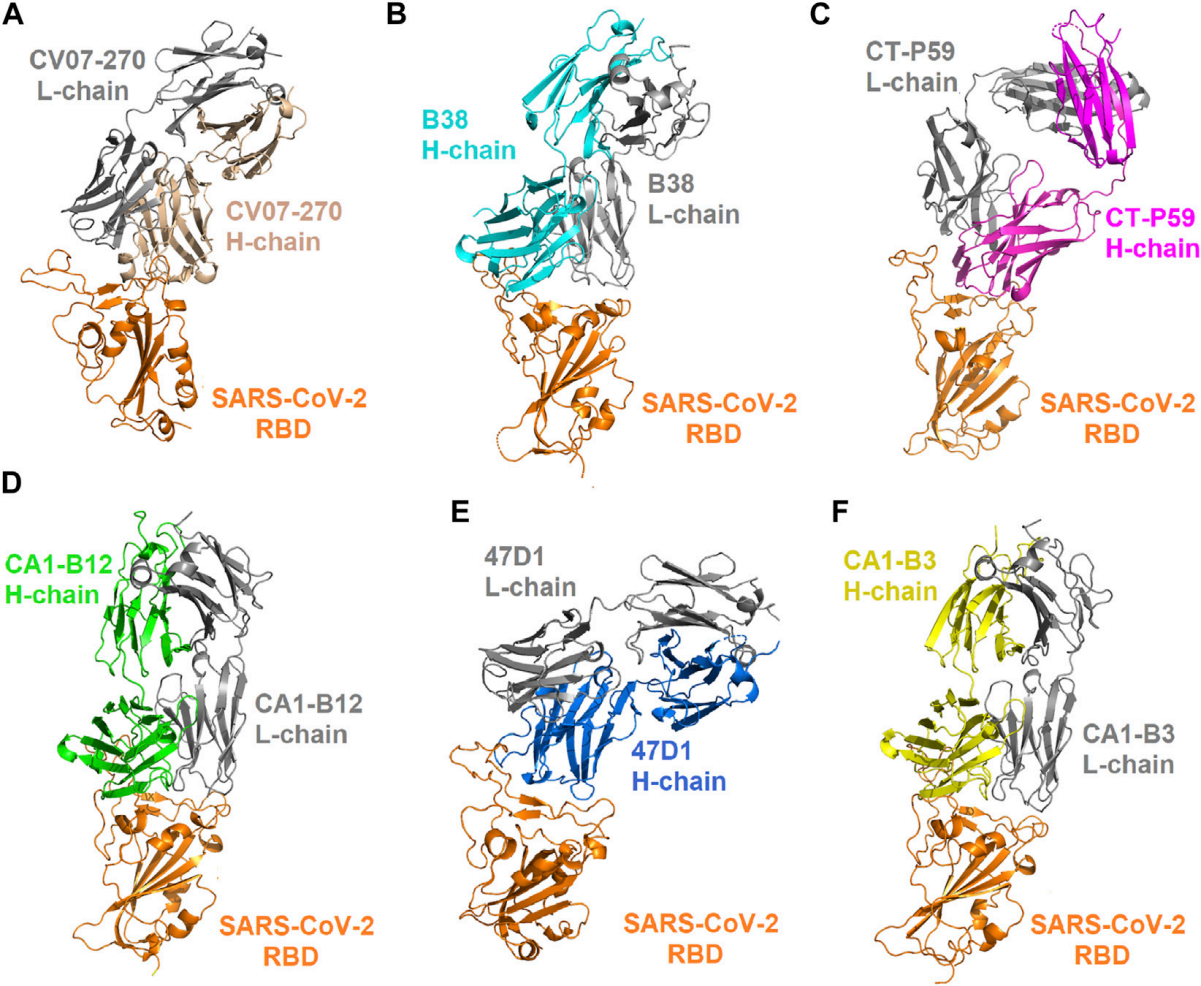
Complex structures of the SARS-CoV-2 RBD bound with convalescent antibodies. **(A)** The complex structure of the RBD–CV07-270 antibody (PDB ID: 6XKP). **(B)** The structure of the RBD in complex with the B38 antibody (PDB ID: 7BZ5). **(C)** The structure of the RBD bound with the CT-P59 antibody (PDB ID: 7CM4). **(D)** The structure of the CA1-B12 antibody in complex with the RBD (PDB ID: 7KFV). **(E)** The structure of the 47D1 antibody bound with the RBD (PDB ID: 7MF1). **(F)** The structure of the CA1-B3 antibody in complex with the RBD (PDB ID: 7KFW).

**FIGURE 2:**
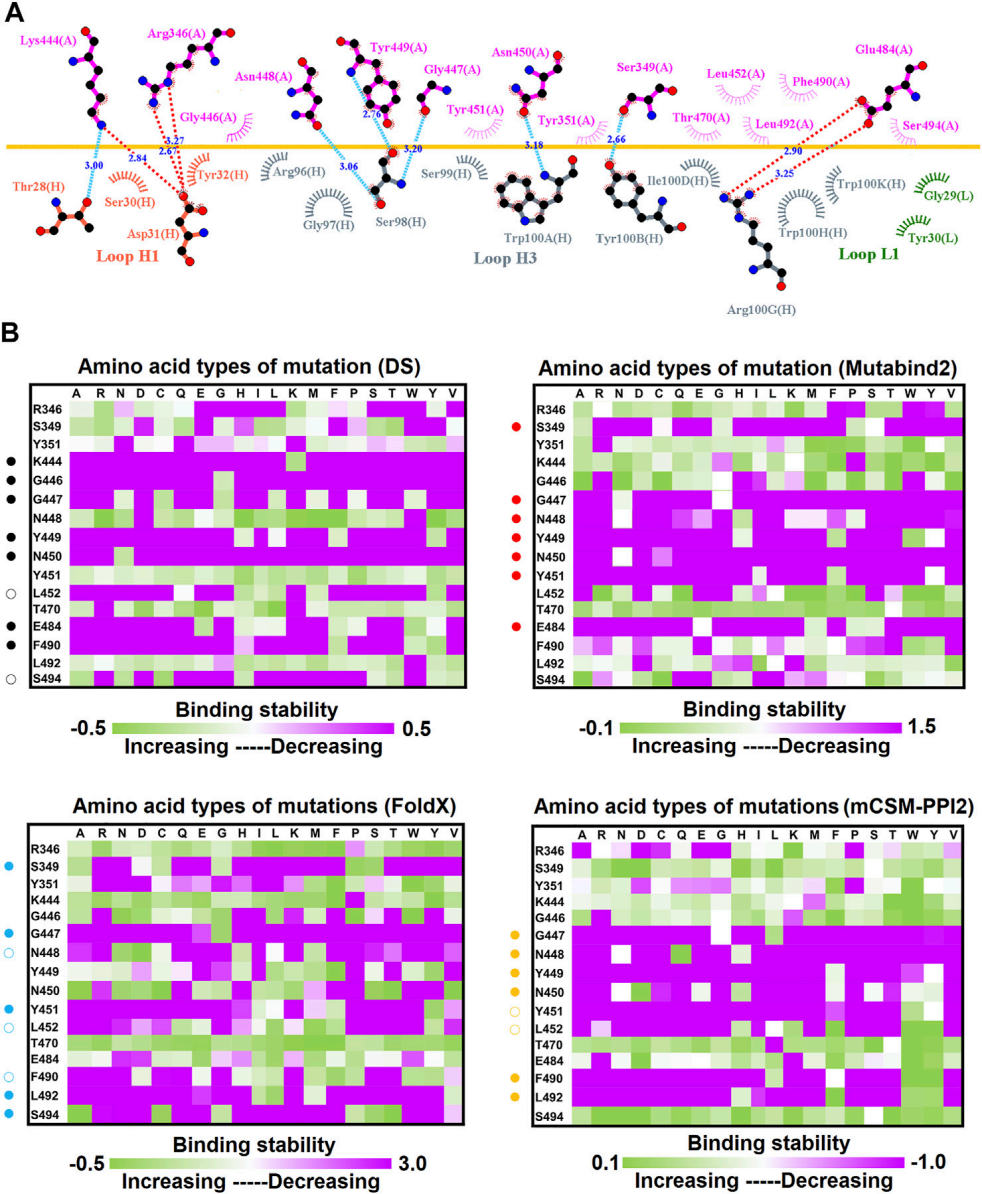
Mutational heatmaps of the RBD targeting antibody CV07-270. **(A)** The LIGPLOT presents the interaction network between CV07-270 and the SARS-CoV-2 RBD. The RBD and light and heavy chains of CV07-270 are labeled A, H, and L, respectively. The golden yellow line denotes the interface of the RBD–CV07-270 complex. The interactive residues of the RBD are labeled and colored magenta. The salt-bridges and hydrogen bonds are indicated by red and cyan dashes, and the hydrophobic interactions are represented by arcs with spokes radiating toward the ligand atoms with which they are in contact. **(B)** The mutational binding stabilities of RBD variants to CV07-270 were estimated using FoldX, MutaBind2, DS 3.5, and mCSM-PPI2. The unit of binding stability was the kcal/mole, with the obtained values employed to create the heatmaps. Green (increased stability) and purple (reduced stability) were used to generate a color gradient and were applied in each box. In each heatmap, the moderately and significantly reduced stabilities were labeled with hollow and solid circles, respectively.

To investigate the mutational effects of the RBD on its interactions with convalescent antibodies, we obtained the three-dimensional structures of the RBD antibody complex from Protein Data Bank. The convalescent antibody CV07-270 isolated from patients with COVID-19 has high affinity to the SARS-CoV-2 S protein (IC_50_ = 82.3 ng/ml; K_D_ is not applicable) (Kreye et al., 2020). The complex structure of RBD–CV07-270 is depicted in Figure 1A, with the LIGPLOT of its molecular interactions presented in Figure 2A. The RBD residues R346, S349, K444, G447, N448, Y449, N450, and E484 primarily form hydrogen bonds, and residues Y351, G446, Y451, L452, T470, F490, L492, and S494 achieve hydrophobic contact with CV07-270. Next, these residues were replaced with distinct amino acids to evaluate their mutational effects on targeting the CV07-270 antibody. The output binding energies upon mutation were used to generate the heatmap, on which the *x*-axis and *y*-axis indicate the amino acid types for various mutations and mutated residues, respectively. The values of the binding energies are colored purple (destabilized) and green (stabilized) within a gradient range. The results demonstrated that single-point mutation at residues E484, F490, G447, L452, L492, N448, N450, S439, S494, Y449, and Y451 were largely unfavorable for binding to antibodies (Figure 2B and Table 1).

**TABLE 1:** Functionally essential residues of the RBD mutations that confer the immune-escaping ability of SARS-CoV-2 in relation to neutralizing convalescent antibodies.

Destabilizing mutations on SARS-CoV-2 RBD																					
	Predictions validated with experiments						Predictions can be tested by future experiments														
Antibodies	K417^∗^	L452^∗^	E484^C^	F490^C∗^	S494	N501	R403^∗^	G416^∗^	Y421^∗^	Y449^∗^	Y453∗	L455^C∗^	F456^C∗^	F486^C^	N487^∗^	Y489^∗^	L492	Y495	G496	G502^∗^	Y505
CV07-270		O	O	O	O					O							O				
B38	O					O	O	O	O			O	O	O	O	O		O	O	O	O
CT-P59		O		O	O					O	O	O	O	O			O				
CA1-B12	O					O	O	O	O		O	O	O		O	O		O	O	O	
CA1-B3	O						O	O	O		O	O	O		O	O				O	O
47D1		O	O	O						O											
	**Emerging SARS-CoV-2 Variants**																				
^b^United Kingdom (Nigeria); B.1.525			E484K																		
^b^United Kingdom																					
B.1.1.7			E484K		S494P	N501Y															
^b^United States (New York); B.1.526			E484K																		
^b^United States (California)																					
B.1.427, B.1.429		L452R																			
^b^United States (New York); B.1.526.1		L452R																			
^b^India																					
B.1.617, B.1.617.1																					
B.1.617.2, B.1.617.3		L452R	E484Q																		
^b^Brazil, P.1	K417N		E484K			N501Y															
^b^Brazil, P.2			E484K		‘																
^b^South Africa, B.1.351	K417N		E484K			N501Y															
^b^Japan, P.1	K417T		E484K			N501Y															
^c^Peru, C.37		L452R		F490S		N501Y															

a Hotspots.

b [U.S. Department of Health and Human Services (https://www.cdc.gov/coronavirus/2019-ncov/cases-updates/variant-surveillance/variant-info.html)].

c [European Centre for disease Prevention and Control (https://www.ecdc.europa.eu/en/covid-19/variants-concern)].

d Mutations at other structurally adjacent sites in the RBD’s receptor-binding ridge can also have substantial antigenic effects.

### Mutational Scanning of the RBD Targeting Convalescent Antibody B38

**FIGURE 3:**
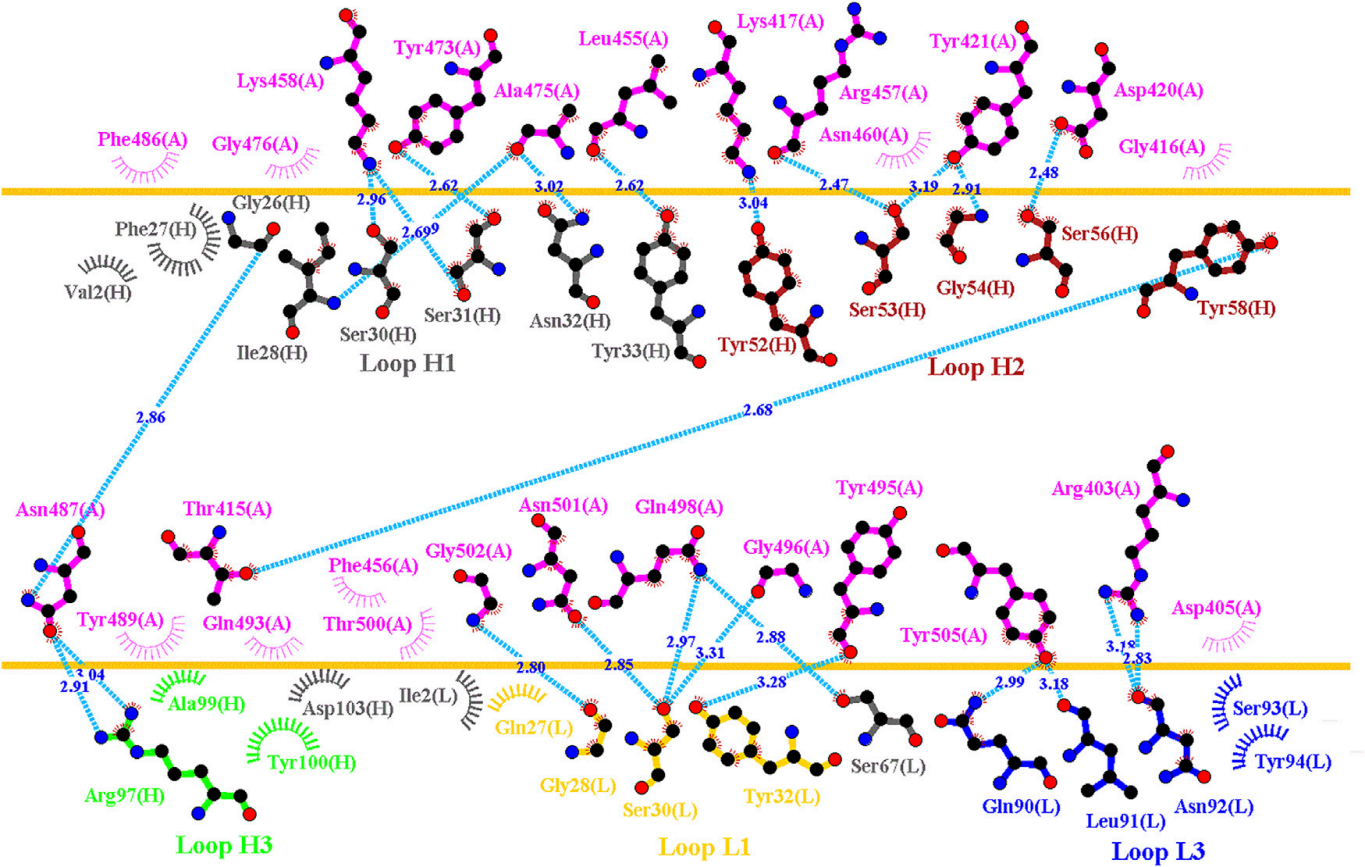
Interactional network of the RBD and B38 antibody analyzed using LIGPLOT. LIGPLOT was employed to examine the interaction network of the SARS-CoV-2 RBD and B38. The RBD and light and heavy chains of B38 are labeled A, H, and L, respectively. The golden yellow line denotes the interface of the RBD–B38 complex. The interactive residues of the RBD are labeled and colored magenta. The hydrogen bonds and hydrophobic interactions are represented by cyan dashes and arcs with spokes radiating toward the ligand atoms with which they are in contact, respectively.

**FIGURE 4:**
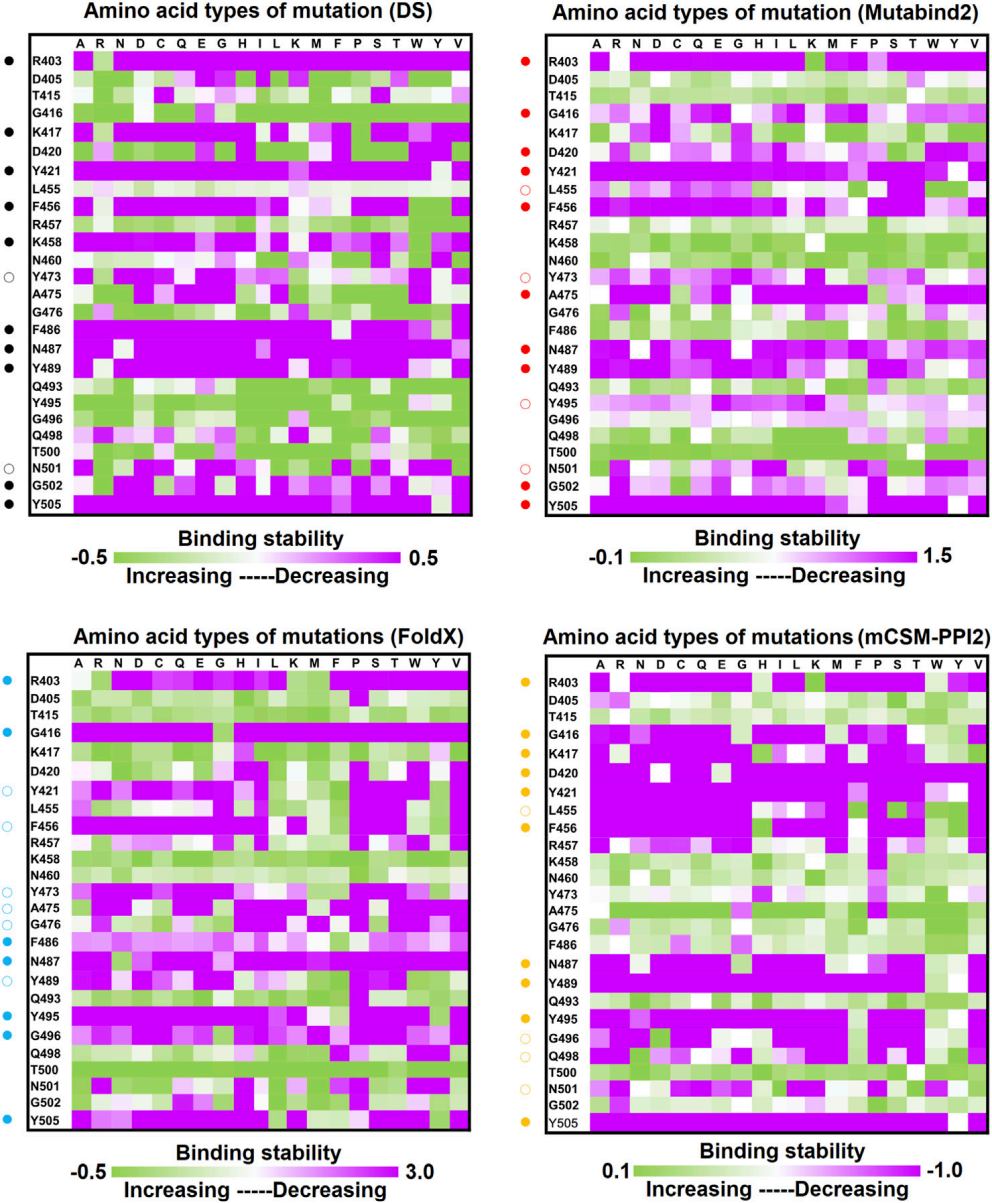
Mutational heatmaps of the RBD targeting antibody B38. The mutational binding stabilities of RBD variants to B38 were estimated using FoldX, MutaBind2, DS 3.5, and mCSM-PPI2. The unit of binding stability was kcal/mole, and the obtained values were employed to create the heatmaps. Green (increased stability) and purple (reduced stability) were used to generate a color gradient and were applied in each box. In each heatmap, the moderately and significantly reduced stabilities are labeled with hollow and solid circles, respectively.

Distinct complex structures were employed to analyze the effects of mutations on RBD binding to convalescent antibodies. The structure of the RBD in complex with a human-origin monoclonal antibody B38 (K_D_ = 14.3 nM) (Wu et al., 2020) from a convalescent patient is illustrated in Figure 1B (Wu et al., 2020). Figure 3 depicts the LIGPLOT of the RBD residues T415, R403, K417, D420, Y421, L455, R457, K458, Y473, A475, N487, Y495, G496, Q498, N501, G502, and Y505 that form hydrogen bonds and that of D405, G416, F456, N460, G476, F486, Y489, Q493, and T500 that made hydrophobic contact with B38 (Figure 3). These functionally essential residues were mutated to evaluate the effects on RBD binding to B38, with the results demonstrating that mutations at the residues R403, G416, K417, D420, Y421, L455, F456, Y473, F486, N487, Y489, Y495, G496, N501, G502, and Y505 were unfavorable for binding to the B38 convalescent antibody (Figure 4 and Table 1).

### Mutational Binding Stability of RBD Variants Targeting Convalescent Antibody CT-P59

**FIGURE 5:**
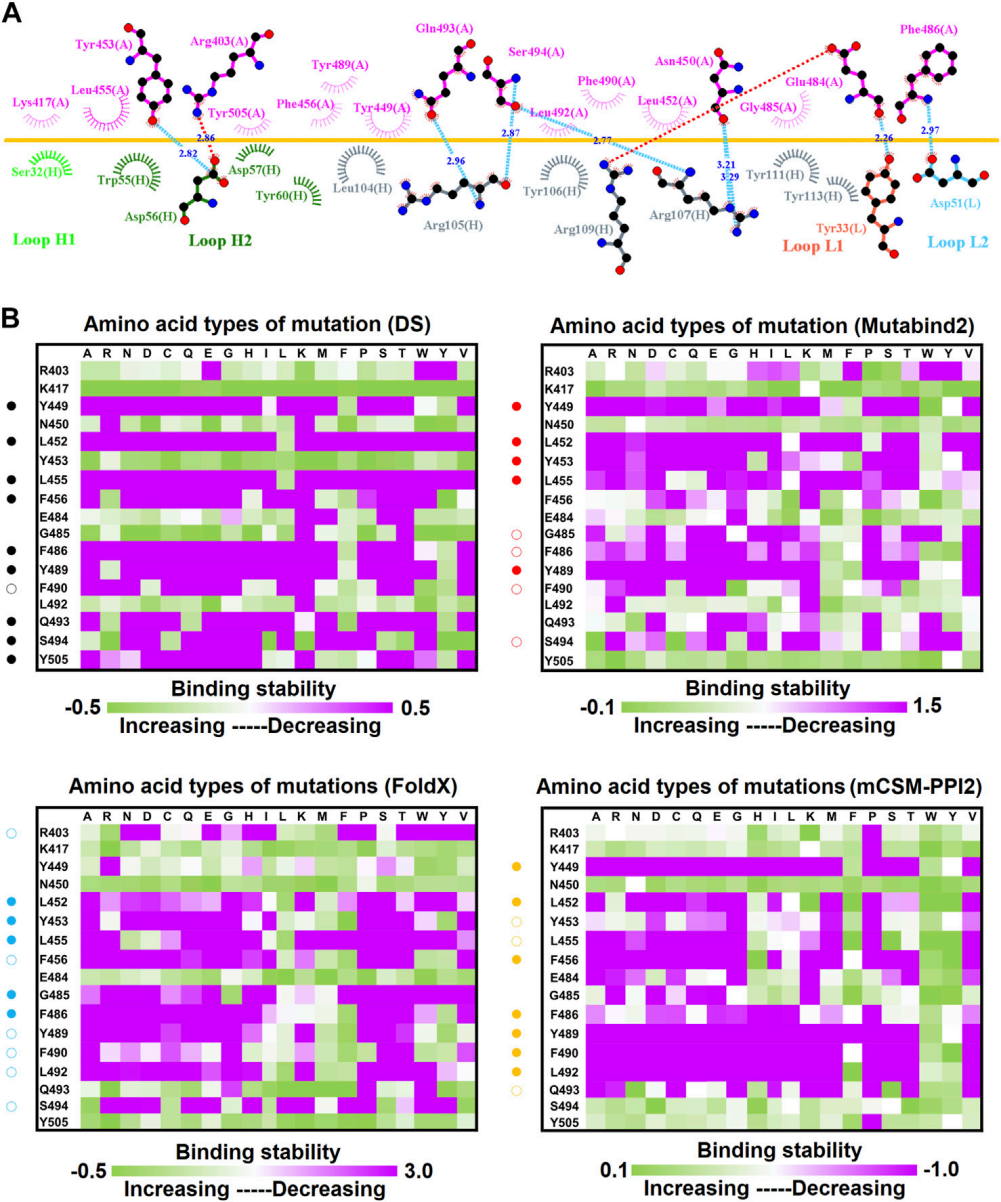
Mutational heatmaps of the RBD targeting antibody CT-P59. **(A)** LIGPLOT was employed to examine the interaction network of CT-P59 and SARS-CoV-2 RBD. The RBD and light and heavy chains of CT-P59 were labeled A, H, and L, respectively. The golden yellow line denotes the interface of the RBD–CT-P59 complex. The interactive residues of the RBD are labeled and colored magenta. The salt-bridges, hydrogen bonds and hydrophobic interactions are represented by red, cyan dashes and arcs with spokes radiating toward the ligand atoms with which they contact. **(B)** The mutational binding stabilities of RBD variants to CT-P59 were estimated using FoldX, MutaBind2, DS 3.5, and mCSM-PPI2. The unit of binding stability was kcal/mole, and the obtained values were employed to create the heatmaps. Green (increased stability) and purple (reduced stability) were used to generate a color gradient and were applied in each box. In each heatmap, the moderately and significantly reduced stabilities were labeled with hollow and solid circles, respectively.

The CT-P59 antibody neutralizes SARS-CoV-2 isolates through blocking of the interaction regions of the RBD (K_D_ = 0.05 nM) for ACE2 (Kim et al., 2021; Ryu et al., 2021). We assessed the destabilizing ability of RBD variants in binding to convalescent antibody CT-P59. The structure of the CT-P59 antibody in complex with the RBD is illustrated in Figure 1C. The molecular interactions analyzed using LIGPLOT demonstrated that hydrogen bond interactions were formed between the RBD residues E484, F486, N450, Q493, R403, S494, and Y453 and CT-P59 (Figure 5A). Additionally, the residues F456, F490, G485, L452, L455, L492, K417, Y449, Y489, and Y505 exhibited hydrophobic interaction with CT-P59. Therefore, RBD variants from the single-point mutations of the aforementioned residues were assessed in terms of binding stability. The results indicated that the residues F456, F486, F490, G485, L452, L455, L492, Q493, S494, Y449, and Y453 were unfavorable for binding to CT-P59 postmutation (Figure 5B and Table 1).

### Single-Point Mutations of the RBD Interfered With its Binding to Convalescent Antibodies C1A-B12 and C1A-B3

**FIGURE 6:**
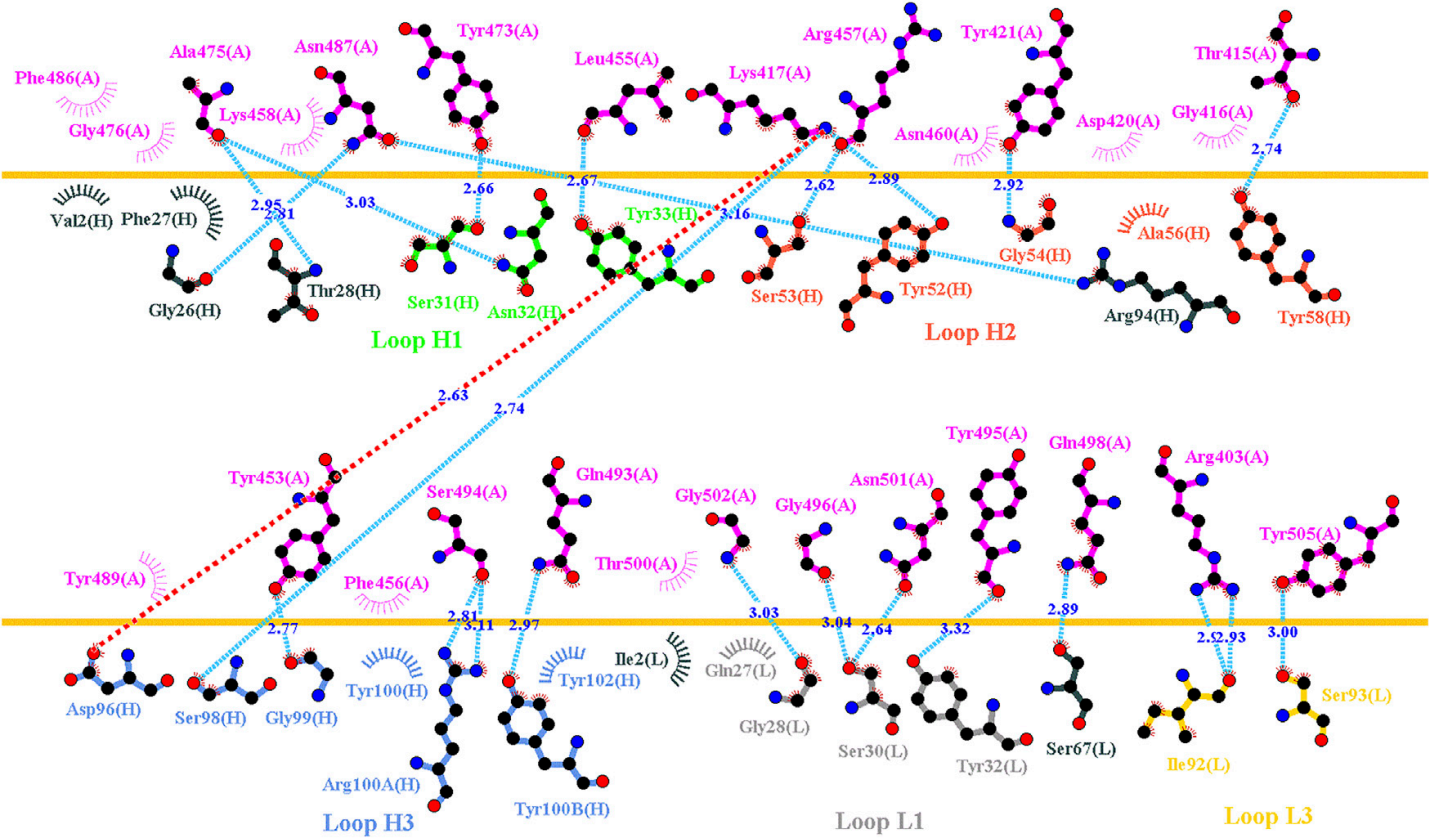
Interactional network of the RBD and CA1-B12 antibody analyzed using LIGPLOT. LIGPLOT was employed to examine the interaction network of the SARS-CoV-2 RBD and CA1-B12. The RBD and light and heavy chains of CA1-B12 were labeled A, H, and L, respectively. The golden yellow line denotes the interface of the RBD–CA1-B12 complex. The interactive residues of the RBD are labeled and colored magenta. The salt-bridges, hydrogen bonds and hydrophobic interactions are represented by red, cyan dashes and arcs with spokes radiating toward the ligand atoms with which they are contact, respectively.

**FIGURE 7:**
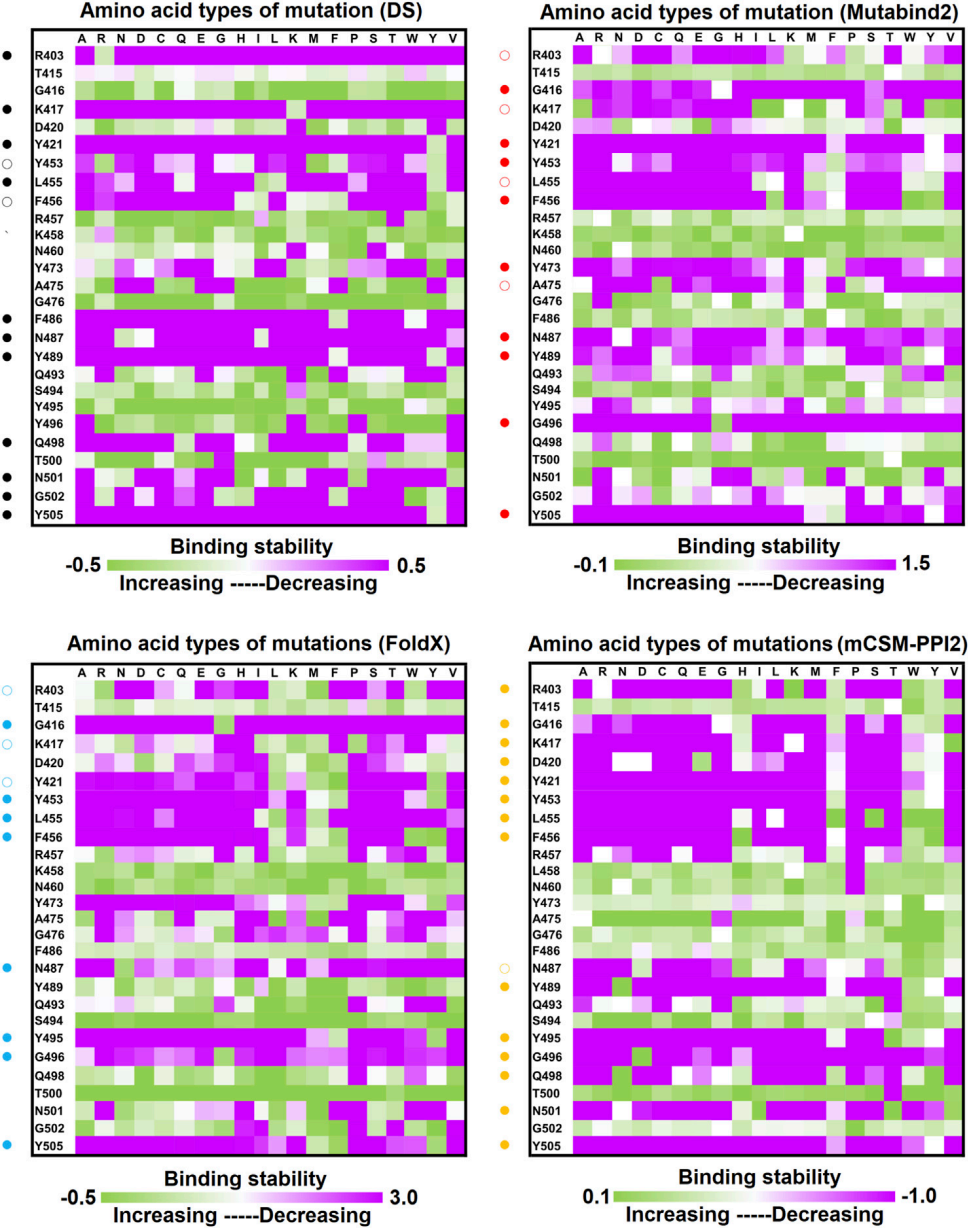
Mutational heatmaps of RBD targeting antibody CA1-B12. The mutational binding stabilities of RBD variants to CA1-B12 were estimated using FoldX, MutaBind2, DS 3.5, and mCSM-PPI2. The unit of binding stability was kcal/mole, and the obtained values were employed to create the heatmaps. Green (increased stability) and purple (reduced stability) were used to generate a color gradient and were applied in each box. In each heatmap, the moderately and significantly reduced stabilities were labeled with hollow and solid circles, respectively.

**FIGURE 8:**
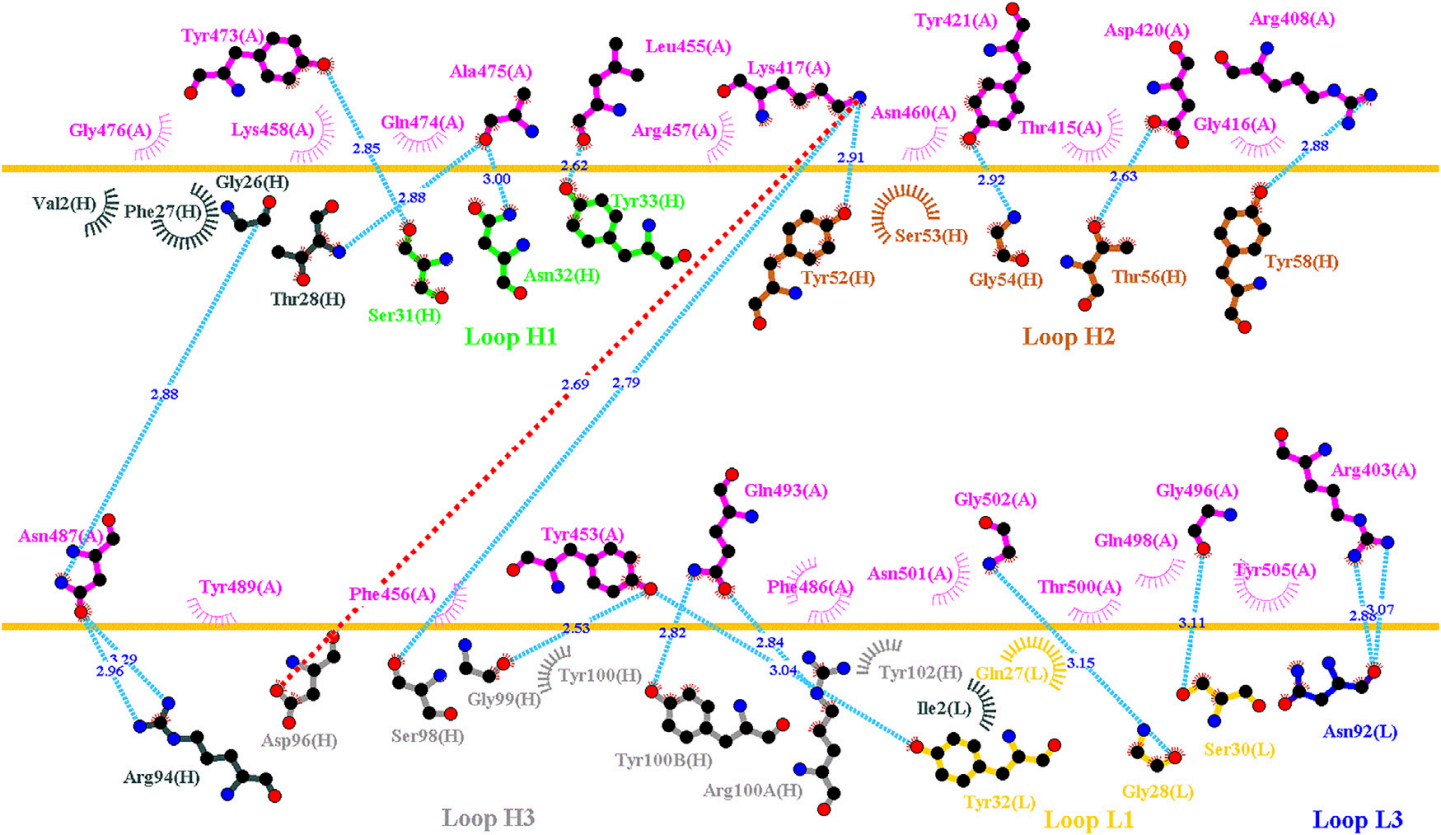
Interactional network of the RBD and CA1-B3 antibody analyzed using LIGPLOT. LIGPLOT was employed to examine the interaction network of the SARS-CoV-2 RBD and CA1-B3. The RBD and light and heavy chains of CA1-B3 are labeled A, H, and L, respectively. The golden yellow line denotes the interface of the RBD–CA1-B3 complex. The interactive residues of the RBD were labeled and colored magenta. The salt-bridges, hydrogen bonds and hydrophobic interactions are represented by red, cyan dashes and arcs with spokes radiating toward the ligand atoms with which they are contact, respectively.

**FIGURE 9:**
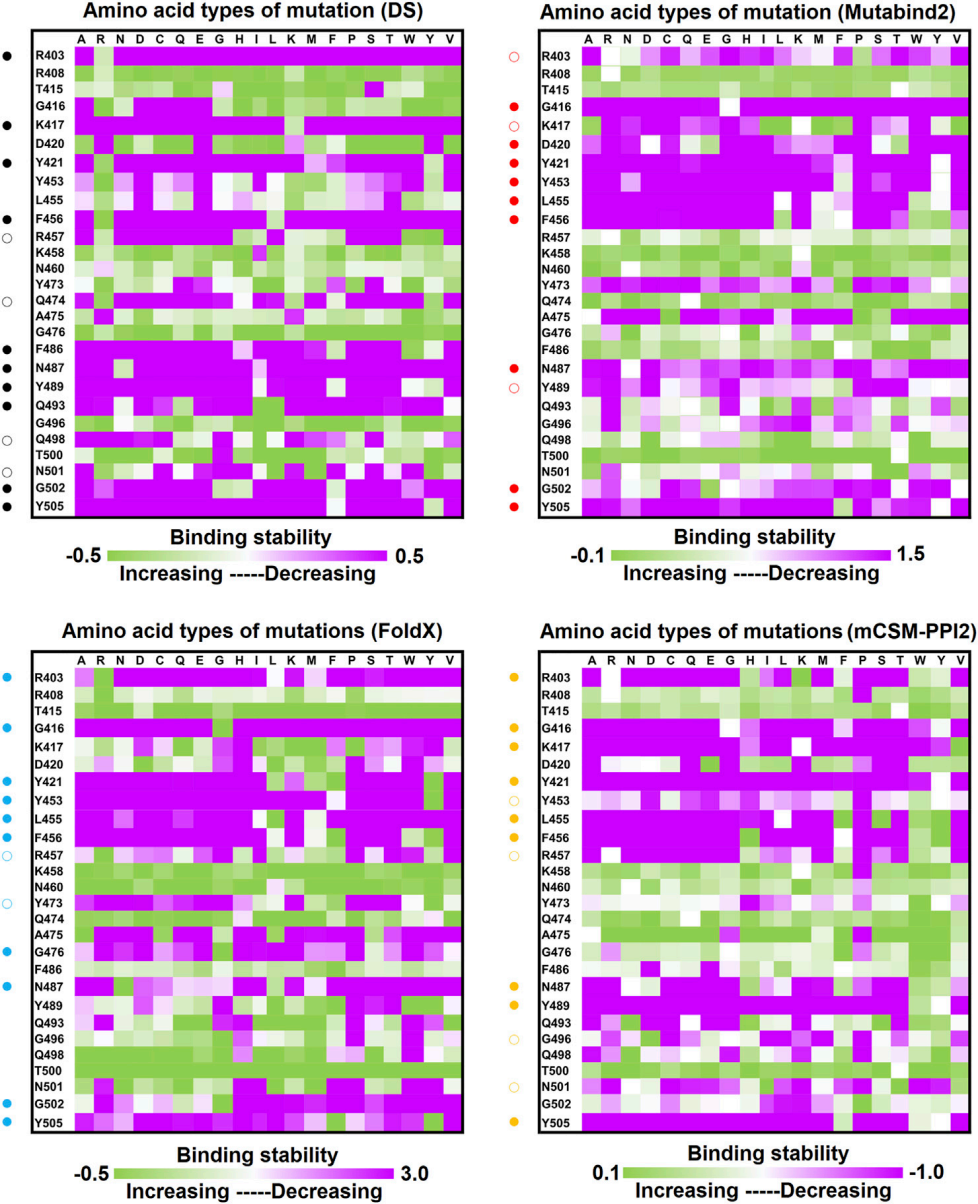
Mutational heatmaps of the RBD targeting antibody CA1-B3. The mutational binding stabilities of RBD variants to CA1-B3 were estimated using FoldX, MutaBind2, DS 3.5, and mCSM-PPI2. The unit of binding stability was kcal/mole, and the obtained values were employed to create the heatmaps. Green (increased stability) and purple (reduced stability) were used to generate a color gradient and were applied in each box. In each heatmap, the moderately and significantly reduced stabilities were labeled with hollow and solid circles, respectively.

C1A-B12 (K_D_ = 4 nM) and C1A-B3 (K_D_ = 70.6 nM), potent neutralizing antibodies targeting SARS-CoV-2, were isolated from COVID-19 convalescents (Clark et al., 2020). Figure 1D depicts the complex structure of RBD–C1A-B12. The detailed molecular interactions of C1A-B12 to RBD were analyzed and are presented in Figure 6. Structurally, RBD interacted with C1A-B12 through the formation of hydrogen bonds (R403, T415, K417, Y421, Y453, L455, R457, Y473, A475, N487, Q493, S494, Y495, G496, Q498, N501, G502, and Y505) and hydrophobic contact (G416, D420, F456, K458, N460, G476, F486, Y489, and T500). The single-point mutations at these interactive RBD residues were examined in terms of their binding stability. As presented in Figure 7 and Table 1, the mutations at residues F456, G416, G496, K417, L455, N487, N501, Q498, R403, Y421, Y453, Y489, Y495, and Y505 did not promote favorable binding to C1A-B12. The mutational effects of the RBD variants on the interactions with the C1A-B3 antibody were also investigated. The structure of the C1A-B3 antibody in complex with the RBD is illustrated in Figure 1F. As revealed through LIGPLOT analysis (Figure 8), the epitope residues of the RBD were A475, D420, G496, G502, K417, L455, N487, Q493, R403, R408, Y421, Y453, and Y473 (forming hydrogen bonds) and residues T415, G416, F456, R457, K458, N460, Q474, G476, Y489, Q498, T500, N501, and Y505 (through hydrophobic interaction). Accordingly, the binding stability of the RBD toward C1A-B3 was systematically analyzed through the individual mutation of these interactive residues. We observed that the single-point mutation at residues R403, G416, K417, Y421, Y453, L455, F456, N487, Y489, G502, and Y505 led to the destabilized binding of C1A-B3 to RBD (Figure 9 and Table 1).

### Effect of Mutation on the Binding Stability of the SARS-CoV-2 RBD Targeting Convalescent Antibody 47D1

**FIGURE 10:**
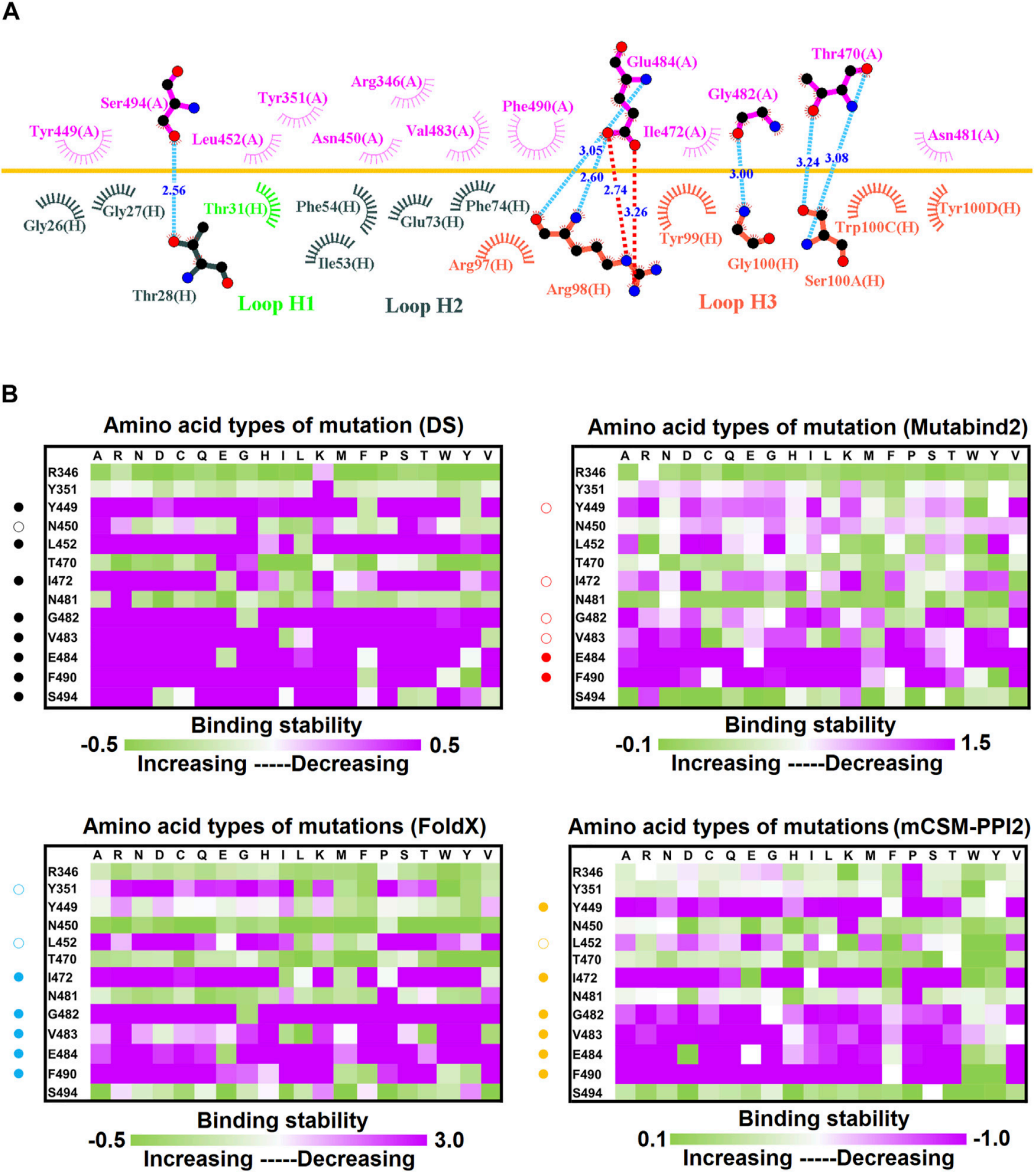
Mutational heatmaps of the RBD targeting antibody 47D1. **(A)** LIGPLOT was employed to examine the interaction network of the SARS-CoV-2 RBD and 47D1. The RBD and light and heavy chains of 47D1 were labeled A, H, and L, respectively. The golden yellow line denotes the interface of the RBD–47D1 complex. The interactive residues of the RBD are labeled and colored magenta. The hydrogen salt-bridges, bonds and hydrophobic interactions are represented by red, cyan dashes and arcs with spokes radiating toward the ligand atoms with which they are contact, respectively. **(B)** The mutational binding stabilities of RBD variants to 47D1 were estimated using FoldX, MutaBind2, DS 3.5, and mCSM-PPI2. The unit of binding stability was kcal/mole, and the obtained values were employed to create the heatmaps. Green (increased stability) and purple (reduced stability) were used to generate a color gradient and were applied in each box. In each heatmap, the moderately and significantly reduced stabilities were labeled with hollow and solid circles, respectively.

The neutralization of the convalescent antibody 47D1 (2 nM) was achieved through the hindering of RBD–ACE2 binding (Zhou X. et al., 2021). The crystal structure of RBD–47D1 is presented in Figure 1E. The LIGPLOT analysis revealed that the RBD residues T470, G482, E484, and S494 interact with 47D1 through hydrogen bonding, whereas the RBD residues R346, Y351, Y449, N450, L452, I472, V483, N481, and F490 hydrophobically interact with 47D1 (Figure 10A). Single-point mutation of the aforementioned residues was performed, followed by determination of their binding stability. The result indicated that the mutations on residues Y449, L452, I472, G482, V483, E484, and F490 were largely unfavorable for binding to 47D1 (Figure 10B).

## Discussion

The novel virus SARS-CoV-2 spread rapidly around the globe, leading to unprecedented health and economic crises. The global SARS-CoV-2 pandemic and its devastating effects are ongoing owing to the paucity of effective therapeutics. COVID-19 infection is mainly mediated through binding of the RBD of SARS-CoV-2 to ACE2 in human cells (Walls et al., 2020; Wang et al., 2020; Zhou et al., 2020). Consequently, the functionally essential RBD is a key target in the development of drugs and vaccines. Several neutralizing antibodies working against SARS-CoV-2 have been developed (Jiang et al., 2020; Pinto et al., 2020; Rogers et al., 2020; Xiaojie et al., 2020; Zhou X. et al., 2021; Lau et al., 2021; Lu et al., 2021), and neutralizing antibodies targeting SARS-CoV-2 have been isolated from convalescent patients (Clark et al., 2020; Hoffmann et al., 2020; Kreye et al., 2020; Wang P. et al., 2021; Zhou D. et al., 2021; Zhou X. et al., 2021; Kim et al., 2021; Planas et al., 2021; Yuan et al., 2021). Most of the antibodies targeting the RBD prevent the entry and replication of SARS-CoV-2 (Huo et al., 2020; Ju et al., 2020; Pinto et al., 2020; Rogers et al., 2020; Xiaojie et al., 2020; Zhou X. et al., 2021; Lu et al., 2021; Rapp et al., 2021). Moreover, the binding level of the RBD to antibodies in the serum is correlated with the neutralizing activity in patients with COVID-19 (Wang P. et al., 2021). However, the evolving rate of SARS-CoV-2 in the current pandemic equates to one to two mutations per month (Callaway, 2020). RBD mutations in particular allow SARS-CoV-2 and circulating strains to escape antibody neutralization (Andreano et al., 2020; Weisblum et al., 2020a; Huo et al., 2020; Starr et al., 2020; Greaney A. J. et al., 2021; Garcia-Beltran et al., 2021b; Greaney A. J. et al., 2021; Eguia et al., 2021). Thus, to successfully combat these immune-escaping variants, the potential RBD mutations that destabilize binding with convalescent antibodies must be investigated.

In our previous study, we made efforts to identify the important residue mutations on SARS-CoV-2 RBD which play critical roles in eroding the neutralizing immunities through computational analyses (Tsai et al., 2021). We analyzed putative mutational effects of RBD on binding to two developed nanobodies (H11-D4 (PDB ID: 6YZ5) and VH1-2-15 (PDB ID: 7L5B)), two synthetic nanobodies (MR17 (PDB ID: 7C8W) and SR4 (PDB ID: 7C8V)) and one Fab (P2B-2F6 (PDB ID: 7BWJ)). We found that the interactive residues of RBD (Y449, L452, L455, E484, Y489, F490, L492, Q493, and S494) can be hotspots conferring the ability to neutralizing antibody escape. These results provide valuable information of the mutational effects of RBD variants on interacting with developed and synthetic SARS-CoV-2-neutralizing antibodies. In addition to these man-made neutralizing antibodies, there are antibodies determined from COVID-19 convalescent patients (Piccoli et al., 2020; Greaney A. J. et al., 2021; Rosati et al., 2021; Tada et al., 2021). Especially, several potent SARS-CoV-2-neutralizing monoclonal antibodies were isolated from convalescent individuals (Clark et al., 2020; Kreye et al., 2020; Wu et al., 2020; Zhou X. et al., 2021; Kim et al., 2021; Xie et al., 2021). These monoclonal antibodies target the RBD of SARS-CoV-2 and compete with its binding to ACE2 preventing virus entry and replication. However, growing evidence show that escape mutations, reducing the neutralizing activity of antibodies in the convalescent plasma of COVID-19 patients, could weaken the effectiveness of antibodies and vaccines under developments (Weisblum et al., 2020a; Greaney A. J. et al., 2021; Wang Z. et al., 2021; Wibmer et al., 2021). To develop potent antiviral prophylaxis combating the SARS-CoV-2 variants which could lead neutralization escape from the convalescent antibodies, it is imperative to investigate the mutational effects of SARS-CoV-2 RBD on binding to these convalescent antibodies. Nowadays, the complex structures of RBD-convalescent antibodies are available; this make it feasible to explore the mutations of RBD rendering the capability of neutralizing antibody escape. Therefore, here we aim to identify the possible immune escape hotspots of RBD targeting convalescent antibodies by using similar methodology of our previous work.

In this study, we systematically and comprehensively investigated the changes in the binding stability of RBD variants to convalescent antibodies. The results revealed that single-point mutations at the RBD residues E484, F490, G447, L452, L492, N448, N450, S439, S494, Y449, and Y451 considerably destabilized the interactions with the CV07-270 convalescent antibody (Figure 2 and Supplementary Tables S3, S9, S15, S21). Furthermore, the RBD residues S349, G447, N448, Y449, N450, and E484 mainly interacted with CV07-270 through hydrogen bonding; L452, F490, L492, and S494 interacted with the antibody through hydrophobic contact. Similarly, the binding stability to the convalescent antibody B38 was disrupted when RBD residues F456, F486, Y489 (contributing to hydrophobic contact), R403, K417, D420, Y421, L455, Y473, N487, G496, N501, G502, and Y505 (forming hydrogen bonds) were mutated through replacement with other amino acids (Figure 4 and Supplementary Tables S4, S10, S16, S22). Some RBD mutations interfered with its interaction to the CT-P59 convalescent antibody (Figure 5 and Supplementary Tables S5, S11, S17, S23); the residues Y453, F486, and Q493 were mostly connected through hydrogen bonding, whereas Y449, L452, F456, G485, F490, and L492 were connected through hydrophobic interaction. Moreover, the amino acid replacements impaired RBD binding to the C1A-B12 convalescent antibody. The mutations at G416, F456, 489 (contributing to hydrophobic contact), R403, K417, Y421, Y453, L455, N487, Y495, G496, N501, and Y505 (forming hydrogen bonds) significantly disrupted the binding stability (Figure 6 and Supplementary Tables S6, S12, S18, S24). The mutations at R403, K417, Y421, Y453, L455, N487, G502 (essentially hydrogen bonds), G416, F456, Y489, and Y505 (mostly hydrophobic interactions) disrupted the interactions between the RBD and C1A-B3 convalescent antibody (Figure 9 and Supplementary Tables S7, S13, S19, S25). We also investigated the changes in the binding of the convalescent antibody 47D1 to the RBD variants. The RBD residues G482 and E484 interacting with 47D1 primarily through hydrogen bonding, and residues Y449, L452, I472, V483, and F490 connecting with 47D1 through hydrophobic interaction disrupted the binding to the antibody when subjected to single-point mutations (Figure 10 and Supplementary Tables S8, S14, S20, S26). Furthermore, we analyzed and integrated the key RBD residues with single-point mutations that destabilize binding to most of the convalescent antibodies. We determined that the RBD residues E484, F456, F486, F490, G416, G496, G502, K417, L452, L455, L492, N487, N501, R403, S494, Y421, Y449, Y489, Y495, and Y505 were prone to disrupt interaction with convalescent antibodies when mutated (Table 1). These residues were aromatic and/or hydrophobic, with the exceptions of E484, K417, N487, N501, R403, and S494. Notably, mutations at F490, F456, G416, G502, K417, L452, L455, Y449, N487, R403, and Y489 were concurrently observed to be unfavorable for the binding of RBD to most of the convalescent antibodies. This indicated that the immune-escaping ability of RBD may be attributable to the hotspots F456, F490, G416, G502, K417, L452, L455, N487, R403, Y449, and Y489, which disrupt interaction with convalescent antibodies, further interfering or even attenuating the immune responses.

We therefore placed our findings among the emerging variants of SARS-CoV-2 to verify the effects of the determined immune-escape hotspots R403, G416, K417, Y449, L452, L455, F456, N487, Y489, F490, and G502. The K417T variant was first observed in Japan (lineage: P.1), with K417N later identified in lineages B.1.351 and P.1 in South Africa and Brazil, respectively (Table 1). The K417N and K417T variants of the RBD destabilized interaction with convalescent antibodies B38, C1A-B12, and C1A-B3 in our study. Furthermore, we observed that the variants of L452 conspicuously decreased the binding stability of the RBD to convalescent antibodies CV07-270, CT-P59, and 47D1 in our predictions. This variant (L452R) was identified in both the United States and India (Table 1). Also, E484 variants reportedly escape from neutralizing antibodies, especially the E484K variant that emerged in several SARS-CoV-2 lineages (Table 1) and the E484Q variant in India (Weisblum et al., 2020a; Chen et al., 2021). Our computational analysis revealed that the variants of E484 significantly disrupted RBD binding to the convalescent antibodies CV07-270 and 47D1. The variants of F490 and S494 also exerted destabilizing effects on RBD binding to convalescent antibodies in our study, which is consistent with the SARS-CoV-2 variants F490S and S494P observed in the United States and Peru (Table 1). Contraction of the highly infectious and potentially lethal variant N501Y leads to a high chance of hospitalization (Leung et al., 2021), with this variant identified in the lineages of C.37, P.1, B1.1.7, and B.1.351 (Singh et al., 2021; Tang et al., 2021; Tegally et al., 2021). This report corroborates our finding that variants of N501 destabilized the binding stability of RBD to convalescent antibodies B38 and CA1-B12. The RBD variants conferring the immune-escaping ability were also investigated using experimental methods (Andreano et al., 2020; Weisblum et al., 2020a; Huo et al., 2020; Starr et al., 2020; Greaney A. J. et al., 2021; Garcia-Beltran et al., 2021b; Greaney A. J. et al., 2021). The reporter virus VSV/SARS-CoV-2 was examined in terms of the mutational effects of RBD variants, with the results revealing that E484K, F490L, and Q493 K/R exert resistance to neutralizing antibodies (Weisblum et al., 2020b). Li et al. also reported that L452R and F490L are resistant to some neutralizing antibodies and sera from convalescent patients (Andreano et al., 2020). Moreover, variants E484K, K417N, K417T, and N501Y were able to escape antibody-induced neutralization, as observed in an emerging SARS-CoV-2 isolate in South Africa (B.1.351; (Garcia-Beltran et al., 2021a). Another study conducted yeast display and deep mutational scanning of the SARS-CoV-2 RBD and demonstrated that single-point mutations at sites E484, F456, F486, F490, K417, L452, L455, N450, and Q493 escaped neutralizing antibodies (Greaney A. J. et al., 2021). Our results indicated that the RBD residues F456, F490, G502, G416, K417, L452, L455, N487, R403, Y489, and Y449 were essential sites; following mutation, their variants profoundly disrupted interaction with neutralizing antibodies. In particular, the results of the hotspots F456, F490, L452, L455, and K417 and destabilizing residues E484, F486, and N501 identified in this study are corroborated through reports on emerging SARS-CoV-2 variants. This robust collection of evidence strongly supports the identification of these hotspots and the precision and reliability of our computational study. Therefore, these results can assist in the further development and application of potent antibody and vaccine therapeutics to combat emerging SARS-CoV-2 variants.

**FIGURE 11:**
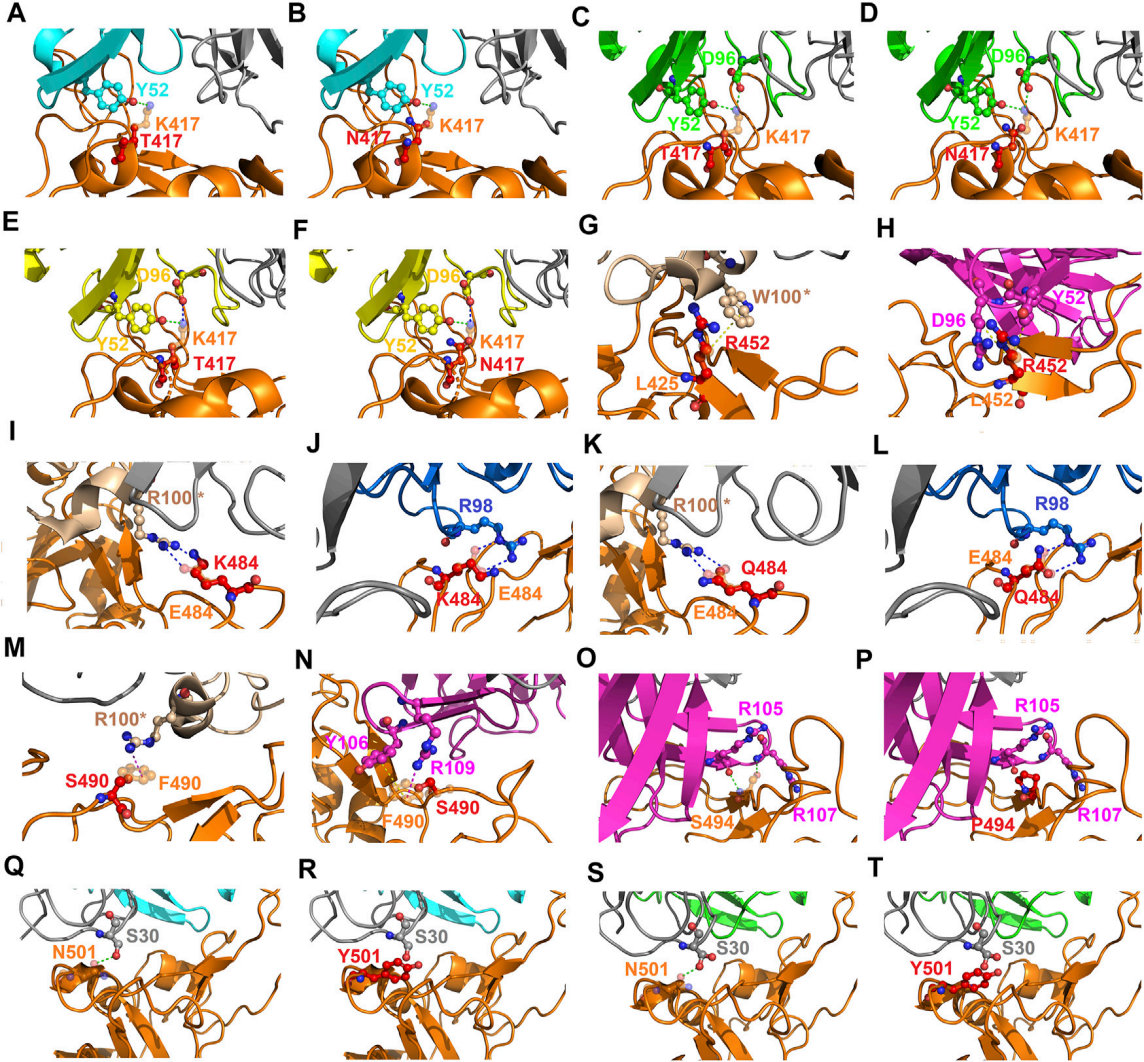
Mutational effects contributing to the modes of action of emerging SARS-CoV-2 RBD variants. **(A)** An overlapped view of the intermolecular interactions of the B38 antibody to K417 (orange chain) and mutant T417 (red chain) of the RBD. **(B)** A superimposition of K417 (orange chain) and mutant N417 (red chain) of the RBD bound with antibody B38. **(C)** The distinct interactions of K417 (orange chain) and mutant T417 (red chain) of the RBD targeting the CA1-B12 antibody. **(D)** A superimposition of K417 (orange chain) and mutant N417 (red chain) of the RBD interacting with the CA1-B12 antibody. **(E)** An overlapped view of K417 (orange chain) and mutant T417 (red chain) of the RBD bound with CA1-B3. **(F)** A superimposition of K417 (orange chain) and mutant N417 (red chain) of the RBD targeting the CA1-B3 antibody. **(G)** A comparison of L452 (orange chain) and mutant R452 (red chain) of the RBD binding to the CV07-270 antibody. **(H)** The distinct interactions of L452 (orange chain) and mutant R452 (red chain) of the RBD targeting the CT-P59 antibody. **(I)** A superimposed view of E484 (orange chain) and mutant K484 (red chain) of the RBD interacting with the CV07-270 antibody. **(J)** An overlapped view of E484 (orange chain) and mutant K484 (red chain) of the RBD binding to 47D1. **(K)** A comparison of E484 (orange chain) and mutant Q484 (red chain) of the RBD in complex with the CV07-270 antibody. **(L)** The differences between E484 (orange chain) and mutant Q484 (red chain) of the RBD interacting with the 47D1 antibody. **(M)** The deviations between F490 (orange chain) and mutant S490 (red chain) of the RBD targeting the CV07-270 antibody. **(N)** A superimposition of F490 (orange chain) and mutant S490 (red chain) of the RBD bound with the CT-P59 antibody. **(O)** The interactions of S494 (orange chain) and **(P)** mutant P494 (red chain) of the RBD binding to the CT-P59 antibody. **(Q)** The interactions of N501 (orange chain) and **(R)** mutant Y501 (red chain) of the RBD targeting the B38 antibody. **(S)** The interactions of N501 (orange chain) and **(T)** mutant Y501 (red chain) of the RBD bound to the CA1-B12 antibody. The structures of proteins are presented with ribbons and dashed lines colored green, blue, red, and yellow denoting a hydrogen bond, cation–π interaction, hydrophobic interaction, and electrostatic interaction, respectively.

The detailed interactions between RBD variants and convalescent antibodies were analyzed to determine possible modes of actions relating to the analyzed hotspots. The RBD residue K417 interacted with Y52 and/or D96 of the convalescent antibodies B38, CA1-B12, and CA1-B3 through hydrogen bonding (Figures 11A–F). However, the hydrogen bonds were disrupted when K417 was replaced with threonine or arginine amino acids, which further destabilized the binding stability of the RBD to the antibodies. The RBD residue L452 interacted with W100 of CV07-270 and Y52 and D96 of CT-P59 (Figures 11G,H) through hydrophobic contact. These hydrophobic interactions were disturbed when L452 was mutated to an arginine amino acid, which further weakened the binding stability of RBD to CV07-270 and CT-P59. Structurally, the charge–charge interactions often make considerable contributions to the binding stability of protein complexes. The residue E484 electrostatically interacted with R100 and R98 of CV07-270 and 47D1, respectively, but this interaction was disrupted when E484 was substituted with lysine or glutamine residues, interfering the convalescent antibody-induced neutralization of RBD (Figures 11I,J). In addition, the aromatic RBD residue F490 exerted hydrophobic and cation–π interactions contributing to binding to the antibodies (Figures 11M,N). These interactions do not occur in the complex structure of the F490S variant, which therefore explains the reduced binding stability of RBD to the antibodies. Furthermore, the RBD residue S494 interacting with R105 and R107 of CT-P59 through hydrogen bonding was disrupted when S494 was mutated to a proline amino acid, thus weakening the binding affinity (Figures 11O,P). In addition, the N501Y variant exhibited considerable concurrency in several countries. In the complex structure of RBD–B38 and RBD–CA1-B12, N501 forms a hydrogen bond with S30. Nevertheless, the bulky aromatic ring of N510Y collides with S30 and other residues at the interface, hence decreasing the binding stability of RBD to convalescent antibodies B38 and CA1-B12 (Figures 11Q–T).

**FIGURE 12:**
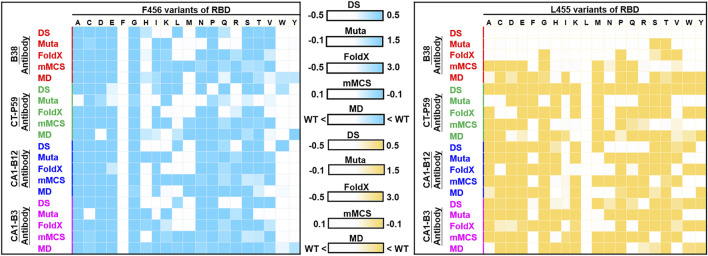
Comparison of MD and non-MD predictions of the mutational effects of F456 and L455 RBD variants targeting convalescent antibodies. The unit of binding stability (DS, Mutabind2 (Muta), FoldX, and mMCS-PPI2 (mMCS)) and affinity (MD) were in kcal/mole, and the obtained values were employed to create the heatmaps. In DS, Muta, FoldX, and mMCS, white (increased stability) and blue/yellow (reduced stability) are used to generate a color gradient and were applied in each box. In MD results, the predicted binding affinity smaller than that of wild-type (WT) is colored in blue/yellow.

In this study, we employed four programs/methods (FoldX, Mutabind2, mMCS-PPI2, and DS) to simultaneously evaluate the mutational binding energy of variants of RBD targeting distinct convalescent antibodies. Notably, mutations could change protein conformations in the binding interface, and this may have significant effects on antibody binding. While the used four programs do not appear to consider protein conformational flexibility, ensemble predictions, and explicit solvent effects. The molecular dynamic (MD) simulations to complexes can incorporate protein flexibility, and explicit solvent effects which are important for electrostatics and hydrogen bonding. Although the energy minimization is included during the calculations of the used program, such as Mutabind2, the standard MD simulation is not incorporated into the predictions of these methods. Hence, to confirm the reliability and precision of the used strategy and methodology in this study, we further employed MD simulations for the strongest mutation hotspots L455 and F456 to again estimate their mutational effects on RBD’s binding to convalescent antibodies. The corresponding mutant and wild-type structures of RBD complexed with convalescent antibodies (B38, CT-P59, CA1-B3 and CA1-B12) were subjected to MD simulations. All the trajectory profiles of total energy changes as functions of MD simulation time were shown in Supplementary Figures S1-S16. Besides, the initial and final conformations before and after MD simulations were compared and shown in Supplementary Figures S17-S32. Furthermore, the final conformations of each complex structure after the MD simulations were subjected to CSM-AB to calculate the binding affinity. The results showed that over 80% of the L455 and F456 variants of RBD displayed decreased binding affinity to convalescent antibodies when compare to that of the wild-type RBD (Supplementary Tables S27,S28). These observations are consistent with the predicted results of the four used programs (FoldX, Mutabind2, mMCS-PPI2, and DS) (Figure 12), providing evidence to support the proposed immune escape hotspots and demonstrating the reliability of the predictions in this study.

In addition to our research, there are approaches conducted to predict the possible mutations in SARS-CoV-2. Rodriguez-Rivas et al. used the epistatic models to predict the mutable sites of proteins and epitopes in SARS-CoV-2 (Rodriguez-Rivas et al., 2022). In their sequence-based predictions, the predicted mutability of SARS-CoV-2 RBD was observed to be well correlated with experimentally determined protein stability. Also, their study identified mutable positions (K417, L452, S477, T478, V483, E484, and N501) of RBD in which current variants of concern are highly overrepresented. Consistently, residues, K417, L452, E484, and N501 were predicted as possible immune escape hotspots in our study. On top of that, Taft and colleagues have performed the predictive profiling of SARS-CoV-2 variants based on protein sequences (Joseph M. Taft, 2021). They built a deep machine learning to integrate and analyze a huge sequence space of SARS-CoV-2 variants, computationally estimating their effects on antibody neutralization. Their DML study can efficiently predict new and possible SARS-CoV-2 variants, but the sequence-based prediction without consideration of structural properties, prime determinants of protein-protein binding affinity, could erode the reliability. In our study, we systematically conducted *in silico* mutagenesis on the structure of RBD complexed with convalescent antibodies to comprehensively predicted the mutational impacts of its variants on binding stability. The results of our structure-based predictions, consistent with experimental measures and some circulating variants, cab be complementary to those of sequence-based predictions. Still, the used method of our study is restricted to give insight into the conformation-destabilizing mutations of allosteric residues that may also result in escape from neutralizing antibodies. In this study, the functionally important residues of RBD which interact and bind to convalescent antibody were mainly subjected to single point mutations. The binding stabilities of the obtained RBD variants in complex with convalescent antibody were predicted to evaluate the mutational effects. We found that single point mutation at specific residues of RBD indeed apparently disrupted the binding of RBD to antibodies. Theses residues of RBD are of great potential to be hotspots which could escape the recognition by convalescent antibodies. Our study mainly aims to identify the immune-escaping hotspot from the binding interface of RBD to convalescent antibody, while the allosteric residues of RBD that result in escape from neutralizing antibodies could be another research scope for future work. In summary, we explored and revealed the probable immune-escaping hotspots from the interface residues of RBD interacting with convalescent antibodies. Single-point mutations at these residues that could considerably impair the interaction between the SARS-CoV-2 RBD and convalescent antibodies. Our findings are immensely beneficial for designing and developing therapeutics to combat emerging SARS-CoV-2 variants.

## Conclusion

In this study, we determined the potential residues that, after mutation, can confer the ability to escape convalescent antibody-induced neutralization on SARS-CoV-2. The complex structure-based analysis revealed that specific variants of RBD significantly impair the binding stability to convalescent antibodies. The single-point mutations at hotspots G502, G416, F456, F490, K417, L452, L455, N487, R403, Y449, and Y489 markedly destabilized binding to convalescent antibodies. The results of the immune-escaping hotspots (K417, L452, L455, F456, and F490) and destabilizing residues (E484, F486, and N501) corroborate experimental observations and clinically emerging SARS-CoV-2 variants. Our study provides insight into the structural hotspots that confer the immune-escaping ability of SARS-CoV-2 RBD in relation to convalescent antibodies with distinct modes of action. The results of our study can assist in the development of new antiviral agents to protect against the emerging variants of SARS-CoV-2.

## Data Availability

The original contributions presented in the study are included in the article/Supplementary Material, further inquiries can be directed to the corresponding author.
