# Supplementary material for: Antigen–Antibody Complex-Guided Exploration of the Hotspots Conferring the Immune-Escaping Ability of the SARS-CoV-2 RBD
Source: Front Mol Biosci. 2022 Mar 22;9:797132. doi: 10.3389/fmolb.2022.797132 (PMC8981523; doi:10.3389/fmolb.2022.797132)
Supplement: Supplementary file 1 [file DataSheet4.docx]

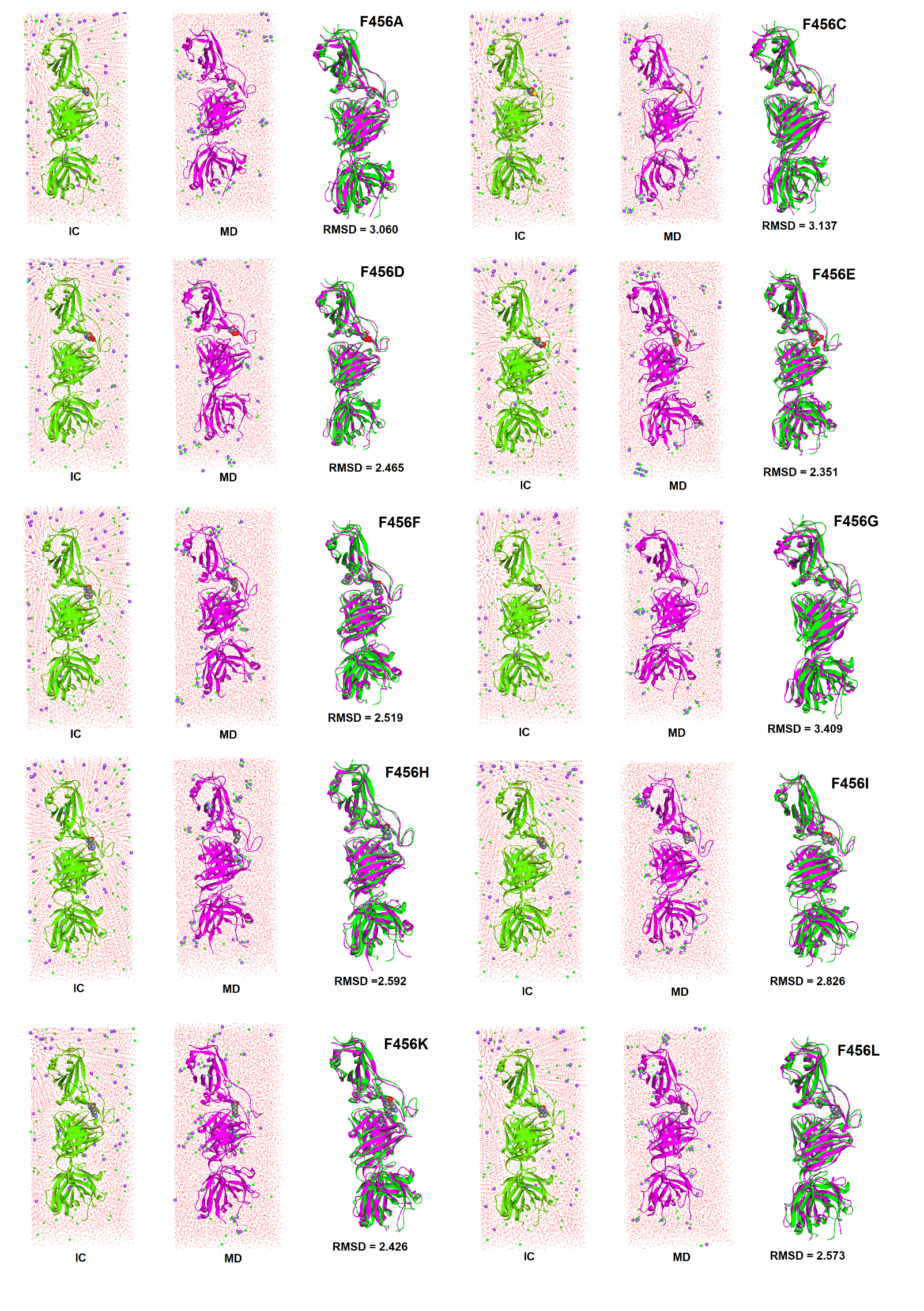


**Figure S25. The snapshots of initial and final conformations of F456 variants of RBD targeting convalescent antibody CA1-B12 (PDB ID: 7KFV)** **before and after MD simulations.** In each panel, the water molecules are shown as red spheres; the sodium and chloride ions are presented as purple and green spheres, respectively; protein structures are displayed in cartoons. The abbreviations **IC** and **MD** denote Initial Conformation and Molecular Dynamic simulation. The mutated residues of RBD are shown as spheres (colored by element). The unit of RMSD is in Å.


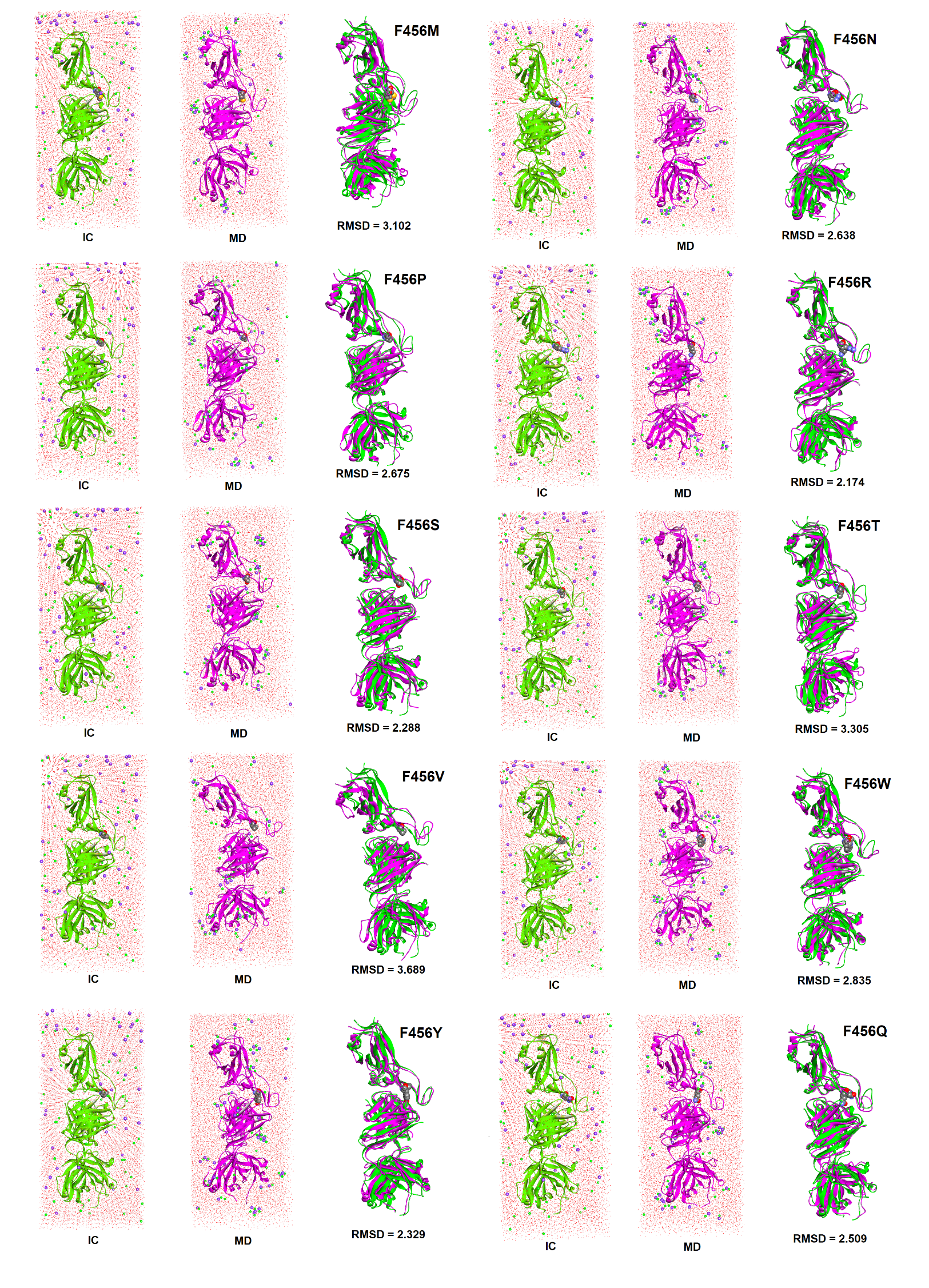


**Figure S26. The snapshots of initial and final conformations of F456 variants of RBD targeting convalescent antibody CA1-B12 (PDB ID: 7KFV)** **before and after MD simulations.** In each panel, the water molecules are shown as red spheres; the sodium and chloride ions are presented as purple and green spheres, respectively; protein structures are displayed in cartoons. The abbreviations **IC** and **MD** denote Initial Conformation and Molecular Dynamic simulation. The mutated residues of RBD are shown as spheres (colored by element). The unit of RMSD is in Å.


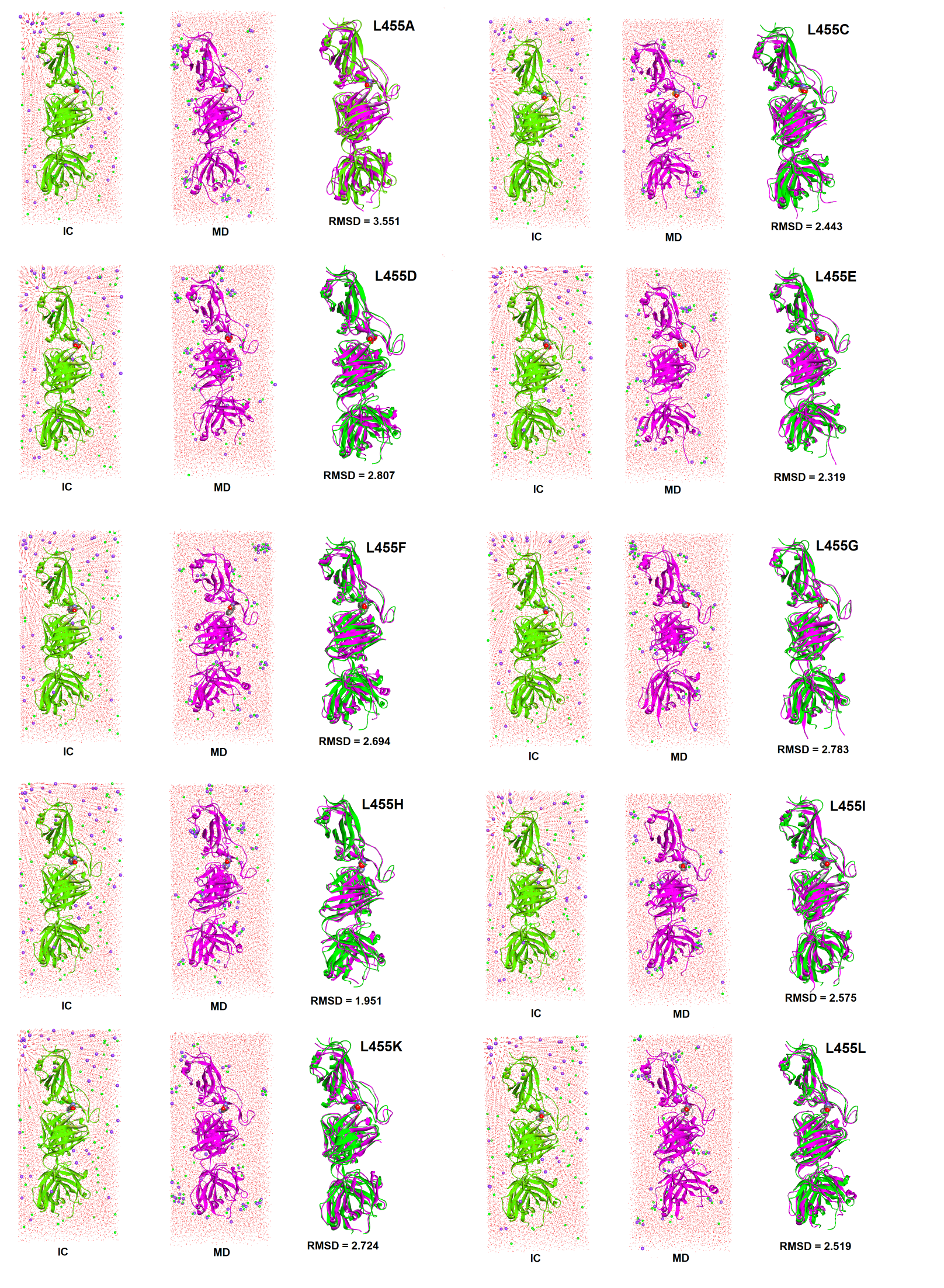


**Figure S27. The snapshots of initial and final conformations of L455 variants of RBD targeting convalescent antibody CA1-B12 (PDB ID: 7KFV)** **before and after MD simulations.** In each panel, the water molecules are shown as red spheres; the sodium and chloride ions are presented as purple and green spheres, respectively; protein structures are displayed in cartoons. The abbreviations **IC** and **MD** denote Initial Conformation and Molecular Dynamic simulation. The mutated residues of RBD are shown as spheres (colored by element). The unit of RMSD is in Å.


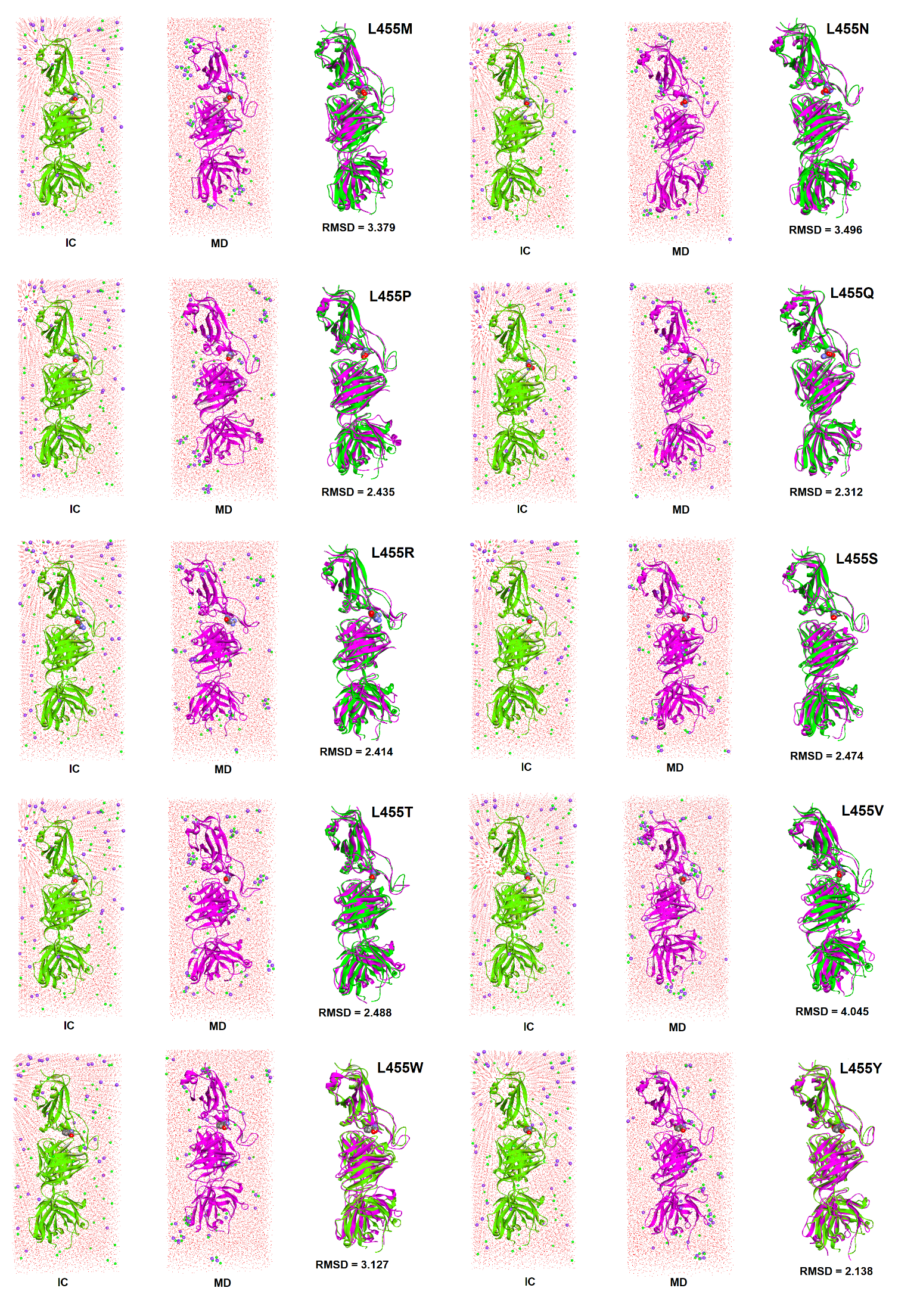


**Figure S28. The snapshots of initial and final conformations of L455 variants of RBD targeting convalescent antibody CA1-B12 (PDB ID: 7KFV)** **before and after MD simulations.** In each panel, the water molecules are shown as red spheres; the sodium and chloride ions are presented as purple and green spheres, respectively; protein structures are displayed in cartoons. The abbreviations **IC** and **MD** denote Initial Conformation and Molecular Dynamic simulation. The mutated residues of RBD are shown as spheres (colored by element). The unit of RMSD is in Å.
