# Supplementary material for: Antigen–Antibody Complex-Guided Exploration of the Hotspots Conferring the Immune-Escaping Ability of the SARS-CoV-2 RBD
Source: Front Mol Biosci. 2022 Mar 22;9:797132. doi: 10.3389/fmolb.2022.797132 (PMC8981523; doi:10.3389/fmolb.2022.797132)
Supplement: Supplementary file 3 [file DataSheet5.docx]

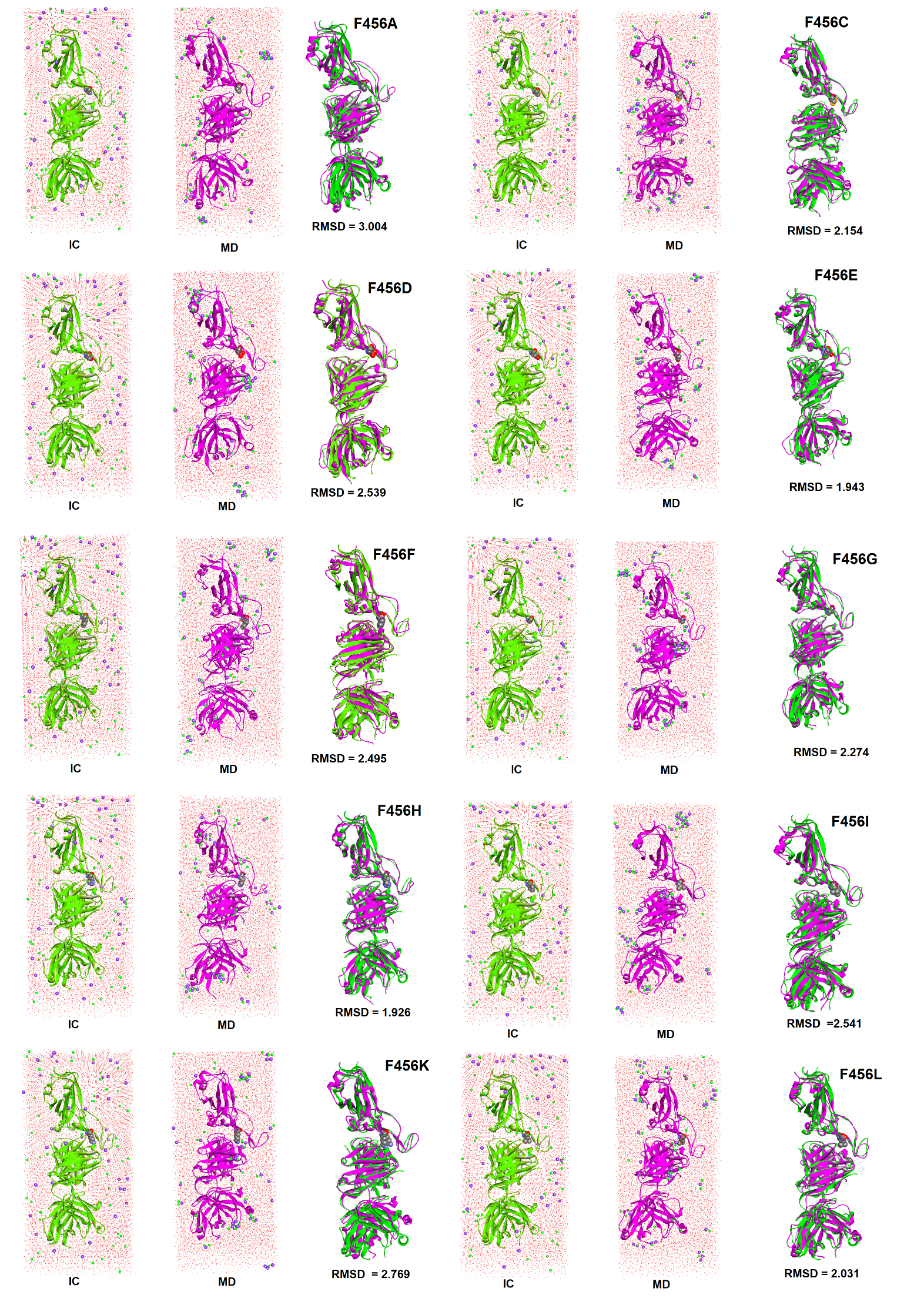


**Figure S29. The snapshots of initial and final conformations of F465 variants of RBD in complex with convalescent antibody CA1-B3 (PDB ID: 7KFW)** **before and after MD simulations.** In each panel, the water molecules are shown as red spheres; the sodium and chloride ions are presented as purple and green spheres, respectively; protein structures are displayed in cartoons. The abbreviations **IC** and **MD** denote Initial Conformation and Molecular Dynamic simulation. The mutated residues of RBD are shown as spheres (colored by element). The unit of RMSD is in Å.


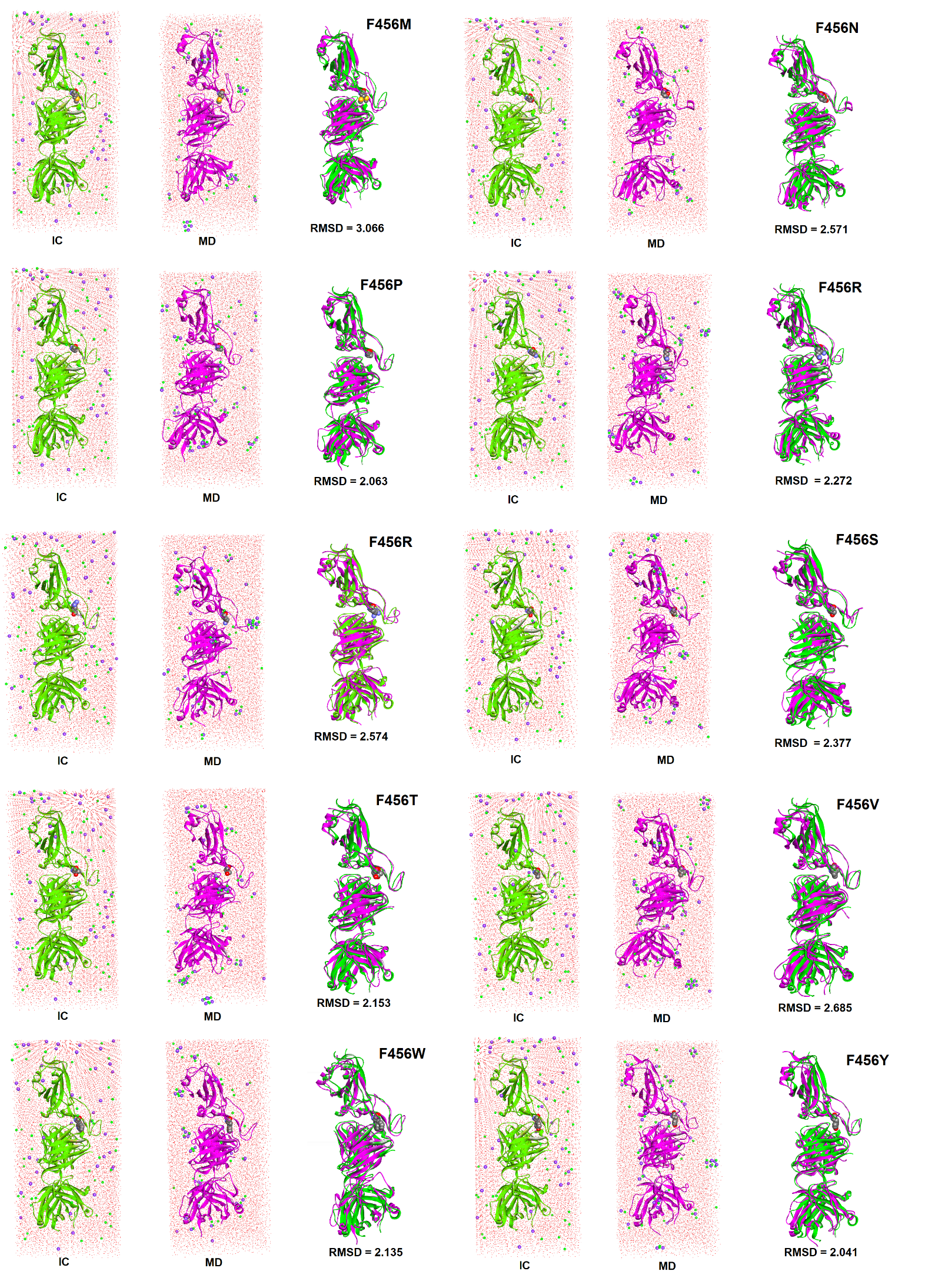


**Figure S30. The snapshots of initial and final conformations of F465 variants of RBD in complex with convalescent antibody CA1-B3 (PDB ID: 7KFW)** **before and after MD simulations.** In each panel, the water molecules are shown as red spheres; the sodium and chloride ions are presented as purple and green spheres, respectively; protein structures are displayed in cartoons. The abbreviations **IC** and **MD** denote Initial Conformation and Molecular Dynamic simulation. The mutated residues of RBD are shown as spheres (colored by element). The unit of RMSD is in Å.


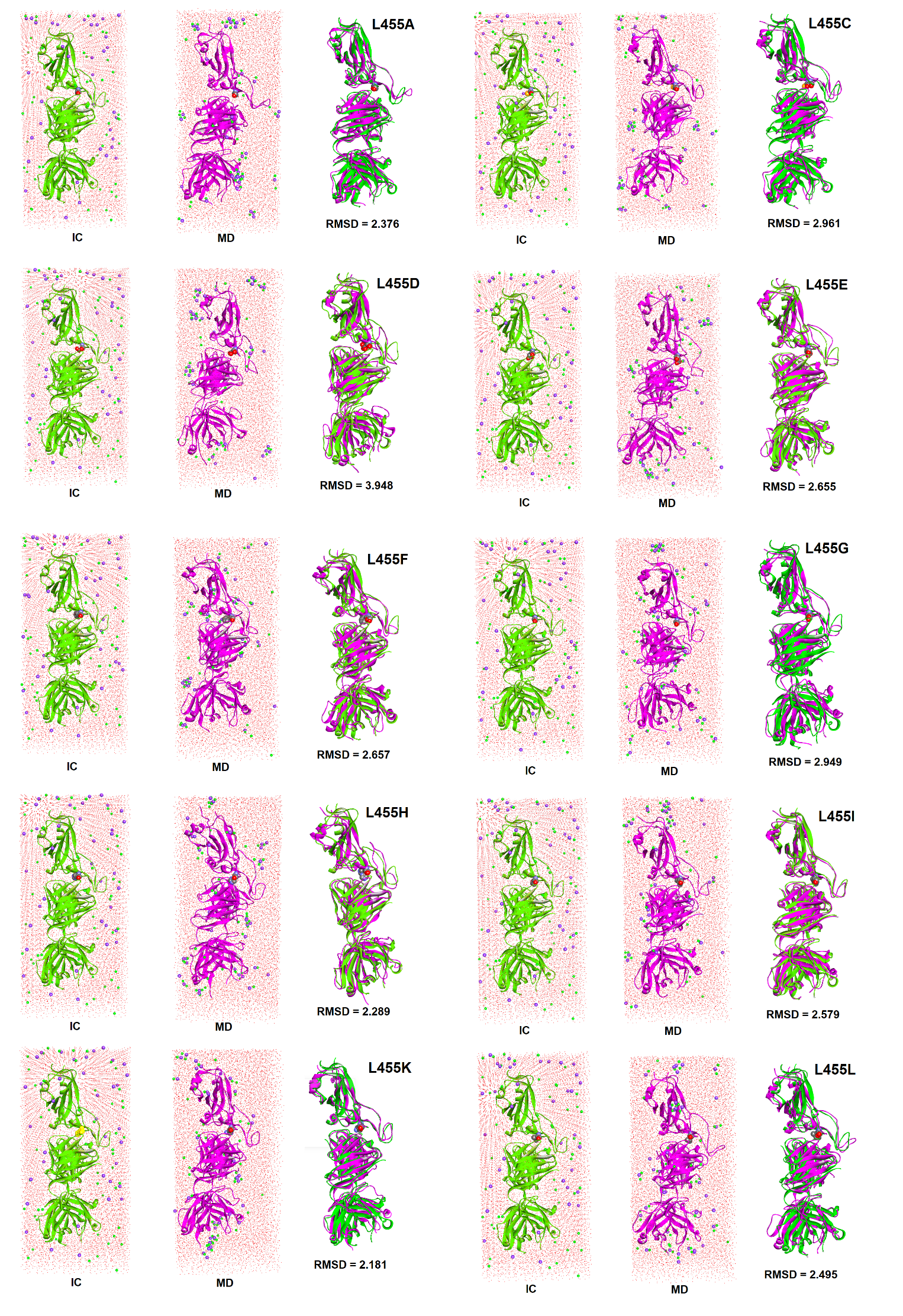


**Figure S31. The snapshots of initial and final conformations of L455 variants of RBD in complex with convalescent antibody CA1-B3 (PDB ID: 7KFW)** **before and after MD simulations.** In each panel, the water molecules are shown as red spheres; the sodium and chloride ions are presented as purple and green spheres, respectively; protein structures are displayed in cartoons. The abbreviations **IC** and **MD** denote Initial Conformation and Molecular Dynamic simulation. The mutated residues of RBD are shown as spheres (colored by element). The unit of RMSD is in Å.


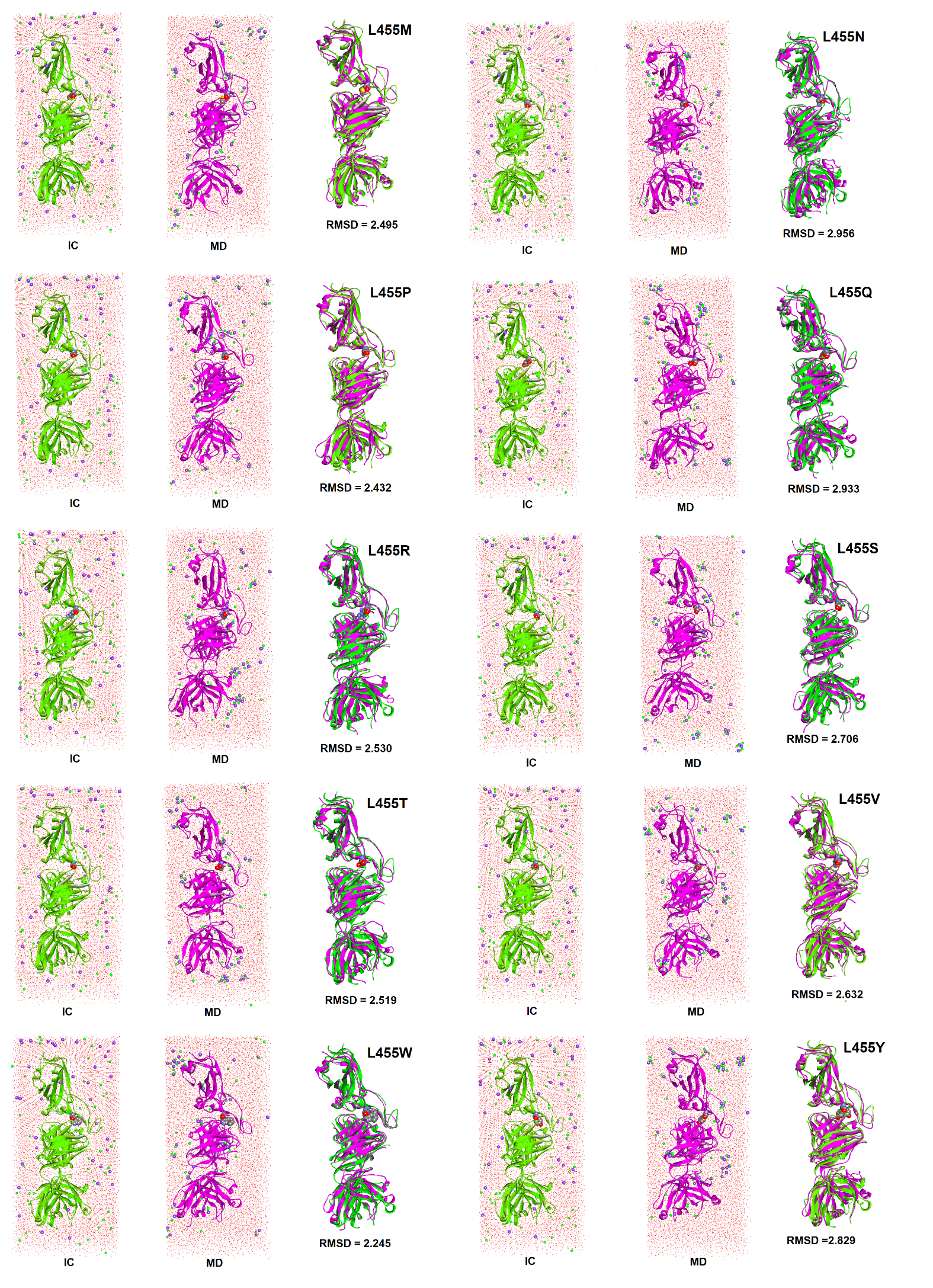


**Figure S32. The snapshots of initial and final conformations of L455 variants of RBD in complex with convalescent antibody CA1-B3 (PDB ID: 7KFW)** **before and after MD simulations.** In each panel, the water molecules are shown as red spheres; the sodium and chloride ions are presented as purple and green spheres, respectively; protein structures are displayed in cartoons. The abbreviations **IC** and **MD** denote Initial Conformation and Molecular Dynamic simulation. The mutated residues of RBD are shown as spheres (colored by element). The unit of RMSD is in Å.
