# Supplementary material for: Antigen–Antibody Complex-Guided Exploration of the Hotspots Conferring the Immune-Escaping Ability of the SARS-CoV-2 RBD
Source: Front Mol Biosci. 2022 Mar 22;9:797132. doi: 10.3389/fmolb.2022.797132 (PMC8981523; doi:10.3389/fmolb.2022.797132)
Supplement: Supplementary file 5 [file DataSheet1.docx]

**Table S1. Parameter setting for solvation of molecular dynamic simulation.**

| **Solvation model** | Explicit Periodic Boundary |
| --- | --- |
| Cell shape | Orthorhombic |
| Minimum Distance From Boundary | 7.0 (Å) |
| Radius of Sphere | 20 (Å) |
| **Add Counterion** | true |
| Cation Type | Sodium |
| Anion Type | Chloride |
| Random Seed | 314159 |

**Table S2. Parameter setting for standard dynamics cascade of molecular dynamic simulation.**

| **Minimization** |  |  | **Production** |  |
| --- | --- | --- | --- | --- |
| Algorithm | Steepest Descent |  | Simulation Time (ps) | 5000 |
| Max Steps | 1000 |  | Time Step (fs) | 2 |
| RMS Gradient | 1 |  | Target Temperature | 300 |
| Constraints | none |  | Save Results interval (ps) | 2 |
| **Minimization2** |  |  | Save Restart File | True |
| Algorithm | Adopted Basis NR |  | Constraints | none |
| Max Steps | 2000 |  | Type | NTP |
| RMS Gradient | 0.1 |  | TMass | 1000 |
| Constraints | none |  | PMass | 1000 |
| **Heating** |  |  | PGamma | 25 |
| Simulation Time (ps) | 4 |  | Reference Pressure | 1 |
| Time Step (fs) | 2 |  | Nonbond List Radius | 14 |
| Initial Temperature | 50 |  | Nonbond Higher Cutoff Distance | 12 |
| Target Temperature | 300 |  | Nonbond Lower Cutoff Distance | 10 |
| Adjust Velocity Frequency | 50 |  | **Electrostatics** | Spherical Cutoff |
| save Results interval (ps) | 2 |  | **Run On Grid** | True |
| Constraints | none |  |  |  |
| **Equilibration** |  |  |  |  |
| Simulation Time (ps) | 10 |  |  |  |
| Time Step (fs) | 2 |  |  |  |
| Target Temperature | 300 |  |  |  |
| Adjust Velocity Frequency | 50 |  |  |  |
| Save Results Interval (ps) | 2 |  |  |  |
| Constraints |  |  |  |  |

**Table S3. The predicted binding stabilities of SARS-CoV-2 RBD targeting CV07-270 by DS.**

|  | **A** | **R** | **N** | **D** | **C** | **Q** | **E** | **G** | **H** | **I** | **L** | **K** | **M** | **F** | **P** | **S** | **T** | **W** | **Y** | **V** |
| --- | --- | --- | --- | --- | --- | --- | --- | --- | --- | --- | --- | --- | --- | --- | --- | --- | --- | --- | --- | --- |
| **R346** | 0.36 | -0.18 | 0.52 | 0.20 | 0.35 | 0.48 | 0.97 | 0.64 | 0.73 | 1.40 | 0.58 | -0.09 | 0.35 | 0.51 | 0.38 | 0.91 | 1.67 | 0.72 | 0.36 | 1.56 |
| **S349** | 0.01 | -0.17 | 0.24 | 0.57 | 0.31 | -0.09 | 0.77 | 0.29 | 0.55 | -0.13 | -0.10 | 0.28 | 0.48 | 3.14 | 0.52 | -0.07 | -0.24 | 2.48 | 1.94 | 0.48 |
| **Y351** | 0.50 | 0.36 | 0.58 | 0.43 | 0.50 | 0.77 | 0.52 | 0.52 | 0.36 | 0.51 | 0.48 | 1.12 | 0.23 | 0.36 | 0.42 | 0.52 | 0.51 | 0.10 | 0.00 | 0.52 |
| **K444** | 1.40 | 1.29 | 0.79 | 1.96 | 1.46 | 1.85 | 2.56 | 1.62 | 1.37 | 0.80 | 0.77 | -0.16 | 0.76 | 1.09 | 0.85 | 1.73 | 1.00 | 1.15 | 1.23 | 0.94 |
| **G446** | 1.83 | 5.33 | 1.76 | 1.97 | 1.10 | 1.90 | 1.86 | 0.12 | 3.11 | 3.61 | 2.34 | 3.16 | 2.28 | 1.53 | 3.37 | 3.44 | 2.70 | 1.28 | 1.81 | 4.20 |
| **G447** | 0.59 | 0.79 | 0.26 | 1.39 | -0.05 | 2.58 | 1.32 | -0.01 | 0.68 | 2.33 | 0.15 | 0.82 | 0.32 | 1.01 | 0.39 | 0.80 | 2.97 | 0.27 | 0.76 | 1.64 |
| **N448** | 0.13 | -0.55 | -0.15 | 0.69 | 0.02 | -0.14 | 0.47 | 0.16 | -0.66 | -0.20 | -0.16 | -0.47 | -0.58 | -0.98 | 0.03 | 0.18 | 0.04 | 3.29 | -0.50 | -0.20 |
| **Y449** | 1.98 | 2.71 | 1.20 | 2.00 | 1.60 | 2.13 | 4.48 | 2.72 | 0.27 | 2.18 | 0.45 | 1.71 | 1.50 | 0.19 | 1.32 | 2.21 | 3.29 | 2.39 | 0.00 | 2.82 |
| **N450** | 2.22 | 2.75 | -0.01 | 2.62 | 1.73 | 1.41 | 4.03 | 2.84 | 1.48 | 2.69 | 1.29 | 3.54 | 3.18 | 3.31 | 0.95 | 2.31 | 2.33 | 7.53 | 3.68 | 1.28 |
| **Y451** | 0.15 | 0.38 | 0.20 | 0.27 | 0.08 | 0.03 | 0.24 | 0.09 | -0.31 | 0.08 | -0.11 | 0.01 | -0.15 | 0.21 | -0.31 | 0.08 | 0.20 | 0.35 | -0.15 | 0.02 |
| **L452** | 1.41 | 2.63 | 1.13 | 1.79 | 1.32 | 0.49 | 1.01 | 1.52 | -0.05 | 0.19 | -0.01 | 1.33 | 0.15 | 0.87 | 0.88 | 1.46 | 1.12 | 2.15 | -0.11 | 0.96 |
| **T470** | 0.13 | 1.26 | 0.23 | -0.36 | 0.24 | -0.19 | -0.48 | 0.27 | 0.17 | -0.24 | -0.66 | 0.87 | 0.34 | 0.05 | 0.05 | 0.06 | -0.03 | 0.24 | 0.24 | -0.14 |
| **E484** | 1.11 | 1.32 | 0.93 | 0.63 | 0.98 | 0.83 | -0.04 | 1.48 | 0.27 | 0.40 | 0.38 | 2.00 | 1.23 | 0.16 | 0.37 | 1.49 | 1.41 | -0.15 | 0.46 | 0.73 |
| **F490** | 1.95 | 3.03 | 1.28 | 1.24 | 1.69 | 1.68 | 1.80 | 2.45 | 0.52 | 1.35 | 1.15 | 2.46 | 1.42 | -0.02 | 2.40 | 1.99 | 1.90 | 0.14 | -0.18 | 2.18 |
| **L492** | 0.22 | 0.47 | 0.13 | 0.00 | 0.17 | 0.10 | 0.30 | 0.53 | -0.08 | 0.07 | -0.03 | 0.14 | -0.12 | 0.18 | 0.23 | 0.17 | -0.10 | 1.02 | 0.13 | -0.08 |
| **S494** | -0.09 | 1.98 | -0.07 | 0.76 | -0.29 | 3.83 | 5.02 | 0.62 | 0.09 | 1.52 | 1.51 | 1.31 | 1.86 | 0.58 | 0.88 | 0.28 | 0.00 | 1.18 | 0.21 | 0.10 |

**Table S4. The predicted binding stabilities of SARS-CoV-2 RBD targeting B38 by DS.**

|  | **A** | **R** | **N** | **D** | **C** | **Q** | **E** | **G** | **H** | **I** | **L** | **K** | **M** | **F** | **P** | **S** | **T** | **W** | **Y** | **V** |
| --- | --- | --- | --- | --- | --- | --- | --- | --- | --- | --- | --- | --- | --- | --- | --- | --- | --- | --- | --- | --- |
| **R403** | 1.92 | -0.26 | 2.16 | 2.89 | 2.05 | 1.88 | 2.96 | 1.88 | 1.76 | 1.83 | 1.73 | 0.50 | 1.14 | 1.52 | 1.78 | 2.01 | 2.13 | 1.32 | 1.30 | 2.07 |
| **D405** | -0.18 | -1.64 | -0.58 | -0.02 | -0.24 | 0.17 | 1.07 | 0.41 | -1.01 | 1.03 | -0.82 | 0.42 | -0.71 | -1.46 | -0.37 | -0.24 | 0.34 | -1.82 | -1.76 | 0.05 |
| **T415** | 0.04 | -0.76 | -0.23 | 0.04 | 0.97 | -0.05 | 0.07 | 0.21 | 0.11 | -0.45 | -0.22 | -0.07 | -0.95 | 0.03 | -0.14 | 1.31 | -0.05 | -0.06 | 0.04 | 0.23 |
| **G416** | -0.47 | -1.06 | -1.52 | 0.05 | -0.75 | -0.70 | 0.36 | -0.14 | -1.30 | -1.19 | -1.04 | -0.61 | -0.63 | -2.12 | -0.71 | -0.81 | -1.00 | -2.18 | -2.72 | -1.17 |
| **K417** | 1.11 | -0.24 | 1.02 | 1.79 | 1.12 | 0.62 | 1.56 | 1.26 | 0.68 | -0.03 | 0.68 | 0.00 | 0.31 | 1.09 | -0.79 | 1.31 | 0.85 | 0.31 | 0.80 | 0.64 |
| **D420** | -0.88 | 0.21 | -1.09 | -0.38 | -1.05 | -1.43 | 0.40 | -0.62 | 0.70 | -1.72 | -2.12 | -0.53 | 0.09 | 2.02 | -1.28 | -1.70 | -1.37 | 2.87 | 0.51 | -1.41 |
| **Y421** | 1.69 | 1.00 | 1.74 | 2.19 | 1.45 | 1.47 | 2.44 | 1.34 | 1.00 | 1.31 | 1.20 | 0.32 | 0.61 | 0.59 | 1.47 | 1.68 | 1.44 | 5.48 | -0.02 | 1.33 |
| **L455** | -0.06 | 0.12 | -0.04 | -0.21 | -0.03 | -0.06 | -0.21 | -0.08 | -0.04 | -0.06 | -0.03 | 0.13 | -0.04 | -0.01 | -0.06 | -0.05 | -0.05 | -0.02 | 0.00 | -0.06 |
| **F456** | 1.66 | -0.04 | 1.32 | 1.89 | 0.88 | 0.64 | 0.97 | 1.76 | 0.63 | 0.32 | 0.79 | 0.05 | 0.13 | 0.00 | 0.88 | 1.52 | 0.76 | -0.93 | -0.77 | 0.90 |
| **R457** | -0.32 | 0.00 | -0.40 | -0.36 | -0.41 | -0.24 | -0.18 | -0.36 | -0.91 | 0.11 | -0.29 | -0.06 | -0.33 | -0.38 | -0.94 | -0.38 | -0.28 | -0.86 | -1.20 | -0.97 |
| **K458** | 0.79 | 0.57 | 0.63 | 0.48 | 0.77 | 0.52 | 0.27 | 0.86 | 0.69 | 0.14 | 0.66 | 0.01 | 0.90 | 0.29 | 0.37 | 0.77 | 0.28 | -0.58 | 0.37 | 0.79 |
| **N460** | 0.03 | -0.16 | -0.13 | -0.12 | 0.04 | 0.09 | 0.02 | 0.24 | 0.06 | -0.75 | -1.29 | 0.28 | 0.09 | -0.58 | -0.73 | 0.48 | -0.16 | -0.86 | 0.78 | -0.60 |
| **Y473** | 0.57 | -0.30 | 0.52 | 0.74 | 0.46 | 0.08 | 0.62 | 0.56 | 0.23 | 0.33 | 0.31 | -0.34 | 0.03 | 0.12 | -0.09 | 0.15 | 0.22 | 0.49 | 0.00 | 0.47 |
| **A475** | 0.00 | -0.83 | -0.75 | 0.83 | 0.19 | 0.50 | 0.99 | 0.55 | -0.29 | 0.09 | 0.51 | -1.28 | -0.18 | -0.78 | -0.54 | -0.50 | -1.11 | 1.80 | 0.00 | -0.05 |
| **G476** | -0.50 | 0.23 | -0.49 | -0.57 | -0.51 | -0.36 | -0.26 | 0.00 | -0.62 | -0.58 | -0.12 | 0.39 | -0.32 | -0.37 | -0.12 | -0.86 | -0.87 | -0.54 | -0.42 | 2.13 |
| **F486** | 2.01 | 2.04 | 1.86 | 1.95 | 1.95 | 1.92 | 1.97 | 2.09 | 1.06 | 1.60 | 1.22 | 2.42 | 1.68 | -0.02 | 1.91 | 2.07 | 1.83 | 0.36 | -0.23 | 1.79 |
| **N487** | 1.21 | 1.12 | 0.00 | 0.94 | 0.90 | 0.76 | 1.11 | 1.39 | 1.34 | 0.26 | 1.23 | 1.24 | 2.90 | 3.37 | 1.22 | 1.04 | 1.03 | 2.57 | 2.71 | 0.24 |
| **Y489** | 1.78 | -0.01 | 1.24 | 1.98 | 1.60 | 1.23 | 1.98 | 2.01 | 0.76 | 1.37 | 0.92 | 0.10 | 0.57 | 0.42 | 1.23 | 1.69 | 1.97 | 0.14 | 0.00 | 1.37 |
| **Q493** | -0.06 | -0.22 | -0.56 | 0.03 | -0.09 | 0.00 | 0.24 | -0.09 | -0.65 | -1.08 | -0.57 | -0.58 | -0.35 | -1.48 | -0.65 | -0.05 | -0.88 | -1.37 | -1.30 | -0.89 |
| **Y495** | -0.68 | -0.61 | -0.63 | -0.28 | -0.73 | -0.66 | -0.44 | -0.44 | -0.70 | -1.22 | -0.76 | -0.60 | -0.62 | -0.12 | -0.76 | -0.67 | -0.60 | 0.12 | -0.05 | -0.98 |
| **G496** | -0.31 | -2.85 | -0.89 | -0.10 | -0.53 | -0.20 | -0.08 | -0.10 | -0.91 | -1.59 | -2.41 | 0.19 | -1.27 | -0.85 | -0.45 | -0.32 | -0.51 | -1.82 | -2.63 | -0.76 |
| **Q498** | 0.16 | 0.45 | 0.17 | 0.12 | 0.34 | -0.08 | 0.17 | 0.32 | -0.39 | -0.91 | -0.02 | 0.51 | -0.08 | -0.56 | -0.57 | 0.33 | 0.02 | -0.35 | -0.45 | -0.23 |
| **T500** | 0.10 | -1.44 | -0.46 | -0.52 | 0.12 | -0.63 | -0.74 | 0.25 | -0.78 | -0.89 | -0.99 | -0.95 | -1.16 | -1.37 | -0.26 | 0.15 | -0.10 | -0.23 | -1.53 | -0.57 |
| **N501** | 0.60 | -1.17 | -0.16 | 0.94 | 0.47 | 0.02 | 0.82 | 0.82 | 0.31 | 0.07 | -1.01 | -0.28 | -0.65 | 2.60 | -0.55 | 0.49 | 0.10 | 4.04 | 1.30 | -0.51 |
| **G502** | 0.08 | -1.39 | 0.69 | 1.31 | -0.28 | 0.35 | 1.48 | -0.11 | 0.49 | 0.03 | 1.94 | 0.24 | 0.39 | 0.23 | 20.69 | 0.24 | 0.50 | -0.04 | 0.12 | 0.54 |
| **Y505** | 3.77 | 1.60 | 3.02 | 3.98 | 3.38 | 3.02 | 3.82 | 4.15 | 1.68 | 1.75 | 1.67 | 1.43 | 2.62 | 0.33 | 2.85 | 4.02 | 3.46 | 0.56 | -0.06 | 2.81 |

**Table S5. The predicted binding stabilities of SARS-CoV-2 RBD targeting CT-P59 by DS.**

|  | **A** | **R** | **N** | **D** | **C** | **Q** | **E** | **G** | **H** | **I** | **L** | **K** | **M** | **F** | **P** | **S** | **T** | **W** | **Y** | **V** |
| --- | --- | --- | --- | --- | --- | --- | --- | --- | --- | --- | --- | --- | --- | --- | --- | --- | --- | --- | --- | --- |
| **R403** | -0.02 | -0.02 | 0.26 | 0.29 | 0.18 | 0.34 | 0.73 | 0.02 | 0.14 | 0.32 | 0.09 | -0.40 | 0.19 | 0.38 | -0.11 | 0.15 | 0.12 | 1.04 | 0.69 | -0.02 |
| **K417** | -0.81 | -1.21 | -0.72 | -0.90 | -0.80 | -0.44 | -0.41 | -0.88 | -0.71 | -1.04 | -0.78 | -1.55 | -0.70 | -0.47 | -1.22 | -1.00 | -1.04 | -0.77 | -0.54 | -0.73 |
| **Y449** | 1.43 | 2.46 | 0.79 | 0.75 | 1.27 | 3.52 | 3.98 | 2.20 | 0.51 | 0.42 | 0.97 | 2.68 | 2.44 | -0.10 | 0.61 | 1.82 | 1.36 | 0.38 | -0.06 | 0.52 |
| **N450** | 0.15 | 0.79 | -0.01 | -0.76 | 0.21 | 0.10 | -0.58 | 0.22 | -0.23 | 0.09 | -0.08 | 0.83 | 0.12 | -0.49 | 0.06 | 0.24 | 0.24 | -0.97 | -0.48 | -0.03 |
| **L452** | 1.95 | 1.79 | 0.83 | 0.84 | 1.41 | 1.19 | 1.15 | 1.97 | 1.61 | 0.51 | -0.01 | 2.88 | 1.89 | 0.90 | 1.10 | 1.71 | 0.88 | 3.61 | 1.26 | 1.00 |
| **Y453** | -0.45 | 0.25 | -0.28 | -0.30 | -0.48 | -0.24 | -0.21 | -0.53 | -0.37 | -0.33 | -0.29 | -0.84 | -0.62 | -0.25 | -0.53 | -0.44 | -0.39 | 0.05 | -0.10 | -0.45 |
| **L455** | 2.05 | 3.01 | 0.96 | 2.30 | 1.74 | 1.46 | 2.49 | 2.28 | 2.41 | 0.67 | -0.10 | 1.41 | 1.44 | 2.49 | 1.45 | 1.89 | 1.16 | 6.77 | 2.88 | 1.26 |
| **F456** | 0.94 | -0.20 | 0.90 | 0.68 | 0.88 | 0.64 | 0.52 | 0.96 | 0.26 | 0.58 | 0.68 | 1.23 | 0.17 | -0.13 | 0.48 | 0.69 | 0.68 | 0.57 | -0.66 | 0.41 |
| **E484** | 0.31 | 0.17 | 0.07 | -0.07 | 0.29 | 0.41 | -0.05 | 0.44 | 0.12 | -0.16 | 0.00 | 2.09 | 0.56 | 0.15 | -0.02 | 0.88 | 0.69 | -0.04 | -0.03 | 0.08 |
| **G485** | -0.43 | 0.27 | -0.57 | -0.88 | -0.32 | -0.47 | -0.75 | -0.04 | -0.61 | 0.35 | -0.29 | 0.98 | 0.29 | 0.15 | -0.59 | 0.08 | 1.10 | -2.42 | -1.78 | -0.46 |
| **F486** | 1.87 | 2.43 | 1.71 | 2.04 | 1.53 | 2.11 | 2.06 | 2.85 | 0.76 | 1.59 | 1.11 | 2.81 | 1.73 | -0.06 | 1.57 | 2.53 | 2.46 | 0.42 | 0.01 | 3.58 |
| **Y489** | 1.25 | 0.14 | 1.09 | 1.02 | 1.16 | 0.86 | 0.67 | 1.55 | 0.53 | 1.69 | 0.77 | 1.17 | 0.90 | 0.21 | 1.10 | 1.34 | 1.91 | 3.99 | -0.06 | 1.17 |
| **F490** | 1.19 | 2.55 | 0.65 | 0.19 | 1.06 | 2.31 | 0.88 | 1.48 | 0.26 | 0.24 | 0.59 | 1.05 | 0.36 | -0.09 | 0.22 | 1.20 | 0.81 | -0.37 | -0.15 | 0.87 |
| **L492** | -0.01 | 0.22 | 0.02 | -0.31 | -0.05 | 0.00 | -0.60 | 0.33 | 0.04 | -0.08 | -0.10 | 0.55 | -0.09 | -0.02 | 0.31 | -0.04 | 0.15 | -0.45 | 0.00 | -0.23 |
| **Q493** | 1.33 | -0.12 | 0.80 | 1.36 | 1.24 | -0.09 | 1.37 | 1.48 | 0.54 | 0.74 | 1.58 | 0.42 | 1.21 | -0.34 | 0.81 | 1.40 | 1.23 | 6.69 | -0.15 | 0.75 |
| **S494** | 0.15 | -0.98 | 1.78 | 4.44 | -0.08 | 0.52 | 2.81 | 0.97 | 1.55 | -1.35 | 0.92 | -0.56 | 2.72 | 5.27 | 20.57 | -0.10 | -0.33 | 13.08 | -0.67 | -0.96 |
| **Y505** | 0.66 | 0.45 | 0.43 | 0.71 | 0.65 | 0.70 | 1.54 | 0.70 | 0.59 | 0.21 | 0.29 | 1.42 | 0.06 | -0.02 | 0.39 | 0.90 | 0.79 | 0.46 | -0.04 | 0.55 |

**Table S6. The predicted binding stabilities of SARS-CoV-2 RBD targeting CA1-B12 by DS.**

|  | **A** | **R** | **N** | **D** | **C** | **Q** | **E** | **G** | **H** | **I** | **L** | **K** | **M** | **F** | **P** | **S** | **T** | **W** | **Y** | **V** |
| --- | --- | --- | --- | --- | --- | --- | --- | --- | --- | --- | --- | --- | --- | --- | --- | --- | --- | --- | --- | --- |
| **R403** | 1.11 | -0.01 | 1.35 | 1.60 | 1.12 | 1.28 | 1.67 | 0.97 | 1.12 | 1.22 | 1.25 | 0.74 | 0.62 | 1.22 | 0.97 | 1.16 | 1.16 | 1.66 | 1.14 | 1.26 |
| **T415** | **0.39** | **0.34** | **0.42** | **0.16** | **0.27** | **0.30** | **0.42** | **0.43** | **0.28** | **0.17** | **0.32** | **0.33** | **0.20** | **0.21** | **0.43** | **0.22** | **0.15** | **0.56** | **0.19** | **0.31** |
| **G416** | -0.19 | -0.77 | -0.55 | 0.01 | -0.50 | 0.27 | -0.05 | 0.00 | -1.33 | -0.23 | -1.14 | -0.67 | -0.74 | -1.63 | -0.13 | -0.15 | -0.71 | -1.03 | -1.67 | -0.42 |
| **K417** | 2.31 | 2.10 | 2.59 | 3.34 | 2.33 | 2.18 | 3.64 | 2.44 | 1.72 | 1.55 | 1.33 | 0.00 | 1.22 | 1.97 | 0.83 | 2.47 | 2.25 | 3.16 | 1.89 | 1.53 |
| **D420** | 0.10 | -0.55 | -0.09 | 0.00 | -0.04 | 0.16 | 0.34 | 0.16 | -0.21 | -0.02 | 0.00 | 1.11 | -0.43 | 0.23 | -0.22 | -0.04 | -0.36 | 0.07 | 0.59 | -0.11 |
| **Y421** | 1.60 | 0.94 | 1.60 | 1.92 | 1.59 | 1.63 | 2.56 | 1.52 | 1.08 | 1.27 | 1.16 | 1.21 | 0.78 | 0.70 | 1.28 | 1.65 | 1.59 | 4.67 | -0.01 | 1.51 |
| **Y453** | 0.45 | -0.25 | 0.47 | 0.52 | 0.36 | 0.34 | 0.67 | 0.31 | 0.17 | 0.67 | 0.46 | 0.79 | -0.45 | 0.05 | 0.47 | 0.48 | 0.64 | 0.35 | -0.07 | 0.69 |
| **L455** | 1.65 | 0.44 | 0.35 | 2.05 | 0.74 | 0.21 | 2.42 | 1.78 | 0.55 | -0.21 | 0.00 | 1.40 | 1.88 | 1.27 | 0.36 | 0.93 | 0.62 | 5.23 | -0.03 | 0.57 |
| **F456** | 1.28 | 0.45 | 0.91 | 1.38 | 1.19 | 0.77 | 1.01 | 1.33 | 0.17 | 0.02 | 0.86 | 0.23 | 0.12 | 0.00 | 0.91 | 1.27 | 1.04 | 1.20 | -0.34 | 0.11 |
| **R457** | -0.51 | 0.00 | -0.54 | -0.68 | -0.60 | -0.41 | -0.49 | -0.55 | -0.38 | 0.36 | -0.33 | -0.01 | -0.45 | -0.37 | -0.35 | -0.58 | 0.57 | -0.24 | -0.94 | 0.11 |
| **K458** | 0.00 | 0.37 | -0.31 | -0.44 | -0.04 | -0.43 | -0.70 | 0.11 | -0.22 | -0.51 | -0.71 | 0.00 | -0.26 | -0.42 | -0.64 | -0.04 | -0.45 | -0.29 | -0.48 | -0.45 |
| **N460** | 0.15 | 0.18 | 0.00 | -0.16 | 0.06 | 0.25 | -0.06 | 0.24 | 0.21 | -0.44 | 0.18 | 0.62 | 0.30 | -0.43 | -0.73 | 0.51 | 0.23 | -0.64 | -0.08 | -0.04 |
| **Y473** | 0.32 | 0.01 | 0.42 | 0.28 | 0.39 | 0.50 | 0.72 | 0.26 | 0.13 | 0.56 | 0.60 | -0.17 | 0.02 | -0.21 | 0.38 | 0.39 | 0.51 | 0.76 | -0.61 | 0.52 |
| **A475** | 0.04 | -1.27 | 1.07 | 2.06 | -0.26 | -0.34 | 1.55 | 0.53 | -1.02 | -1.63 | -0.96 | -0.48 | 0.27 | 0.55 | -0.68 | -0.06 | -0.81 | -0.80 | 1.29 | 0.01 |
| **G476** | -0.65 | 0.02 | -0.60 | -1.45 | -0.70 | -0.81 | -1.12 | 0.05 | -1.10 | -1.12 | -0.66 | -0.33 | -0.57 | -0.74 | -0.66 | -0.95 | -1.09 | -0.49 | -0.69 | 0.08 |
| **F486** | 1.54 | 1.01 | 1.48 | 1.05 | 1.55 | 1.43 | 0.92 | 1.61 | 0.63 | 1.01 | 0.87 | 1.94 | 0.90 | -0.01 | 1.52 | 1.58 | 1.54 | 0.27 | 0.64 | 1.35 |
| **N487** | 1.24 | 1.50 | -0.01 | 0.30 | 0.95 | 0.89 | 0.88 | 1.39 | 1.40 | 0.14 | 0.86 | 2.39 | 1.39 | 2.38 | 0.64 | 1.00 | 0.90 | 1.68 | 2.13 | 0.36 |
| **Y489** | 1.08 | 0.78 | 0.96 | 1.24 | 1.05 | 1.40 | 1.82 | 1.08 | 0.67 | 1.14 | 0.52 | 0.92 | 0.65 | 0.20 | 1.03 | 1.16 | 1.13 | 0.87 | 0.06 | 0.60 |
| **Q493** | 0.25 | 0.95 | -0.27 | 0.04 | 0.30 | 0.00 | 0.61 | 0.31 | -0.11 | -0.52 | -0.54 | 1.15 | -1.11 | 1.66 | 0.19 | 0.31 | 0.23 | 1.04 | 1.78 | -0.06 |
| **S494** | 0.01 | -0.09 | -0.05 | -0.57 | -0.09 | 0.01 | -0.25 | 0.03 | -0.51 | -0.01 | -0.52 | 0.40 | -0.24 | -0.63 | -0.32 | 0.00 | -0.12 | -1.06 | -0.43 | -0.01 |
| **Y495** | -0.64 | 0.02 | -0.55 | -0.61 | -0.69 | -0.54 | -0.56 | -0.65 | -0.46 | -0.61 | -0.61 | -0.22 | -0.52 | 0.03 | -0.57 | -0.65 | -0.79 | 0.31 | -0.01 | -0.69 |
| **Y496** | 0.98 | -0.84 | -0.19 | -0.12 | -0.36 | -0.41 | -0.25 | 0.00 | -0.39 | -0.92 | -0.39 | 0.53 | -0.38 | -0.97 | 0.50 | -0.40 | -0.68 | -1.22 | -1.09 | 0.72 |
| **Q498** | 1.12 | 2.13 | 1.07 | 0.84 | 1.23 | 0.00 | 0.62 | 1.24 | 0.30 | -0.23 | 0.62 | 0.53 | 0.74 | 0.51 | 0.14 | 1.23 | 0.99 | 0.34 | 0.34 | 0.77 |
| **T500** | 0.18 | -1.05 | -0.60 | -0.76 | 0.19 | -0.40 | -0.83 | 0.49 | -0.62 | -0.88 | -1.01 | -0.50 | -0.73 | -0.04 | -0.19 | 0.38 | -0.22 | -0.09 | -0.01 | -0.48 |
| **N501** | 0.85 | 0.22 | 0.03 | 0.58 | 0.70 | 0.36 | 0.94 | 1.05 | -0.64 | 0.31 | -0.91 | 0.07 | 0.75 | 2.48 | -0.29 | 0.74 | 0.82 | 1.67 | 3.34 | -0.18 |
| **G502** | 0.76 | 0.03 | 1.32 | 0.32 | 1.12 | 0.42 | 0.96 | 0.01 | -0.12 | 2.58 | 0.56 | 1.43 | 0.63 | -0.05 | 11.50 | 1.00 | 1.44 | -0.53 | 0.01 | 1.78 |
| **Y505** | 3.03 | 2.99 | 2.74 | 3.04 | 2.69 | 3.51 | 3.99 | 3.47 | 1.46 | 2.00 | 1.77 | 3.12 | 3.15 | 0.50 | 2.43 | 3.25 | 2.79 | 0.85 | 0.02 | 2.24 |

**Table S7. The predicted binding stabilities of SARS-CoV-2 RBD targeting CA1-B3 by DS.**

|  | **A** | **R** | **N** | **D** | **C** | **Q** | **E** | **G** | **H** | **I** | **L** | **K** | **M** | **F** | **P** | **S** | **T** | **W** | **Y** | **V** |
| --- | --- | --- | --- | --- | --- | --- | --- | --- | --- | --- | --- | --- | --- | --- | --- | --- | --- | --- | --- | --- |
| **R403** | 1.25 | -0.04 | 1.45 | 1.66 | 1.31 | 1.29 | 1.77 | 1.14 | 1.51 | 1.29 | 1.42 | 1.23 | 1.16 | 1.56 | 1.2 | 1.35 | 1.38 | 1.22 | 1.01 | 1.3 |
| **R408** | -0.47 | -0.01 | -0.44 | -0.72 | -0.47 | -0.45 | -0.72 | -0.48 | -0.47 | -0.51 | -0.47 | -0.08 | -0.5 | -0.42 | -0.56 | -0.46 | -0.46 | -0.2 | -0.38 | -0.46 |
| **T415** | 0.01 | 0.1 | -0.27 | -0.19 | -0.1 | -0.5 | -0.44 | 0.3 | -0.75 | -0.75 | -0.67 | -0.38 | -0.49 | -0.82 | -0.45 | 0.73 | 0 | 0.1 | -0.73 | -0.59 |
| **G416** | 1 | -1.6 | 0.04 | 0.8 | 0.88 | 1.02 | 1.55 | -0.06 | -0.69 | -0.29 | -1.67 | -0.19 | -0.27 | -0.43 | -0.06 | -0.12 | -0.12 | -1.68 | -2.34 | -0.44 |
| **K417** | 3.17 | 3.57 | 3.78 | 5.41 | 3.31 | 2.98 | 4.28 | 3.33 | 3.32 | 2.52 | 2.94 | -0.07 | 2.06 | 2.78 | 2.35 | 3.58 | 3.37 | 6.18 | 2.17 | 2.91 |
| **D420** | -0.39 | 2.37 | -1.12 | -0.01 | -0.73 | -0.81 | 0.53 | -0.34 | -0.46 | -0.47 | -1.42 | 0.36 | -0.25 | 0.14 | -0.98 | -0.79 | -1.01 | -1.25 | 0.68 | -1.09 |
| **Y421** | 1.46 | -0.38 | 1.16 | 1.91 | 1.42 | 1.52 | 2.17 | 1.38 | 0.8 | 0.56 | 0.97 | 0.61 | 0.33 | 0.43 | 1.16 | 1.38 | 1.44 | 3.79 | -0.04 | 1.36 |
| **Y453** | 0.35 | -0.7 | 0.34 | 0.56 | 0.3 | 0.37 | 0.72 | 0.24 | 0.03 | 0.59 | 0.25 | -0.31 | -0.34 | 0.06 | 0.33 | 0.38 | 0.45 | 0.86 | -0.11 | 0.61 |
| **L455** | 0.29 | -0.24 | 0.28 | 0.72 | 0.28 | -0.03 | 0.72 | 0.26 | 0.33 | 0.12 | 0.21 | -0.34 | 0.04 | 0.06 | 0.32 | 0.34 | 0.56 | -0.26 | 0 | 0.16 |
| **F456** | 1.93 | -0.49 | 1.09 | 2.42 | 1.6 | 1.21 | 2.29 | 1.83 | 0.7 | 0.61 | -0.07 | 2.59 | 1.86 | 0.61 | 1.95 | 1.77 | 1.69 | 4.27 | 1.63 | 1.31 |
| **R457** | 1.19 | -0.09 | 0.87 | 1.37 | 1.12 | 0.5 | 1 | 1.16 | -0.2 | -0.02 | 0.58 | 0.11 | 0.08 | -0.13 | 0.63 | 0.98 | 1.12 | -0.47 | -0.6 | 0.81 |
| **K458** | -0.41 | 0 | -0.32 | -0.54 | -0.49 | -0.17 | -0.32 | -0.45 | -0.53 | 0.45 | -0.4 | 0.1 | -0.25 | -0.62 | -0.14 | -0.48 | -0.66 | -0.11 | -0.26 | 0.02 |
| **N460** | 0.07 | 0.3 | 0.01 | -0.3 | 0.08 | -0.18 | -0.42 | 0.15 | -0.46 | -0.04 | -0.25 | 0.07 | -0.13 | -0.24 | 0.16 | 0.14 | 0.12 | 0.04 | -0.16 | -0.14 |
| **Y473** | 0.24 | -0.47 | 0 | -0.13 | 0.17 | 0.52 | 0.45 | 0.24 | 0.18 | -0.52 | -0.77 | 0.15 | -0.24 | 0.42 | -0.42 | 0.57 | 0.21 | -1.17 | 0.16 | -0.29 |
| **Q474** | 0.48 | -0.42 | 0.62 | 0.62 | 0.55 | 0.74 | 1.09 | 0.48 | 0.26 | 0.62 | 0.48 | -0.21 | 0.71 | 0.12 | 0.51 | 0.55 | 0.69 | 0.9 | -0.32 | 0.59 |
| **A475** | -0.12 | 0.49 | 0.07 | -0.41 | 0.13 | 0.05 | -0.08 | 0.06 | 0.05 | -0.01 | -0.02 | 0.45 | 0.06 | -0.16 | -0.14 | 0.11 | -0.08 | -0.51 | -0.32 | -0.11 |
| **G476** | -0.49 | -0.1 | -0.45 | -0.9 | -0.54 | -0.08 | -0.82 | -0.06 | -0.52 | -1.14 | -0.64 | -0.16 | -0.93 | -1.01 | -0.78 | -0.71 | -0.93 | -0.95 | -0.44 | -0.82 |
| **F486** | 1.22 | 0.95 | 1.12 | 0.82 | 1.24 | 1.07 | 0.71 | 1.27 | 0.31 | 0.61 | 0.69 | 1.17 | 0.46 | -0.05 | 1.19 | 1.25 | 1.15 | -0.44 | 0.14 | 1.05 |
| **N487** | 1.42 | 1.22 | -0.05 | 0.99 | 0.87 | 0.89 | 1.04 | 1.58 | 0.54 | 0.31 | 1.03 | 0.65 | 1.16 | 3.28 | 0.94 | 1.11 | 0.92 | 1.69 | 1.4 | 0.72 |
| **Y489** | 0.99 | 0.63 | 0.92 | 1.15 | 0.79 | 1.42 | 1.72 | 1 | 0.52 | 0.22 | 0.49 | 0.85 | 0.51 | 0.14 | 0.88 | 0.99 | 0.66 | 0.23 | 0 | 0.55 |
| **Q493** | 0.66 | 0.49 | 0.18 | 0.75 | 0.44 | -0.16 | 0.49 | 0.52 | 0.73 | -0.65 | -0.5 | 0.92 | 1.04 | 1.01 | 0.68 | 0.59 | 0.48 | 2.3 | 0.65 | 0.26 |
| **G496** | -0.4 | -0.78 | 0.26 | -0.02 | -0.36 | -1.22 | -0.09 | -0.02 | -0.47 | -1.5 | -0.88 | -0.27 | -1.35 | -0.78 | -0.61 | -0.36 | -0.06 | -0.6 | -0.73 | -0.43 |
| **Q498** | 0.68 | 1.14 | 0.6 | 0.47 | 0.79 | 0 | 0.16 | 0.77 | 0.21 | -0.51 | 0.23 | 1.21 | 0.43 | 0.38 | 0 | 0.81 | 0.07 | -0.06 | 0.27 | 0.41 |
| **T500** | 0.11 | -1.41 | -0.52 | -0.68 | 0.08 | -0.48 | -0.75 | 0.49 | -0.33 | -0.9 | -0.91 | -0.96 | -1.12 | -0.03 | -0.13 | 0.24 | -0.21 | -0.13 | -0.93 | -0.43 |
| **N501** | 0.84 | -0.39 | -0.01 | 0.21 | 0.64 | 0.26 | 0.47 | 0.95 | 0.88 | 0.21 | -0.63 | 0.48 | -0.67 | 2.36 | 0.23 | 0.59 | 0.29 | 2.53 | -0.5 | 0.03 |
| **G502** | 1.15 | 0.41 | 0.82 | 1.23 | 0.92 | 1.87 | 2.34 | -0.08 | 0.01 | 2.63 | 1.3 | 2.37 | 2.5 | 0.07 | 24.71 | 1.49 | 1.28 | 0.37 | 0.77 | 2.51 |
| **Y505** | 2.2 | 5.34 | 1.9 | 2.28 | 1.98 | 3.19 | 3.79 | 2.61 | 1.44 | 1.58 | 1.39 | 1.5 | 2.47 | 0.24 | 1.59 | 2.27 | 1.73 | 1.02 | -0.03 | 1.33 |

**Table S8. The predicted binding stabilities of SARS-CoV-2 RBD targeting 47D1 by DS.**

|  | **A** | **R** | **N** | **D** | **C** | **Q** | **E** | **G** | **H** | **I** | **L** | **K** | **M** | **F** | **P** | **S** | **T** | **W** | **Y** | **V** |
| --- | --- | --- | --- | --- | --- | --- | --- | --- | --- | --- | --- | --- | --- | --- | --- | --- | --- | --- | --- | --- |
| **R346** | -0.38 | 0.00 | -0.45 | -0.73 | -0.38 | -0.27 | -0.45 | -0.29 | -0.59 | -0.45 | -0.55 | 0.57 | -0.58 | -0.85 | -0.40 | -0.24 | -0.30 | -0.79 | -0.81 | -0.53 |
| **Y351** | 0.29 | 0.45 | 0.35 | -0.02 | 0.29 | 0.05 | -0.02 | 0.28 | 0.22 | 0.15 | 0.31 | 0.93 | 0.10 | 0.19 | 0.28 | 0.29 | 0.30 | -0.14 | 0.00 | 0.35 |
| **Y449** | 1.99 | 2.54 | 1.21 | 0.69 | 1.61 | 1.72 | 1.12 | 2.96 | 0.74 | 1.37 | 1.04 | 3.03 | 2.59 | 0.03 | 1.55 | 2.33 | 2.23 | 1.30 | 0.00 | 1.34 |
| **N450** | 0.76 | 0.59 | -0.01 | 0.26 | 0.56 | 0.18 | 0.06 | 0.95 | 0.14 | -0.26 | -0.21 | 0.67 | 0.31 | 0.03 | 0.27 | 1.14 | 0.66 | 0.43 | 0.02 | 0.05 |
| **L452** | 1.43 | 1.71 | 1.18 | 1.02 | 1.35 | 0.92 | 1.40 | 1.48 | 0.59 | 0.82 | -0.01 | 1.59 | 0.79 | 1.19 | 1.23 | 1.43 | 1.27 | 1.41 | 0.71 | 1.11 |
| **T470** | 0.00 | -0.55 | -0.31 | -0.34 | 0.03 | -0.34 | 0.80 | 0.69 | -0.53 | -0.90 | -0.55 | 0.43 | -0.21 | -0.71 | 0.45 | 0.40 | 0.00 | -0.78 | -0.64 | -0.40 |
| **I472** | 1.36 | 5.69 | 1.06 | 0.85 | 1.42 | 0.85 | -0.03 | 1.52 | 0.65 | 0.00 | -0.29 | 2.79 | 0.51 | 0.60 | 1.20 | 1.36 | 1.14 | 0.89 | 0.57 | 0.71 |
| **N481** | 0.02 | 1.37 | -0.10 | -0.65 | -0.03 | -0.20 | -0.69 | 0.25 | -0.07 | -0.37 | -0.06 | 0.69 | -0.09 | -0.15 | -0.16 | 0.13 | 0.05 | 0.05 | -0.24 | -0.05 |
| **G482** | 2.77 | 4.85 | 1.81 | 2.11 | 2.30 | 6.80 | 2.26 | 0.01 | 1.15 | 3.68 | 4.31 | 14.67 | 1.33 | 0.90 | 1.11 | 3.06 | 4.17 | 0.73 | 0.83 | 3.58 |
| **V483** | 1.60 | 2.08 | 1.05 | 1.17 | 1.43 | 1.91 | 1.48 | 2.19 | 1.74 | -0.17 | 0.53 | 2.61 | 2.04 | 1.55 | 1.04 | 2.11 | 1.19 | 1.35 | 1.07 | -0.01 |
| **E484** | 1.87 | 2.54 | 1.57 | 1.31 | 1.91 | 1.12 | 0.00 | 2.62 | 1.26 | 2.28 | 0.70 | 2.93 | 0.82 | 0.46 | 13.36 | 2.14 | 2.45 | 1.53 | 0.47 | 0.98 |
| **F490** | 3.38 | 1.92 | 2.34 | 2.26 | 3.13 | 3.57 | 3.09 | 4.20 | 0.95 | 2.76 | 2.20 | 3.61 | 2.71 | -0.02 | 4.38 | 3.60 | 3.43 | 0.38 | -0.47 | 3.18 |
| **S494** | 0.78 | 1.27 | 1.23 | 0.08 | 0.50 | 1.77 | 0.90 | 0.86 | 1.40 | 0.49 | 1.27 | 1.79 | 0.86 | 0.51 | 1.54 | -0.01 | 0.06 | 1.18 | 0.66 | 0.19 |

**Table S9. The predicted binding stabilities of SARS-CoV-2 RBD targeting CV07-270 by Mutabind2.**

|  | **A** | **R** | **N** | **D** | **C** | **Q** | **E** | **G** | **H** | **I** | **L** | **K** | **M** | **F** | **P** | **S** | **T** | **W** | **Y** | **V** |
| --- | --- | --- | --- | --- | --- | --- | --- | --- | --- | --- | --- | --- | --- | --- | --- | --- | --- | --- | --- | --- |
| **R346** | 0.53 | NA | 0.47 | 0.64 | 0.72 | 0.10 | 0.52 | 0.72 | 0.95 | 0.57 | 1.02 | 0.03 | 0.88 | 1.94 | 1.49 | 0.43 | 0.41 | 1.65 | 1.48 | 0.49 |
| **S349** | 0.73 | 2.10 | 1.57 | 2.31 | 1.29 | 2.31 | 2.30 | 1.00 | 2.18 | 1.51 | 2.08 | 2.76 | 1.58 | 1.68 | 0.77 | NA | 1.62 | 2.18 | 2.45 | 1.55 |
| **Y351** | 0.50 | 1.58 | 1.07 | 0.96 | 0.67 | 0.84 | 0.72 | 0.84 | 0.82 | 0.91 | 1.36 | 1.01 | -0.05 | -0.03 | 0.04 | 0.74 | 0.53 | -0.14 | NA | 0.51 |
| **K444** | 0.50 | 0.76 | 0.18 | 0.57 | 1.09 | 0.52 | 0.89 | 1.39 | 0.23 | 1.16 | 0.53 | NA | 0.23 | -0.43 | 1.75 | 0.27 | -0.20 | 0.19 | -0.90 | 0.62 |
| **G446** | -0.31 | 1.59 | -0.74 | 0.74 | -0.15 | 0.87 | 0.94 | NA | 1.51 | 1.45 | 0.90 | 1.34 | -0.54 | 0.85 | -0.55 | 0.90 | -0.17 | 2.08 | 1.13 | -0.16 |
| **G447** | 2.19 | 2.42 | 1.11 | 2.32 | 2.43 | 1.65 | 2.42 | NA | 2.12 | 2.20 | 2.38 | 2.76 | 1.95 | 2.20 | 2.12 | 2.06 | 1.92 | 2.05 | 2.40 | 2.34 |
| **N448** | 2.00 | 1.60 | NA | 1.52 | 1.71 | 1.44 | 1.36 | 1.83 | 0.56 | 1.80 | 1.63 | 1.30 | 1.30 | 1.42 | 1.08 | 1.87 | 1.79 | 1.86 | 1.83 | 1.48 |
| **Y449** | 1.90 | 2.83 | 1.58 | 2.65 | 2.04 | 1.87 | 3.00 | 2.11 | 1.06 | 2.46 | 1.53 | 1.88 | 1.54 | 0.50 | 2.19 | 1.83 | 2.39 | 0.20 | NA | 2.38 |
| **N450** | 2.32 | 3.22 | NA | 3.65 | 1.39 | 2.92 | 3.55 | 2.24 | 3.15 | 2.48 | 3.37 | 2.60 | 2.56 | 3.76 | 3.17 | 2.21 | 1.55 | 3.14 | 3.47 | 2.18 |
| **Y451** | 1.86 | 1.93 | 2.06 | 2.07 | 1.76 | 1.70 | 2.15 | 1.89 | 1.52 | 1.09 | 1.96 | 1.92 | 2.03 | 0.64 | 1.69 | 2.19 | 1.73 | 2.09 | NA | 1.84 |
| **L452** | 1.80 | -0.37 | 0.80 | 1.57 | 1.06 | -0.75 | 3.00 | 1.77 | 0.91 | 1.39 | NA | -0.11 | -0.56 | 0.23 | -0.10 | 1.63 | 1.33 | -1.22 | -0.40 | 0.70 |
| **T470** | 0.21 | 0.85 | 0.13 | 0.52 | 0.25 | 0.13 | 0.05 | 0.24 | 0.27 | 0.23 | 0.23 | 0.28 | 0.03 | 0.18 | 0.22 | 0.16 | NA | 0.21 | 0.00 | 0.27 |
| **E484** | 1.56 | 1.85 | 1.88 | 2.19 | 1.84 | 1.58 | NA | 1.76 | 1.92 | 1.91 | 2.00 | 2.44 | 0.93 | 2.38 | 0.94 | 0.99 | 1.78 | 2.97 | 2.35 | 2.23 |
| **F490** | 1.34 | 1.42 | 1.18 | 1.43 | 1.06 | 1.08 | 1.70 | 1.26 | 0.68 | 1.28 | 1.39 | 1.21 | 0.98 | NA | 1.43 | 1.46 | 1.61 | 0.29 | 0.48 | 1.72 |
| **L492** | 1.06 | 1.23 | 1.07 | 2.21 | 0.74 | 1.10 | 0.83 | 1.40 | 1.44 | -0.01 | NA | 2.57 | 0.45 | 1.02 | 1.01 | 0.84 | 0.92 | 1.01 | 1.02 | 0.05 |
| **S494** | -0.10 | 1.37 | 1.28 | 0.54 | -0.58 | 1.61 | 1.68 | 0.61 | 0.80 | 1.58 | 2.01 | 1.35 | 1.39 | 1.29 | 0.35 | NA | 0.51 | 1.08 | 1.23 | 1.03 |

**Table S10. The predicted binding stabilities of SARS-CoV-2 RBD targeting B38 by Mutabind2.**

|  | **A** | **R** | **N** | **D** | **C** | **Q** | **E** | **G** | **H** | **I** | **L** | **K** | **M** | **F** | **P** | **S** | **T** | **W** | **Y** | **V** |
| --- | --- | --- | --- | --- | --- | --- | --- | --- | --- | --- | --- | --- | --- | --- | --- | --- | --- | --- | --- | --- |
| **R403** | 2.42 | NA | 1.88 | 2.79 | 2.06 | 1.49 | 2.76 | 2.45 | 1.81 | 2.24 | 2.26 | -0.10 | 1.43 | 2.44 | 1.05 | 2.16 | 2.63 | 2.41 | 2.61 | 2.43 |
| **D405** | 0.48 | 0.58 | 0.23 | NA | 0.38 | 0.18 | 0.28 | 0.74 | 0.54 | 0.75 | 0.50 | 0.42 | 0.27 | 0.41 | 0.60 | 0.37 | 1.08 | 0.74 | 0.57 | 0.79 |
| **T415** | 0.14 | 0.16 | 0.51 | 0.24 | 0.28 | 0.26 | 0.32 | 0.33 | 0.20 | 0.09 | 0.21 | 0.19 | -0.05 | 0.28 | 0.23 | 0.15 | NA | 0.31 | 0.15 | 0.29 |
| **G416** | 0.90 | 1.13 | 0.82 | 1.81 | 0.94 | 1.39 | 1.51 | NA | 0.88 | 1.27 | 2.46 | 0.86 | 1.35 | 1.49 | 0.93 | 0.74 | 1.15 | 1.22 | 1.14 | 1.23 |
| **K417** | -0.07 | 0.64 | 1.25 | 1.51 | 0.99 | 0.52 | 0.20 | 1.37 | 0.39 | -1.42 | -0.96 | NA | -1.08 | -1.31 | 0.46 | 0.44 | -0.23 | 0.42 | -0.48 | -1.09 |
| **D420** | 0.68 | 1.33 | 0.28 | NA | 1.12 | 1.14 | 0.82 | 1.35 | 1.14 | 1.96 | 0.95 | 0.86 | 0.74 | 1.12 | 1.00 | -0.14 | 0.25 | 1.49 | 1.48 | 1.23 |
| **Y421** | 2.36 | 2.00 | 1.59 | 2.68 | 2.74 | 2.46 | 2.00 | 2.59 | 1.45 | 2.24 | 1.48 | 1.94 | 1.41 | 1.09 | 2.48 | 2.43 | 2.16 | 1.39 | NA | 2.63 |
| **L455** | 1.13 | 0.48 | 1.09 | 0.93 | 1.32 | 1.13 | 1.25 | 1.12 | 0.16 | 0.66 | NA | 0.62 | 0.78 | -0.70 | 0.70 | 1.81 | 1.51 | -0.91 | -0.80 | 0.82 |
| **F456** | 2.32 | 1.38 | 1.49 | 2.81 | 1.76 | 1.47 | 1.87 | 2.92 | 1.39 | 2.15 | 0.84 | 2.42 | 1.00 | NA | 2.39 | 2.34 | 2.04 | 0.90 | 1.03 | 2.08 |
| **R457** | 0.63 | 0.13 | NA | 0.60 | 0.74 | 0.25 | 0.46 | 0.25 | 0.31 | 0.57 | 0.62 | 0.26 | 0.56 | 0.57 | -0.38 | 0.48 | 0.62 | 0.64 | 0.39 | 0.45 |
| **K458** | 0.10 | 0.08 | 0.18 | -0.21 | 0.14 | -0.20 | -0.16 | 0.15 | -0.23 | 0.00 | 0.00 | NA | -0.10 | -0.37 | -0.74 | -0.08 | 0.18 | -0.18 | -0.32 | 0.14 |
| **N460** | -0.08 | 0.08 | NA | 0.47 | 0.09 | 0.17 | 0.14 | -0.71 | 0.23 | -0.20 | 0.13 | -0.13 | -0.08 | 0.25 | -0.14 | -0.04 | 0.14 | -0.22 | 0.21 | -0.10 |
| **Y473** | 0.99 | 1.28 | 0.89 | 1.35 | 1.03 | 1.11 | 1.32 | 1.53 | 1.15 | 0.99 | 0.71 | 1.68 | 0.62 | 0.22 | 1.08 | 1.03 | 1.30 | 0.61 | NA | 0.98 |
| **A475** | NA | 2.43 | 1.77 | 1.54 | 0.26 | 1.15 | 2.17 | 0.73 | 1.95 | 1.94 | 2.74 | 3.25 | 1.87 | 2.10 | 0.17 | 1.00 | 0.89 | 2.52 | 2.04 | 1.48 |
| **G476** | 0.00 | 1.24 | -0.15 | 0.67 | 0.02 | 0.68 | 0.05 | NA | 0.30 | 0.84 | 0.68 | 1.29 | 0.95 | 0.68 | 0.91 | -0.19 | 0.48 | 1.14 | 0.65 | 0.89 |
| **F486** | -0.04 | 0.41 | 0.00 | 0.55 | 0.17 | 0.08 | 0.10 | 0.53 | 0.61 | 0.58 | 0.07 | 0.03 | -0.02 | NA | 0.32 | -0.08 | 0.01 | -0.11 | 0.22 | 0.58 |
| **N487** | 1.45 | 1.58 | NA | 1.84 | 1.14 | 1.61 | 2.20 | 0.21 | 1.46 | 1.48 | 1.40 | 2.45 | 1.27 | 1.53 | 1.32 | 0.91 | 1.14 | 1.37 | 1.22 | 1.34 |
| **Y489** | 2.05 | 1.42 | 2.02 | 2.52 | 1.22 | 1.26 | 1.56 | 2.18 | 1.40 | 1.49 | 1.04 | 1.20 | 0.79 | 0.26 | 2.17 | 1.93 | 1.25 | 0.65 | NA | 1.16 |
| **Q493** | 0.27 | 0.67 | 0.06 | 0.23 | 0.17 | NA | 0.08 | 0.63 | 0.14 | 0.18 | 0.00 | 0.77 | -0.13 | -0.05 | 1.15 | 0.30 | 0.14 | 0.26 | -0.04 | 0.14 |
| **Y495** | 0.92 | 1.02 | 1.07 | 1.13 | 0.96 | 0.91 | 1.47 | 1.25 | 1.21 | 1.17 | 1.32 | 2.04 | 0.92 | 0.21 | 0.80 | 0.84 | 0.94 | 0.97 | NA | 0.96 |
| **G496** | 0.70 | 0.87 | 0.66 | 0.56 | 0.57 | 0.89 | 0.77 | NA | 0.84 | 0.75 | 0.91 | 0.95 | 0.96 | 0.97 | 0.49 | 0.72 | 0.49 | 0.94 | 0.84 | 0.85 |
| **Q498** | 0.36 | 0.03 | -0.04 | 0.46 | -0.97 | NA | 0.12 | 0.42 | 0.40 | -0.38 | -0.25 | -0.44 | -0.38 | 0.88 | 1.01 | 0.38 | 0.19 | 1.09 | 0.16 | -0.29 |
| **T500** | -0.01 | -0.30 | -0.14 | -0.09 | -0.08 | -0.10 | -0.02 | -0.04 | 0.01 | -0.18 | -0.16 | -0.19 | -0.24 | -0.21 | -0.23 | -0.01 | NA | -0.25 | -0.20 | -0.25 |
| **N501** | -0.39 | 1.42 | NA | 0.85 | 0.25 | 0.87 | 1.16 | 0.90 | 1.65 | 1.71 | 0.28 | 0.59 | 0.39 | 1.91 | 1.22 | -0.52 | -0.46 | 1.51 | 1.98 | 1.14 |
| **G502** | 0.74 | 1.73 | 0.90 | 0.93 | -0.15 | 1.02 | 1.36 | NA | 1.17 | 1.03 | 1.14 | 0.97 | 0.51 | 0.81 | 2.18 | 0.69 | 0.83 | 1.13 | 1.05 | 0.93 |
| **Y505** | 2.89 | 3.36 | 3.06 | 3.56 | 3.05 | 3.21 | 3.60 | 2.82 | 3.30 | 2.71 | 1.96 | 2.52 | 1.24 | 0.83 | 3.19 | 3.15 | 2.45 | 2.02 | NA | 2.70 |

**Table S11. The predicted binding stabilities of SARS-CoV-2 RBD targeting CT-P59 by Mutabind2.**

|  | **A** | **R** | **N** | **D** | **C** | **Q** | **E** | **G** | **H** | **I** | **L** | **K** | **M** | **F** | **P** | **S** | **T** | **W** | **Y** | **V** |
| --- | --- | --- | --- | --- | --- | --- | --- | --- | --- | --- | --- | --- | --- | --- | --- | --- | --- | --- | --- | --- |
| **R403** | 0.64 | NA | 0.43 | 1.10 | 0.78 | 0.55 | 1.03 | 1.03 | 1.31 | 1.35 | 1.29 | 0.36 | 0.69 | 1.80 | -0.22 | 0.03 | 1.15 | 1.71 | 1.52 | 1.08 |
| **K417** | -0.09 | 0.23 | 0.23 | 0.55 | 0.30 | 0.25 | 0.23 | 0.38 | 0.10 | -0.37 | -0.15 | NA | -0.20 | -0.40 | 0.24 | 0.14 | -0.04 | 0.23 | -0.48 | -0.33 |
| **Y449** | 1.67 | 1.63 | 1.73 | 1.46 | 1.75 | 1.84 | 1.23 | 1.67 | 1.57 | 1.39 | 1.65 | 2.84 | 1.11 | 0.63 | 1.54 | 2.18 | 1.69 | -0.34 | NA | 1.59 |
| **N450** | 0.46 | 0.43 | NA | 0.61 | 0.60 | 0.38 | 0.31 | 0.35 | 0.31 | 0.22 | 0.50 | 0.59 | 0.35 | 0.29 | 0.43 | 0.18 | 0.26 | 0.60 | 0.18 | 0.32 |
| **L452** | 2.04 | 1.59 | 1.41 | 2.42 | 2.24 | 2.03 | 3.03 | 2.35 | 2.29 | 2.38 | NA | 1.81 | 1.57 | 2.61 | 0.81 | 1.74 | 1.80 | 0.67 | 2.70 | 2.21 |
| **Y453** | 1.81 | 1.89 | 1.33 | 2.43 | 1.73 | 1.70 | 3.07 | 1.69 | 0.48 | 1.56 | 1.28 | 1.12 | 1.06 | 0.27 | 1.68 | 1.64 | 1.50 | 0.63 | NA | 1.85 |
| **L455** | 1.38 | 1.54 | 1.34 | 1.56 | 0.97 | 0.77 | 2.73 | 1.45 | 2.26 | 1.45 | NA | 1.90 | 1.84 | 1.63 | 1.32 | 2.47 | 2.67 | 0.93 | 1.16 | 1.10 |
| **F456** | 1.02 | 0.99 | 0.94 | 1.35 | 1.49 | 1.20 | 1.06 | 1.40 | 0.95 | 0.70 | -0.04 | 2.05 | 0.31 | NA | 1.37 | 1.13 | 0.97 | -0.11 | 0.41 | 1.07 |
| **E484** | 1.11 | 1.04 | 0.73 | 0.34 | 0.49 | 0.94 | NA | 1.61 | 0.95 | 0.32 | 0.00 | 0.99 | -0.05 | 0.68 | -0.57 | 0.23 | 0.45 | 0.72 | 0.75 | 0.41 |
| **G485** | 1.44 | 0.78 | 1.15 | 2.47 | 1.07 | 1.82 | 1.94 | NA | 2.68 | 2.87 | 1.48 | 1.23 | 0.88 | 0.82 | 2.60 | 1.11 | 2.08 | 2.44 | 0.80 | 2.85 |
| **F486** | 1.24 | 1.41 | 1.23 | 1.78 | 1.18 | 1.66 | 1.55 | 1.86 | 1.21 | 1.62 | 1.20 | 1.39 | 0.37 | NA | 1.71 | 1.18 | 1.29 | 0.23 | 0.05 | 1.72 |
| **Y489** | 2.00 | 1.59 | 2.05 | 2.45 | 1.56 | 2.05 | 1.94 | 2.25 | 1.64 | 2.03 | 1.84 | 1.94 | 0.54 | 0.29 | 2.10 | 2.13 | 2.07 | 0.95 | NA | 1.74 |
| **F490** | 1.05 | 1.37 | 1.55 | 1.61 | 0.61 | 1.70 | 1.99 | -0.29 | 0.95 | 0.96 | 1.15 | 1.52 | 0.46 | 0.44 | 1.10 | 1.39 | 0.18 | 0.60 | 0.73 | 1.60 |
| **L492** | 1.04 | 1.51 | 0.81 | 0.82 | 0.72 | 0.68 | 0.85 | 0.84 | 0.64 | 0.12 | NA | 1.94 | 0.02 | 0.42 | 0.47 | 0.75 | 0.51 | 0.62 | 0.75 | 1.01 |
| **Q493** | 1.00 | 0.80 | 0.14 | 2.07 | 0.90 | NA | 2.13 | 2.20 | 1.34 | 0.88 | 1.02 | 1.32 | 0.55 | 0.79 | 2.31 | 0.68 | 1.04 | 3.28 | 0.31 | 1.00 |
| **S494** | -0.40 | 1.51 | 1.14 | 1.30 | -1.00 | 1.26 | 1.98 | 0.54 | 2.70 | 0.95 | 2.60 | 2.96 | 1.11 | 0.91 | NA | 0.12 | 1.12 | 2.98 | 2.58 | 0.80 |
| **Y505** | 0.22 | -0.62 | 0.25 | 0.72 | 0.36 | 0.35 | 0.48 | 0.09 | 0.31 | 0.26 | 0.17 | 0.22 | 0.32 | 0.18 | 0.30 | 0.25 | 0.04 | -0.07 | NA | 0.27 |

**Table S12. The predicted binding stabilities of SARS-CoV-2 RBD targeting CA1-B12 by Mutabind2.**

|  | **A** | **R** | **N** | **D** | **C** | **Q** | **E** | **G** | **H** | **I** | **L** | **K** | **M** | **F** | **P** | **S** | **T** | **W** | **Y** | **V** |
| --- | --- | --- | --- | --- | --- | --- | --- | --- | --- | --- | --- | --- | --- | --- | --- | --- | --- | --- | --- | --- |
| **R403** | 1.48 | NA | 1.02 | 1.77 | 1.58 | 1.12 | 1.82 | 1.88 | 1.49 | 1.80 | 1.24 | 0.38 | 0.86 | 1.17 | 0.39 | 0.79 | 1.69 | 0.80 | 1.22 | 1.60 |
| **T415** | 0.39 | 0.34 | 0.42 | 0.16 | 0.27 | 0.30 | 0.42 | 0.43 | 0.28 | 0.17 | 0.32 | 0.33 | 0.20 | 0.21 | 0.43 | 0.22 | NA | 0.56 | 0.19 | 0.31 |
| **G416** | 1.27 | 2.21 | 1.33 | 1.54 | 1.30 | 2.08 | 1.39 | NA | 1.89 | 2.03 | 2.23 | 2.04 | 1.56 | 2.00 | 2.11 | 1.18 | 1.82 | 2.07 | 1.93 | 2.02 |
| **K417** | 0.01 | 1.43 | 1.33 | 2.26 | 1.47 | 1.35 | 2.10 | 2.73 | 2.15 | -0.37 | -0.38 | NA | -0.36 | 0.78 | 1.91 | 1.20 | 0.29 | 2.17 | 0.04 | -0.19 |
| **D420** | 1.08 | 1.12 | 0.21 | NA | 0.94 | 0.61 | -0.06 | 1.29 | 1.01 | 0.91 | 0.78 | 0.75 | 0.38 | 0.67 | 0.71 | 1.03 | -0.35 | 0.89 | 0.75 | 0.72 |
| **Y421** | 1.91 | 2.42 | 2.11 | 2.47 | 1.94 | 2.25 | 2.45 | 2.37 | 2.11 | 1.88 | 1.91 | 2.52 | 1.78 | 0.96 | 2.23 | 1.89 | 2.06 | 1.62 | NA | 2.12 |
| **Y453** | 1.70 | 1.53 | 0.88 | 1.34 | 1.11 | 1.59 | 1.66 | 1.56 | 1.42 | 1.28 | 1.40 | 1.65 | 1.03 | 0.44 | 1.19 | 1.33 | 1.30 | 0.95 | 0.03 | 1.03 |
| **L455** | 2.88 | 2.02 | 2.16 | 2.60 | 1.84 | 2.52 | 2.43 | 2.97 | 2.27 | 0.60 | NA | 2.64 | 0.44 | 0.73 | 2.98 | 2.89 | 2.43 | 0.70 | 0.56 | 2.04 |
| **F456** | 2.52 | 1.83 | 1.93 | 3.04 | 2.29 | 2.03 | 1.99 | 2.62 | 2.46 | 1.97 | 0.12 | 2.97 | 1.16 | NA | 1.94 | 2.02 | 2.05 | -0.25 | 0.01 | 1.91 |
| **R457** | 0.56 | NA | 0.07 | 0.51 | 0.77 | 0.24 | 0.44 | -0.01 | 0.51 | 0.58 | 0.52 | 0.28 | 0.53 | 0.60 | 0.01 | 0.55 | 0.45 | 0.62 | 0.62 | 0.48 |
| **K458** | 0.08 | 0.15 | 0.09 | -0.28 | 0.15 | 0.10 | -0.15 | 0.31 | -0.20 | 0.16 | 0.04 | NA | -0.22 | -0.29 | -0.64 | 0.06 | 0.13 | -0.17 | -0.43 | 0.17 |
| **N460** | 0.18 | -0.16 | NA | 0.23 | 0.37 | 0.07 | 0.10 | -0.41 | 0.28 | 0.11 | -0.05 | -0.11 | -0.05 | 0.28 | -0.15 | 0.18 | 0.23 | 0.25 | 0.22 | 0.16 |
| **Y473** | 1.55 | 1.85 | 1.17 | 1.71 | 1.48 | 1.47 | 1.72 | 1.80 | 1.38 | 1.16 | 0.89 | 1.86 | 0.90 | 0.21 | 1.40 | 1.75 | 1.73 | 1.16 | NA | 1.17 |
| **A475** | NA | 2.78 | 1.82 | 1.70 | 0.02 | 1.63 | 1.26 | 1.51 | 1.54 | 2.03 | 1.08 | 3.37 | 0.64 | 2.19 | 0.37 | 0.92 | 1.82 | 2.31 | 2.13 | 1.75 |
| **G476** | 0.09 | 1.55 | -1.33 | 0.01 | 0.08 | 0.64 | 0.24 | NA | -0.79 | 0.80 | 0.73 | 1.40 | 0.78 | -0.10 | -0.20 | -0.03 | 0.86 | 0.55 | 0.53 | 0.45 |
| **F486** | 0.03 | 0.44 | 0.03 | 0.53 | 0.10 | 0.17 | 0.52 | 0.45 | 0.38 | 0.36 | 0.24 | 0.61 | -0.05 | NA | 0.33 | -0.13 | -0.06 | 0.41 | 0.18 | 0.51 |
| **N487** | 1.53 | 1.69 | NA | 2.33 | 1.38 | 1.17 | 1.67 | 1.52 | 1.82 | 1.56 | 1.03 | 1.70 | 1.15 | 1.60 | 1.50 | 1.31 | 1.34 | 1.77 | 1.20 | 1.43 |
| **Y489** | 1.44 | 1.20 | 1.55 | 1.71 | 0.71 | 1.25 | 1.66 | 1.83 | 1.39 | 1.16 | 1.03 | 1.27 | 0.83 | 0.69 | 1.73 | 1.51 | 1.38 | 0.36 | NA | 1.53 |
| **Q493** | 0.27 | 1.16 | 1.28 | 1.27 | NA | 0.36 | 0.39 | 1.35 | 0.71 | 0.20 | 0.08 | 0.30 | -0.25 | -0.40 | 0.92 | 0.92 | 0.20 | 1.27 | 2.12 | 0.13 |
| **S494** | -0.05 | 0.28 | 0.35 | -0.01 | -0.84 | 0.18 | -0.10 | 0.29 | 0.32 | 0.22 | -0.10 | 0.31 | 0.00 | 0.44 | -0.56 | NA | 0.13 | 0.20 | 0.54 | 0.34 |
| **Y495** | 0.90 | 1.51 | 1.34 | 0.55 | 0.85 | 0.63 | 1.34 | 0.87 | 0.94 | 1.04 | 0.95 | 1.47 | 0.84 | 0.00 | 1.17 | 0.70 | 1.12 | 0.63 | NA | 1.01 |
| **G496** | 1.84 | 2.06 | 1.67 | 1.58 | 2.04 | 1.73 | 1.90 | NA | 1.61 | 1.63 | 1.62 | 2.36 | 1.76 | 1.56 | 1.53 | 1.88 | 1.77 | 1.57 | 1.63 | 1.67 |
| **Q498** | 0.66 | 1.25 | 0.34 | 0.75 | -0.41 | NA | 0.24 | 1.05 | 0.75 | -0.07 | 0.10 | -0.31 | -0.47 | 0.91 | 0.82 | 0.84 | 0.85 | 0.68 | 0.29 | 0.30 |
| **T500** | 0.18 | -0.13 | -0.11 | 0.01 | -0.22 | -0.07 | 0.10 | -0.10 | -0.02 | -0.44 | -0.19 | -0.57 | -0.22 | -0.27 | -0.25 | 0.24 | NA | -0.20 | -0.16 | -0.16 |
| **N501** | -0.41 | 1.48 | NA | 0.20 | -0.10 | 0.89 | 0.71 | 1.48 | 1.57 | 0.39 | 0.32 | 1.02 | -0.38 | 1.82 | 0.63 | -0.72 | -0.94 | 0.44 | 2.06 | 0.55 |
| **G502** | 0.90 | 1.49 | 0.91 | 1.07 | 0.08 | 0.71 | 1.06 | NA | 1.04 | 1.72 | 1.11 | 0.86 | 0.80 | 0.79 | 1.79 | 0.81 | 1.63 | 0.78 | 1.01 | 1.43 |
| **Y505** | 1.92 | 1.65 | 1.61 | 2.17 | 1.91 | 1.97 | 2.33 | 1.67 | 1.77 | 1.90 | 1.51 | 1.98 | 0.91 | 0.57 | 1.95 | 2.07 | 1.42 | 1.65 | NA | 2.05 |

**Table S13. The predicted binding stabilities of SARS-CoV-2 RBD targeting CA1-B3 by Mutabind2.**

|  | **A** | **R** | **N** | **D** | **C** | **Q** | **E** | **G** | **H** | **I** | **L** | **K** | **M** | **F** | **P** | **S** | **T** | **W** | **Y** | **V** |
| --- | --- | --- | --- | --- | --- | --- | --- | --- | --- | --- | --- | --- | --- | --- | --- | --- | --- | --- | --- | --- |
| **R403** | 1.56 | NA | 0.62 | 1.06 | 1.39 | 0.96 | 1.24 | 1.78 | 1.34 | 1.44 | 1.14 | 0.88 | 0.78 | 1.36 | 0.23 | 1.16 | 1.48 | 1.30 | 1.00 | 1.67 |
| **R408** | 0.00 | NA | 0.01 | 0.21 | 0.01 | 0.00 | 0.05 | 0.03 | 0.03 | 0.19 | 0.04 | 0.00 | 0.02 | 0.01 | 0.07 | 0.00 | 0.00 | 0.25 | 0.14 | 0.08 |
| **T415** | 0.25 | 0.09 | 0.27 | 0.27 | 0.14 | 0.14 | 0.14 | 0.46 | 0.09 | 0.08 | 0.00 | 0.17 | 0.05 | 0.01 | 0.12 | 0.12 | NA | 0.44 | 0.30 | 0.11 |
| **G416** | 1.78 | 2.62 | 1.80 | 2.07 | 2.36 | 2.42 | 1.47 | NA | 2.56 | 2.70 | 2.45 | 2.58 | 2.42 | 2.86 | 2.25 | 1.84 | 2.41 | 2.58 | 2.77 | 2.47 |
| **K417** | 0.00 | 2.43 | 1.34 | 1.87 | 1.54 | 1.18 | 1.30 | 2.72 | 1.22 | -0.30 | -0.30 | NA | -0.30 | 0.00 | 1.95 | 1.08 | 0.92 | 2.33 | 0.59 | -0.20 |
| **D420** | 1.18 | 1.76 | 1.42 | NA | 1.41 | 0.63 | 0.24 | 1.88 | 1.88 | 1.62 | 1.19 | 1.02 | 1.06 | 2.41 | 2.00 | 0.73 | 0.20 | 2.14 | 2.17 | 1.77 |
| **Y421** | 2.52 | 2.39 | 2.24 | 2.37 | 2.44 | 1.39 | 2.21 | 2.66 | 1.53 | 2.41 | 1.59 | 2.14 | 1.34 | 0.91 | 2.55 | 2.46 | 2.43 | 1.87 | NA | 2.43 |
| **Y453** | 1.67 | 2.00 | 0.99 | 1.94 | 1.76 | 1.93 | 1.91 | 1.71 | 1.55 | 1.72 | 1.70 | 1.82 | 1.64 | 0.58 | 1.66 | 1.26 | 1.59 | 1.86 | NA | 1.81 |
| **L455** | 2.54 | 2.24 | 2.21 | 2.73 | 1.87 | 1.84 | 2.49 | 2.96 | 2.54 | 1.75 | NA | 2.66 | 0.69 | 0.94 | 2.79 | 2.60 | 2.06 | 0.37 | 0.75 | 1.93 |
| **F456** | 1.79 | 1.53 | 1.65 | 1.90 | 1.48 | 1.65 | 1.88 | 1.90 | 1.91 | 1.50 | 0.18 | 2.06 | 0.63 | NA | 1.80 | 1.58 | 1.22 | 0.18 | 0.08 | 1.18 |
| **R457** | 0.58 | NA | 0.04 | 0.50 | 0.72 | 0.50 | 0.30 | 0.03 | 0.41 | 0.69 | 0.59 | 0.69 | 0.58 | 0.58 | 0.23 | 0.46 | 0.37 | 0.51 | 0.63 | 0.32 |
| **K458** | -0.01 | 0.11 | 0.06 | -0.10 | 0.09 | -0.23 | -0.23 | 0.06 | -0.23 | 0.08 | 0.10 | NA | -0.06 | -0.26 | -0.80 | 0.05 | 0.08 | -0.05 | -0.35 | 0.13 |
| **N460** | -0.13 | 0.04 | NA | 0.26 | 0.25 | 0.03 | 0.19 | -0.69 | 0.19 | 0.07 | -0.08 | 0.88 | -0.11 | 0.23 | -0.07 | -0.03 | 0.18 | 0.29 | 0.17 | 0.35 |
| **Y473** | 1.19 | 1.67 | 1.35 | 1.72 | 1.47 | 1.56 | 1.12 | 1.39 | 1.33 | 0.98 | 1.02 | 1.75 | 0.99 | -0.12 | 1.84 | 1.35 | 1.65 | 1.05 | NA | 0.97 |
| **Q474** | -0.30 | 0.21 | -0.10 | 0.05 | 0.19 | NA | -0.07 | 0.04 | 0.23 | 0.00 | 0.04 | -0.03 | 0.14 | 0.23 | -0.55 | -0.27 | -0.04 | 0.35 | 0.32 | 0.00 |
| **A475** | NA | 2.56 | 2.12 | 1.86 | -0.15 | 1.62 | 2.62 | 1.27 | 2.11 | 2.52 | 0.88 | 2.85 | 1.83 | 2.55 | -0.11 | 0.16 | 1.48 | 2.42 | 2.18 | 1.97 |
| **G476** | 0.32 | 0.94 | -0.43 | 0.58 | 0.34 | 0.52 | 0.60 | NA | -0.63 | 0.65 | 0.52 | 1.13 | 0.66 | 0.15 | 0.08 | 0.26 | 0.73 | 0.07 | -0.06 | 0.47 |
| **F486** | 0.02 | 0.21 | 0.08 | 0.42 | 0.10 | 0.12 | 0.35 | 0.60 | 0.17 | 0.42 | 0.30 | 0.12 | -0.07 | NA | 0.39 | -0.16 | -0.09 | -0.25 | 0.41 | 0.49 |
| **N487** | 1.48 | 1.54 | NA | 1.90 | 1.15 | 1.05 | 1.35 | 1.25 | 1.82 | 1.44 | 1.20 | 1.38 | 1.16 | 1.52 | 1.47 | 1.03 | 1.14 | 1.97 | 1.89 | 1.47 |
| **Y489** | 1.42 | 1.74 | 1.09 | 1.59 | 0.77 | 0.90 | 1.26 | 1.65 | 0.89 | 0.99 | 0.95 | 0.90 | 0.59 | 0.31 | 1.47 | 1.51 | 1.61 | 0.77 | NA | 0.78 |
| **Q493** | 0.60 | 1.54 | 0.42 | 0.89 | 0.69 | NA | 0.39 | 1.45 | 0.73 | -0.10 | 0.19 | 1.37 | 0.00 | 1.12 | 1.05 | 0.71 | 0.17 | 0.53 | 1.26 | 0.13 |
| **G496** | 0.58 | 1.59 | 0.48 | 1.02 | 0.40 | 0.84 | 0.61 | NA | 1.09 | 1.33 | 1.39 | 2.22 | 0.91 | 1.27 | 1.02 | 0.48 | 1.31 | 0.74 | 0.81 | 0.88 |
| **Q498** | 0.70 | 0.24 | 0.70 | -0.50 | 0.63 | NA | 0.92 | 0.94 | 0.35 | 0.05 | -0.60 | -0.30 | 0.42 | 0.81 | 0.30 | 0.41 | 0.76 | -0.10 | 0.59 | 0.59 |
| **T500** | -0.20 | 0.03 | 0.00 | -0.20 | -0.10 | 0.03 | 0.00 | 0.00 | 0.01 | -0.10 | -0.10 | -0.10 | -0.10 | -0.20 | -0.60 | -0.10 | NA | -0.20 | -0.10 | -0.10 |
| **N501** | 0.02 | 1.24 | NA | 0.41 | 0.59 | 0.58 | 0.76 | 1.02 | 0.64 | 0.80 | 0.77 | 0.98 | 0.17 | 0.60 | 0.76 | -0.30 | -0.70 | 1.06 | 0.53 | 0.21 |
| **G502** | 0.94 | 1.26 | 0.73 | 0.53 | 1.52 | 1.34 | 0.00 | NA | 0.88 | 0.99 | 1.28 | 1.30 | 1.28 | 2.29 | 1.42 | 1.53 | 0.74 | 1.45 | 1.91 | 0.01 |
| **Y505** | 1.68 | 1.45 | 1.15 | 1.67 | 1.74 | 1.84 | 1.94 | 1.32 | 1.79 | 1.94 | 1.91 | 1.59 | 1.74 | 0.31 | 1.63 | 1.03 | 1.37 | 1.41 | NA | 1.91 |

**Table S14. The predicted binding stabilities of SARS-CoV-2 RBD targeting 47D1 by Mutabind2.**

|  | **A** | **R** | **N** | **D** | **C** | **Q** | **E** | **G** | **H** | **I** | **L** | **K** | **M** | **F** | **P** | **S** | **T** | **W** | **Y** | **V** |
| --- | --- | --- | --- | --- | --- | --- | --- | --- | --- | --- | --- | --- | --- | --- | --- | --- | --- | --- | --- | --- |
| **R346** | 0.04 | NA | 0.20 | 0.03 | 0.26 | -0.10 | 0.06 | 0.00 | 0.04 | 0.19 | 0.12 | 0.00 | 0.04 | 0.01 | 0.06 | -0.20 | 0.00 | 0.08 | 0.00 | 0.19 |
| **Y351** | 0.77 | 1.04 | 0.93 | 1.00 | 0.86 | 0.89 | 1.01 | 1.06 | 0.87 | 0.68 | 0.19 | 1.10 | 0.39 | 0.11 | 0.81 | 0.90 | 0.84 | 0.35 | NA | 0.42 |
| **Y449** | 1.35 | 1.62 | 0.73 | 1.36 | 1.14 | 1.21 | 1.24 | 1.22 | 0.89 | 1.44 | 0.76 | 1.29 | 0.78 | 0.35 | 0.95 | 1.12 | 1.05 | -0.20 | NA | 1.49 |
| **N450** | 0.78 | 0.66 | NA | 1.04 | 1.11 | 0.76 | 0.87 | 1.16 | 0.77 | 1.04 | 1.21 | 1.07 | 0.67 | 1.01 | 0.92 | 0.58 | 0.92 | 1.02 | 1.05 | 1.02 |
| **L452** | 1.31 | -0.10 | 0.78 | 1.62 | 1.49 | 0.94 | 0.86 | 1.53 | 0.83 | 1.09 | NA | 0.09 | 0.00 | 0.84 | 0.14 | 1.10 | 1.03 | -1.00 | 1.62 | 0.03 |
| **T470** | 0.62 | 0.84 | 0.00 | 0.33 | 0.24 | 0.21 | 0.61 | 0.84 | 0.54 | 0.67 | 0.12 | 0.59 | 0.11 | 0.22 | 0.96 | 0.36 | NA | 0.20 | -0.10 | 0.64 |
| **I472** | 1.09 | 1.32 | 0.90 | 2.14 | 0.53 | 1.23 | 1.25 | 1.12 | 1.43 | NA | 1.02 | 2.10 | 0.03 | 1.27 | 0.65 | 0.79 | 0.94 | 1.33 | 1.27 | 0.00 |
| **N481** | 0.00 | 0.05 | NA | 0.00 | 0.00 | 0.00 | 0.00 | 0.38 | 0.00 | 1.00 | 0.07 | 0.00 | 0.03 | -0.10 | 0.65 | 0.00 | 0.45 | 0.00 | -0.10 | 1.23 |
| **G482** | 1.65 | 1.05 | 0.91 | 1.29 | 0.51 | 1.14 | 0.91 | NA | 1.40 | 2.12 | 1.33 | 0.88 | 1.20 | 0.32 | 0.46 | 1.48 | 1.65 | 0.20 | 0.31 | 2.09 |
| **V483** | 1.15 | 2.50 | 1.32 | 1.48 | -0.51 | 0.52 | 0.96 | 1.29 | 1.29 | 0.55 | 0.00 | 1.23 | 0.30 | 1.54 | 1.36 | 0.96 | 0.86 | 1.66 | 1.44 | NA |
| **E484** | 1.37 | 2.64 | 2.09 | 2.24 | 2.08 | 2.24 | NA | 1.97 | 2.25 | 2.35 | 2.38 | 2.52 | 1.18 | 2.46 | 1.51 | 1.15 | 0.86 | 2.69 | 2.49 | 1.85 |
| **F490** | 1.94 | 1.98 | 1.85 | 1.36 | 0.85 | 1.55 | 1.55 | 1.44 | 1.59 | 1.75 | 1.63 | 2.47 | 1.00 | NA | 1.72 | 1.76 | 1.25 | 0.76 | -0.12 | 1.68 |
| **S494** | -0.27 | 1.31 | -0.08 | -0.64 | -0.93 | 0.26 | -0.79 | -0.08 | 0.76 | 0.34 | 0.46 | 1.19 | -0.23 | 0.77 | -0.26 | NA | 0.02 | 0.45 | 0.47 | 0.02 |

**Table S15. The predicted binding stabilities of SARS-CoV-2 RBD targeting CV07-270 by FoldX.**

|  | **A** | **R** | **N** | **D** | **C** | **Q** | **E** | **G** | **H** | **I** | **L** | **K** | **M** | **F** | **P** | **S** | **T** | **W** | **Y** | **V** |
| --- | --- | --- | --- | --- | --- | --- | --- | --- | --- | --- | --- | --- | --- | --- | --- | --- | --- | --- | --- | --- |
| **R346** | 1.34 | 0.00 | 1.30 | 1.36 | 0.74 | 0.33 | 0.73 | 1.45 | 0.62 | -0.36 | -0.53 | -0.33 | -0.37 | -1.22 | 2.61 | 1.13 | 0.44 | -0.17 | -0.35 | 0.00 |
| **S349** | 0.59 | 13.75 | 4.99 | 2.19 | 0.85 | 8.37 | 5.18 | 1.11 | 23.80 | 7.96 | 7.85 | 8.57 | 6.56 | 18.04 | -0.20 | 0.00 | 4.50 | 33.44 | 20.77 | 4.08 |
| **Y351** | 1.86 | 4.84 | 3.26 | 3.40 | 2.36 | 2.68 | 2.55 | 2.94 | 2.71 | 3.02 | 4.04 | 2.61 | 0.48 | -0.51 | 1.14 | 2.48 | 1.93 | -0.70 | 0.00 | 1.85 |
| **K444** | 0.69 | -0.17 | 0.65 | -0.09 | 0.73 | 0.24 | -0.15 | 1.19 | 1.25 | 0.37 | 0.21 | 0.00 | -0.01 | -1.04 | 4.69 | 0.92 | 1.31 | 0.94 | -1.46 | 0.61 |
| **G446** | 0.75 | 3.44 | -0.38 | 0.03 | 1.66 | 2.03 | 0.83 | 0.00 | 3.45 | 3.31 | 0.21 | 2.90 | 1.33 | 3.18 | -1.29 | 2.43 | 3.68 | -0.47 | 3.50 | 1.94 |
| **G447** | 3.28 | 3.83 | 4.22 | 6.01 | 3.75 | 4.25 | 2.78 | 0.00 | 4.82 | 12.03 | 5.40 | 3.41 | 3.61 | 4.96 | 6.22 | 3.56 | 7.24 | 7.50 | 5.16 | 7.49 |
| **N448** | 2.86 | 3.68 | 0.00 | -0.66 | 2.01 | 3.93 | 3.95 | 4.23 | 0.98 | 5.22 | 2.31 | 3.05 | 0.97 | 3.07 | 9.04 | 2.91 | 2.69 | 5.81 | 3.63 | 2.74 |
| **Y449** | 2.07 | 1.81 | 1.55 | 2.57 | 1.91 | 2.38 | 3.76 | 2.81 | 1.44 | 5.36 | 0.42 | 1.07 | 0.56 | -0.58 | 5.54 | 1.98 | 3.37 | 1.58 | 0.00 | 5.47 |
| **N450** | -0.45 | 2.86 | 0.00 | 0.17 | -0.68 | 4.04 | 1.26 | 0.87 | 40.12 | 0.88 | 0.80 | 0.12 | 2.54 | 23.23 | 3.85 | -0.31 | -1.06 | 14.86 | 31.52 | -0.59 |
| **Y451** | 4.62 | 4.54 | 4.43 | 6.05 | 3.82 | 3.57 | 5.98 | 5.89 | 2.82 | 1.62 | 2.29 | 4.07 | 2.45 | 1.21 | 4.93 | 5.43 | 4.24 | 3.96 | 0.00 | 2.58 |
| **L452** | 3.13 | 4.68 | 2.88 | 2.56 | 2.79 | 0.79 | 1.79 | 3.90 | 2.58 | 2.12 | 0.00 | 2.40 | -0.26 | 0.61 | 3.95 | 3.69 | 3.42 | 8.37 | 0.84 | 2.88 |
| **T470** | 0.20 | -0.20 | 0.90 | 0.50 | 0.00 | -0.16 | -0.29 | 0.65 | 0.50 | 0.12 | -0.08 | -0.46 | -1.23 | -0.51 | 0.81 | 0.81 | 0.00 | 0.11 | 0.09 | -0.23 |
| **E484** | 1.75 | 2.16 | 2.88 | 2.84 | 1.43 | 1.59 | 0.00 | 2.45 | 2.63 | 0.77 | -0.02 | 2.51 | 0.33 | 1.96 | 0.57 | 2.20 | 2.12 | 2.77 | 2.30 | 0.73 |
| **F490** | 3.05 | 3.66 | 3.15 | 1.80 | 2.98 | 3.74 | 2.09 | 3.88 | 1.90 | 1.35 | 1.03 | 3.39 | 0.85 | 0.00 | 0.79 | 3.90 | 3.78 | 0.61 | 0.53 | 2.46 |
| **L492** | 3.85 | 9.50 | 3.31 | 4.02 | 3.33 | 3.76 | 2.84 | 5.34 | 4.23 | 0.88 | 0.00 | 5.65 | 1.74 | 5.04 | 7.21 | 5.13 | 3.86 | 12.16 | 5.54 | 1.48 |
| **S494** | -0.87 | 2.98 | 3.32 | 4.10 | -0.25 | 5.17 | 3.71 | 0.87 | 30.82 | 4.04 | 4.25 | 4.94 | 3.99 | 7.35 | 0.97 | 0.00 | -0.83 | 12.75 | 7.59 | 2.47 |

**Table S16. The predicted binding stabilities of SARS-CoV-2 RBD targeting B38 by FoldX.**

|  | **A** | **R** | **N** | **D** | **C** | **Q** | **E** | **G** | **H** | **I** | **L** | **K** | **M** | **F** | **P** | **S** | **T** | **W** | **Y** | **V** |
| --- | --- | --- | --- | --- | --- | --- | --- | --- | --- | --- | --- | --- | --- | --- | --- | --- | --- | --- | --- | --- |
| **R403** | 1.82 | 0.00 | 3.69 | 9.11 | 2.69 | 2.75 | 4.59 | 2.81 | 28.86 | 2.75 | 3.64 | 0.35 | 0.04 | 14.13 | 5.56 | 2.99 | 3.84 | 17.10 | 14.93 | 3.26 |
| **D405** | -0.10 | 0.83 | 0.78 | 0.00 | 0.91 | 0.59 | 0.59 | 0.92 | -0.55 | 0.10 | 1.18 | 0.34 | 0.05 | 0.66 | 5.56 | 0.91 | 1.73 | 1.20 | 1.11 | 0.80 |
| **T415** | 0.34 | -0.15 | 0.24 | 0.56 | -0.01 | 0.25 | 0.46 | 0.61 | -0.06 | -0.28 | -0.09 | -0.35 | -0.51 | 0.23 | 1.30 | 0.10 | 0.00 | 1.05 | -0.14 | 0.03 |
| **G416** | 5.52 | 6.24 | 6.05 | 4.61 | 6.10 | 6.34 | 4.76 | 0.00 | 6.89 | 9.35 | 5.99 | 6.13 | 5.13 | 5.48 | 15.03 | 6.27 | 9.07 | 6.95 | 7.29 | 11.06 |
| **K417** | 0.43 | -0.60 | -0.37 | 0.40 | 0.40 | -0.60 | -0.31 | 1.49 | 2.63 | -1.21 | -0.59 | 0.00 | -1.35 | 0.45 | -1.55 | 0.91 | 0.66 | 0.15 | 2.12 | -0.63 |
| **D420** | 1.94 | 1.50 | -0.49 | 0.00 | 0.56 | 1.90 | -0.06 | 2.11 | 6.71 | 3.54 | -0.32 | 1.85 | -0.05 | 1.18 | 8.31 | 0.52 | 1.88 | 5.73 | 1.79 | 3.63 |
| **Y421** | 2.40 | 3.28 | 2.45 | 4.05 | 3.33 | 2.64 | 4.09 | 2.96 | 2.24 | 3.14 | 0.23 | 1.72 | 0.47 | -0.53 | 3.11 | 3.02 | 3.55 | 4.79 | 0.00 | 4.56 |
| **L455** | 2.51 | 0.13 | 0.57 | 1.63 | 1.88 | 1.61 | 2.00 | 3.72 | 0.96 | 2.86 | 0.00 | 0.35 | 1.37 | -0.23 | 7.00 | 3.03 | 3.13 | 1.31 | 0.09 | 3.09 |
| **F456** | 4.60 | 3.65 | 4.62 | 4.80 | 4.08 | 4.21 | 4.08 | 5.89 | 3.12 | 6.74 | 1.77 | 3.92 | 1.33 | 0.00 | 5.86 | 5.48 | 4.20 | 1.04 | -0.05 | 3.37 |
| **R457** | 1.94 | 0.00 | 2.19 | 2.36 | 2.38 | 1.56 | 1.87 | 2.83 | 0.80 | 5.25 | 0.90 | 1.50 | 0.76 | 0.82 | 7.31 | 1.83 | 2.45 | 2.99 | 1.09 | 4.66 |
| **K458** | -0.05 | -0.09 | 0.32 | -0.86 | 0.44 | -0.50 | -0.51 | 0.11 | 0.42 | -0.12 | -0.19 | 0.00 | -0.30 | -0.57 | -1.27 | 0.39 | 0.26 | -0.72 | -0.82 | 0.21 |
| **N460** | 0.59 | 0.98 | 0.00 | 1.09 | 0.96 | 0.85 | 0.61 | 1.01 | 0.91 | 1.18 | 0.79 | 0.55 | 0.88 | 1.02 | -0.46 | 0.35 | 1.73 | 1.22 | 1.05 | 1.47 |
| **Y473** | 2.52 | 4.24 | 3.69 | 3.36 | 2.65 | 3.37 | 3.53 | 3.94 | 2.31 | 1.91 | 1.76 | 2.35 | 0.36 | 0.37 | 6.61 | 3.65 | 3.27 | 2.49 | 0.00 | 2.09 |
| **A475** | 0.00 | 9.69 | 3.97 | 1.74 | 0.59 | 6.13 | 3.91 | 0.85 | 51.44 | 7.02 | 3.69 | 8.22 | 1.19 | 12.37 | 3.87 | 1.47 | 3.98 | 13.95 | 16.01 | 5.38 |
| **G476** | 1.70 | 2.79 | 1.03 | 0.75 | -0.22 | 2.12 | 0.54 | 0.00 | 23.05 | 4.10 | 5.24 | 2.00 | 4.61 | 1.70 | 6.65 | 0.14 | 3.20 | 4.10 | 2.32 | 4.83 |
| **F486** | 2.36 | 2.31 | 2.56 | 2.34 | 2.35 | 2.28 | 2.34 | 2.58 | 2.16 | 2.60 | 2.00 | 2.28 | 1.90 | 0.00 | 1.56 | 2.48 | 2.56 | 2.32 | 2.07 | 2.60 |
| **N487** | 4.03 | 3.46 | 0.00 | 2.56 | 3.14 | 3.68 | 4.85 | 2.25 | 4.12 | 5.07 | 5.51 | 3.15 | 2.83 | 7.42 | 7.12 | 4.54 | 4.89 | 9.47 | 8.10 | 4.89 |
| **Y489** | 2.93 | 3.06 | 2.06 | 5.72 | 2.58 | 2.07 | 4.51 | 4.37 | 1.30 | 2.34 | 2.14 | 1.38 | -0.15 | -0.92 | 4.80 | 2.86 | 3.95 | -0.22 | 0.00 | 1.32 |
| **Q493** | 0.86 | -0.10 | 0.23 | -0.17 | 0.50 | 0.00 | -0.68 | 1.52 | 0.02 | 0.29 | -0.41 | 0.19 | -0.51 | -0.34 | 4.98 | 1.19 | 1.20 | 0.25 | -0.12 | 0.46 |
| **Y495** | 4.57 | 4.73 | 5.48 | 6.10 | 4.81 | 4.55 | 4.98 | 5.57 | 3.99 | 5.97 | 2.61 | 3.38 | 1.97 | 0.63 | 4.92 | 5.81 | 4.84 | 3.70 | 0.00 | 5.75 |
| **G496** | 2.38 | 2.81 | 2.52 | 3.17 | 3.51 | 2.52 | 2.73 | 0.00 | 2.89 | 4.75 | 2.66 | 2.40 | 3.57 | 2.25 | 7.76 | 3.43 | 4.50 | 2.56 | 2.29 | 4.58 |
| **Q498** | 0.51 | 0.95 | 0.99 | 1.60 | 0.51 | 0.00 | 1.05 | 1.47 | 2.18 | 0.55 | -0.67 | -0.18 | -0.10 | 4.22 | 2.29 | 1.13 | 1.09 | 5.77 | 6.58 | 0.51 |
| **T500** | -0.51 | -2.02 | -0.80 | -0.85 | -0.62 | -0.77 | -0.76 | -0.45 | -0.68 | -0.95 | -1.21 | -1.16 | -1.55 | -1.58 | -0.88 | -0.56 | 0.00 | -1.59 | -1.47 | -0.77 |
| **N501** | -0.35 | 4.51 | 0.00 | -0.25 | -0.28 | 2.12 | 1.61 | 1.00 | 3.95 | 2.00 | -0.85 | 2.22 | -0.49 | 2.70 | 4.20 | 0.20 | 0.36 | 10.05 | 8.96 | 0.76 |
| **G502** | -0.20 | 1.44 | 1.98 | 1.65 | 0.82 | 2.88 | 2.42 | 0.00 | 3.87 | 1.90 | -0.52 | 0.46 | -0.41 | -0.03 | 2.96 | 0.89 | 0.96 | 2.09 | 0.24 | 0.97 |
| **Y505** | 3.03 | 2.45 | 2.40 | 3.13 | 3.04 | 3.27 | 3.78 | 3.74 | 3.33 | 3.31 | 0.30 | 5.10 | 1.07 | 1.03 | 2.05 | 4.00 | 3.77 | 3.35 | -0.05 | 3.31 |

**Table S17. The predicted binding stabilities of SARS-CoV-2 RBD targeting CT-P59 by FoldX.**

|  | **A** | **R** | **N** | **D** | **C** | **Q** | **E** | **G** | **H** | **I** | **L** | **K** | **M** | **F** | **P** | **S** | **T** | **W** | **Y** | **V** |
| --- | --- | --- | --- | --- | --- | --- | --- | --- | --- | --- | --- | --- | --- | --- | --- | --- | --- | --- | --- | --- |
| **R403** | 1.47 | 0.00 | 5.41 | 5.64 | 1.84 | 2.03 | 4.87 | 2.31 | 27.16 | 4.62 | 1.13 | 0.92 | 0.22 | 11.67 | 7.04 | 1.88 | 4.10 | 8.00 | 10.33 | 3.25 |
| **K417** | 0.90 | -0.56 | 0.63 | 1.54 | 0.82 | 0.26 | 1.04 | 1.51 | 0.38 | -0.40 | -0.25 | 0.00 | 0.09 | 0.32 | -0.36 | 1.36 | 0.51 | 0.28 | 0.02 | 0.28 |
| **Y449** | 1.23 | 3.73 | 1.81 | 1.08 | 1.47 | 1.98 | 0.89 | 1.71 | 2.36 | 0.68 | 1.67 | 2.17 | 0.71 | 0.58 | 2.03 | 2.42 | 2.07 | -0.14 | 0.00 | 1.15 |
| **N450** | 0.22 | 0.05 | 0.00 | -0.18 | 0.30 | -0.33 | -0.70 | 0.60 | 0.51 | -0.11 | -0.11 | -0.28 | -0.62 | 0.37 | 1.70 | 0.05 | 0.23 | 0.47 | 0.49 | 0.39 |
| **L452** | 2.99 | 2.41 | 2.76 | 2.84 | 2.60 | 2.39 | 2.30 | 4.21 | 2.80 | 2.01 | 0.00 | 3.25 | 1.25 | 2.44 | 4.80 | 3.46 | 2.20 | 6.88 | 4.25 | 2.16 |
| **Y453** | 3.91 | 1.82 | 3.52 | 6.26 | 3.42 | 3.42 | 5.54 | 5.08 | 2.03 | 6.50 | 1.24 | 1.76 | 0.86 | -0.66 | 6.58 | 4.13 | 5.03 | 2.16 | 0.00 | 5.92 |
| **L455** | 3.12 | 6.05 | 0.68 | 1.41 | 2.40 | 3.09 | 4.92 | 4.74 | 38.47 | 1.73 | 0.00 | 5.12 | 3.42 | 7.76 | 7.28 | 3.49 | 4.38 | 19.19 | 14.45 | 2.45 |
| **F456** | 4.26 | 3.09 | 3.86 | 5.17 | 3.64 | 2.81 | 4.09 | 5.12 | 2.74 | 4.97 | 1.16 | 2.52 | 0.88 | 0.00 | 5.72 | 4.87 | 4.07 | 0.61 | 0.77 | 3.01 |
| **E484** | 0.88 | 0.74 | 1.16 | 1.41 | 0.65 | 0.31 | 0.00 | 1.82 | 1.21 | -0.37 | -1.00 | 0.51 | -1.05 | 0.34 | -0.39 | 1.29 | 0.71 | -0.39 | 0.41 | 0.07 |
| **G485** | 2.53 | 3.96 | 5.26 | 7.16 | 2.44 | 2.71 | 4.78 | 0.00 | 15.58 | 5.01 | 1.88 | 2.21 | 1.85 | 10.47 | 9.95 | 3.09 | 6.36 | 9.25 | 11.65 | 4.89 |
| **F486** | 3.17 | 3.27 | 3.16 | 3.98 | 3.29 | 3.50 | 3.32 | 4.43 | 3.30 | 2.27 | 2.04 | 1.93 | 1.70 | 0.00 | 6.69 | 4.17 | 3.21 | 2.34 | 0.51 | 2.94 |
| **Y489** | 3.86 | 3.73 | 4.43 | 5.59 | 2.75 | 3.47 | 4.16 | 5.37 | 2.56 | 1.97 | 1.44 | 3.53 | 0.76 | -0.19 | 5.92 | 4.27 | 2.79 | 1.78 | 0.00 | 1.57 |
| **F490** | 3.01 | 2.14 | 2.44 | 2.09 | 2.67 | 2.31 | 1.86 | 3.27 | 1.33 | 1.83 | 1.46 | 2.37 | 0.97 | 0.00 | 0.68 | 3.39 | 3.08 | 0.65 | 0.85 | 2.68 |
| **L492** | 3.55 | 4.90 | 2.93 | 3.56 | 3.04 | 3.31 | 2.66 | 5.30 | 2.95 | 1.30 | 0.00 | 3.26 | 0.64 | 0.73 | 5.91 | 5.14 | 4.08 | 4.43 | 2.26 | 2.01 |
| **Q493** | 1.63 | -0.57 | 1.47 | 1.35 | 0.45 | 0.00 | 1.79 | 2.75 | 0.26 | -0.23 | -1.45 | -0.52 | -1.07 | -0.54 | 5.75 | 1.37 | 1.60 | 6.57 | -0.36 | 0.22 |
| **S494** | -0.23 | 6.44 | 3.08 | 2.98 | 0.60 | 3.26 | 3.37 | 1.38 | 38.69 | 0.64 | 4.12 | 4.67 | 1.83 | 10.29 | 3.90 | 0.00 | 2.22 | 26.99 | 14.72 | -0.19 |
| **Y505** | 0.06 | -1.18 | 0.47 | 1.59 | 0.55 | 0.22 | 1.41 | 0.01 | 0.23 | 0.80 | -0.25 | -0.40 | -0.04 | -0.38 | -0.43 | 0.53 | 1.36 | 0.03 | 0.00 | 1.39 |

**Table S18. The predicted binding stabilities of SARS-CoV-2 RBD targeting CA1-B12 by FoldX.**

|  | **A** | **R** | **N** | **D** | **C** | **Q** | **E** | **G** | **H** | **I** | **L** | **K** | **M** | **F** | **P** | **S** | **T** | **W** | **Y** | **V** |
| --- | --- | --- | --- | --- | --- | --- | --- | --- | --- | --- | --- | --- | --- | --- | --- | --- | --- | --- | --- | --- |
| **R403** | 1.61 | 0.00 | 3.60 | 4.58 | 2.09 | 1.59 | 3.47 | 2.63 | 7.26 | 3.41 | 0.76 | 1.07 | -0.28 | 4.22 | 5.25 | 2.15 | 4.15 | 0.41 | 4.10 | 4.16 |
| **T415** | 1.43 | 0.67 | 0.43 | 1.57 | 1.00 | 1.16 | 1.22 | 0.94 | 1.14 | 0.38 | 0.81 | 0.38 | 0.87 | 0.58 | 1.94 | 0.63 | 0.00 | 1.18 | 0.65 | 0.72 |
| **G416** | 6.85 | 8.53 | 5.90 | 4.57 | 7.33 | 5.67 | 5.52 | 0.00 | 7.21 | 10.11 | 6.93 | 7.72 | 6.22 | 5.49 | 15.13 | 7.10 | 9.81 | 5.99 | 5.65 | 11.10 |
| **K417** | 1.99 | 1.46 | 0.51 | 2.45 | 1.88 | 1.29 | 1.88 | 3.04 | 3.46 | 0.57 | 1.00 | 0.00 | 0.55 | 3.71 | 0.07 | 2.85 | 2.22 | 6.58 | 1.83 | 1.13 |
| **D420** | 1.45 | 2.29 | 1.85 | 0.00 | 1.29 | 2.58 | 2.19 | 2.70 | 3.58 | 2.94 | 0.40 | 1.40 | -0.25 | 0.32 | 7.83 | 2.65 | 2.08 | 1.47 | 0.67 | 1.38 |
| **Y421** | 2.93 | 3.59 | 2.96 | 3.94 | 2.84 | 2.46 | 3.72 | 3.82 | 2.60 | 3.42 | 0.25 | 2.10 | 0.22 | -0.56 | 4.18 | 3.92 | 3.81 | 4.69 | 0.00 | 4.13 |
| **Y453** | 4.57 | 3.90 | 4.34 | 6.40 | 4.16 | 3.74 | 5.42 | 5.84 | 2.98 | 4.81 | 2.09 | 3.91 | 1.58 | 0.47 | 6.06 | 5.42 | 4.27 | 1.94 | 0.00 | 4.74 |
| **L455** | 3.81 | 4.03 | 2.93 | 3.39 | 2.31 | 3.01 | 3.35 | 4.71 | 5.79 | 1.28 | 0.00 | 2.80 | 0.47 | 7.01 | 7.38 | 4.50 | 4.67 | 20.05 | 12.66 | 2.39 |
| **F456** | 4.51 | 3.55 | 4.49 | 4.74 | 3.82 | 3.39 | 3.21 | 6.10 | 3.00 | 5.35 | 0.93 | 2.74 | 1.18 | 0.00 | 6.47 | 5.44 | 4.92 | -0.27 | -0.46 | 3.57 |
| **R457** | 1.65 | 0.00 | 2.13 | 2.25 | 2.01 | 1.25 | 1.39 | 3.08 | 1.12 | 4.41 | 0.35 | 1.44 | 0.43 | 0.41 | 7.74 | 1.72 | 2.70 | 3.84 | 0.75 | 3.80 |
| **K458** | 0.00 | 0.07 | 0.44 | -0.48 | 0.46 | -0.13 | -0.82 | 0.29 | 0.47 | -0.15 | -0.44 | 0.00 | -0.56 | -0.64 | -1.26 | 0.39 | 0.29 | -0.61 | -0.89 | 0.26 |
| **N460** | -0.01 | -0.58 | 0.00 | 0.54 | 0.19 | -0.29 | -0.08 | 0.41 | 0.05 | -0.04 | 0.02 | -0.66 | -0.52 | 0.31 | -0.98 | -0.35 | 0.48 | 0.53 | 0.20 | 0.35 |
| **Y473** | 3.29 | 4.18 | 3.81 | 3.63 | 3.03 | 2.98 | 3.73 | 4.28 | 2.41 | 2.14 | 1.61 | 2.17 | 0.65 | 0.52 | 6.20 | 4.35 | 3.67 | 1.83 | 0.00 | 2.61 |
| **A475** | 0.00 | 5.66 | 2.26 | 1.25 | 0.17 | 3.59 | 1.12 | 1.13 | 41.08 | 3.69 | -2.16 | 2.47 | -0.90 | 9.85 | 3.11 | 1.54 | 2.42 | 12.95 | 12.02 | 1.95 |
| **G476** | 1.22 | 3.24 | 2.18 | 1.31 | 1.03 | 2.03 | 1.78 | 0.00 | 7.14 | 2.52 | 2.75 | 2.38 | 2.66 | 1.54 | 4.57 | 1.31 | 2.65 | 4.21 | 1.94 | 1.75 |
| **F486** | 0.90 | 0.82 | 1.04 | 0.72 | 1.12 | 0.78 | 0.59 | 1.22 | 0.76 | 0.75 | 0.57 | 0.56 | 0.28 | 0.00 | 0.27 | 1.01 | 0.98 | 0.85 | 0.02 | 1.04 |
| **N487** | 3.01 | 3.90 | 0.00 | 2.48 | 2.04 | 2.58 | 2.29 | 2.10 | 3.10 | 4.76 | 1.57 | 3.24 | 2.02 | 7.18 | 6.99 | 2.87 | 4.10 | 9.41 | 7.92 | 3.95 |
| **Y489** | 0.60 | 1.65 | -0.28 | 0.95 | 0.52 | 1.09 | 0.20 | 1.69 | -0.23 | 1.99 | -0.48 | 0.78 | -0.49 | -0.61 | 0.72 | 0.53 | -0.15 | -1.11 | 0.00 | 0.91 |
| **Q493** | 1.73 | 1.69 | 1.97 | 1.15 | 1.50 | 0.00 | -0.04 | 2.65 | 1.47 | -0.50 | -0.51 | -0.03 | -0.76 | -1.15 | 5.07 | 1.70 | 1.60 | 4.31 | 3.18 | -0.29 |
| **S494** | -1.16 | -0.86 | -0.27 | -1.22 | -0.75 | -1.09 | -1.88 | -0.08 | 0.00 | -0.66 | -0.90 | -1.28 | -1.50 | -0.54 | -2.37 | 0.00 | 0.17 | 0.00 | -0.67 | -0.70 |
| **Y495** | 4.85 | 4.99 | 5.79 | 6.26 | 4.88 | 4.78 | 5.98 | 5.83 | 4.50 | 6.02 | 3.64 | 3.38 | 2.07 | 0.75 | 5.58 | 5.63 | 5.20 | 2.68 | 0.00 | 5.65 |
| **G496** | 1.88 | 3.27 | 2.79 | 2.62 | 2.21 | 2.39 | 2.89 | 0.00 | 2.76 | 4.36 | 3.00 | 2.43 | 2.29 | 2.33 | 7.46 | 2.84 | 3.88 | 2.74 | 2.63 | 4.19 |
| **Q498** | 0.53 | 0.71 | 1.53 | -0.64 | 1.29 | 0.00 | 0.26 | 1.41 | 0.02 | -0.58 | -0.79 | 0.18 | 0.48 | 1.29 | 3.39 | 1.66 | 0.83 | 2.50 | 1.68 | -0.21 |
| **T500** | -0.79 | -1.36 | -1.02 | -1.00 | -1.03 | -1.34 | -1.08 | -0.61 | -0.99 | -1.18 | -1.48 | -1.65 | -1.77 | -1.77 | -1.27 | -0.83 | 0.00 | -2.01 | -1.67 | -0.86 |
| **N501** | 0.84 | 3.24 | 0.00 | 0.78 | 0.91 | 1.68 | 2.04 | 2.00 | 5.21 | 1.56 | -0.20 | 1.94 | -0.70 | 3.84 | 6.11 | 0.93 | 0.55 | 9.87 | 8.06 | 1.64 |
| **G502** | -0.28 | 1.43 | 0.44 | 1.03 | 0.67 | 1.23 | 0.46 | 0.00 | 2.80 | 4.32 | 0.26 | 1.09 | -0.32 | -0.83 | 3.67 | 0.71 | 3.46 | 0.44 | -0.60 | 3.39 |
| **Y505** | 3.12 | 3.09 | 3.00 | 3.64 | 3.22 | 3.00 | 3.58 | 3.98 | 2.97 | 2.70 | 2.19 | 3.33 | 0.70 | -0.23 | 3.19 | 3.13 | 2.55 | 2.53 | 0.00 | 3.44 |

**Table S19. The predicted binding stabilities of SARS-CoV-2 RBD targeting CA1-B3 by FoldX.**

|  | **A** | **R** | **N** | **D** | **C** | **Q** | **E** | **G** | **H** | **I** | **L** | **K** | **M** | **F** | **P** | **S** | **T** | **W** | **Y** | **V** |
| --- | --- | --- | --- | --- | --- | --- | --- | --- | --- | --- | --- | --- | --- | --- | --- | --- | --- | --- | --- | --- |
| **R403** | 2.33 | 0.00 | 3.73 | 8.36 | 3.63 | 3.31 | 5.21 | 3.26 | 6.01 | 2.97 | 1.66 | 2.90 | 1.85 | 9.27 | 3.10 | 2.82 | 4.20 | 10.88 | 9.77 | 3.36 |
| **R408** | 1.07 | 0.00 | 1.11 | 1.52 | 1.30 | 0.61 | 1.42 | 1.26 | 1.29 | 1.45 | 1.25 | 0.36 | 1.14 | 1.53 | 0.28 | 1.41 | 1.48 | 1.53 | 1.58 | 1.57 |
| **T415** | 0.23 | -1.14 | -0.28 | 1.09 | -0.40 | -0.53 | -0.39 | 0.45 | -1.13 | -0.98 | -1.22 | -1.13 | -1.42 | -0.99 | 0.09 | -0.03 | 0.00 | -0.20 | -0.91 | -0.82 |
| **G416** | 5.95 | 7.44 | 5.59 | 5.57 | 5.51 | 6.04 | 3.91 | 0.00 | 8.70 | 10.86 | 8.61 | 6.41 | 4.57 | 6.36 | 13.78 | 7.84 | 7.70 | 9.58 | 6.61 | 11.42 |
| **K417** | 1.51 | 0.61 | 1.51 | 2.67 | 1.85 | -0.13 | 1.19 | 2.63 | 3.14 | 0.11 | 0.46 | 0.00 | 0.00 | 2.31 | -0.57 | 2.27 | 2.11 | 2.83 | 4.76 | 1.04 |
| **D420** | 1.29 | 2.10 | 1.57 | 0.00 | 0.82 | 1.54 | 1.09 | 2.54 | 7.37 | 1.78 | -0.47 | 0.89 | 0.13 | 0.79 | 5.68 | 2.18 | 1.60 | 5.11 | 2.07 | 1.44 |
| **Y421** | 3.12 | 4.59 | 3.52 | 4.65 | 3.14 | 2.99 | 4.80 | 3.94 | 3.11 | 5.19 | 0.57 | 2.43 | 0.53 | -0.18 | 3.51 | 4.28 | 4.25 | 6.05 | 0.00 | 3.92 |
| **Y453** | 5.68 | 4.72 | 5.52 | 7.46 | 5.40 | 4.95 | 6.77 | 6.90 | 4.09 | 4.87 | 3.38 | 5.07 | 3.03 | 1.70 | 7.16 | 5.65 | 5.62 | 4.87 | 0.00 | 4.78 |
| **L455** | 4.10 | 9.76 | 2.41 | 3.21 | 3.25 | 2.67 | 3.56 | 5.14 | 6.43 | 1.64 | 0.00 | 3.55 | 1.45 | 9.72 | 7.11 | 5.24 | 3.70 | 25.15 | 15.52 | 3.31 |
| **F456** | 4.92 | 4.50 | 4.94 | 5.06 | 4.16 | 3.71 | 4.10 | 6.42 | 3.98 | 4.72 | 1.49 | 3.42 | 1.52 | 0.00 | 7.10 | 5.86 | 4.44 | 1.00 | 0.03 | 3.52 |
| **R457** | 1.86 | 0.00 | 1.90 | 2.26 | 2.34 | 1.43 | 2.50 | 3.04 | 1.06 | 5.39 | 0.73 | 1.83 | 0.80 | 0.58 | 8.96 | 1.81 | 3.61 | 2.83 | 0.83 | 4.76 |
| **K458** | -0.31 | 0.06 | 0.33 | -0.21 | 0.33 | -0.28 | -0.86 | 0.32 | 0.54 | 0.09 | -0.19 | 0.00 | -0.39 | -0.37 | -1.99 | 0.32 | 0.26 | -0.19 | -0.41 | 0.23 |
| **N460** | -0.22 | -0.94 | 0.00 | 0.48 | -0.24 | -0.49 | 0.00 | 0.22 | -0.45 | -0.43 | 1.79 | 0.70 | -0.98 | -0.20 | -0.98 | -0.33 | 0.07 | 0.17 | -0.16 | 0.64 |
| **Y473** | 2.66 | 3.30 | 3.25 | 2.98 | 2.49 | 2.77 | 3.23 | 3.82 | 2.14 | 1.20 | 1.47 | 3.37 | 0.88 | 0.26 | 6.08 | 3.71 | 3.07 | 1.64 | 0.00 | 1.50 |
| **Q474** | 0.14 | 0.17 | 0.40 | -0.38 | 0.08 | 0.00 | -0.49 | 0.12 | 1.80 | -0.45 | -0.30 | -0.29 | -0.12 | 1.02 | -0.82 | 0.69 | 0.35 | 1.26 | 1.76 | -0.08 |
| **A475** | 0.00 | 13.09 | 10.02 | 4.34 | 0.16 | 3.01 | 3.90 | 0.92 | 17.68 | 6.58 | 0.19 | 7.95 | 2.94 | 19.35 | 4.21 | 0.61 | 2.58 | 18.53 | 17.57 | 3.49 |
| **G476** | 1.95 | 3.20 | 2.71 | 3.21 | 2.13 | 2.57 | 3.51 | 0.00 | 5.84 | 2.94 | 3.86 | 3.05 | 2.20 | 2.21 | 6.28 | 1.31 | 2.21 | 4.83 | 2.49 | 1.70 |
| **F486** | 1.12 | 0.97 | 1.26 | 1.15 | 1.18 | 0.81 | 0.49 | 1.43 | 0.71 | 1.14 | 0.79 | 0.85 | 0.30 | 0.00 | 0.79 | 1.26 | 1.25 | 0.75 | 1.21 | 1.30 |
| **N487** | 3.31 | 3.46 | 0.00 | 2.47 | 2.28 | 1.83 | 1.89 | 1.84 | 3.60 | 4.46 | 0.74 | 2.93 | 1.77 | 5.39 | 7.14 | 3.23 | 4.44 | 7.89 | 6.98 | 4.21 |
| **Y489** | 1.94 | 1.30 | 1.41 | 2.51 | 1.82 | 1.13 | 0.82 | 3.17 | 0.81 | 1.50 | -0.45 | 0.99 | 0.20 | -0.74 | 3.05 | 2.80 | 3.16 | -0.22 | 0.00 | 1.72 |
| **Q493** | 1.88 | 3.15 | 1.39 | 0.89 | 1.50 | 0.00 | -1.06 | 2.76 | 2.96 | -0.34 | -0.33 | -0.63 | -0.02 | 1.06 | 6.55 | 1.77 | 0.76 | 4.41 | 2.51 | -0.20 |
| **G496** | 1.10 | 1.67 | 0.93 | 1.16 | 0.86 | 0.50 | 0.55 | 0.00 | 2.49 | 1.72 | 1.46 | 1.11 | 0.17 | 1.32 | 6.03 | 1.33 | 1.26 | 4.19 | 1.27 | 1.80 |
| **Q498** | -0.43 | -0.58 | -0.90 | 0.01 | -0.45 | 0.00 | 0.34 | -0.04 | 2.25 | -0.24 | -1.23 | 0.25 | -1.05 | 1.89 | 2.05 | -0.32 | 1.21 | 4.35 | 1.67 | 1.07 |
| **T500** | -0.73 | -1.06 | -0.89 | -0.90 | -0.94 | 0.00 | -0.96 | -0.62 | -0.82 | -1.04 | -1.30 | -1.37 | -1.45 | -1.81 | -1.45 | -0.79 | 0.00 | -1.59 | -1.59 | -0.78 |
| **N501** | 0.46 | 2.84 | 0.00 | 0.81 | 0.45 | -0.89 | 1.10 | 0.84 | 7.63 | 3.66 | -0.35 | 0.93 | -0.34 | 3.82 | 4.61 | 1.01 | 0.75 | 8.93 | 5.43 | 1.50 |
| **G502** | 1.46 | 3.52 | 2.23 | 1.61 | 2.00 | 0.99 | 1.79 | 0.00 | 5.71 | 10.60 | 3.13 | 2.98 | 2.78 | 8.74 | 6.86 | 2.59 | 3.27 | 3.25 | 8.93 | 5.82 |
| **Y505** | 2.84 | 2.41 | 2.05 | 3.08 | 3.26 | 2.90 | 3.40 | 2.74 | 3.27 | 2.80 | 3.39 | 2.59 | 1.98 | -0.75 | 1.86 | 2.33 | 2.86 | 2.69 | 0.00 | 3.08 |

**Table S20. The predicted binding stabilities of SARS-CoV-2 RBD targeting 47D1 by FoldX.**

|  | **A** | **R** | **N** | **D** | **C** | **Q** | **E** | **G** | **H** | **I** | **L** | **K** | **M** | **F** | **P** | **S** | **T** | **W** | **Y** | **V** |
| --- | --- | --- | --- | --- | --- | --- | --- | --- | --- | --- | --- | --- | --- | --- | --- | --- | --- | --- | --- | --- |
| **R346** | 0.72 | 0.00 | 0.50 | 0.29 | 0.43 | 0.19 | 0.53 | 0.64 | 0.78 | 0.01 | -0.08 | 0.08 | 0.07 | -0.21 | 1.35 | 0.38 | 0.07 | -0.38 | -0.05 | 0.50 |
| **Y351** | 1.59 | 2.89 | 3.19 | 3.34 | 2.13 | 2.70 | 2.67 | 3.00 | 2.60 | 3.19 | -0.41 | 2.62 | 0.27 | -0.25 | 3.34 | 2.27 | 2.52 | -0.52 | 0.00 | 0.60 |
| **Y449** | 1.25 | 2.07 | 0.94 | 1.83 | 1.48 | 1.50 | 1.20 | 1.57 | 1.39 | 2.12 | 0.57 | 1.77 | 0.43 | 0.12 | 1.37 | 1.27 | 0.94 | 0.61 | 0.00 | 1.89 |
| **N450** | -0.31 | -0.81 | 0.00 | -0.32 | -0.71 | -0.51 | 0.27 | -0.01 | 0.18 | -0.89 | -1.57 | -0.47 | -1.61 | -1.05 | 0.98 | -0.91 | -0.61 | -1.10 | -1.16 | -0.29 |
| **L452** | 3.37 | 1.88 | 3.45 | 4.92 | 3.01 | 2.53 | 1.48 | 4.35 | 2.87 | 2.50 | 0.00 | 1.86 | 0.28 | 0.89 | 5.25 | 3.77 | 3.32 | 2.43 | 1.84 | 2.96 |
| **T470** | 0.20 | 0.28 | 0.61 | 0.65 | -0.32 | 0.57 | 0.57 | 1.16 | 0.21 | -0.37 | -1.72 | 0.18 | -2.13 | -1.10 | 0.82 | 1.14 | 0.00 | -1.30 | -1.32 | -0.59 |
| **I472** | 2.96 | 8.76 | 4.56 | 5.39 | 2.82 | 4.67 | 5.53 | 3.97 | 15.65 | 0.00 | 1.42 | 5.53 | 0.51 | 15.63 | 1.39 | 4.57 | 3.16 | 19.12 | 19.10 | 0.96 |
| **N481** | 0.99 | 0.26 | 0.00 | 0.16 | 0.70 | -0.26 | -0.06 | 0.08 | 0.45 | 2.09 | 0.66 | 0.15 | 0.47 | 0.20 | 3.82 | 0.99 | 1.59 | 0.28 | 0.20 | 2.52 |
| **G482** | 9.66 | 11.95 | 21.82 | 15.84 | 17.85 | 14.02 | 14.27 | 0.00 | 13.64 | 19.85 | 12.52 | 12.84 | 12.71 | 12.43 | 19.32 | 15.77 | 24.82 | 12.36 | 12.67 | 19.46 |
| **V483** | 1.88 | 3.00 | 1.98 | 1.04 | 1.90 | 2.02 | 1.63 | 2.61 | 9.79 | 0.20 | -0.99 | 2.67 | 1.48 | 4.86 | 7.42 | 1.69 | -0.32 | 4.44 | 4.63 | 0.00 |
| **E484** | 2.50 | 3.83 | 3.66 | 2.65 | 2.93 | 2.68 | 0.00 | 3.89 | 4.04 | 5.83 | 3.33 | 3.58 | 2.19 | 3.71 | 7.18 | 3.04 | 2.12 | 5.01 | 3.20 | 3.46 |
| **F490** | 3.94 | 5.97 | 4.27 | 3.49 | 3.30 | 5.07 | 2.46 | 2.34 | 1.72 | 3.74 | 3.18 | 3.01 | 0.35 | 0.00 | 2.65 | 5.28 | 3.65 | 0.79 | 0.21 | 3.96 |
| **S494** | -0.54 | 1.66 | 0.91 | 0.55 | -0.09 | 1.17 | -1.72 | 0.70 | 1.51 | -0.45 | 0.60 | 2.16 | -0.66 | 1.59 | -1.25 | 0.00 | 0.34 | 0.59 | 2.13 | -0.64 |

**Table S21. The predicted binding stabilities of SARS-CoV-2 RBD targeting CV07-270 by mCSM-PPI2.**

|  | **A** | **R** | **N** | **D** | **C** | **Q** | **E** | **G** | **H** | **I** | **L** | **K** | **M** | **F** | **P** | **S** | **T** | **W** | **Y** | **V** |
| --- | --- | --- | --- | --- | --- | --- | --- | --- | --- | --- | --- | --- | --- | --- | --- | --- | --- | --- | --- | --- |
| **R346** | -1.04 | NA | -0.88 | -1.30 | -0.97 | -0.79 | -1.43 | -1.22 | -0.52 | -0.83 | -0.79 | 0.38 | -0.81 | -0.67 | -1.51 | -0.75 | -0.88 | -0.42 | -0.51 | -0.90 |
| **S349** | -0.66 | -0.18 | 1.05 | 0.37 | -0.47 | -0.44 | -0.35 | -0.69 | 0.08 | -0.33 | 0.38 | -0.35 | -0.43 | -0.50 | -0.34 | NA | -0.37 | -0.28 | -0.09 | -0.46 |
| **Y351** | -0.86 | -0.46 | -0.62 | -0.96 | -0.86 | -0.92 | -0.92 | -0.93 | -0.60 | -0.80 | -0.44 | -0.70 | -0.88 | 0.05 | -1.43 | -0.79 | -0.85 | 0.20 | NA | -0.83 |
| **K444** | -0.74 | -0.66 | -0.58 | -0.44 | -0.72 | -0.49 | -0.41 | -0.75 | -0.26 | -0.51 | -0.75 | NA | -0.94 | -0.30 | -0.67 | -0.61 | 0.60 | 0.16 | -0.11 | -0.61 |
| **G446** | -0.20 | -1.17 | -0.52 | -0.39 | -0.58 | -0.71 | -0.49 | NA | -0.30 | -0.19 | -0.62 | -0.95 | -0.19 | 0.12 | -0.52 | -0.45 | 0.03 | 0.34 | 0.03 | -0.19 |
| **G447** | -1.26 | -1.96 | -1.17 | -1.27 | -1.63 | -1.75 | -1.25 | NA | -1.22 | -1.89 | -0.22 | -1.81 | -1.74 | -1.06 | -2.05 | -1.14 | -1.59 | -1.17 | -0.99 | -1.65 |
| **N448** | -1.85 | -1.94 | NA | -1.00 | -1.87 | 0.92 | -1.76 | -1.92 | -0.64 | -1.86 | -1.81 | -1.85 | -2.03 | -1.97 | -2.40 | -1.63 | -1.58 | -1.89 | -1.48 | -2.16 |
| **Y449** | -2.69 | -2.55 | -2.68 | -3.31 | -2.83 | -2.76 | -3.38 | -2.89 | -1.80 | -2.59 | -1.98 | -2.61 | -2.60 | -0.71 | -4.04 | -2.62 | -2.71 | -0.96 | NA | -2.77 |
| **N450** | -1.45 | -1.27 | NA | 0.40 | -0.98 | -1.05 | -0.75 | -1.22 | -0.34 | -1.08 | -2.48 | -1.42 | -1.22 | -0.75 | -1.80 | -0.82 | -0.98 | -0.74 | -0.67 | -1.23 |
| **Y451** | -2.22 | -2.12 | -2.03 | -2.47 | -2.39 | -2.21 | -2.45 | -2.48 | -1.97 | -2.20 | -1.24 | -2.26 | -2.33 | -0.95 | -2.86 | -2.07 | -2.08 | -0.25 | NA | -2.25 |
| **L452** | -1.59 | -0.89 | -1.31 | -2.26 | -1.38 | -1.21 | -2.47 | -1.64 | -0.42 | -0.99 | NA | -0.16 | -1.40 | 1.25 | -1.15 | -1.16 | -1.07 | 1.51 | 1.10 | -1.28 |
| **T470** | -0.28 | -0.20 | -0.27 | -0.07 | -0.34 | -0.22 | -0.02 | -0.35 | 0.00 | -0.19 | -2.15 | -0.20 | -0.23 | -0.08 | -0.25 | 0.25 | NA | 0.22 | 0.06 | -0.21 |
| **E484** | -0.74 | -1.24 | -0.82 | 0.30 | -0.81 | -0.85 | NA | -0.62 | -0.28 | -0.53 | -0.19 | -1.13 | -0.67 | 0.02 | -0.46 | -0.87 | -0.81 | 0.13 | 0.00 | -0.53 |
| **F490** | -1.30 | -1.06 | -1.07 | -1.61 | -1.07 | -1.32 | -1.43 | -1.62 | -1.15 | -1.17 | -0.44 | -1.20 | -1.25 | NA | -2.13 | -1.11 | -1.15 | 1.04 | 0.65 | -1.26 |
| **L492** | -1.46 | -1.23 | -1.23 | -2.09 | -1.64 | -1.12 | -2.04 | -1.66 | -0.60 | -0.97 | -1.20 | -1.14 | -1.17 | 0.83 | -1.76 | -1.30 | -1.26 | -0.11 | -0.08 | -1.16 |
| **S494** | -0.30 | 0.18 | 0.11 | 1.13 | -0.19 | 0.02 | -0.18 | -0.39 | 0.33 | -0.21 | -0.35 | 0.07 | -0.30 | -0.48 | 0.01 | NA | 0.94 | -0.05 | 0.02 | -0.20 |

**Table S22. The predicted binding stabilities of SARS-CoV-2 RBD targeting B38 by mCSM-PPI2.**

|  | **A** | **R** | **N** | **D** | **C** | **Q** | **E** | **G** | **H** | **I** | **L** | **K** | **M** | **F** | **P** | **S** | **T** | **W** | **Y** | **V** |
| --- | --- | --- | --- | --- | --- | --- | --- | --- | --- | --- | --- | --- | --- | --- | --- | --- | --- | --- | --- | --- |
| **R403** | -1.89 | NA | -1.98 | -2.29 | -1.81 | -2.08 | -2.20 | -2.19 | -0.55 | -1.27 | -1.55 | 0.51 | -1.56 | -1.07 | -1.80 | -1.75 | -1.61 | -0.57 | -0.96 | -1.73 |
| **D405** | -0.86 | -0.90 | -0.63 | NA | -0.73 | -0.62 | -0.22 | -0.71 | -0.25 | -0.48 | -0.56 | -0.77 | -0.54 | -0.26 | -0.46 | 0.25 | -0.53 | -0.07 | -0.19 | -0.61 |
| **T415** | -0.32 | NA | -0.35 | -0.16 | -0.52 | -0.41 | -0.34 | -0.47 | -0.62 | -0.27 | -0.53 | -0.58 | -0.23 | -0.51 | -0.62 | -0.42 | -0.07 | -0.02 | -0.41 | -0.43 |
| **G416** | -0.98 | -1.25 | -0.91 | -1.07 | -1.18 | -1.12 | -1.20 | -0.48 | -1.37 | -1.31 | -1.14 | -1.33 | -0.53 | -1.67 | -0.94 | -1.07 | NA | -0.41 | -0.24 | -1.20 |
| **K417** | -1.21 | -0.63 | -1.38 | -1.56 | -1.29 | -1.28 | -1.43 | -1.76 | 0.03 | -0.90 | NA | -0.83 | -1.45 | -0.61 | -1.71 | -1.29 | -0.98 | -0.55 | -0.34 | 0.75 |
| **D420** | -1.98 | -1.70 | -1.29 | NA | -1.59 | -1.69 | -0.60 | -1.62 | -1.09 | -1.46 | -1.44 | -1.87 | -1.34 | -1.41 | -1.89 | -1.84 | -1.84 | -1.29 | -1.11 | -1.46 |
| **Y421** | -2.26 | -2.34 | -2.20 | -2.89 | -2.36 | -2.37 | -2.84 | -2.57 | -2.12 | -2.28 | -2.32 | -2.39 | -2.30 | -0.88 | -3.51 | -2.30 | -2.31 | -0.84 | NA | -2.46 |
| **L455** | -1.32 | -1.01 | -1.04 | -2.04 | -1.25 | -1.22 | -2.07 | -1.41 | -0.71 | -0.84 | NA | -0.92 | -1.18 | 0.63 | -1.29 | 0.36 | -0.97 | 0.08 | 1.62 | -1.05 |
| **F456** | -1.62 | -1.71 | -1.40 | -1.75 | -1.53 | -1.37 | -1.71 | -1.74 | 0.78 | -1.52 | -1.70 | -1.47 | -1.54 | NA | -2.20 | -1.32 | -1.35 | -0.28 | 1.16 | -1.55 |
| **R457** | -1.07 | NA | -0.88 | -0.94 | -0.94 | -0.83 | -0.99 | -1.06 | -0.60 | -0.80 | -0.77 | -0.63 | -1.13 | -0.69 | -2.02 | -0.81 | -0.88 | -0.42 | -0.71 | -0.99 |
| **K458** | -0.45 | -0.06 | -0.19 | -0.47 | -0.46 | -0.14 | -0.46 | -0.51 | 0.57 | -0.33 | -0.41 | NA | -0.31 | -0.47 | -1.06 | -0.22 | -0.25 | -0.34 | -0.30 | -0.40 |
| **N460** | -0.55 | -0.02 | NA | -0.11 | -0.65 | -0.26 | -0.35 | -0.48 | -0.23 | -0.54 | -0.61 | 0.58 | -0.39 | -0.50 | -0.89 | -0.21 | -0.30 | -0.38 | -0.51 | -0.60 |
| **Y473** | -0.79 | -0.55 | -0.69 | -0.80 | -0.58 | -0.65 | -0.77 | -0.80 | -0.96 | -0.79 | -0.82 | -0.62 | -0.79 | -0.65 | -0.92 | -0.57 | -0.62 | 0.11 | NA | -0.83 |
| **A475** | NA | 0.66 | 1.03 | 1.65 | 0.17 | 0.77 | 1.51 | -0.91 | 0.56 | 0.30 | 0.41 | 0.50 | -0.38 | 1.31 | -1.04 | 0.35 | 0.47 | 1.75 | 1.06 | -0.17 |
| **G476** | -0.20 | -0.87 | -0.41 | -0.30 | -0.37 | -0.50 | -0.27 | NA | -0.29 | -0.29 | -0.37 | -0.67 | -0.28 | 0.07 | -0.65 | -0.34 | -0.44 | 0.44 | 0.10 | -0.38 |
| **F486** | -0.60 | NA | -0.43 | -0.41 | -0.90 | -0.47 | -0.39 | -0.91 | -0.62 | -0.41 | -0.54 | -0.61 | -0.30 | -0.56 | -0.45 | -0.33 | -0.33 | 0.01 | 0.01 | -0.55 |
| **N487** | -1.27 | -1.33 | -0.69 | -1.32 | -1.37 | -1.32 | -1.19 | -0.29 | -1.05 | -1.08 | -1.46 | -1.17 | -0.60 | NA | -0.92 | -1.29 | -1.35 | -0.45 | -0.67 | -1.01 |
| **Y489** | -1.54 | -1.46 | -1.42 | -1.53 | -1.34 | -1.35 | -1.53 | -1.55 | -1.61 | -1.63 | -1.61 | -1.34 | -1.61 | -1.00 | -1.48 | -1.22 | -1.24 | -0.37 | NA | -1.52 |
| **Q493** | -0.52 | -0.37 | 0.51 | -0.17 | -0.44 | NA | -0.01 | -0.57 | 0.08 | -0.44 | -0.43 | -0.66 | -0.44 | -0.22 | -0.44 | 0.45 | -0.29 | -0.01 | -0.01 | -0.46 |
| **Y495** | -1.10 | -1.06 | -0.92 | -1.40 | -1.06 | -1.26 | -1.34 | -1.29 | -1.23 | -1.09 | -1.11 | -1.16 | -1.17 | -0.52 | -1.52 | -1.13 | -1.07 | -0.37 | NA | -1.07 |
| **G496** | -0.89 | -1.33 | -1.03 | 0.83 | -1.16 | -1.34 | -0.70 | NA | -0.58 | -1.25 | -1.15 | -1.37 | -1.07 | -0.36 | -1.34 | -1.05 | -1.14 | -0.71 | -0.48 | -1.05 |
| **Q498** | -1.07 | -1.14 | 0.57 | -0.87 | -0.93 | NA | -0.81 | -1.16 | -0.48 | -0.83 | -0.88 | -1.25 | -1.00 | -0.31 | -1.63 | -0.82 | -1.02 | -0.53 | 2.12 | -0.98 |
| **T500** | -0.26 | -0.08 | 0.60 | -0.04 | -0.34 | -0.20 | -0.05 | -0.21 | 0.11 | -0.12 | -0.18 | -0.04 | -0.14 | -0.08 | -0.12 | 0.45 | NA | 0.07 | 0.05 | -0.11 |
| **N501** | -0.88 | -1.12 | NA | -0.62 | -0.98 | -1.02 | -0.92 | -0.97 | -0.57 | 0.80 | -1.00 | -1.21 | -0.76 | -0.72 | -1.35 | -0.59 | 0.53 | -0.97 | -0.76 | -0.02 |
| **G502** | -0.24 | -0.95 | -0.56 | -0.52 | -0.59 | -0.60 | -0.48 | NA | -0.47 | -0.77 | -0.79 | -0.84 | -0.71 | -0.41 | 0.77 | -0.22 | -0.67 | -0.43 | -0.29 | -0.54 |
| **Y505** | -2.60 | -2.54 | -2.56 | -3.29 | -2.51 | -2.53 | -3.19 | -2.77 | -1.93 | -2.46 | -2.35 | -2.51 | -2.48 | -1.17 | -3.88 | -2.63 | -2.64 | -1.40 | NA | -2.62 |

**Table S23. The predicted binding stabilities of SARS-CoV-2 RBD targeting CT-P59 by mCSM-PPI2.**

|  | **A** | **R** | **N** | **D** | **C** | **Q** | **E** | **G** | **H** | **I** | **L** | **K** | **M** | **F** | **P** | **S** | **T** | **W** | **Y** | **V** |
| --- | --- | --- | --- | --- | --- | --- | --- | --- | --- | --- | --- | --- | --- | --- | --- | --- | --- | --- | --- | --- |
| **R403** | -0.78 | NA | -0.80 | -0.80 | -0.75 | -0.79 | -0.88 | -0.81 | -0.07 | -0.58 | -0.64 | 0.40 | -0.78 | -0.46 | -1.11 | -0.64 | -0.70 | -0.19 | -0.41 | -0.77 |
| **K417** | -0.47 | -0.22 | -0.33 | -0.53 | -0.51 | -0.46 | -0.55 | -0.72 | -0.17 | -0.25 | -0.34 | NA | -0.39 | -0.29 | -1.35 | -0.31 | -0.34 | 0.14 | -0.17 | 0.01 |
| **Y449** | -1.87 | -1.66 | -1.54 | -2.05 | -1.56 | -1.84 | -1.93 | -2.19 | -1.28 | -1.65 | -1.70 | -1.86 | -1.80 | -0.33 | -3.22 | -1.84 | -1.74 | -0.30 | NA | -1.87 |
| **N450** | -0.19 | -0.16 | NA | -0.01 | -0.23 | -0.14 | -0.08 | -0.27 | 0.06 | -0.15 | -0.19 | -0.16 | -0.20 | -0.06 | -0.19 | -0.04 | -0.06 | 0.28 | 0.01 | -0.15 |
| **L452** | -1.44 | -0.66 | -1.08 | -1.79 | -1.12 | -0.97 | -1.95 | -1.41 | -0.62 | -0.99 | NA | 0.21 | -1.41 | 1.03 | -1.17 | -0.92 | -0.92 | 0.83 | 0.76 | -1.32 |
| **Y453** | -0.90 | -0.76 | -0.66 | -0.97 | -0.77 | -0.94 | -0.94 | -1.03 | -0.82 | -0.89 | -0.90 | -0.88 | -0.99 | -0.35 | -1.62 | -0.77 | -0.80 | -0.27 | NA | -0.94 |
| **L455** | -1.45 | -1.08 | -0.96 | -1.85 | -1.29 | -1.12 | -1.79 | -1.51 | -0.45 | -0.88 | NA | -0.93 | -1.22 | 0.72 | -1.26 | 0.36 | -0.82 | 1.15 | 1.42 | -1.04 |
| **F456** | -1.34 | -1.22 | -1.20 | -1.46 | -1.31 | -1.13 | -1.44 | -1.48 | 0.21 | -1.21 | -0.79 | -1.21 | -1.18 | NA | -2.08 | -1.19 | -1.17 | -0.06 | 0.45 | -1.25 |
| **E484** | -1.19 | -0.98 | -0.95 | -0.52 | -0.97 | -0.80 | NA | -1.10 | -0.39 | -0.53 | -0.63 | -1.09 | -0.82 | -0.20 | -0.65 | -0.94 | -1.00 | -0.17 | -0.03 | -0.97 |
| **G485** | -0.69 | -1.26 | -0.83 | -1.35 | -0.90 | -1.00 | -1.25 | NA | -0.50 | -0.25 | -0.46 | -1.14 | -0.56 | 0.09 | -0.44 | -0.69 | -0.87 | 1.08 | 0.09 | -0.60 |
| **F486** | -0.93 | -0.88 | -0.80 | -0.96 | -0.96 | -0.87 | -0.98 | -0.97 | -1.25 | -1.16 | -1.00 | -0.87 | -0.93 | NA | -1.16 | -0.78 | -0.82 | -0.25 | -0.86 | -0.94 |
| **Y489** | -1.85 | -1.78 | -1.74 | -2.13 | -1.87 | -1.84 | -2.19 | -1.81 | -1.66 | -1.81 | -1.76 | -1.84 | -1.82 | -1.09 | -1.66 | -1.74 | -1.72 | -0.15 | NA | -1.78 |
| **F490** | -1.53 | -1.36 | -1.28 | -1.75 | -1.29 | -1.59 | -1.55 | -1.85 | -1.43 | -1.45 | -1.47 | -1.46 | -1.45 | NA | -2.29 | -1.32 | -1.33 | -0.30 | 0.66 | -1.45 |
| **L492** | -1.67 | -1.61 | -1.52 | -2.41 | -1.61 | -1.44 | -2.38 | -1.84 | -1.05 | -1.31 | -1.05 | -1.48 | -1.35 | 0.82 | -2.26 | -1.50 | -1.48 | -0.62 | -0.42 | -1.52 |
| **Q493** | -1.35 | -0.98 | 0.70 | -0.84 | -1.36 | NA | -0.77 | -1.47 | -0.31 | -0.99 | -0.75 | -1.16 | -1.10 | -0.70 | -1.19 | 0.90 | -1.13 | -0.21 | -0.24 | -1.13 |
| **S494** | -0.46 | -0.26 | -0.40 | 1.19 | -0.56 | -0.57 | -0.30 | -0.80 | 0.07 | -0.60 | NA | -0.32 | -0.57 | -0.58 | -0.80 | NA | 1.59 | -0.04 | -0.22 | -0.52 |
| **Y505** | -0.58 | -0.32 | -0.25 | -0.63 | -0.42 | -0.47 | -0.61 | -0.80 | -0.27 | -0.53 | -0.61 | -0.30 | -0.65 | -0.08 | -1.31 | -0.33 | -0.33 | -0.42 | NA | -0.56 |

**Table S24. The predicted binding stabilities of SARS-CoV-2 RBD targeting CA1-B12 by mCSM-PPI2.**

|  | **A** | **R** | **N** | **D** | **C** | **Q** | **E** | **G** | **H** | **I** | **L** | **K** | **M** | **F** | **P** | **S** | **T** | **W** | **Y** | **V** |
| --- | --- | --- | --- | --- | --- | --- | --- | --- | --- | --- | --- | --- | --- | --- | --- | --- | --- | --- | --- | --- |
| **R403** | -1.41 | NA | -1.61 | -1.59 | -1.44 | -1.52 | -1.61 | -1.58 | -0.13 | -0.72 | -1.03 | 0.24 | -1.23 | -0.45 | -1.77 | -1.26 | -1.28 | -0.08 | -0.41 | -1.30 |
| **T415** | -0.28 | -0.51 | -0.25 | -0.37 | -0.42 | -0.32 | -0.49 | -0.45 | -0.17 | -0.20 | -0.31 | -0.29 | -0.30 | -0.39 | -0.55 | -0.22 | NA | 0.02 | -0.40 | -0.26 |
| **G416** | -0.90 | -1.17 | -0.94 | -1.03 | -1.01 | -1.05 | -1.08 | NA | -0.52 | -1.15 | -1.11 | -1.10 | -1.06 | -0.45 | -1.53 | -0.88 | -1.03 | -0.50 | -0.35 | -1.03 |
| **K417** | -2.30 | -1.64 | -2.57 | -2.79 | -2.08 | -2.34 | -2.47 | -2.66 | -0.60 | -1.57 | -1.51 | NA | -2.10 | -0.88 | -2.70 | -2.20 | -2.12 | -0.88 | -0.82 | 0.84 |
| **D420** | -1.72 | -1.45 | NA | NA | -1.17 | -1.38 | -0.18 | -1.30 | -0.76 | -0.92 | -0.89 | -1.71 | -1.01 | -0.84 | -1.36 | -1.58 | -1.54 | -0.59 | -0.79 | -1.06 |
| **Y421** | -2.08 | -2.01 | -1.86 | -2.55 | -2.08 | -2.11 | -2.45 | -2.45 | -2.04 | -2.22 | -2.20 | -2.11 | -2.20 | -0.83 | -3.30 | -2.00 | -2.02 | -0.92 | NA | -2.27 |
| **Y453** | -1.17 | -1.24 | -1.06 | -1.28 | -1.07 | -1.16 | -1.27 | -1.28 | -1.11 | -1.20 | -1.23 | -1.17 | -1.19 | -0.49 | -1.92 | -1.11 | -1.09 | -0.43 | NA | -1.18 |
| **L455** | -1.74 | -1.24 | -1.27 | -2.07 | -1.35 | -1.47 | -2.09 | -1.72 | -0.77 | -1.03 | NA | -1.19 | -1.31 | 0.96 | -1.20 | 0.78 | -1.14 | 0.18 | 1.64 | -1.28 |
| **F456** | -1.53 | -1.35 | -1.32 | -1.58 | -1.36 | -1.22 | -1.63 | -1.68 | 0.83 | -1.43 | -1.54 | -1.38 | -1.49 | NA | -2.04 | -1.14 | -1.16 | -0.28 | 1.17 | -1.43 |
| **R457** | -1.08 | NA | -0.92 | -1.04 | -1.01 | -0.82 | -1.07 | -1.06 | -0.50 | -0.80 | -0.74 | -0.62 | -1.04 | -0.56 | -1.94 | -0.76 | -0.92 | -0.25 | -0.58 | -0.92 |
| **L458** | -0.29 | -0.06 | -0.11 | -0.46 | -0.31 | -0.07 | -0.43 | -0.43 | 0.31 | -0.23 | -0.22 | NA | -0.29 | -0.14 | -1.05 | -0.18 | -0.22 | -0.11 | -0.10 | -0.30 |
| **N460** | -0.37 | -0.02 | NA | -0.11 | -0.48 | -0.16 | -0.29 | -0.44 | -0.15 | -0.26 | -0.34 | 0.33 | -0.25 | -0.20 | -1.08 | -0.14 | -0.19 | -0.20 | -0.14 | -0.35 |
| **Y473** | -0.63 | -0.49 | -0.54 | -0.57 | -0.49 | -0.47 | -0.59 | -0.63 | -0.87 | -0.65 | -0.67 | -0.45 | -0.64 | -0.56 | -0.59 | -0.45 | NA | 0.28 | NA | -0.63 |
| **A475** | NA | 0.76 | 1.05 | 1.48 | 0.21 | 0.85 | 1.75 | -0.96 | 0.88 | 0.25 | 0.24 | 0.56 | -0.56 | 1.45 | -0.86 | 0.35 | -0.41 | 1.75 | 1.15 | -0.27 |
| **G476** | -0.22 | -0.70 | -0.37 | -0.30 | -0.42 | -0.40 | -0.21 | NA | -0.18 | -0.30 | -0.40 | -0.57 | -0.26 | 0.13 | -0.62 | -0.35 | 0.61 | 0.44 | 0.14 | -0.39 |
| **F486** | -0.50 | -0.38 | -0.41 | -0.84 | -0.51 | -0.42 | -0.85 | -0.56 | -0.29 | -0.43 | -0.09 | -0.38 | -0.37 | -0.20 | -0.37 | -0.38 | -0.48 | 0.08 | -0.13 | -0.41 |
| **N487** | -1.01 | -1.27 | -1.28 | -0.50 | -1.07 | -1.19 | -1.02 | -0.96 | -0.05 | -0.67 | -0.67 | -1.22 | -0.92 | NA | -0.76 | -0.95 | -0.35 | 0.01 | -0.19 | -0.76 |
| **Y489** | -1.38 | -1.42 | 0.56 | -1.28 | -1.30 | -1.20 | -1.22 | -1.35 | -1.61 | -1.34 | -1.34 | -1.29 | -1.39 | -0.83 | -1.33 | -1.15 | -1.11 | -0.22 | NA | -1.37 |
| **Q493** | -0.96 | -0.78 | NA | -0.88 | -1.00 | NA | -0.64 | -1.08 | -0.06 | -0.65 | -0.68 | -0.87 | -0.67 | -0.51 | -0.57 | 0.64 | -1.15 | -0.16 | -0.20 | -0.78 |
| **S494** | -0.43 | 0.28 | 0.16 | 0.94 | -0.23 | 0.12 | 0.13 | -0.47 | 0.25 | -0.37 | -0.34 | 0.12 | -0.39 | -0.35 | -0.26 | NA | -0.88 | -0.04 | -0.07 | -0.38 |
| **Y495** | -1.22 | -1.34 | -1.11 | -1.43 | -1.19 | -1.23 | -1.39 | -1.38 | -1.34 | -1.44 | -1.35 | -1.21 | -1.32 | -0.72 | -1.90 | -1.14 | 0.83 | -0.37 | NA | -1.31 |
| **G496** | -1.34 | -1.96 | -1.22 | 0.98 | -1.54 | -1.63 | -0.93 | -1.21 | -0.88 | -1.87 | -1.72 | -1.80 | -1.60 | -1.03 | -1.80 | -1.52 | -1.09 | -1.16 | -0.95 | -1.50 |
| **Q498** | -1.25 | -1.32 | 0.78 | -1.05 | -1.09 | NA | -0.91 | NA | -0.48 | -1.10 | -1.19 | -1.31 | -1.10 | -0.35 | -1.70 | -0.93 | -1.49 | -0.61 | 2.13 | -1.11 |
| **T500** | -0.19 | -0.02 | 0.64 | 0.03 | -0.23 | -0.10 | 0.03 | -0.11 | 0.19 | -0.03 | -0.11 | -0.08 | -0.01 | 0.00 | -0.01 | 0.53 | -1.04 | 0.19 | 0.14 | -0.04 |
| **N501** | -1.19 | -1.54 | NA | -0.98 | -1.20 | -1.48 | -1.37 | -1.16 | -0.66 | 0.79 | -1.01 | -1.38 | -1.02 | -0.86 | -1.25 | -1.03 | 0.64 | -1.02 | -0.94 | -0.02 |
| **G502** | -0.38 | -0.81 | -0.53 | -0.40 | -0.69 | -0.57 | -0.58 | NA | -0.43 | -0.76 | -0.75 | -0.76 | -0.70 | -0.45 | 0.60 | -0.39 | -0.61 | -0.52 | -0.40 | -0.62 |
| **Y505** | -2.34 | -2.39 | -2.33 | -2.57 | -2.06 | -2.47 | -2.50 | -2.64 | -1.81 | -2.14 | -2.14 | -2.51 | -2.33 | -0.89 | -3.90 | -2.31 | -2.32 | -0.92 | NA | -2.32 |

**Table S25. The predicted binding stabilities of SARS-CoV-2 RBD targeting CA1-B3 by mCSM-PPI2.**

|  | **A** | **R** | **N** | **D** | **C** | **Q** | **E** | **G** | **H** | **I** | **L** | **K** | **M** | **F** | **P** | **S** | **T** | **W** | **Y** | **V** |
| --- | --- | --- | --- | --- | --- | --- | --- | --- | --- | --- | --- | --- | --- | --- | --- | --- | --- | --- | --- | --- |
| **R403** | -1.23 | NA | -1.22 | -1.21 | -1.17 | -1.35 | -1.28 | -1.32 | -0.28 | -0.88 | -0.99 | 0.22 | -1.30 | -0.58 | -1.92 | -1.13 | -1.17 | -0.35 | -0.54 | -1.18 |
| **R408** | -0.29 | NA | -0.34 | -0.46 | -0.35 | -0.28 | -0.53 | -0.48 | -0.18 | -0.32 | -0.33 | -0.17 | -0.46 | -0.14 | -1.23 | -0.26 | -0.31 | -0.04 | -0.19 | -0.39 |
| **T415** | -0.16 | -0.29 | -0.08 | -0.22 | -0.37 | -0.21 | -0.22 | -0.43 | -0.02 | -0.22 | -0.24 | -0.13 | -0.20 | -0.18 | -0.16 | 0.00 | NA | 0.23 | 0.05 | -0.20 |
| **G416** | -1.19 | -1.52 | -1.34 | -1.42 | -1.40 | -1.42 | -1.39 | NA | -0.88 | -1.42 | -1.40 | -1.42 | -1.34 | -0.79 | -1.74 | -1.32 | -1.36 | -0.92 | -0.71 | -1.29 |
| **K417** | -2.53 | -1.72 | -2.62 | -2.89 | -2.38 | -2.44 | -2.41 | -2.67 | -0.87 | -1.70 | -1.78 | NA | -2.24 | -1.14 | -2.63 | -2.31 | -2.40 | -1.09 | -0.93 | 1.13 |
| **D420** | -1.71 | -0.76 | -0.74 | NA | -0.58 | -1.43 | 0.15 | -1.18 | -0.29 | -0.16 | -0.16 | -1.28 | -0.21 | -0.31 | -0.87 | -1.29 | -1.16 | -0.25 | -0.26 | -0.22 |
| **Y421** | -2.34 | -2.32 | -2.36 | -3.02 | -2.36 | -2.48 | -2.78 | -2.63 | -2.14 | -2.43 | -2.35 | -2.40 | -2.37 | -0.99 | -3.68 | -2.29 | -2.33 | -0.97 | NA | -2.52 |
| **Y453** | -0.77 | -0.82 | -0.77 | -0.98 | -0.62 | -0.92 | -0.96 | -0.84 | -0.95 | -0.80 | -0.83 | -0.87 | -0.78 | -0.46 | -1.48 | -0.79 | -0.76 | -0.33 | NA | -0.77 |
| **L455** | -1.73 | -1.26 | -1.31 | -2.09 | -1.35 | -1.46 | -2.08 | -1.72 | -0.97 | -1.16 | NA | -1.24 | -1.36 | 0.73 | -1.19 | 0.87 | -1.18 | -0.07 | 1.64 | -1.33 |
| **F456** | -1.51 | -1.46 | -1.41 | -1.63 | -1.40 | -1.35 | -1.69 | -1.66 | 0.80 | -1.55 | -1.69 | -1.36 | -1.54 | NA | -2.07 | -1.19 | -1.20 | -0.28 | 1.14 | -1.50 |
| **R457** | -1.13 | NA | -1.04 | -1.09 | -1.03 | -1.00 | -1.12 | -1.27 | -0.41 | -0.86 | -0.89 | -0.77 | -1.10 | -0.48 | -2.29 | -0.86 | -0.99 | -0.25 | -0.56 | -1.03 |
| **K458** | -0.23 | -0.03 | -0.04 | -0.39 | -0.22 | -0.04 | -0.38 | -0.43 | 0.42 | -0.16 | -0.16 | NA | -0.21 | 0.05 | -1.07 | -0.12 | -0.15 | 0.07 | 0.05 | -0.25 |
| **N460** | -0.47 | -0.17 | NA | -0.05 | -0.62 | -0.22 | -0.30 | -0.46 | -0.22 | -0.40 | -0.45 | 0.56 | -0.26 | -0.40 | -0.83 | -0.21 | -0.23 | -0.26 | -0.43 | -0.49 |
| **Y473** | -0.77 | -0.65 | -0.74 | -0.75 | -0.64 | -0.71 | -0.74 | -0.77 | -1.01 | -0.89 | -0.84 | -0.74 | -0.81 | -0.69 | -0.88 | -0.66 | -0.70 | 0.15 | NA | -0.83 |
| **Q474** | -0.32 | 0.04 | -0.03 | 0.11 | -0.22 | NA | 0.08 | -0.25 | 0.02 | -0.26 | -0.39 | -0.09 | -0.27 | -0.17 | -0.27 | -0.03 | -0.07 | 0.27 | 0.10 | -0.31 |
| **A475** | NA | 0.52 | 0.94 | 1.57 | 0.13 | 0.70 | 1.64 | -0.93 | 0.48 | 0.22 | 0.14 | 0.35 | -0.60 | 1.40 | -1.11 | 0.24 | 0.44 | 1.66 | 1.04 | -0.33 |
| **G476** | -0.45 | -0.85 | -0.40 | -0.40 | -0.51 | -0.54 | -0.27 | NA | -0.47 | -0.47 | -0.58 | -0.64 | -0.50 | 0.13 | -0.88 | -0.36 | -0.54 | 0.51 | 0.12 | -0.59 |
| **F486** | -0.70 | -0.64 | -0.60 | -1.19 | -0.70 | -0.61 | -1.20 | -0.76 | -0.30 | -0.66 | -0.12 | -0.60 | -0.52 | NA | -0.60 | -0.55 | -0.52 | 0.07 | -0.13 | -0.62 |
| **N487** | -1.13 | -1.22 | NA | -0.58 | -1.24 | -1.27 | -1.25 | -1.14 | -0.13 | -0.83 | -0.76 | -1.33 | -0.99 | -0.26 | -0.89 | -1.09 | -1.27 | -0.17 | -0.34 | -0.84 |
| **Y489** | -1.63 | -1.55 | -1.58 | -1.56 | -1.46 | -1.44 | -1.46 | -1.55 | -1.90 | -1.64 | -1.68 | -1.43 | -1.69 | -1.06 | -1.59 | -1.39 | -1.38 | -0.28 | NA | -1.69 |
| **Q493** | -1.04 | -0.84 | 0.23 | -1.30 | -1.05 | NA | -1.07 | -1.09 | 0.05 | -0.61 | -0.64 | -1.07 | -0.72 | -0.40 | -0.56 | 0.26 | -1.11 | -0.02 | -0.10 | -0.73 |
| **G496** | -0.69 | -0.92 | -0.78 | 0.65 | -1.01 | -0.86 | -0.53 | NA | -0.65 | -1.17 | -1.13 | -0.89 | -1.01 | -0.57 | -1.15 | -0.73 | -0.90 | -0.68 | -0.30 | -0.93 |
| **Q498** | -0.99 | -0.85 | 0.98 | -0.76 | -0.80 | NA | -0.59 | -0.98 | -0.23 | -0.72 | -0.78 | -0.96 | -0.72 | -0.10 | -1.40 | -0.69 | -0.77 | -0.36 | 1.93 | -0.80 |
| **T500** | -0.23 | 0.11 | 0.53 | 0.05 | -0.19 | -0.01 | 0.09 | -0.14 | 0.16 | -0.01 | -0.02 | 0.07 | -0.04 | 0.04 | -0.03 | 0.46 | NA | 0.13 | 0.10 | -0.02 |
| **N501** | -0.86 | -1.34 | NA | -0.61 | -1.00 | -0.96 | -0.96 | -0.89 | -0.59 | 0.81 | -0.92 | -1.25 | -0.73 | -0.92 | -1.05 | -0.63 | 0.58 | -1.10 | -0.97 | -0.13 |
| **G502** | -0.56 | -0.83 | -0.62 | -0.74 | -0.86 | -0.59 | -0.75 | NA | -0.67 | -0.93 | -0.98 | -0.99 | -0.84 | -0.61 | 0.53 | -0.44 | -0.77 | -0.60 | -0.51 | -0.76 |
| **Y505** | -1.56 | -1.51 | -1.27 | -1.61 | -1.33 | -1.60 | -1.57 | -1.71 | -1.01 | -1.47 | -1.46 | -1.58 | -1.56 | -0.44 | -3.00 | -1.47 | -1.42 | -0.59 | NA | -1.60 |

**Table S26. The predicted binding stabilities of SARS-CoV-2 RBD targeting 47D1 by mCSM-PPI2.**

|  | **A** | **R** | **N** | **D** | **C** | **Q** | **E** | **G** | **H** | **I** | **L** | **K** | **M** | **F** | **P** | **S** | **T** | **W** | **Y** | **V** |
| --- | --- | --- | --- | --- | --- | --- | --- | --- | --- | --- | --- | --- | --- | --- | --- | --- | --- | --- | --- | --- |
| **R346** | -0.31 | NA | -0.42 | -0.51 | -0.36 | -0.35 | -0.55 | -0.60 | -0.19 | -0.32 | -0.34 | 0.22 | -0.35 | -0.13 | -1.14 | -0.33 | -0.34 | -0.05 | -0.16 | -0.40 |
| **Y351** | -0.33 | -0.28 | -0.30 | -0.49 | -0.28 | -0.40 | -0.42 | -0.45 | -0.53 | -0.37 | -0.37 | -0.28 | -0.43 | -0.14 | -0.95 | -0.32 | -0.29 | 0.14 | NA | -0.36 |
| **Y449** | -1.01 | -0.98 | -0.75 | -1.16 | -0.88 | -1.08 | -1.10 | -1.17 | -0.71 | -0.98 | -0.99 | -1.05 | -1.02 | -0.46 | -1.93 | -1.01 | -0.93 | -0.38 | NA | -1.01 |
| **N450** | -0.42 | -0.44 | NA | 0.72 | -0.24 | -0.29 | 0.26 | -0.25 | 0.31 | -0.13 | -0.25 | -1.11 | -0.23 | -0.05 | -0.08 | -0.19 | -0.25 | 0.03 | -0.07 | -0.21 |
| **L452** | -0.74 | -0.23 | -0.59 | -0.82 | -0.63 | -0.56 | -1.04 | -0.74 | -0.32 | -0.81 | NA | 0.33 | -0.80 | 0.96 | -0.73 | -0.48 | -0.46 | 1.19 | 0.71 | -0.89 |
| **T470** | -0.32 | -0.28 | -0.08 | -0.26 | -0.28 | -0.20 | -0.26 | -0.38 | 0.18 | -0.14 | -0.07 | -0.25 | -0.16 | -0.03 | -0.01 | 0.48 | NA | 0.26 | 0.18 | -0.20 |
| **I472** | -1.31 | -1.06 | -1.29 | -1.77 | -1.09 | -0.87 | 0.47 | -1.54 | -1.24 | NA | -1.34 | -1.12 | -1.50 | 0.13 | -1.45 | -1.24 | -1.05 | 0.92 | -0.03 | -1.36 |
| **N481** | -0.40 | -0.47 | NA | -0.01 | -0.56 | -0.38 | -0.35 | -0.47 | 0.07 | -0.29 | -0.30 | -0.59 | -0.34 | -0.18 | -1.16 | -0.23 | -0.37 | -0.39 | -0.24 | -0.38 |
| **G482** | -0.78 | -1.40 | -0.84 | -1.05 | -0.78 | -1.16 | -1.18 | NA | -0.72 | -1.03 | -0.97 | -1.45 | -0.90 | -0.56 | -0.80 | -0.83 | -0.97 | -0.38 | -0.51 | -0.95 |
| **V483** | -1.23 | -0.88 | -1.03 | -1.49 | -1.00 | -1.05 | -1.38 | -1.49 | -0.48 | -0.81 | -0.92 | -0.79 | -1.10 | -0.65 | -2.12 | -1.10 | -0.82 | -0.62 | -0.35 | NA |
| **E484** | -1.25 | -1.67 | -1.06 | 0.86 | -1.05 | -1.24 | NA | -1.10 | -0.61 | -0.79 | -0.90 | -1.65 | -0.95 | -0.37 | -0.96 | -1.05 | -1.12 | -0.03 | -0.23 | -1.04 |
| **F490** | -2.20 | -2.14 | -2.05 | -2.58 | -1.98 | -2.17 | -2.44 | -2.45 | -1.72 | -1.95 | -1.94 | -2.11 | -2.11 | NA | -3.29 | -2.03 | -2.09 | 1.31 | 0.88 | -2.09 |
| **S494** | -0.19 | 0.02 | -0.13 | 0.93 | -0.24 | -0.19 | -0.10 | -0.35 | 0.36 | -0.13 | -0.06 | -0.12 | -0.03 | -0.06 | 0.16 | NA | 0.44 | 0.27 | 0.09 | -0.10 |

**Table S27. The binding affinities of L455 variants of RBD targeting convalescent antibodies after MD simulation.** The binding affinity is in unit of Kcal/mol.

The highlighted values indicated the deceased binding affinities of L455 variants of RBD complexed with antibodies.

|  |  | **RBD Variants** | | | | | | | | | | | | | | | | | | | |
| --- | --- | --- | --- | --- | --- | --- | --- | --- | --- | --- | --- | --- | --- | --- | --- | --- | --- | --- | --- | --- | --- |
|  |  | **A** | **C** | **D** | **E** | **F** | **G** | **H** | **I** | **K** | **L** | **M** | **N** | **P** | **Q** | **R** | **S** | **T** | **V** | **W** | **Y** |
| **Antibodies** | **CA1-B3** | -15.02 | -13.93 | -12.63 | -13.25 | -15.46 | -13.74 | -12.81 | -11.63 | -13.87 | **-15.09** | -12.38 | -12.36 | -13.9 | -16.04 | -14.62 | -14.38 | -13.01 | -15.74 | -14.11 | -14.04 |
|  | **CA1-B12** | -14.73 | -13.09 | -13.48 | -12.88 | -12.51 | -13.73 | -16.02 | -15 | -13.92 | **-15.11** | -15.24 | -15.12 | -14.31 | -12.55 | -13.17 | -14.6 | -12.74 | -15.87 | -15.36 | -13.34 |
|  | **CT-P59** | -12.98 | -4.81 | -15.05 | -14.46 | -16.71 | -5.72 | -11.14 | -13.61 | -15.38 | **-15.21** | -13.22 | -13.19 | -14.53 | -10.84 | -10.73 | -9.8 | -12 | -14.61 | -14.09 | -11.99 |
|  | **B38** | -13.91 | -13.12 | -13.58 | -12.62 | -12.66 | -13.3 | -14.03 | -13.14 | -14.21 | **-14.04** | -11.11 | -14.38 | -13.16 | -12.19 | -14.03 | -9.72 | -13.46 | -13.06 | -11.97 | -13.1 |

**Table S28. The binding affinities of F456 variants of RBD targeting convalescent antibodies after MD simulation.** The binding affinity is in unit of Kcal/mol.

The highlighted values indicated the deceased binding affinities of F456 variants of RBD complexed with antibodies.

|  |  | **RBD Variants** | | | | | | | | | | | | | | | | | | | |
| --- | --- | --- | --- | --- | --- | --- | --- | --- | --- | --- | --- | --- | --- | --- | --- | --- | --- | --- | --- | --- | --- |
|  |  | **A** | **C** | **D** | **E** | **F** | **G** | **H** | **I** | **K** | **L** | **M** | **N** | **P** | **Q** | **R** | **S** | **T** | **V** | **W** | **Y** |
| **Antibodies** | **CA1-B3** | -12.08 | -12.62 | -11.81 | -11.65 | **-15.09** | -11.75 | -12.75 | -12.03 | -7.81 | -12.48 | -11.04 | -12.83 | -12.5 | -12.38 | -7.97 | -11.16 | -12.18 | -12.77 | -14.68 | -13.47 |
|  | **CA1-B12** | -10.66 | -11.98 | -12.3 | -12.56 | **-15.11** | -13.45 | -15.9 | -13.21 | -15.03 | -16.15 | -11.76 | -11.89 | -10.58 | -12.95 | -12.45 | -12.28 | -14.53 | -15.25 | -15.01 | -15.3 |
|  | **CT-P59** | -13.96 | -13.7 | -15.95 | -13.43 | **-15.21** | -14 | -14.8 | -14.93 | -15.57 | -14.77 | -6.54 | -13.34 | -12.48 | -14.32 | -14.14 | -14.62 | -14.2 | -15.12 | -16.54 | -12.56 |
|  | **B38** | -11.58 | -10.92 | -12.26 | -11.87 | **-14.04** | -11.67 | -13.64 | -13.37 | -11.59 | -11.59 | -14.34 | -11.69 | -13.07 | -13.88 | -12.39 | -13.04 | -13.43 | -13.89 | -13.54 | -13.54 |


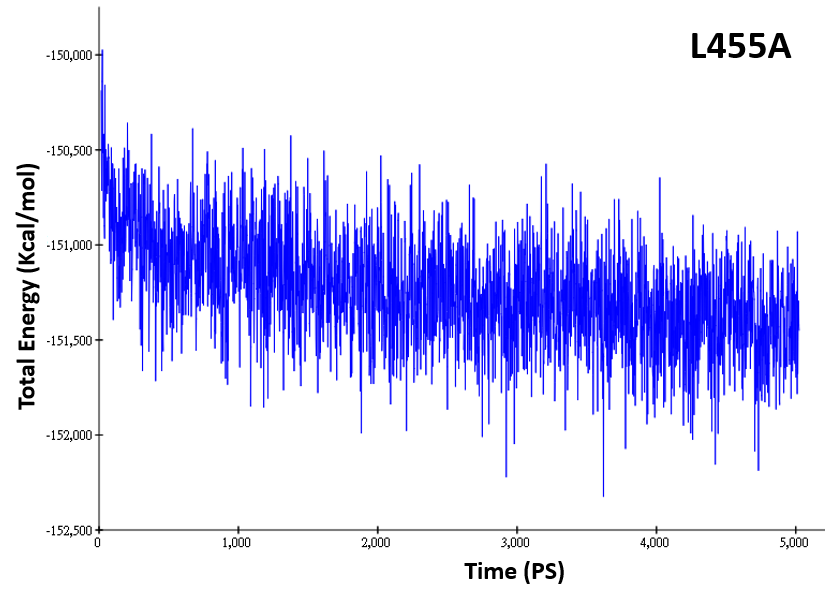

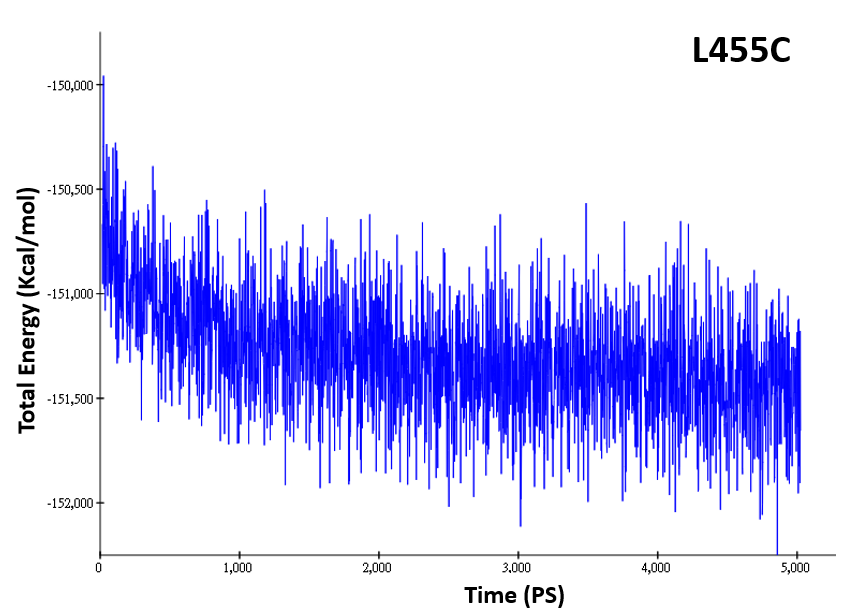

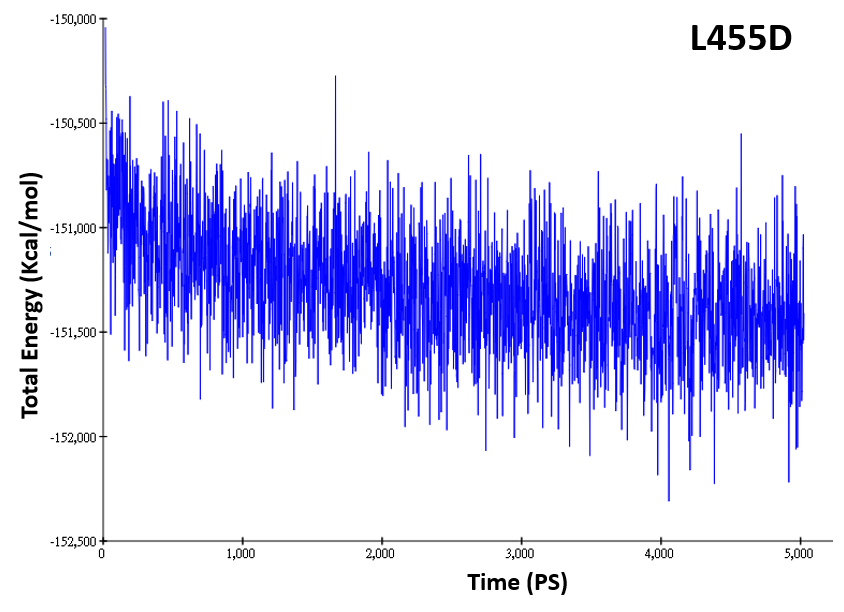


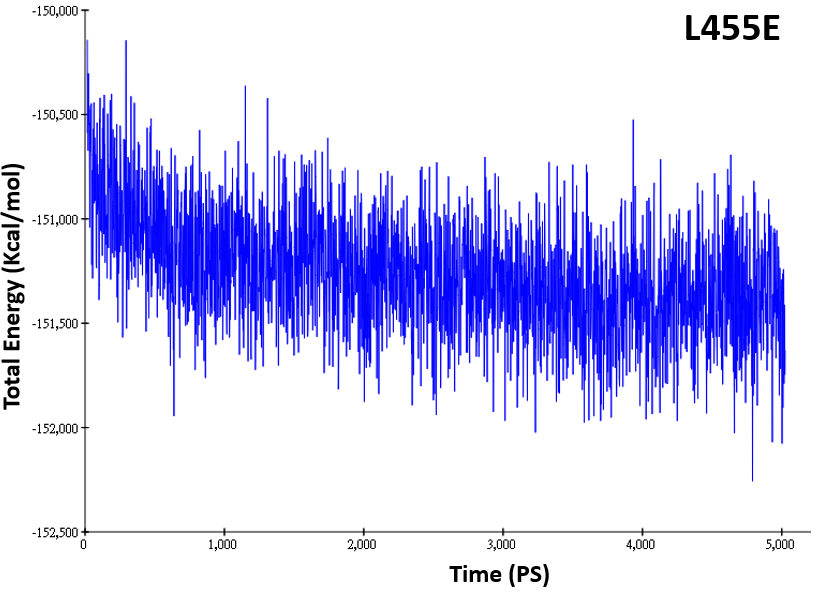

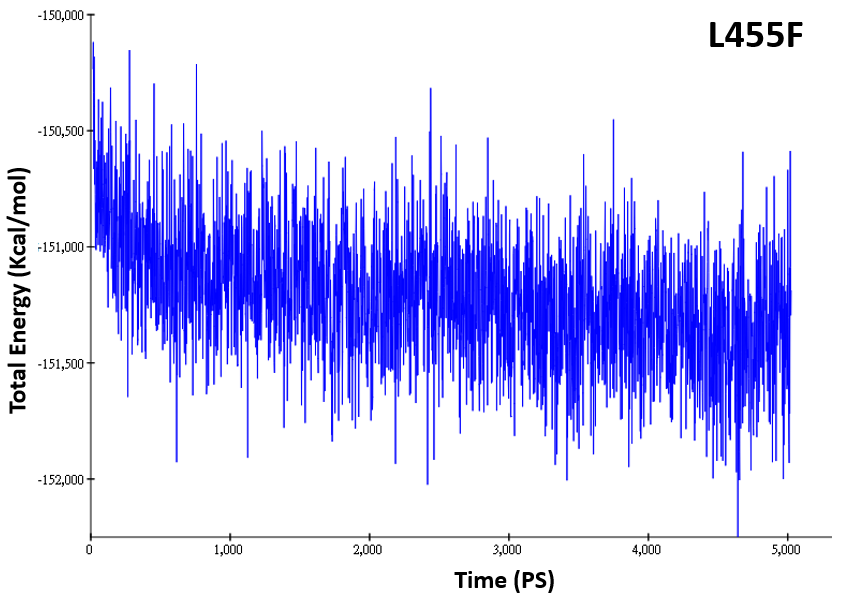

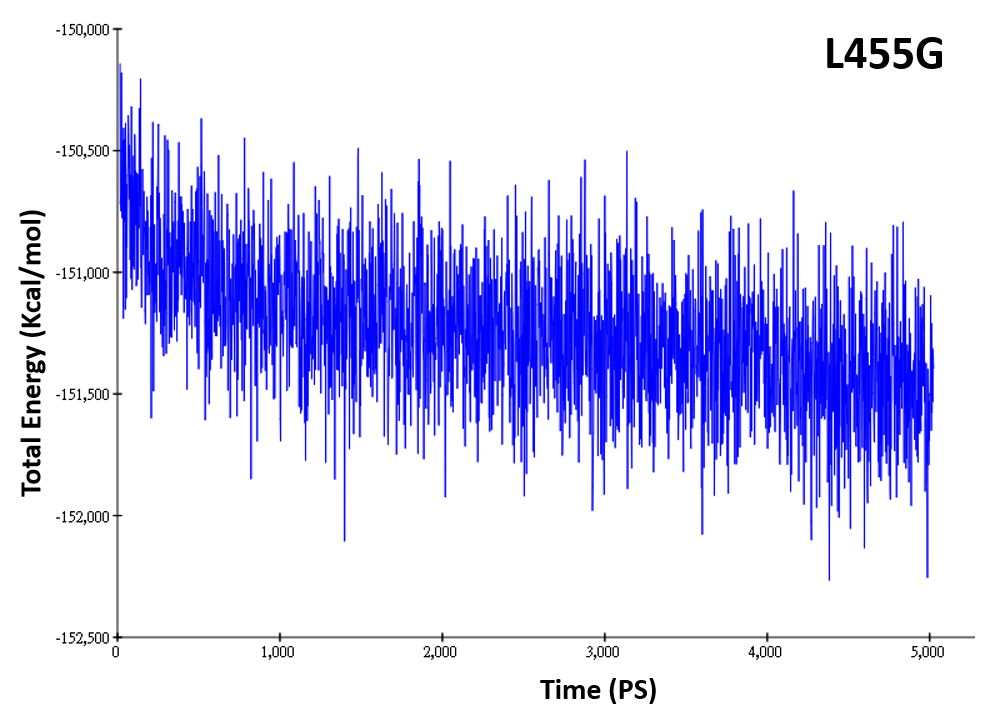


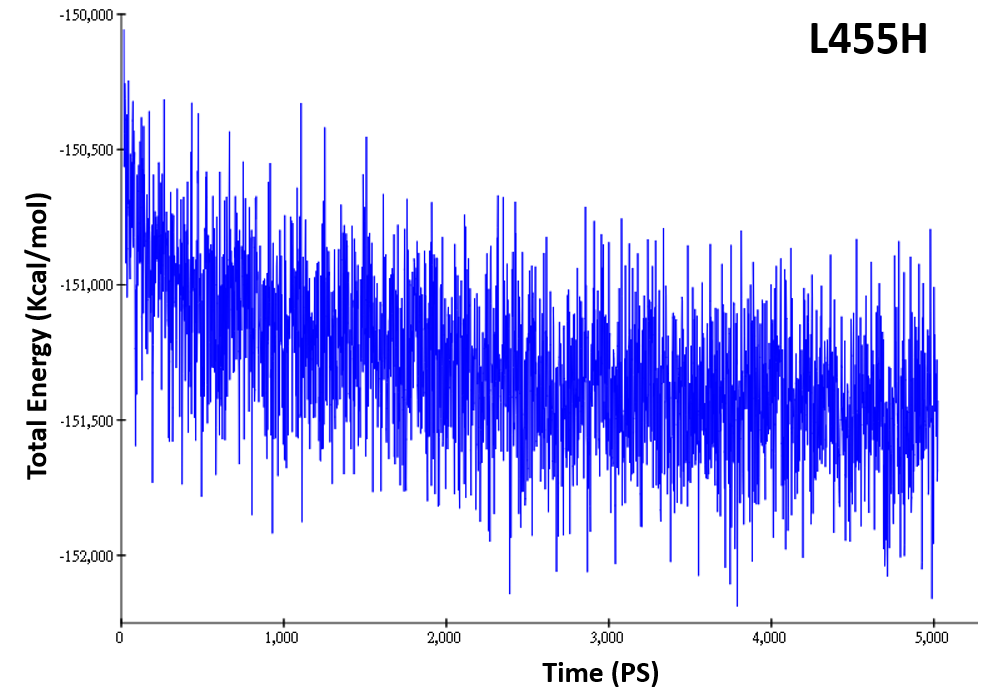

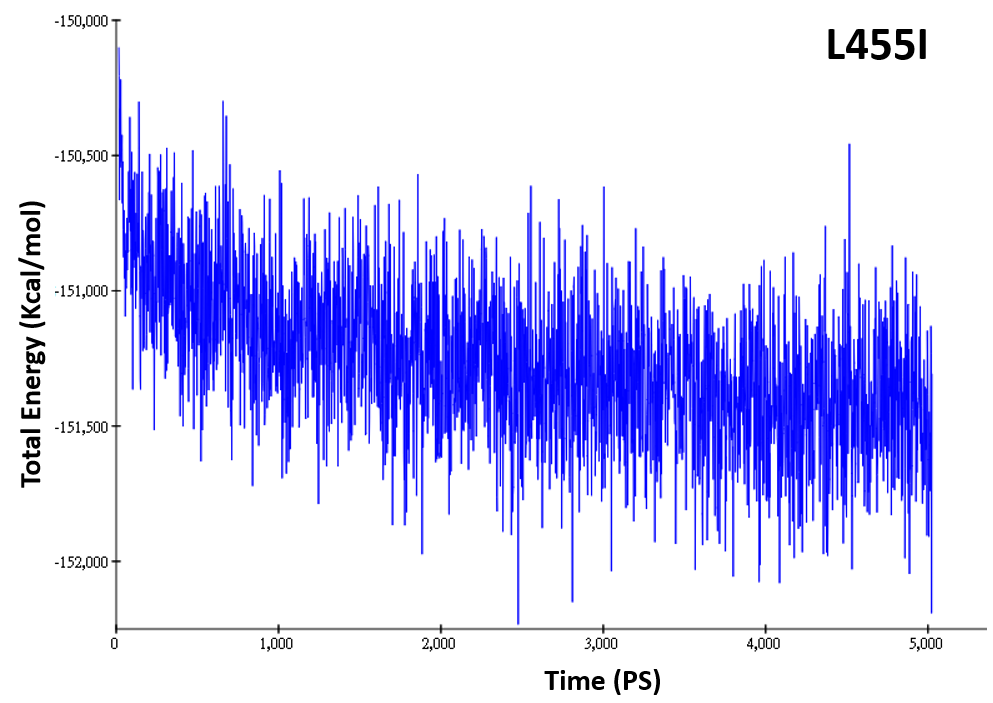

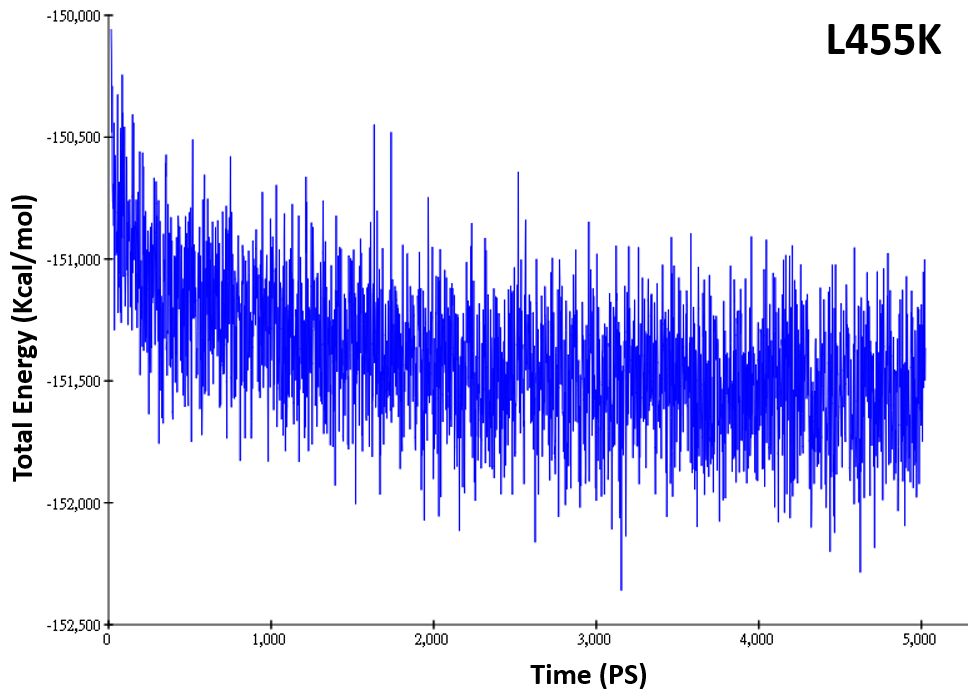


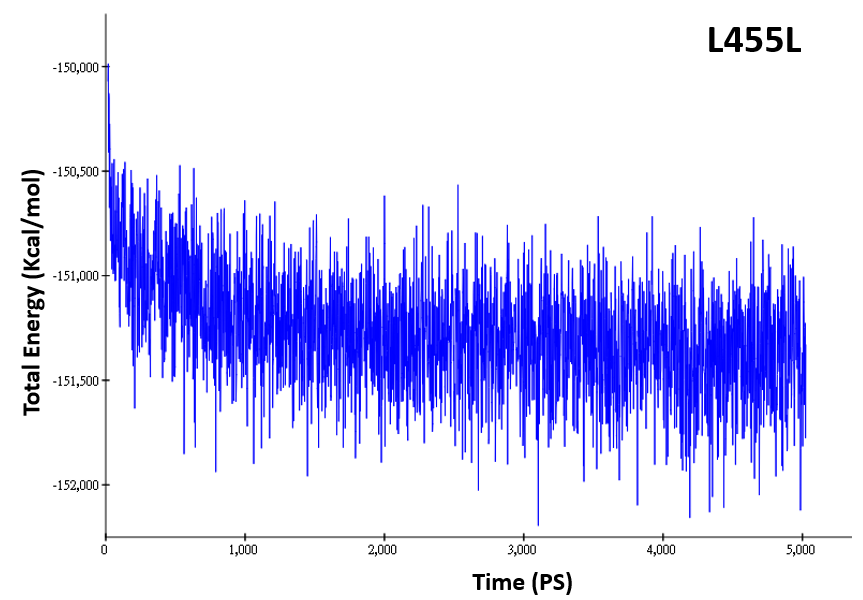


**Figure S1. The molecular dynamics simulations of L455 and its variants of RBD in complex with convalescent antibody CT-P59 (PDB ID: 7CM4).** The total energy as a function of simulation time for 5 ns is shown.


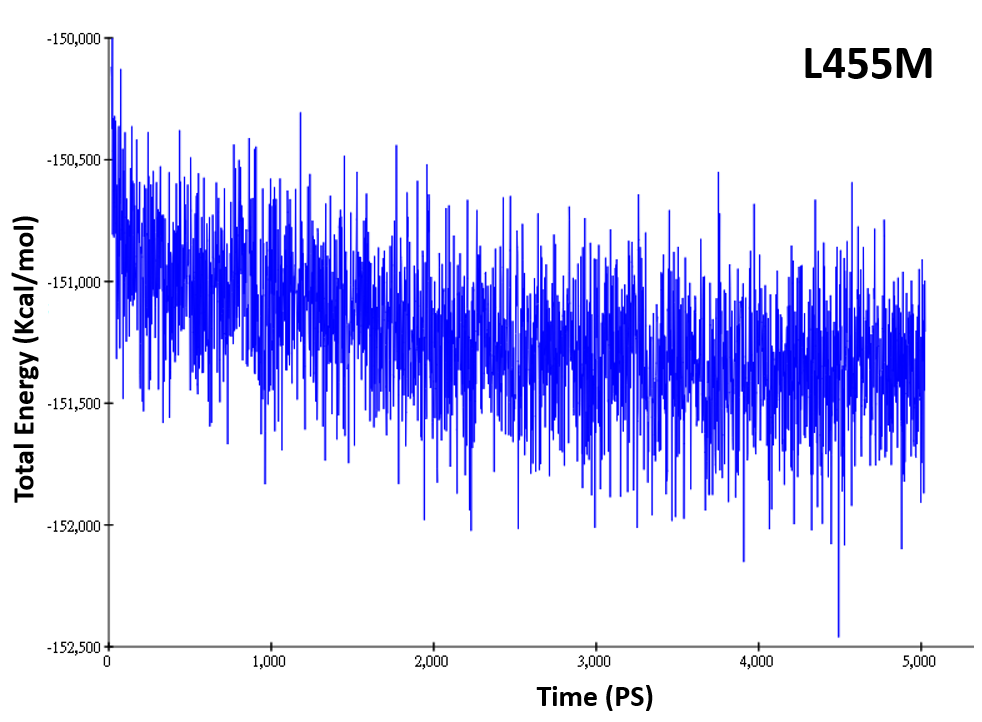

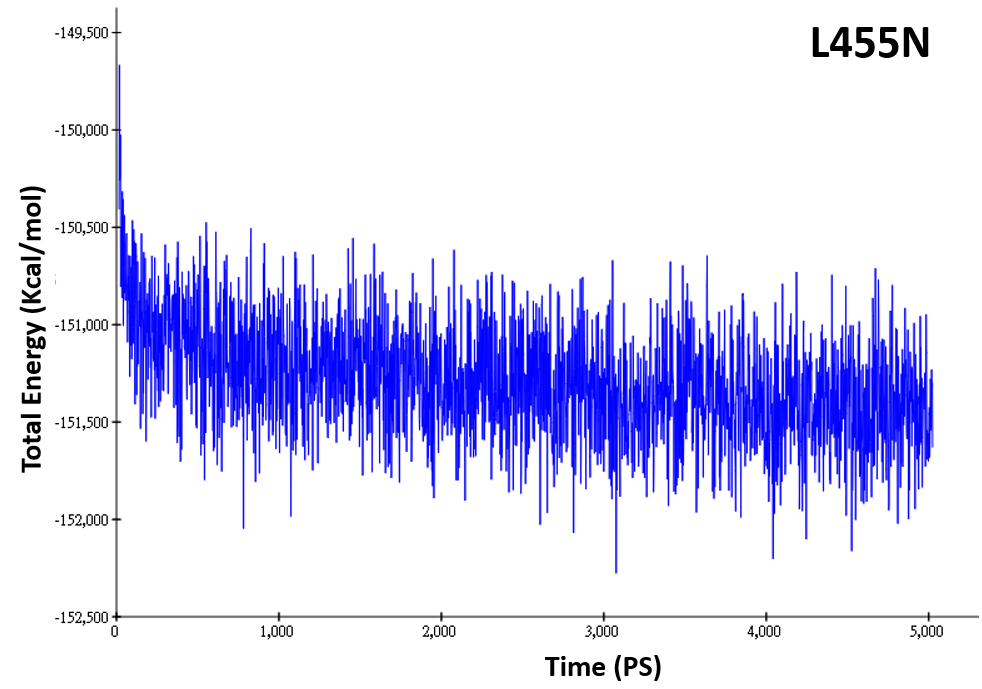

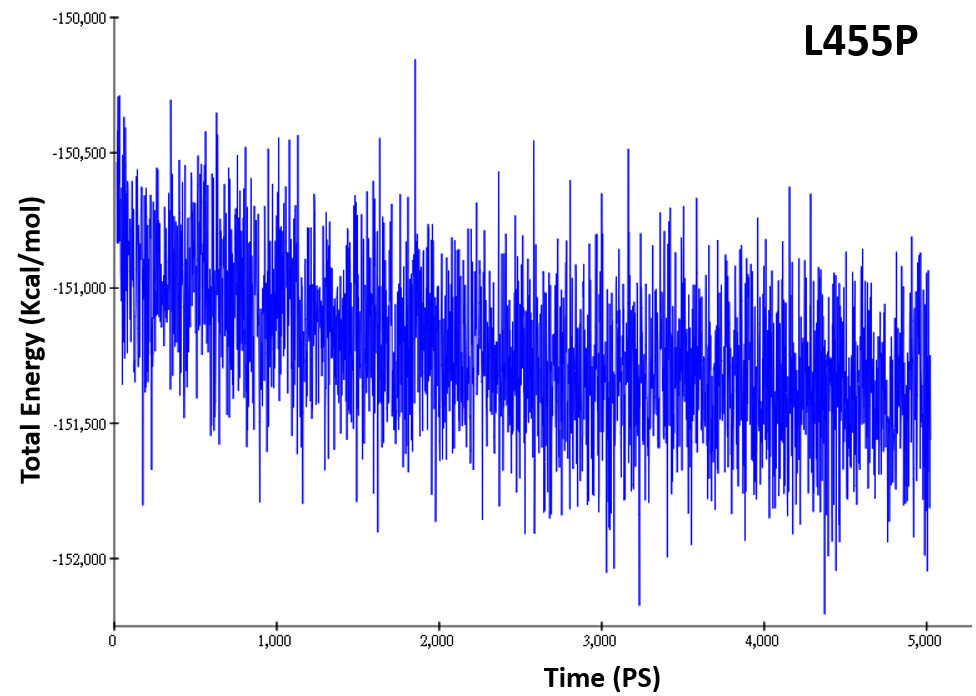


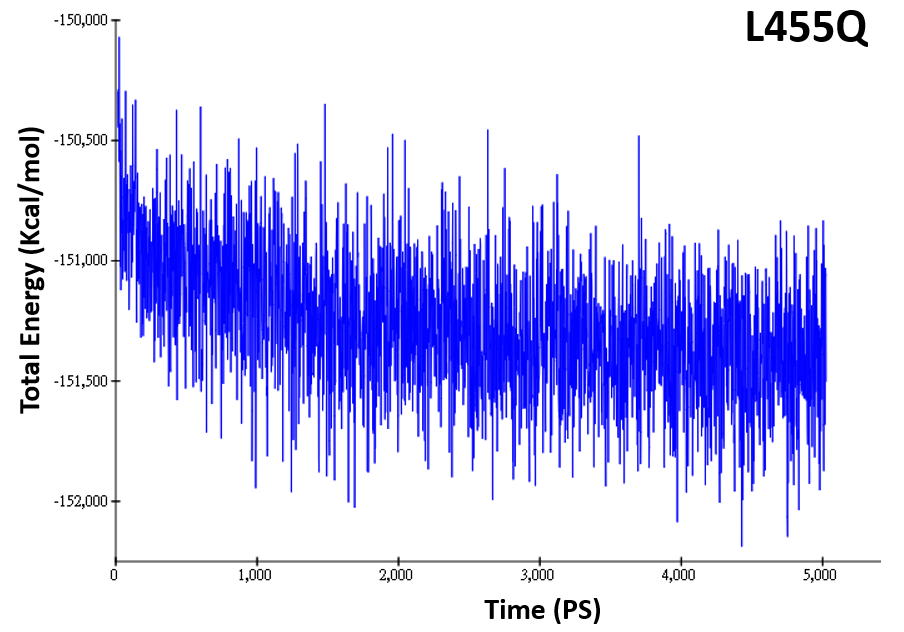

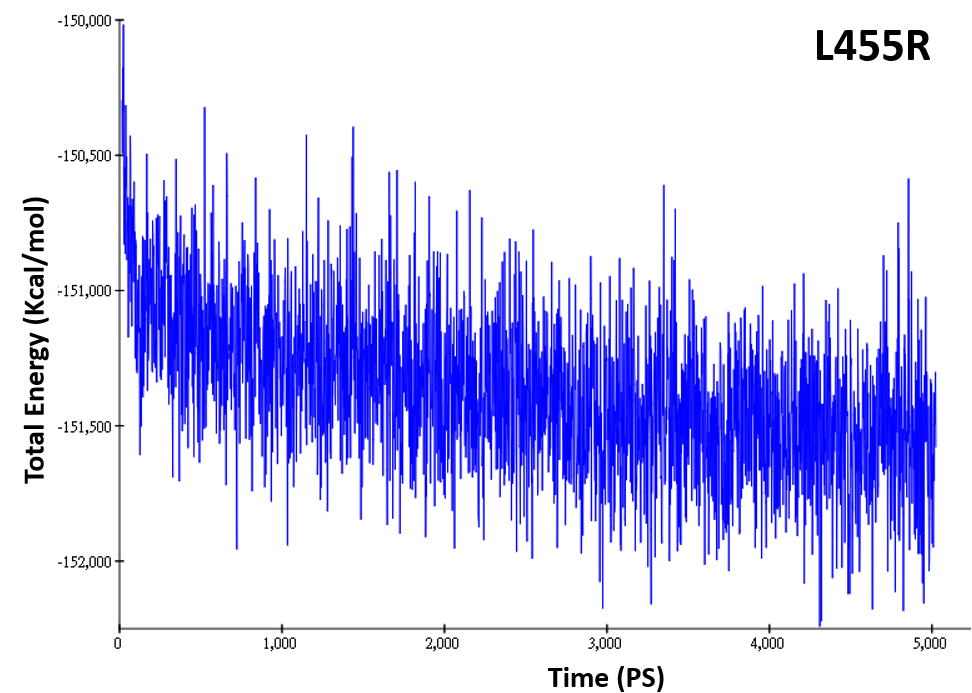

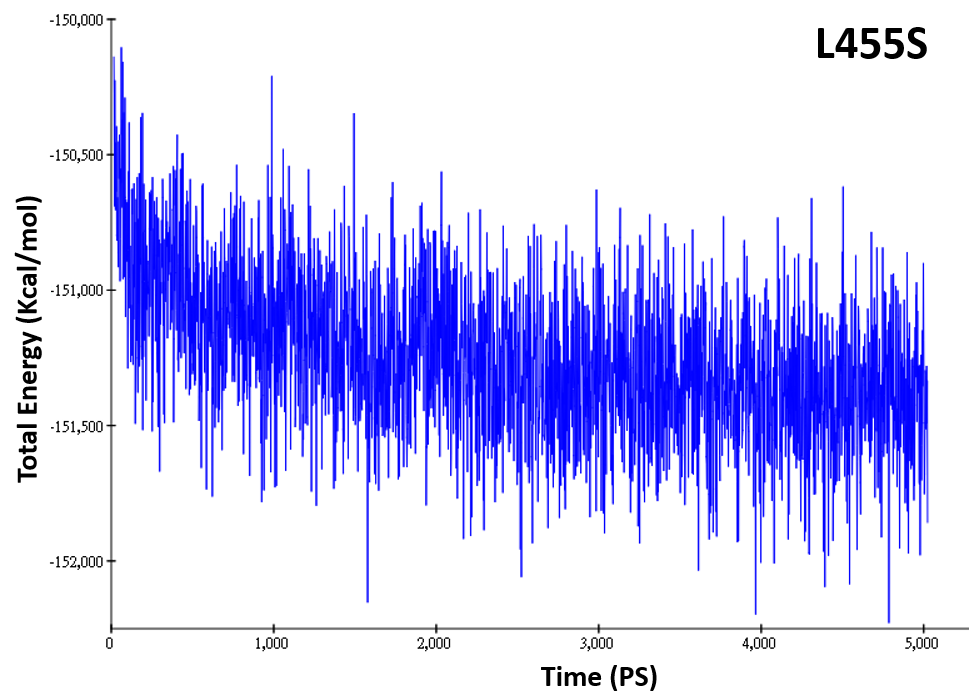

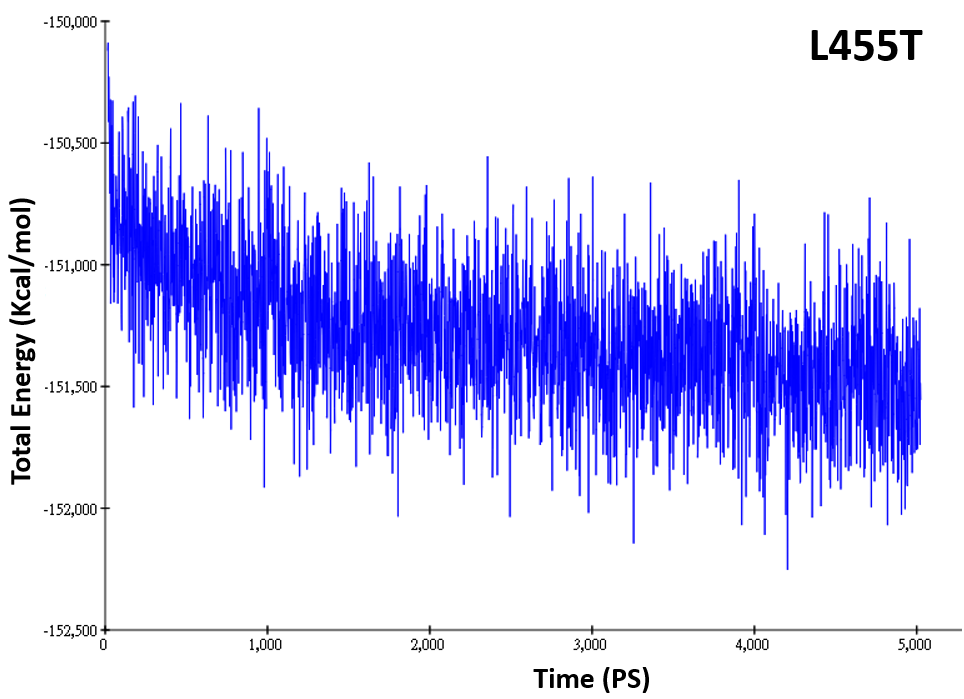

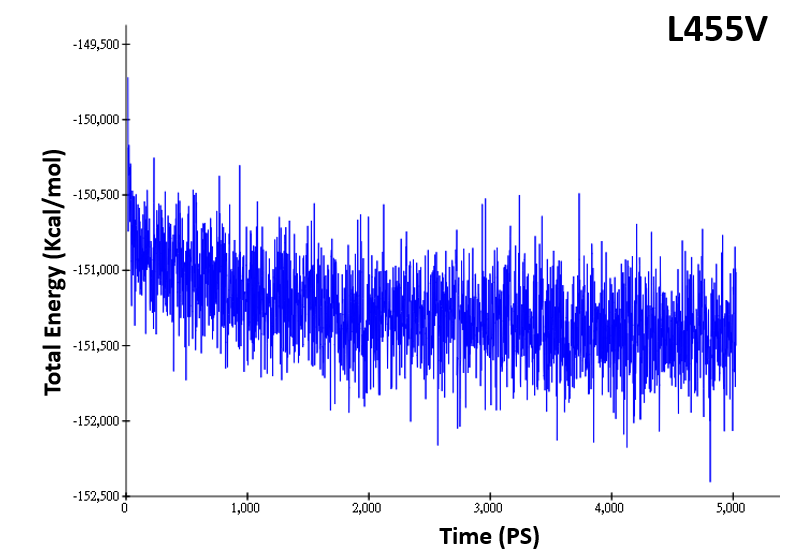

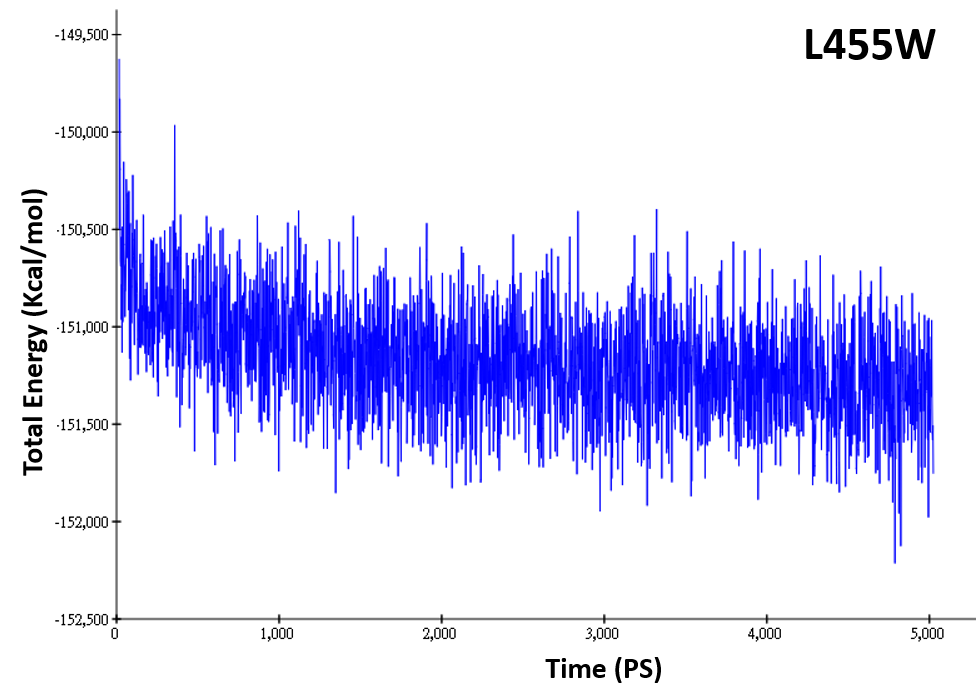


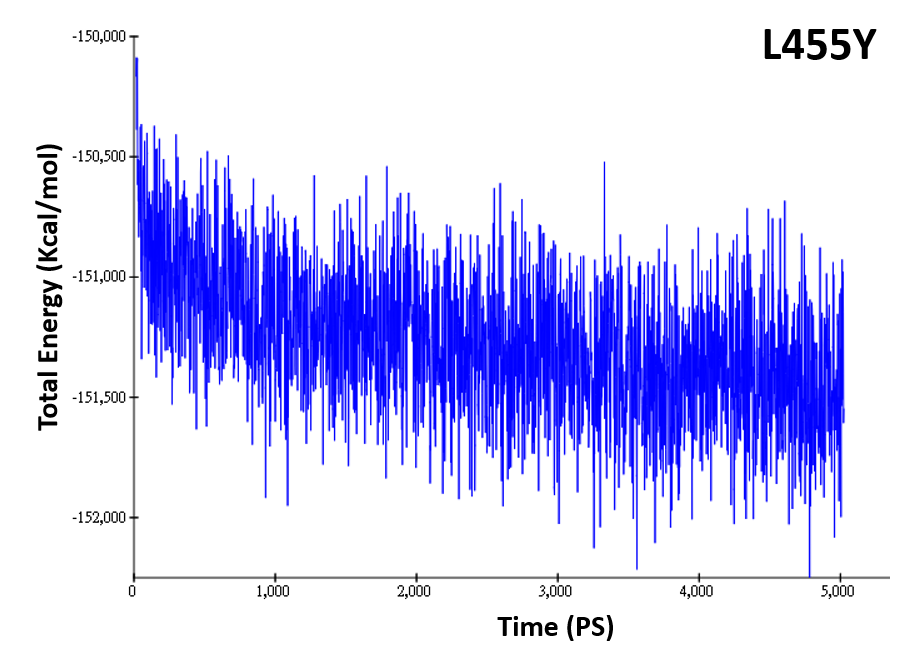


**Figure S2. The molecular dynamics simulations of L455 variants of RBD targeting convalescent antibody CT-P59 (PDB ID: 7CM4).** The trajectory profiles of total energies of L455 variants in complex with CT-P59 during MD simulation time for 5 ns are presented.


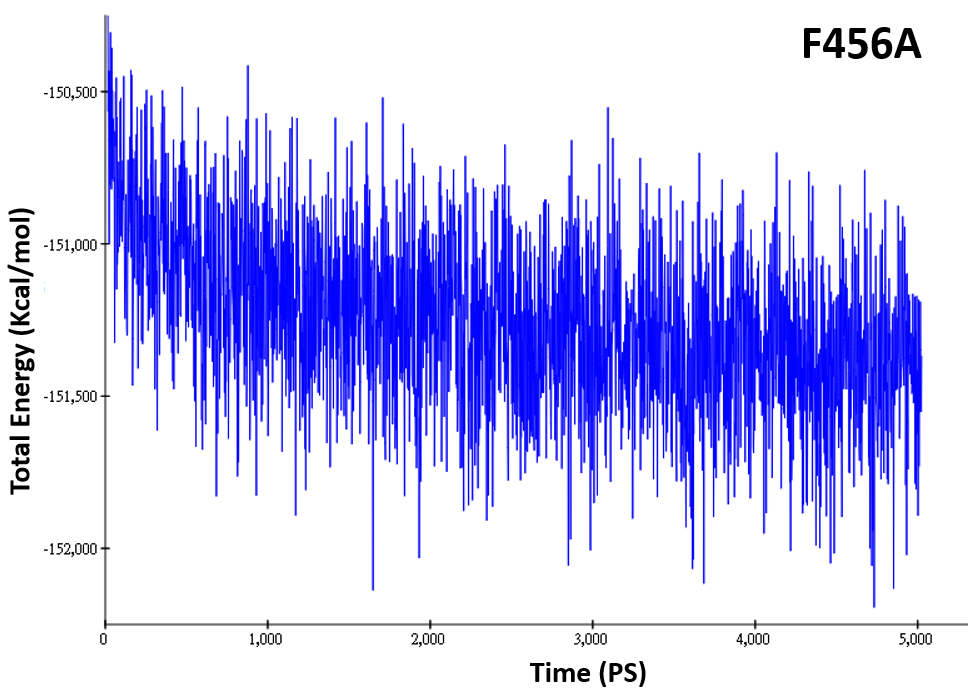

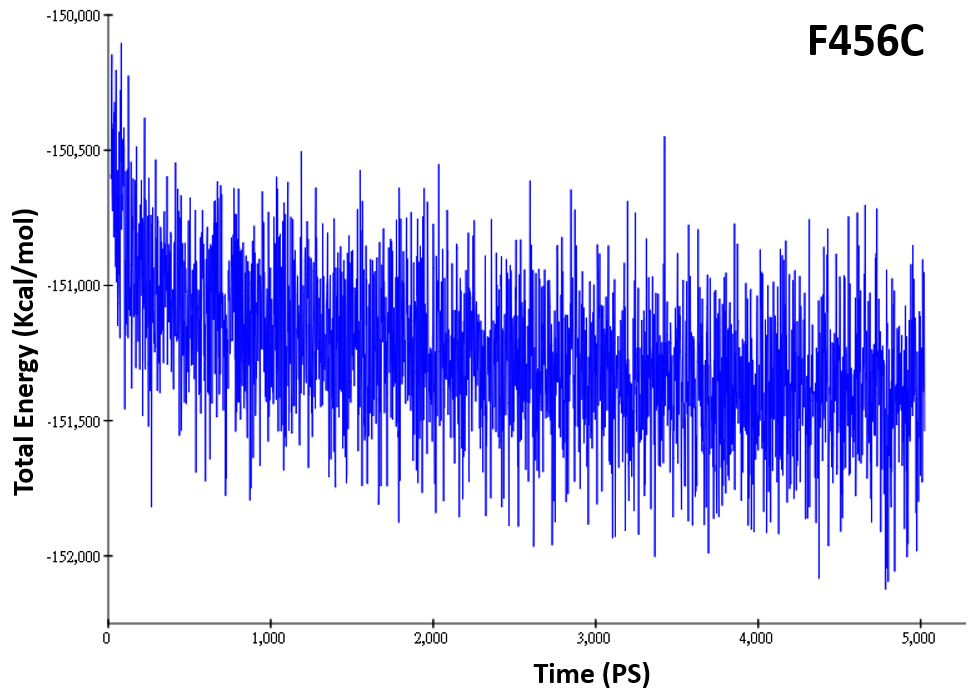

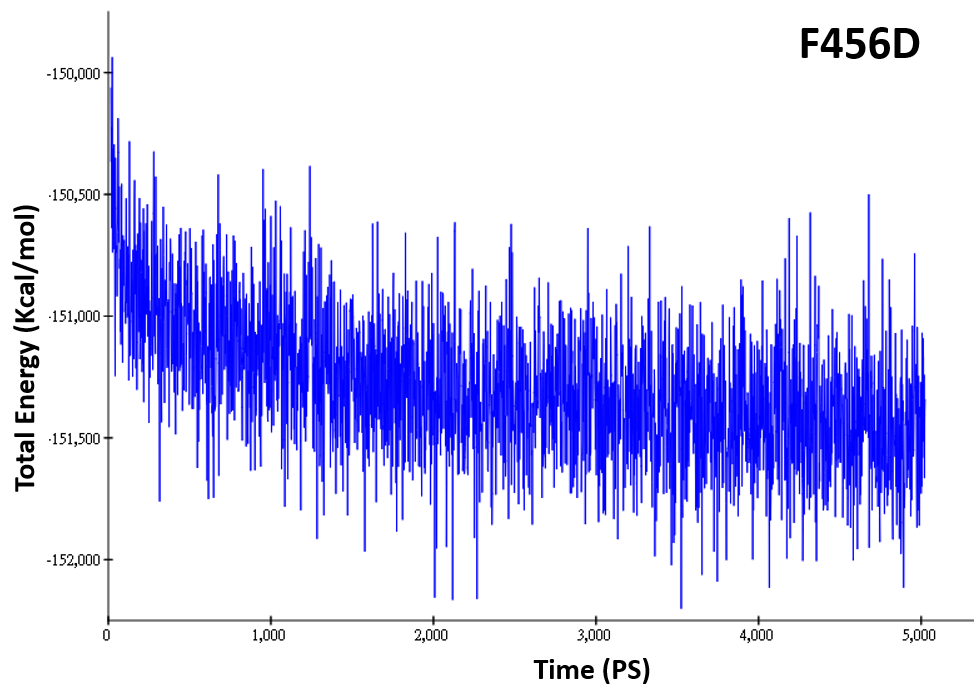


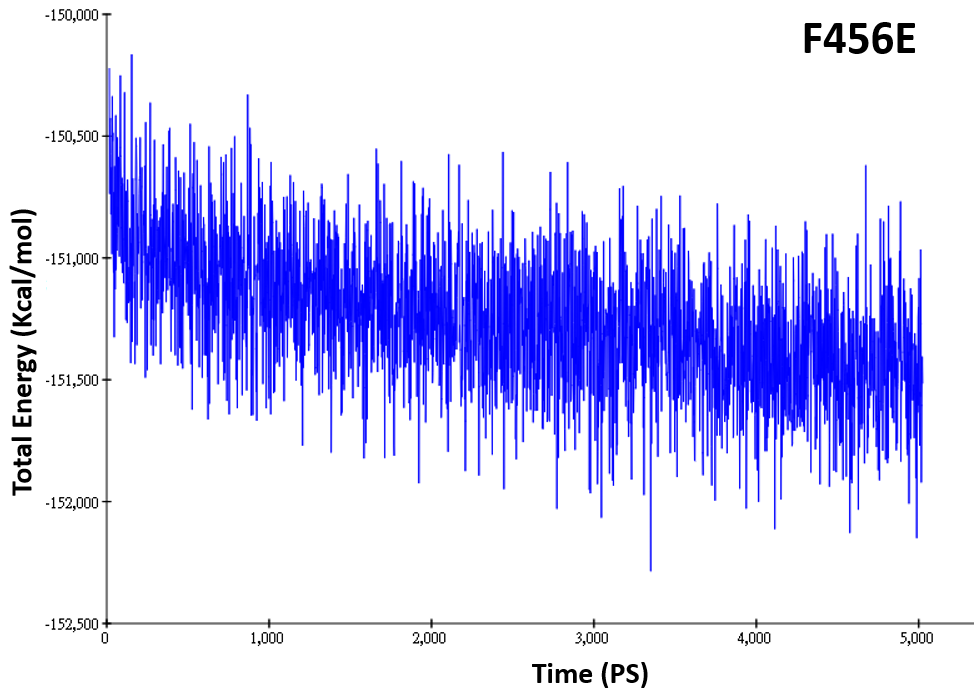

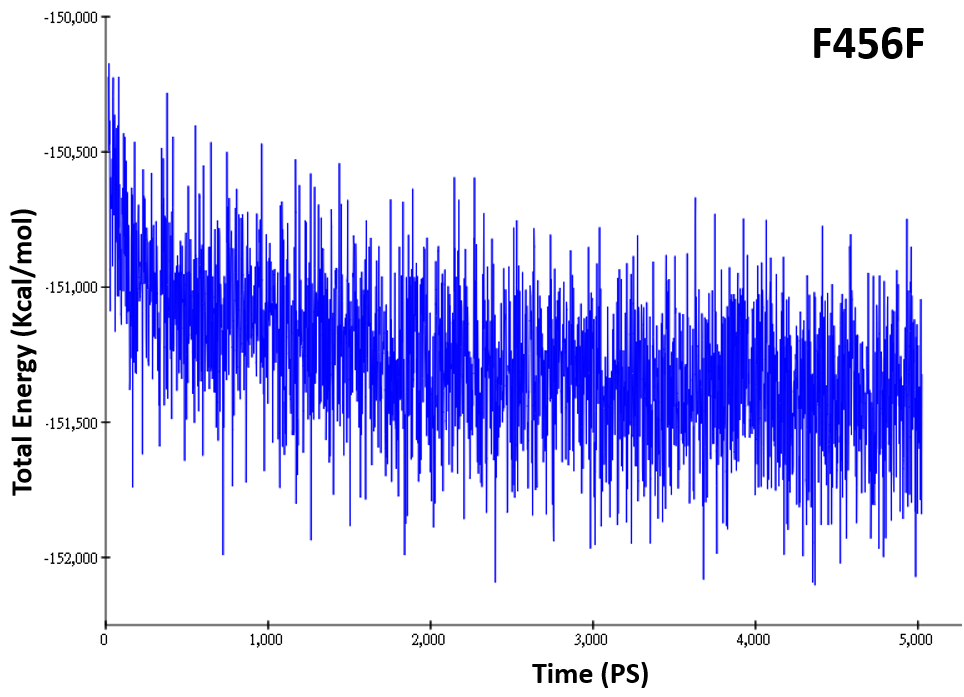

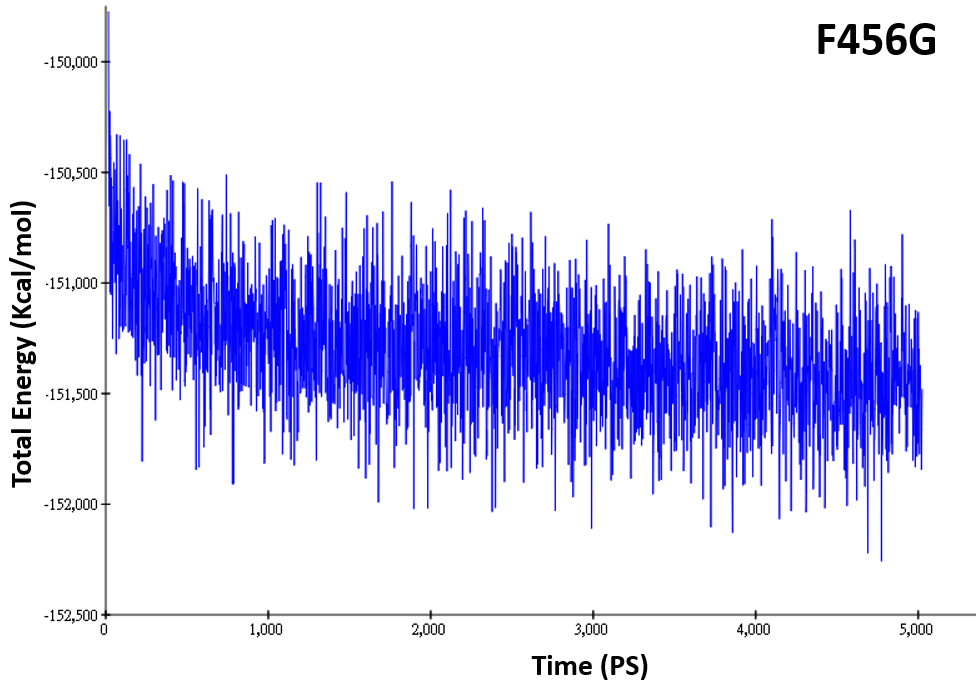


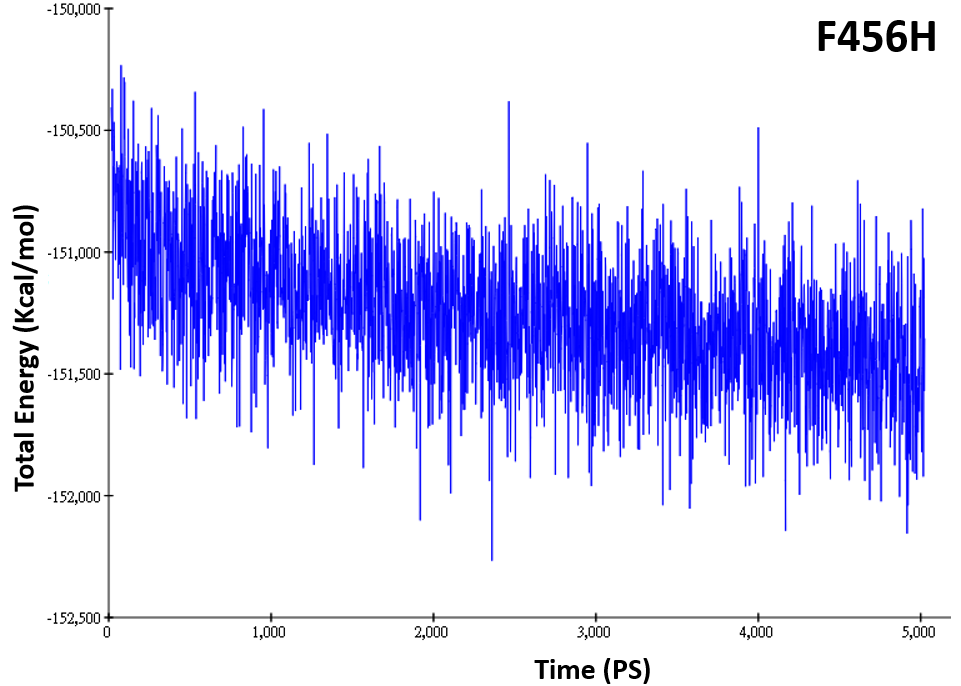

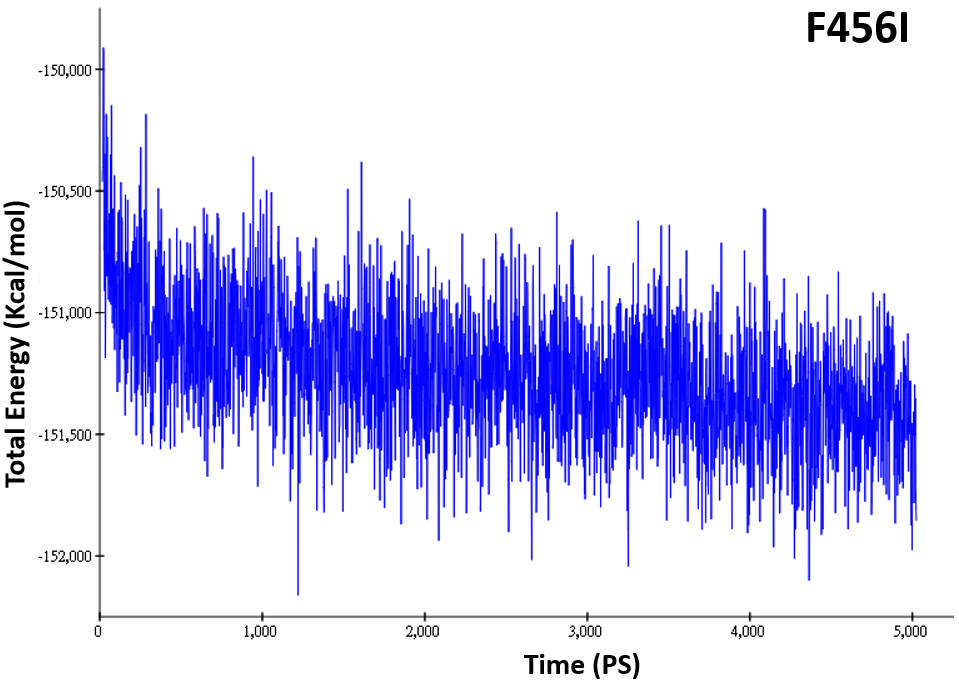

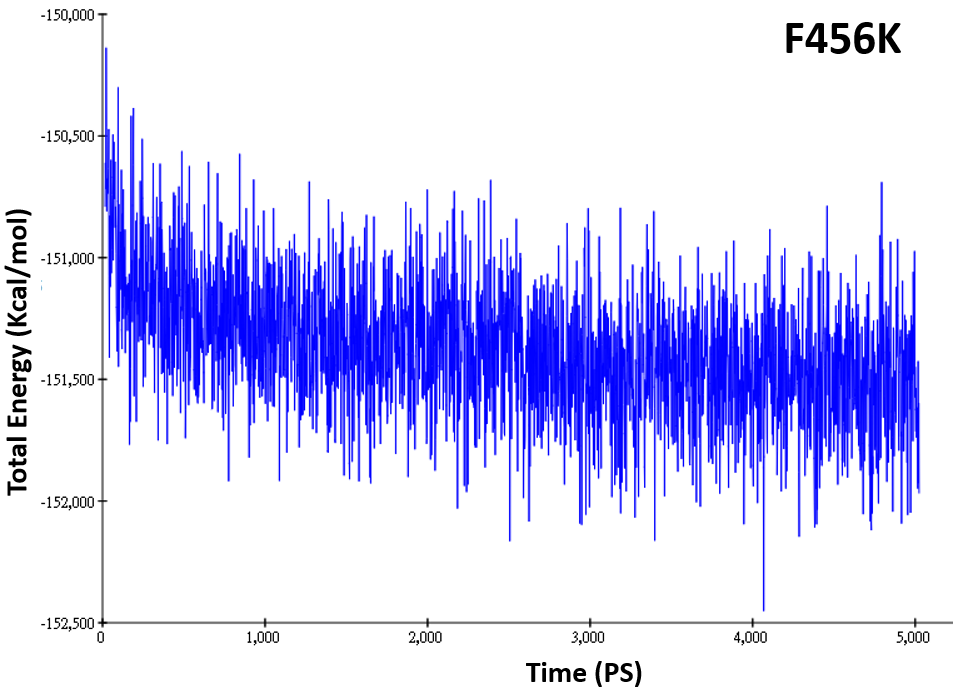


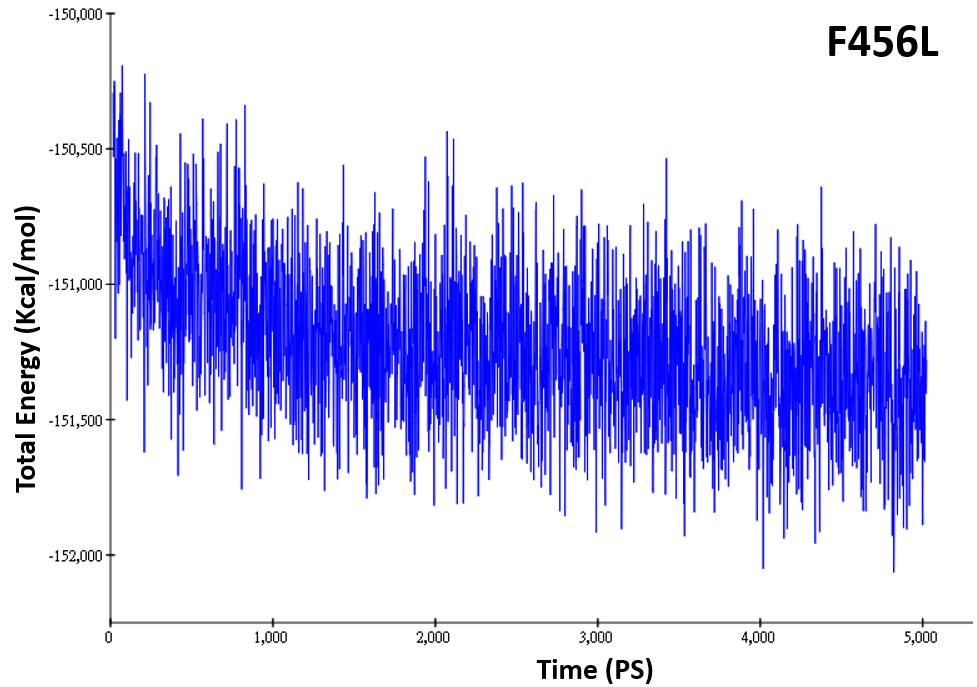


**Figure S3. The molecular dynamics simulations of F456 and its variants of RBD in complex with convalescent antibody CT-P59 (PDB ID: 7CM4).** The total energies as functions of MD simulation time for 5 ns are shown.


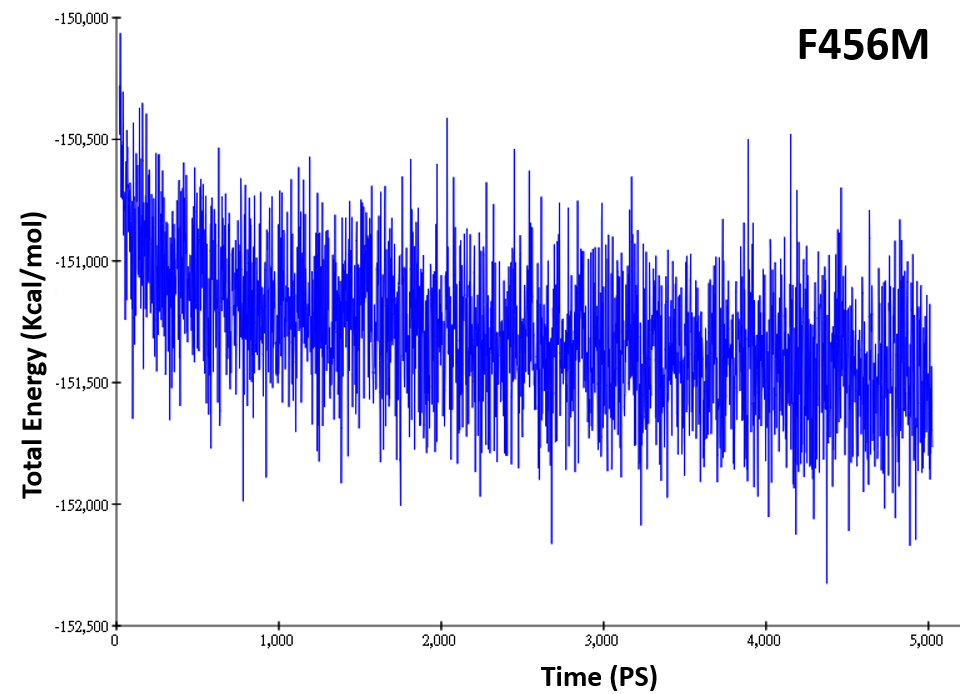

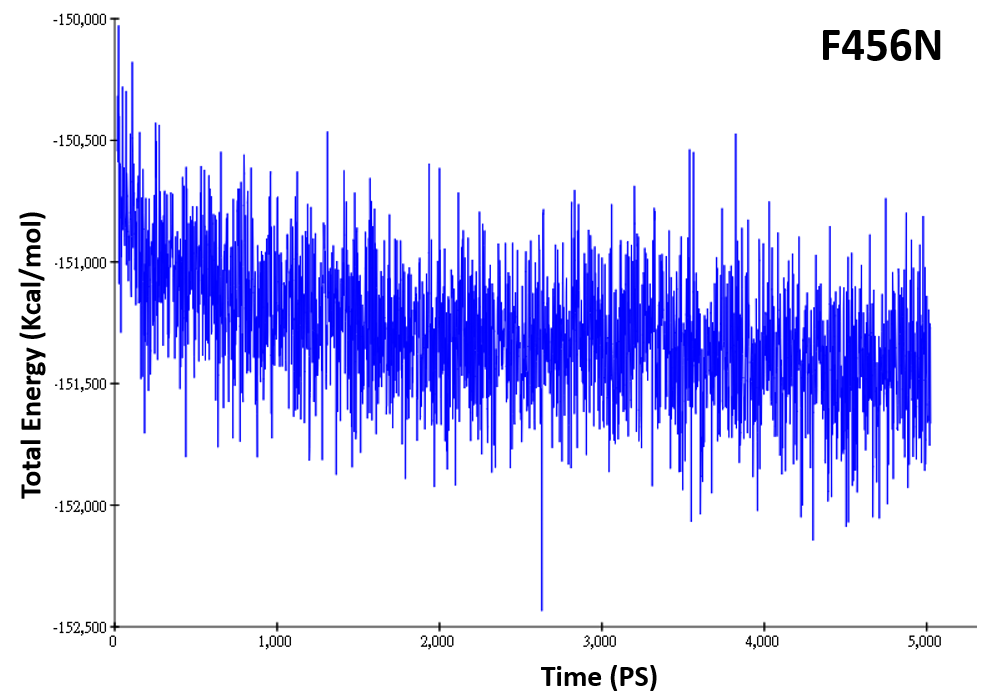

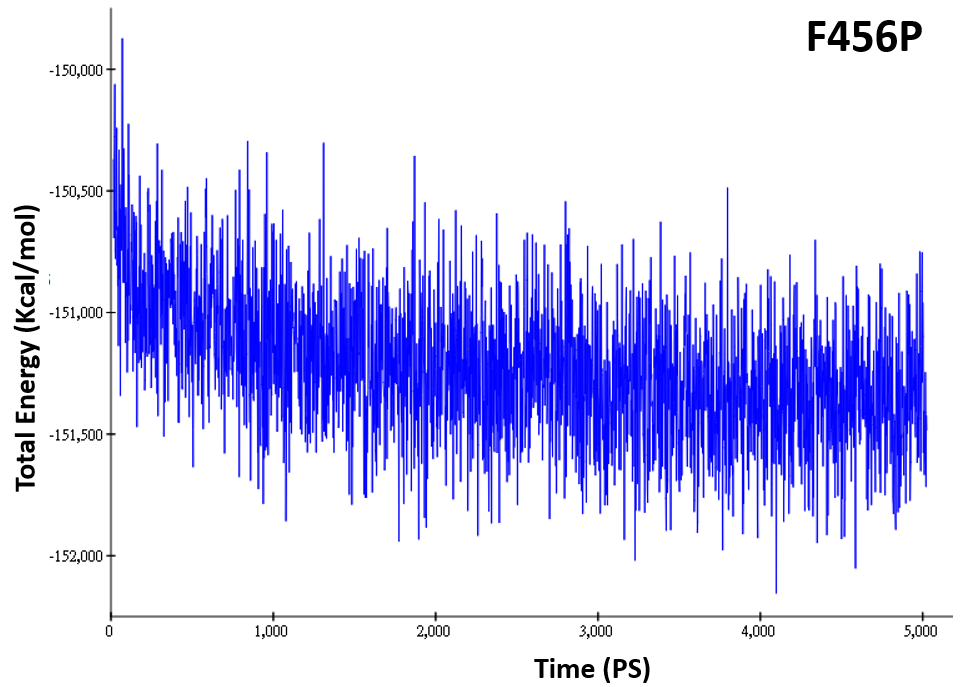


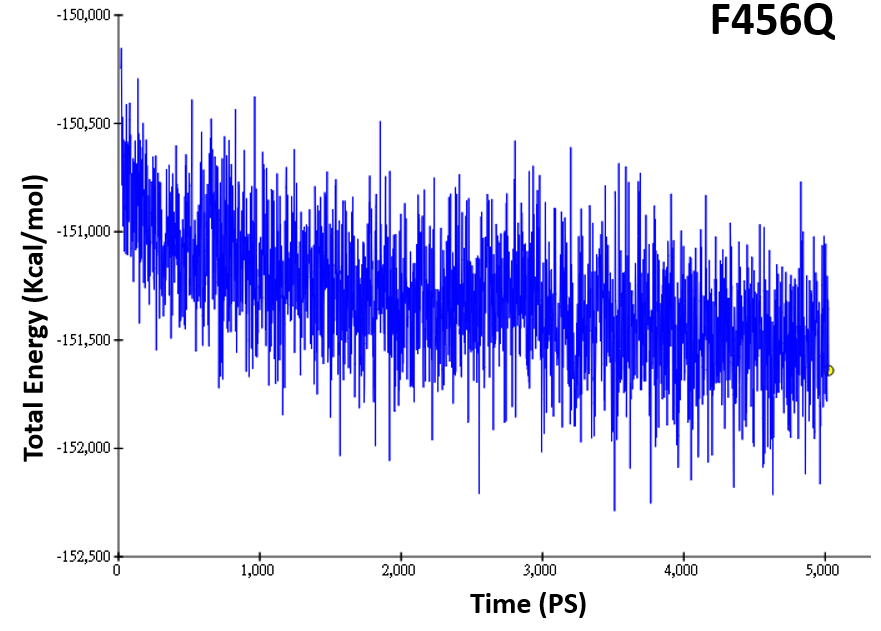

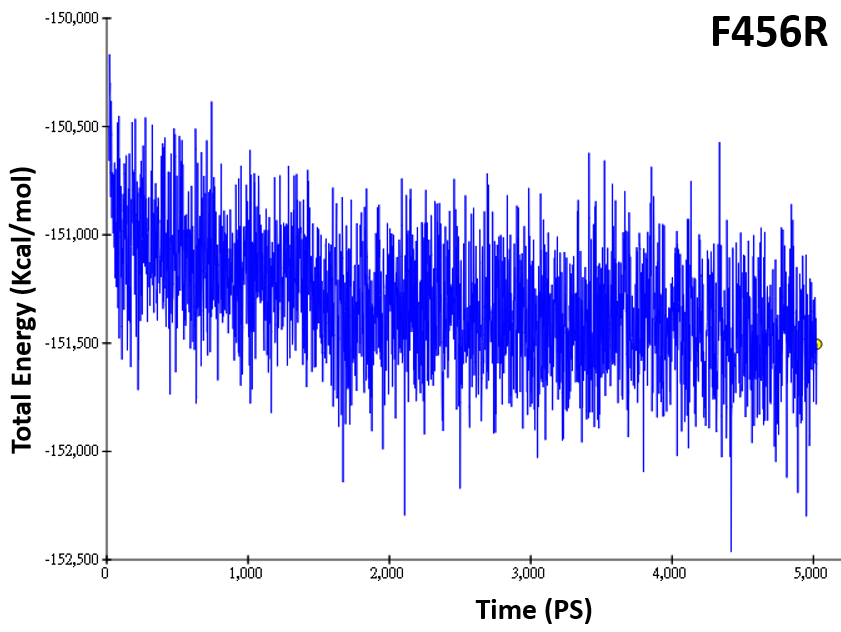

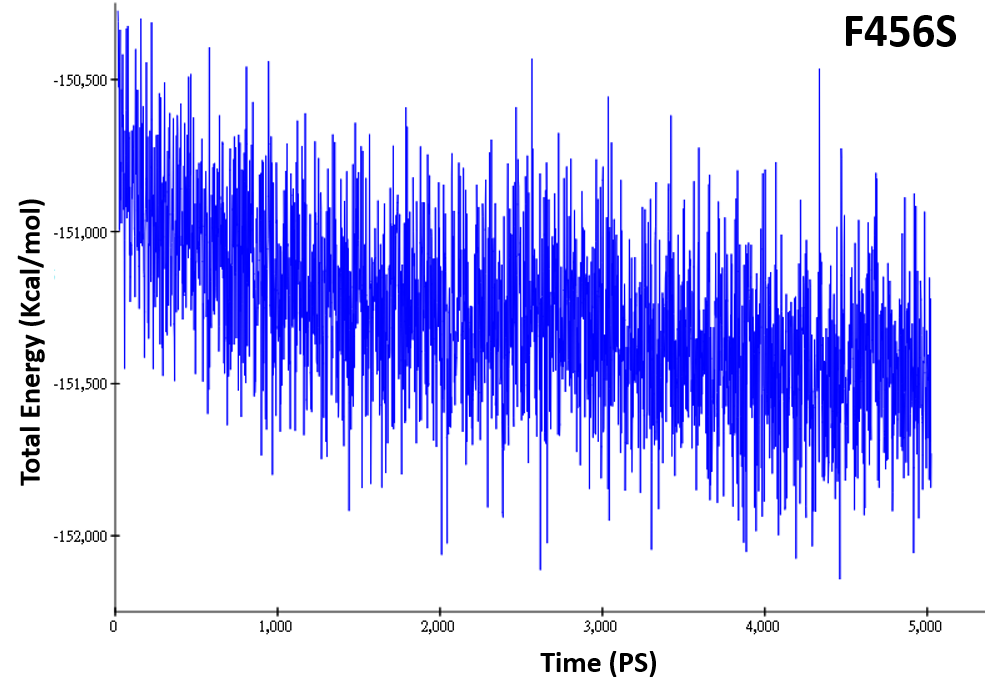


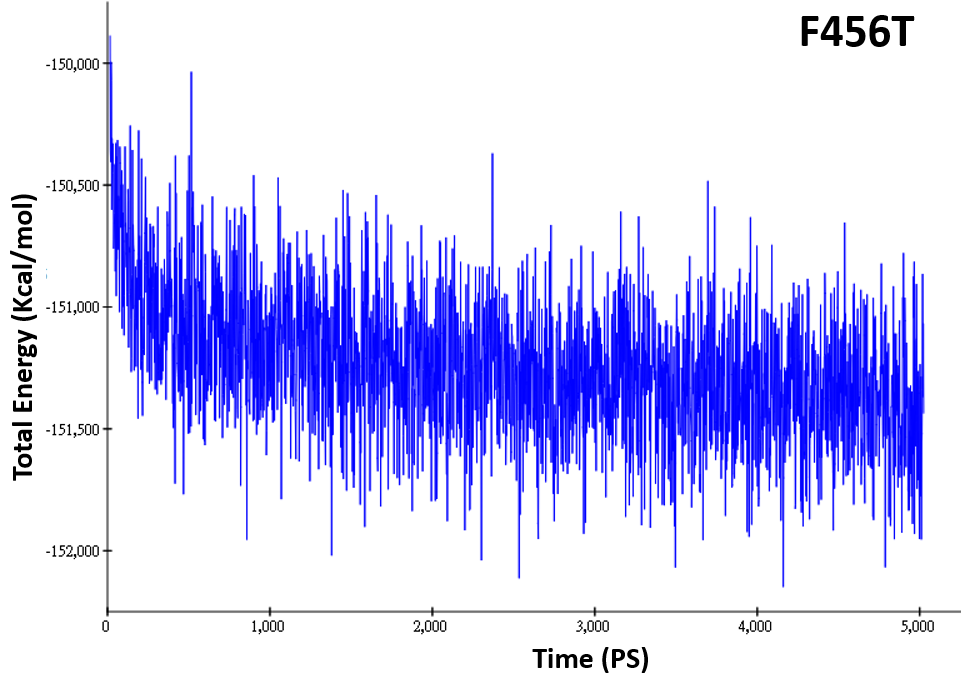

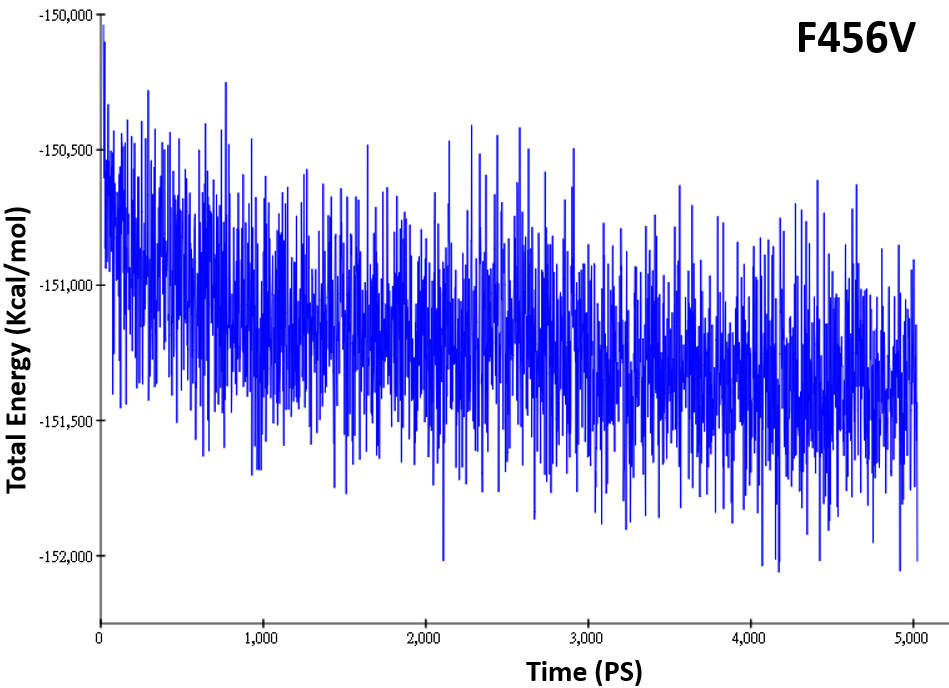

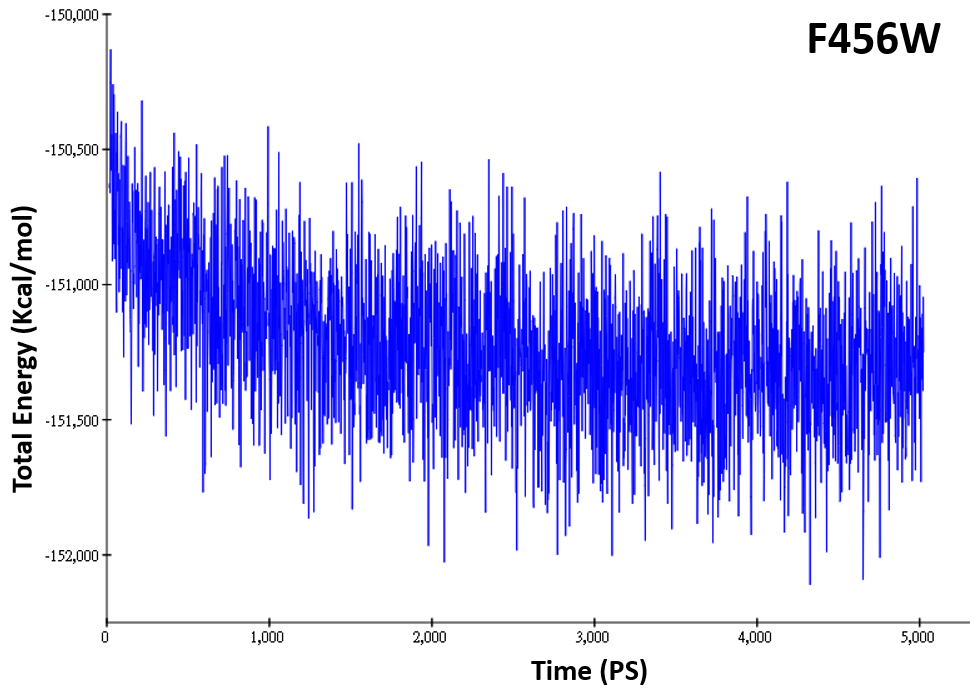


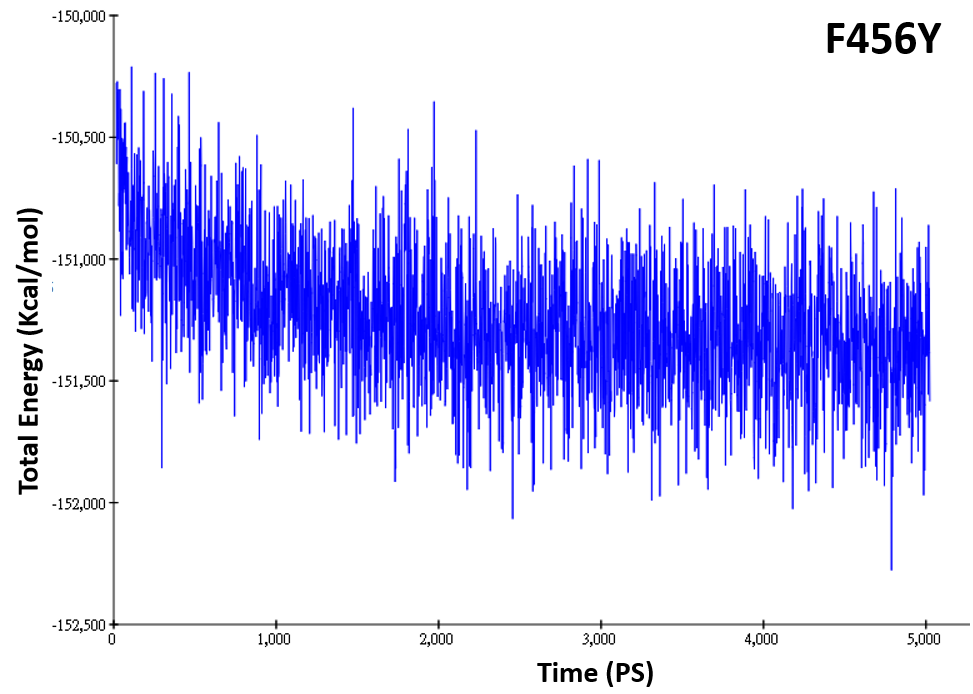


**Figure S4. The molecular dynamics simulations of F456 variants of RBD targeting convalescent antibody CT-P59 (PDB ID: 7CM4) for 5 ns.** The total energies with respect to MD simulation time for 5 ns are presented.


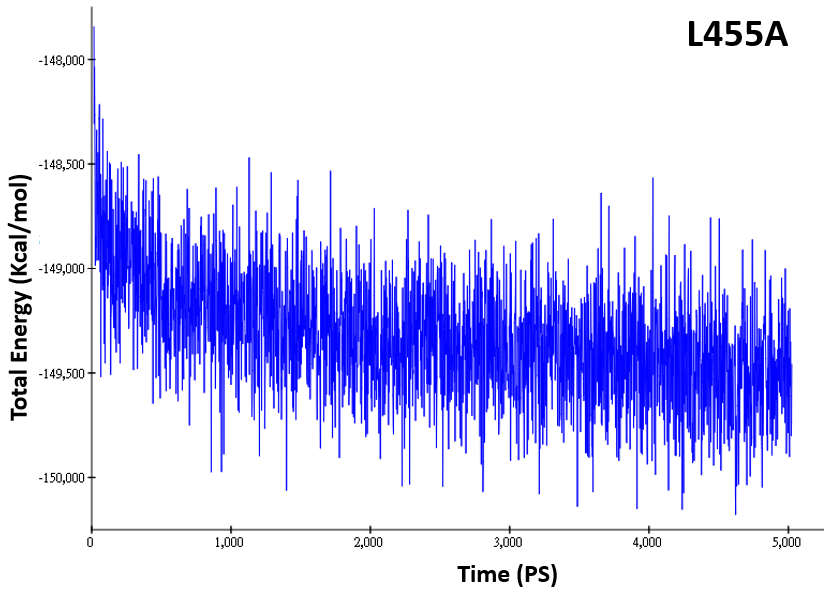

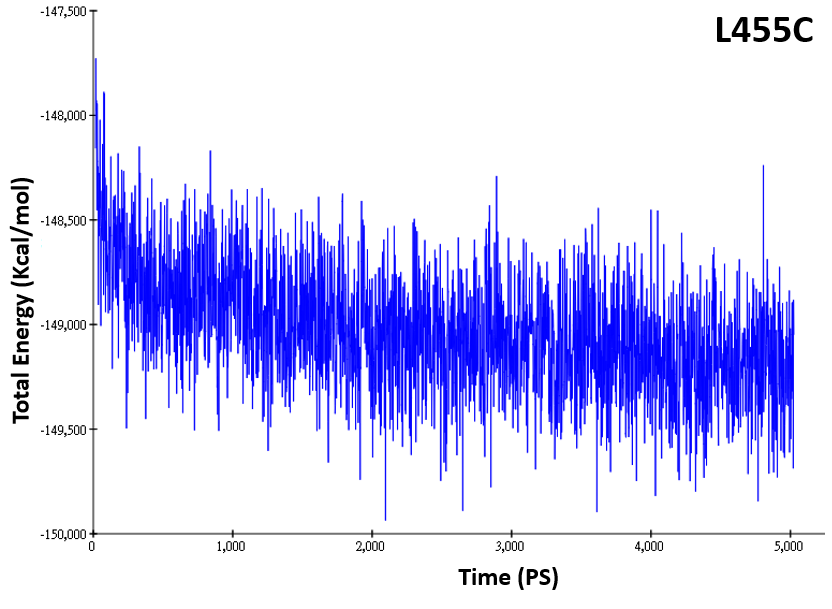

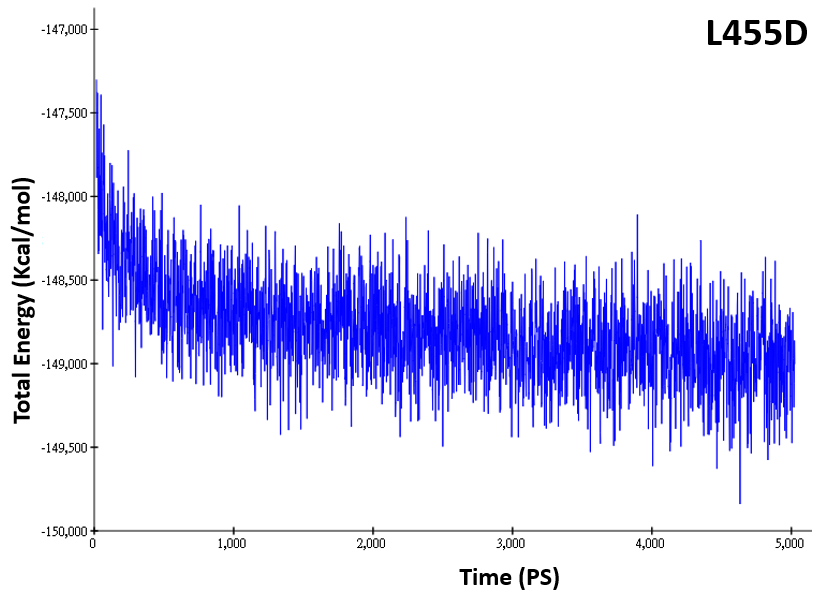

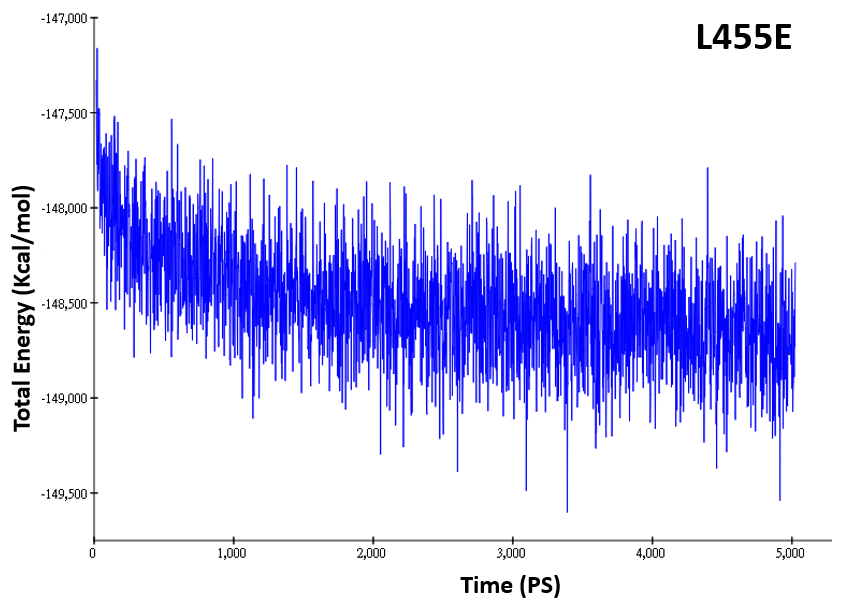

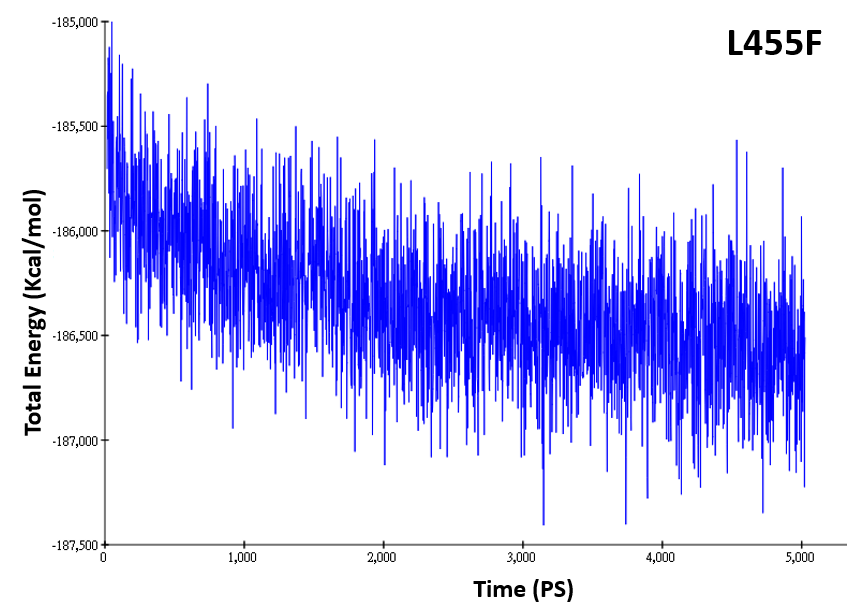

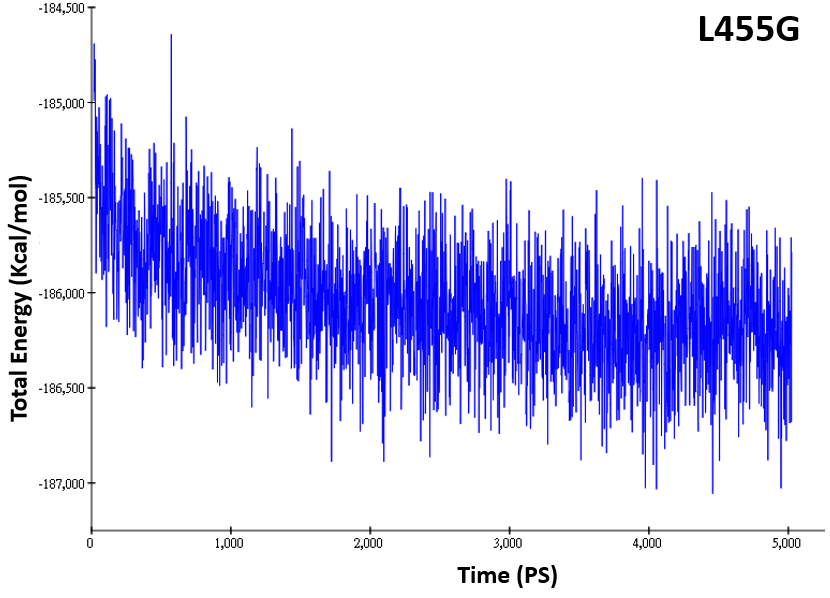

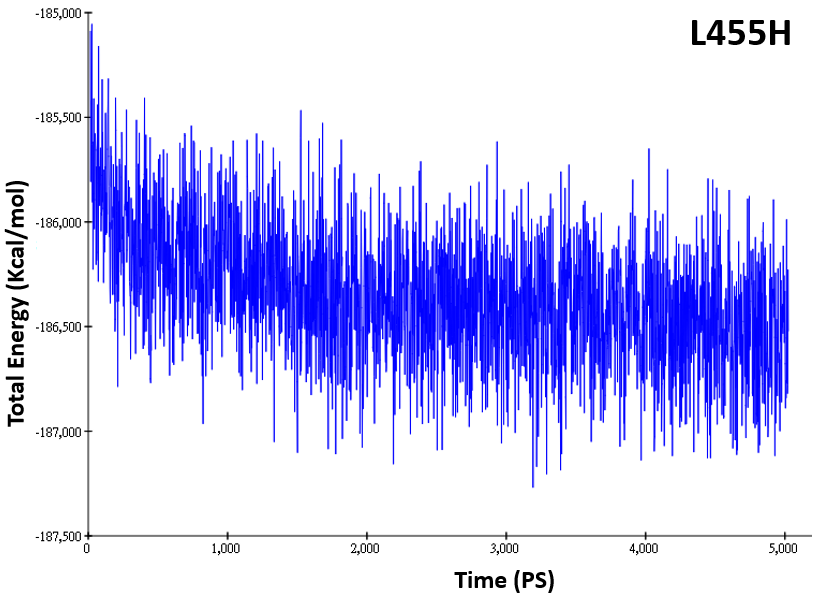

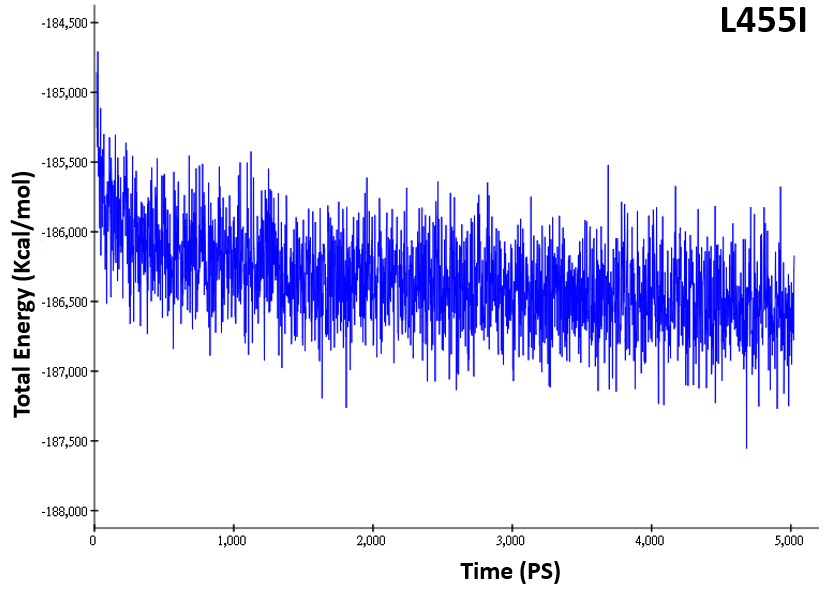

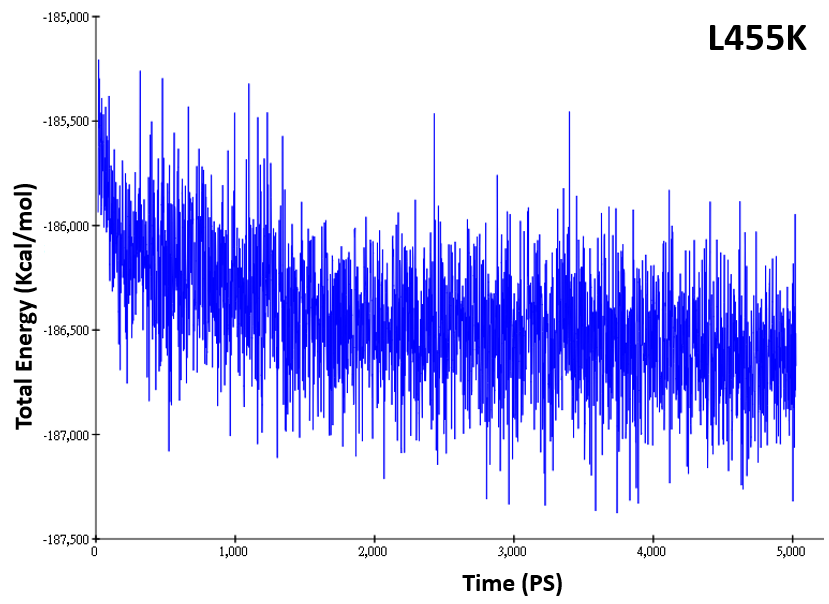

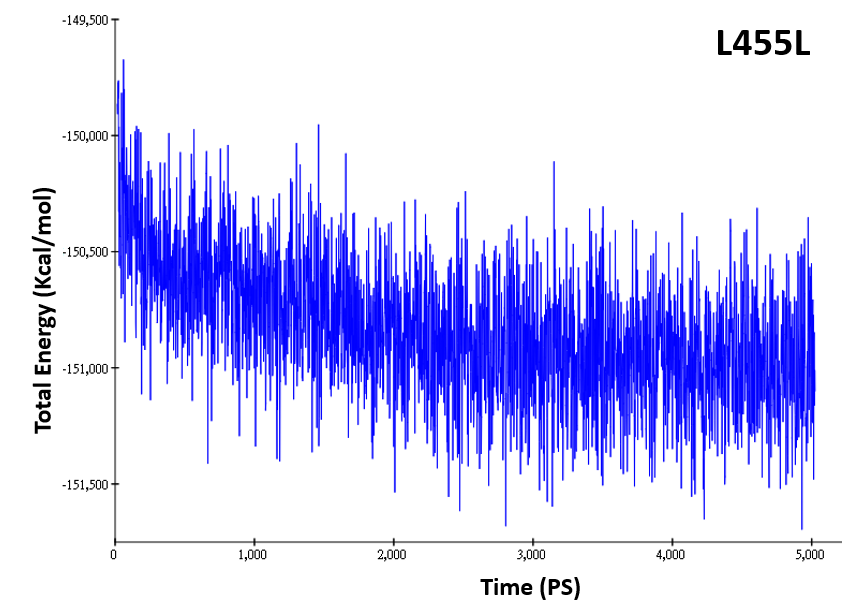


**Figure S5. The molecular dynamics simulations of L455 and its variants of RBD in complex with convalescent antibody B38 (PDB ID: 7BZ5).** The total energy as a function of simulation time for 5 ns is shown.


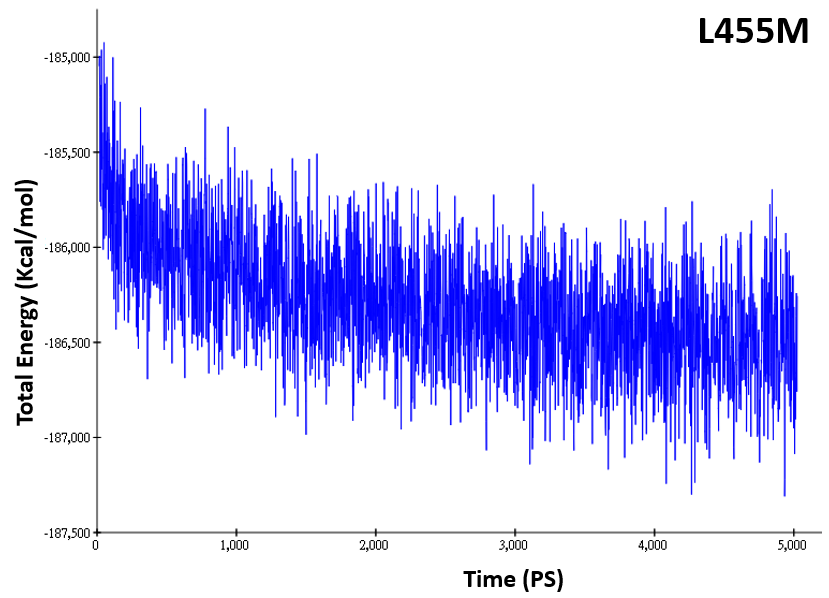

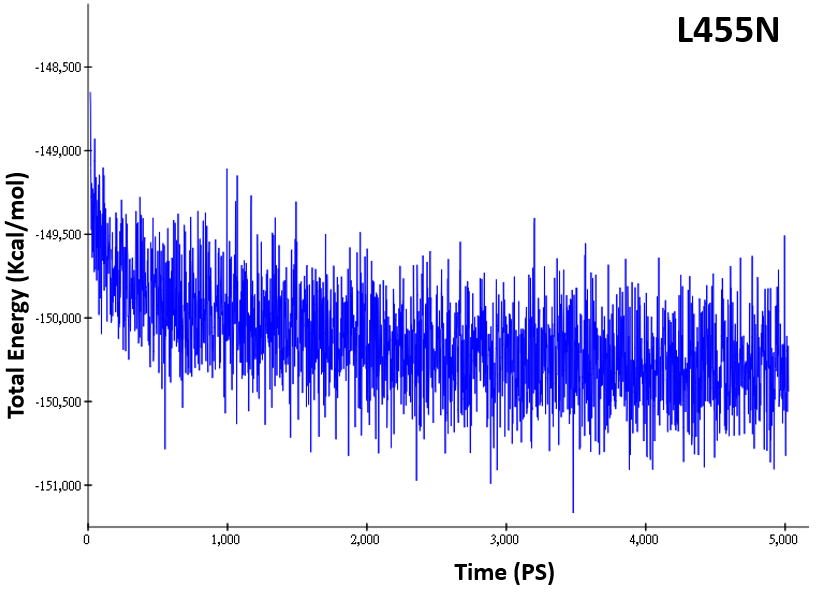

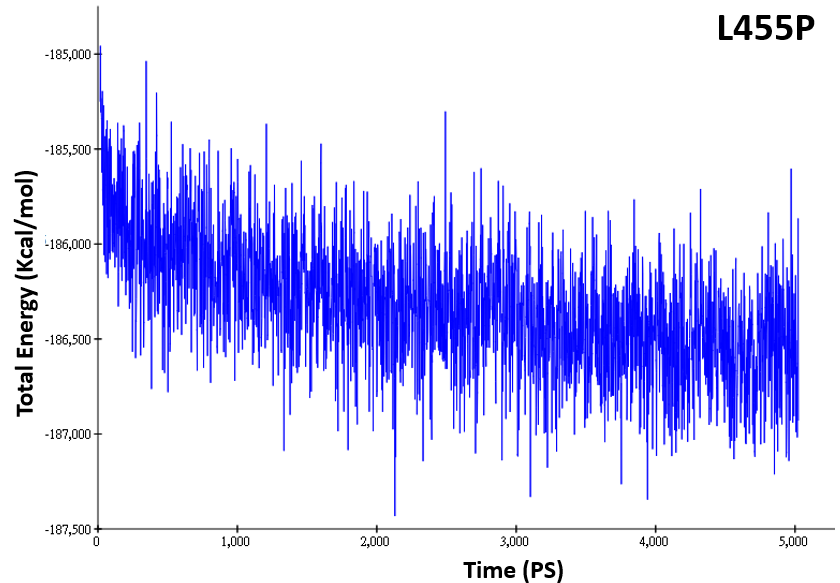


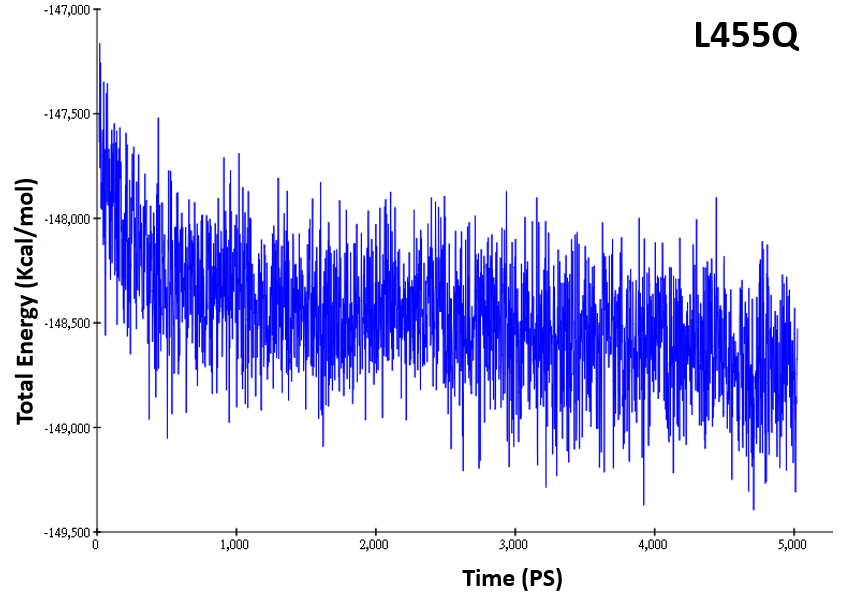

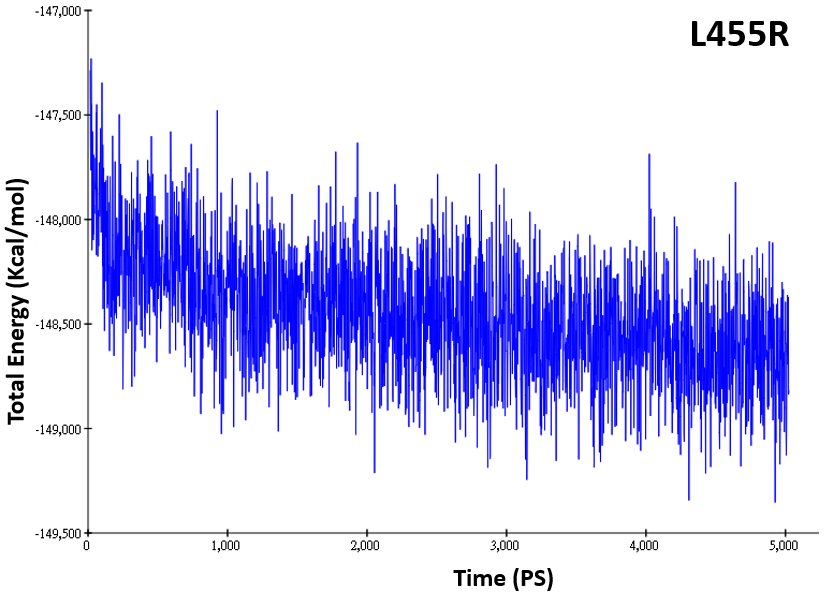

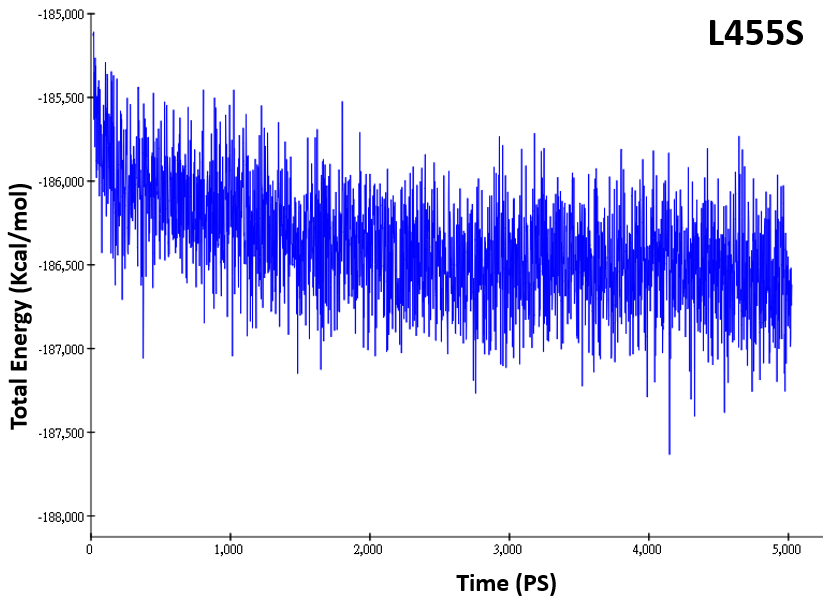

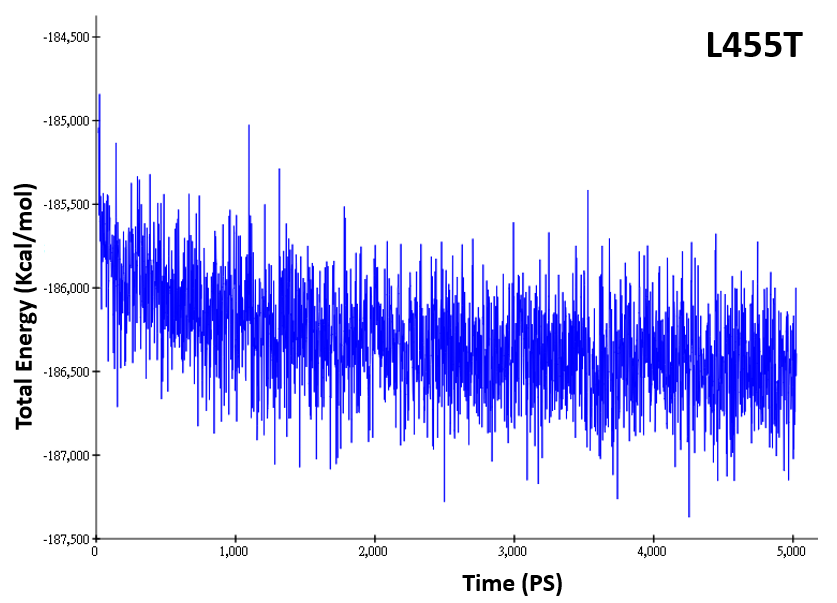

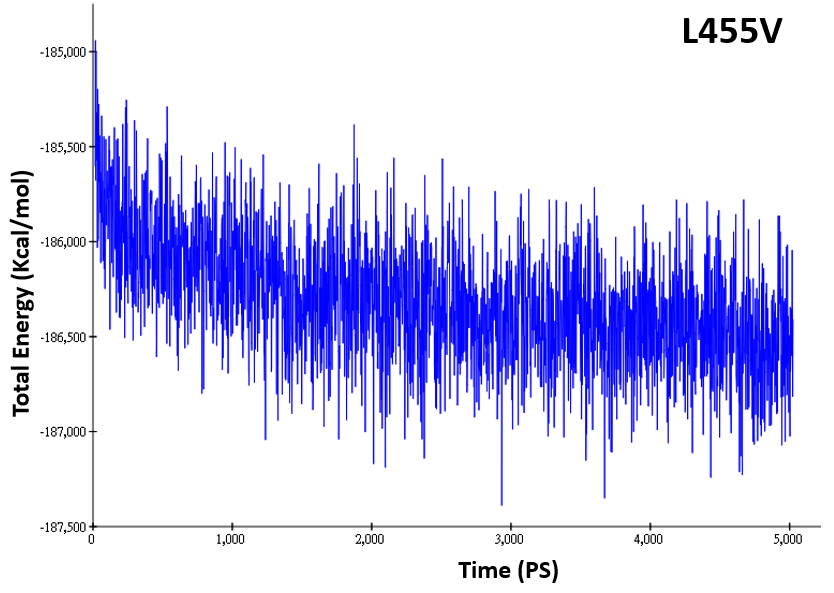

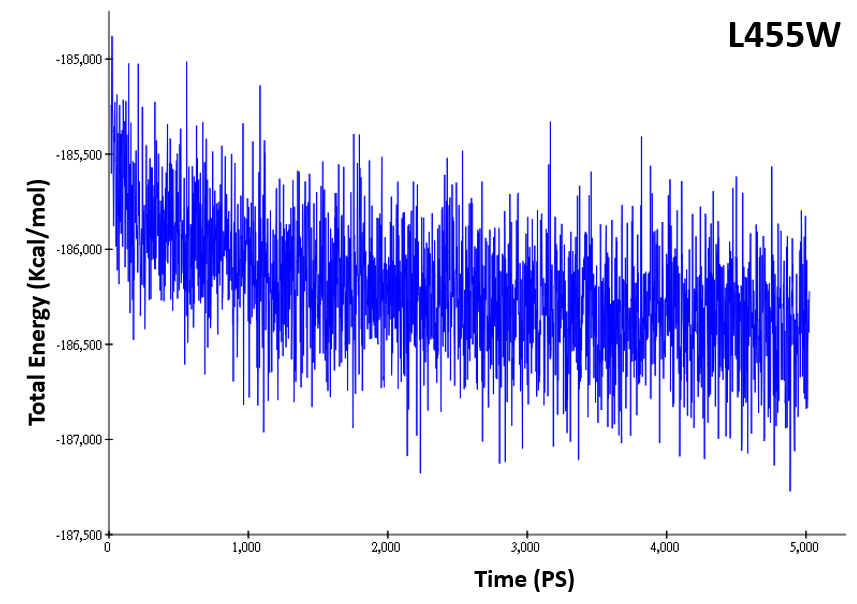


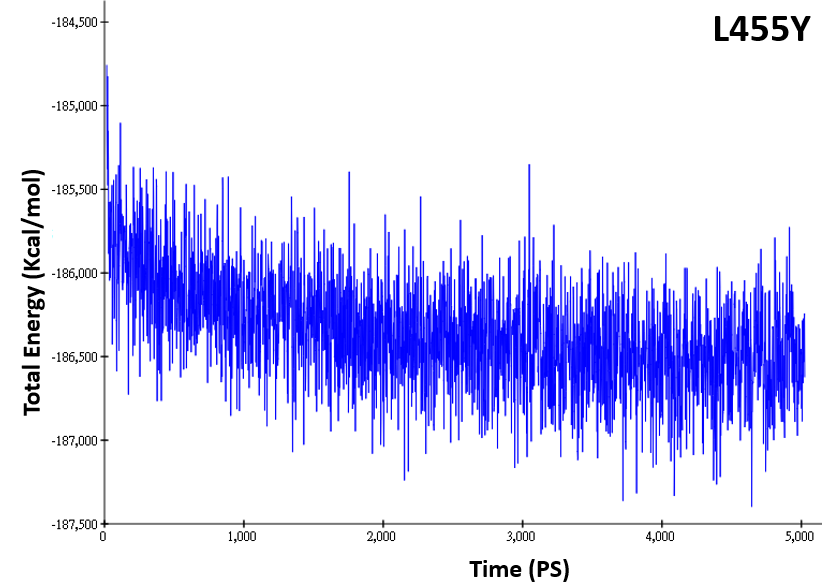


**Figure S6. The molecular dynamics simulations of L455 variants of RBD targeting convalescent antibody B38 (PDB ID: 7BZ5).** The trajectory profiles of total energies of L455 variants in complex with B38 during 5 ns are presented.


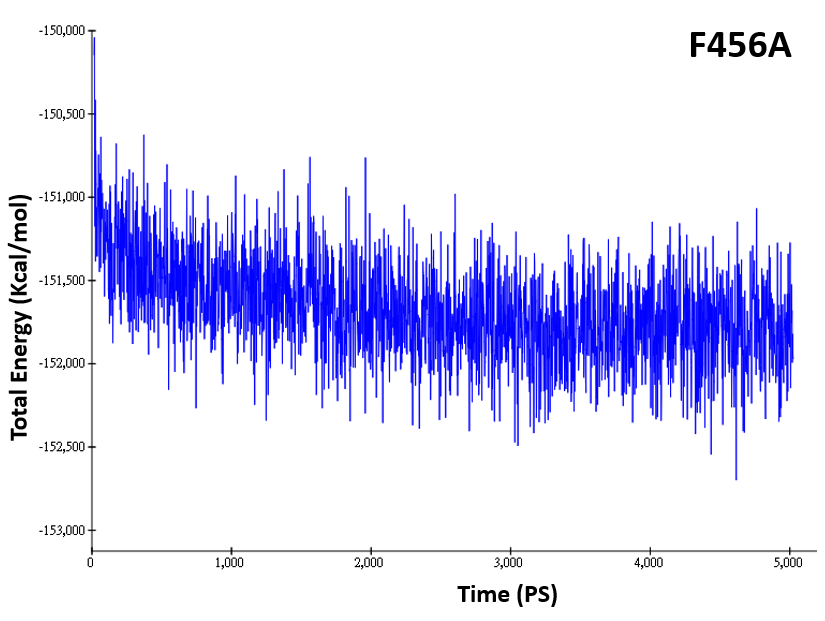

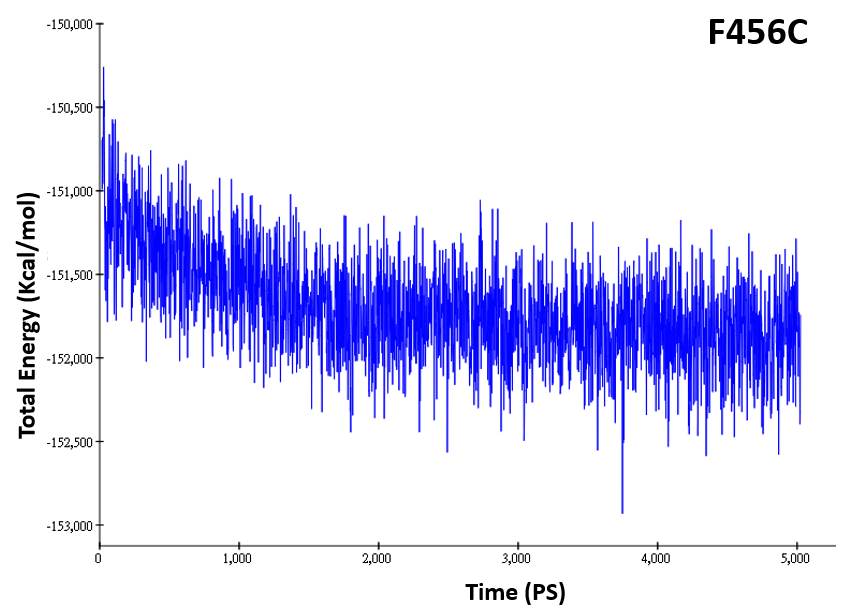

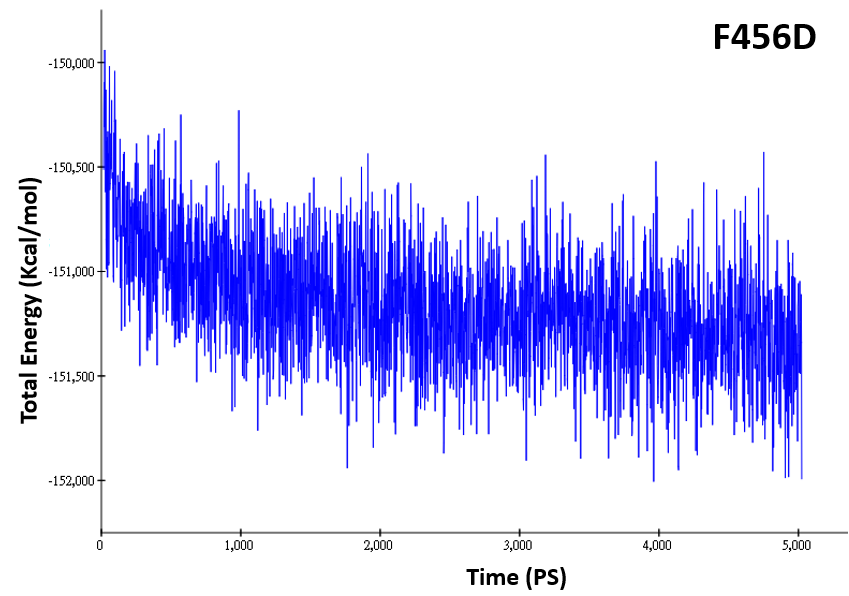


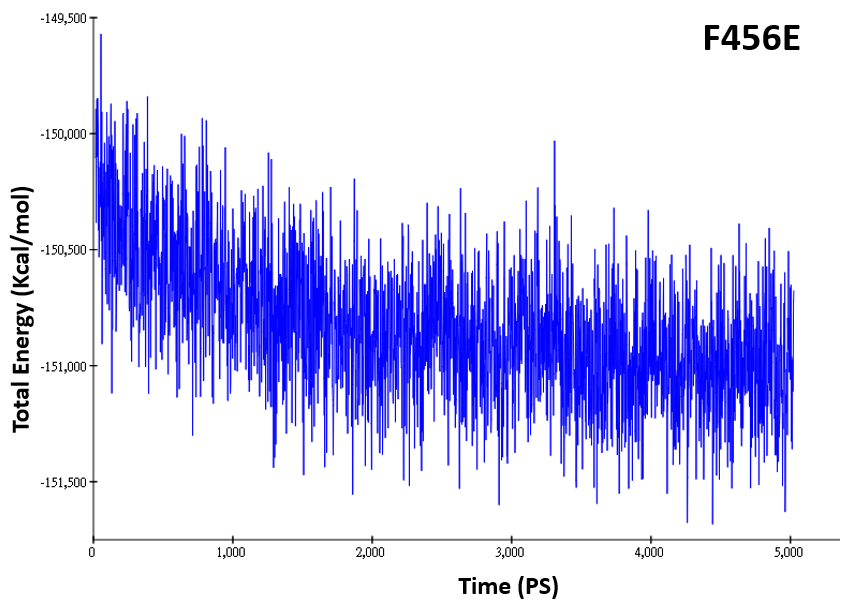

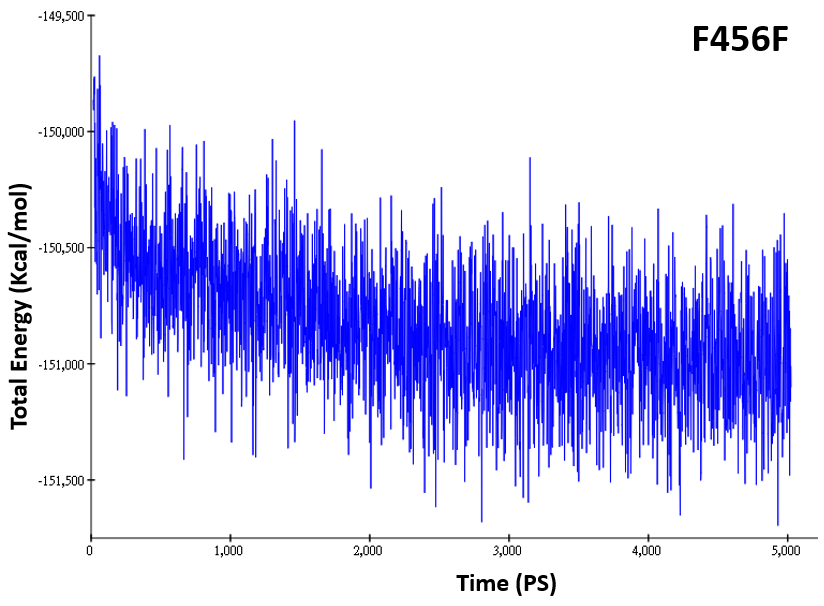

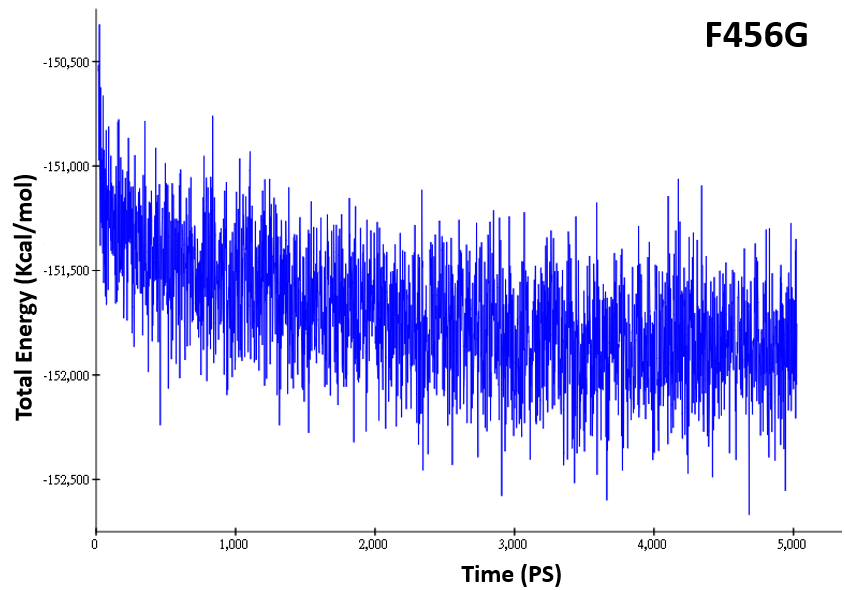


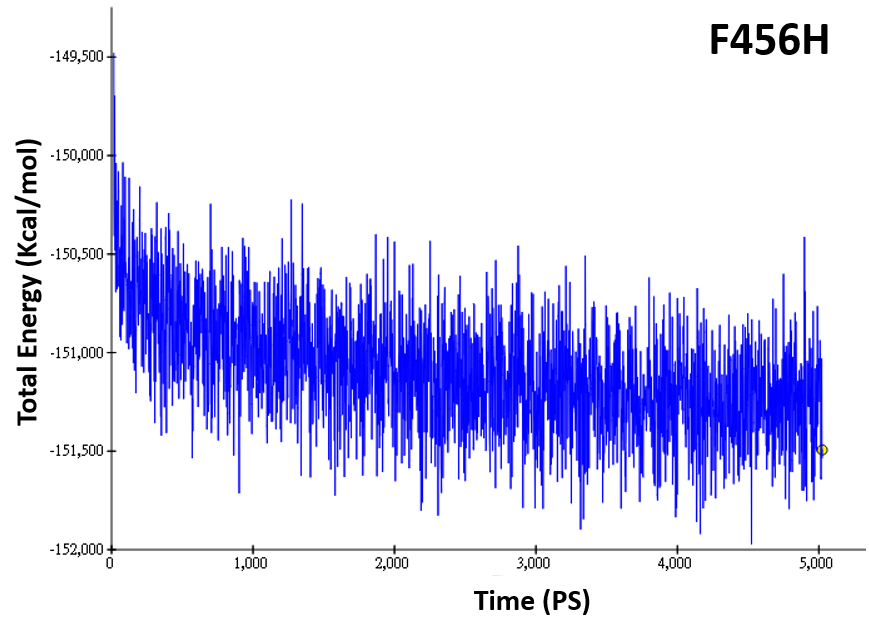

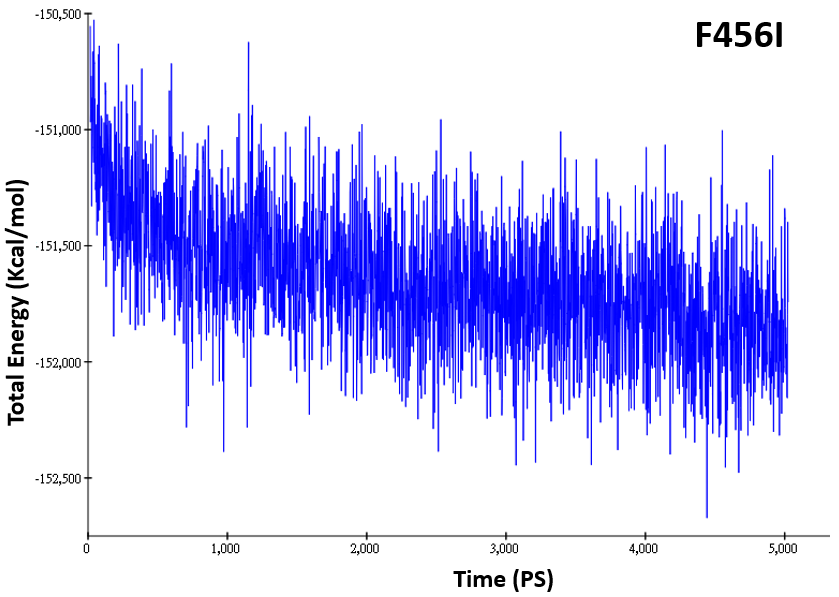

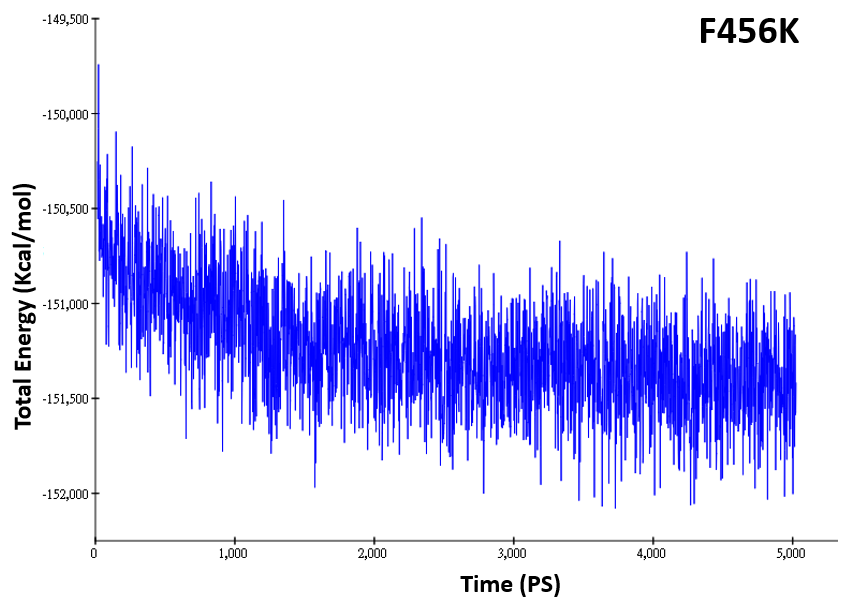

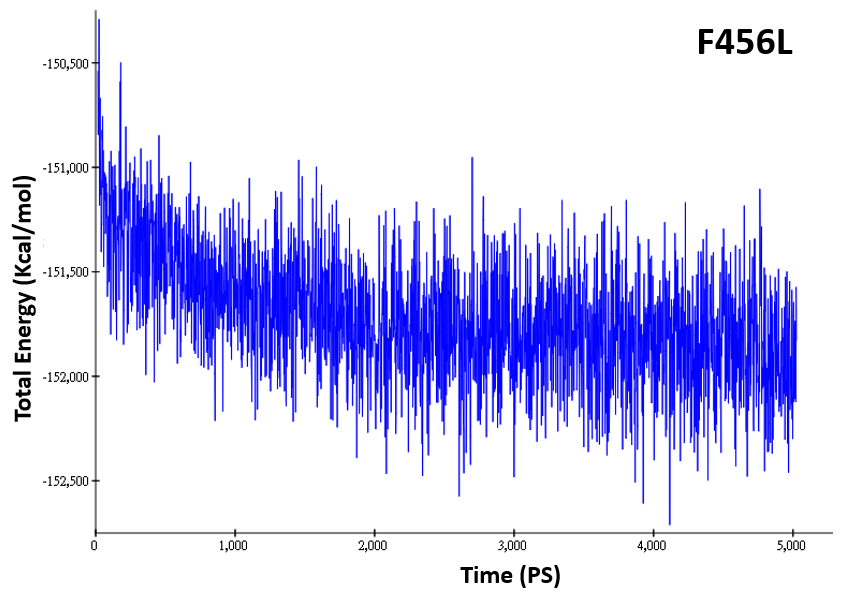


**Figure S7. The molecular dynamics simulations of F456 and its variants of RBD in complex with convalescent antibody B38 (PDB ID: 7BZ5)**. The total energies as functions of MD simulation time for 5 ns are shown.


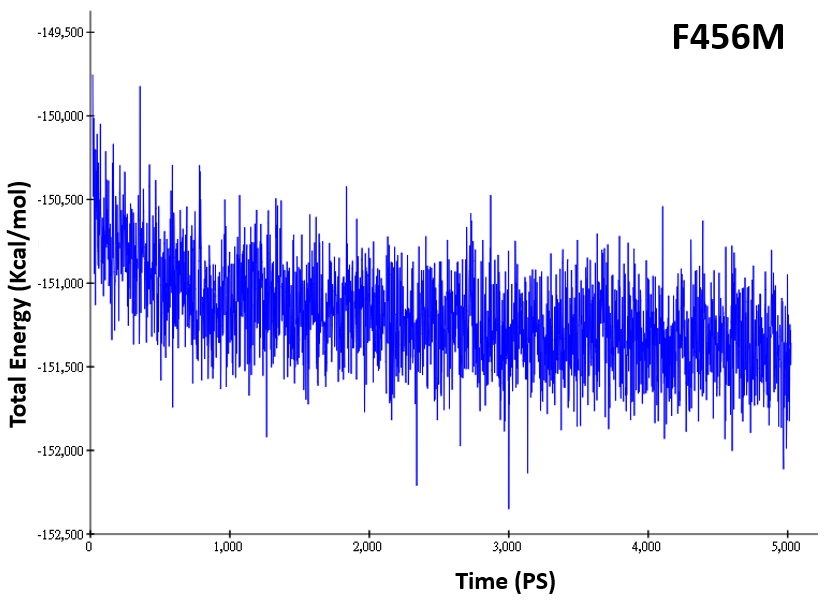

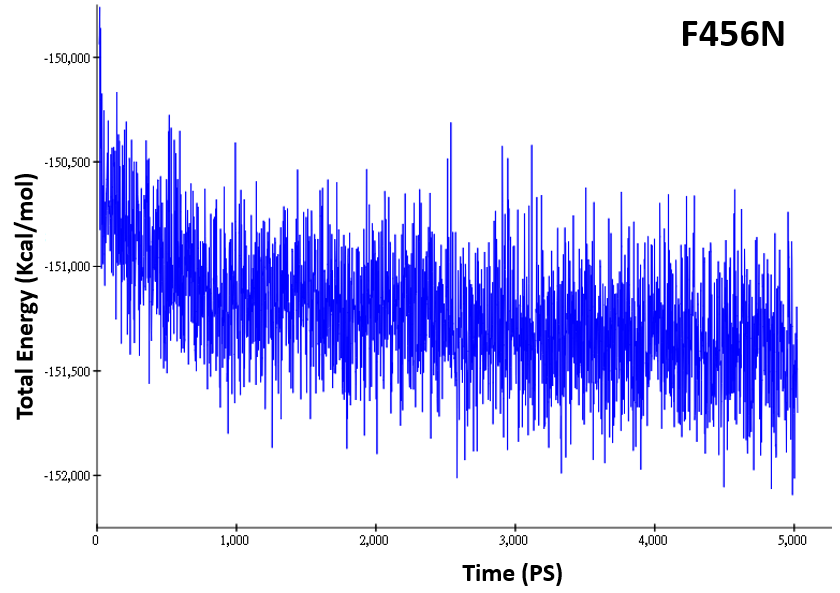

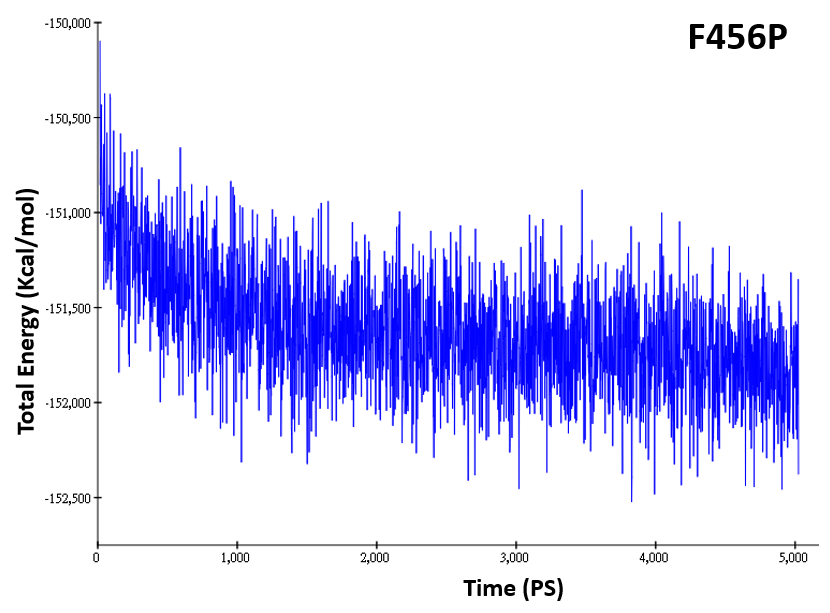


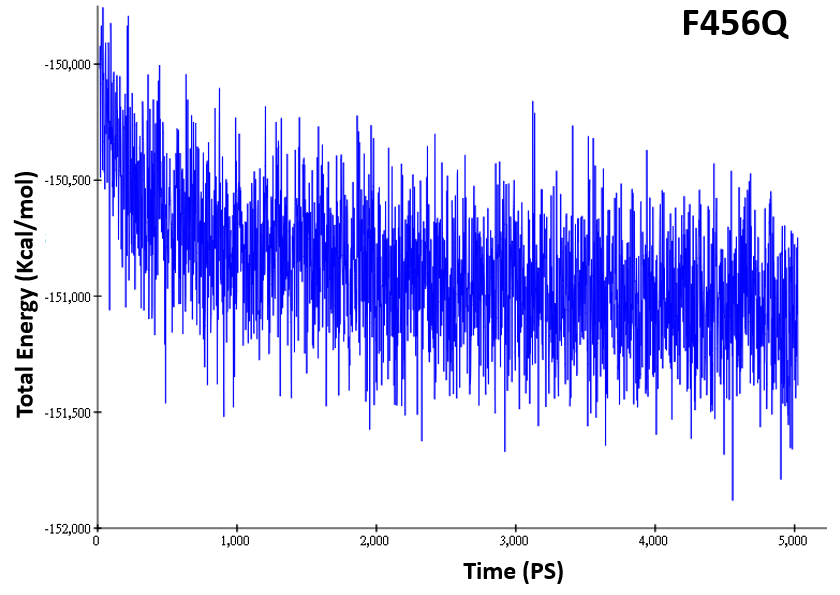

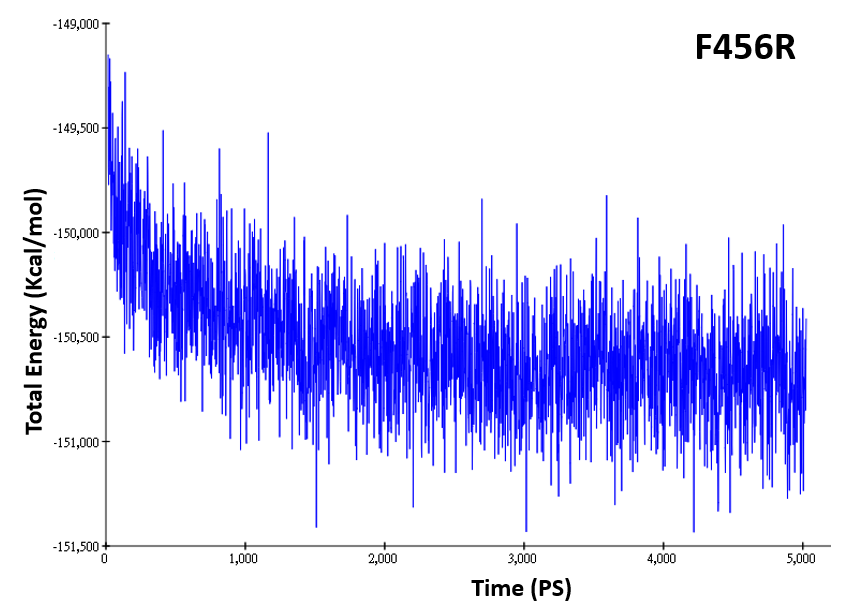

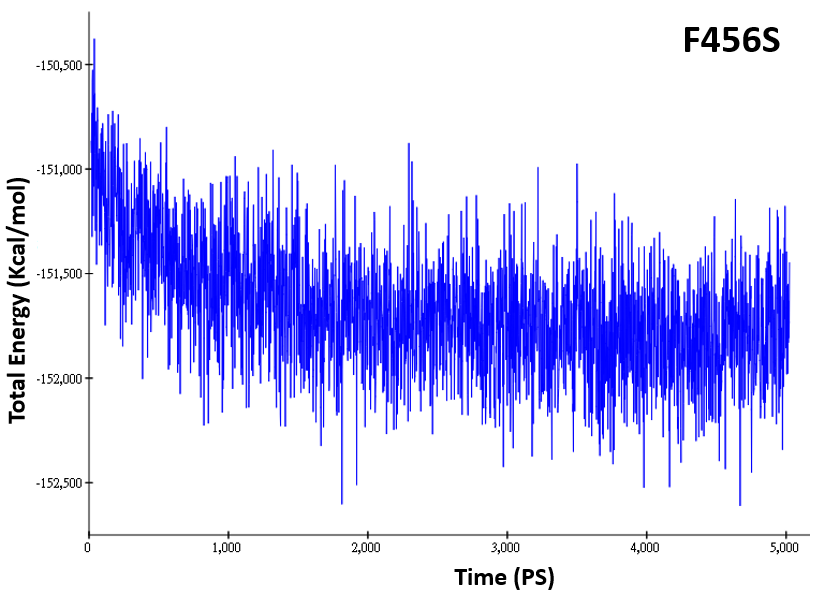


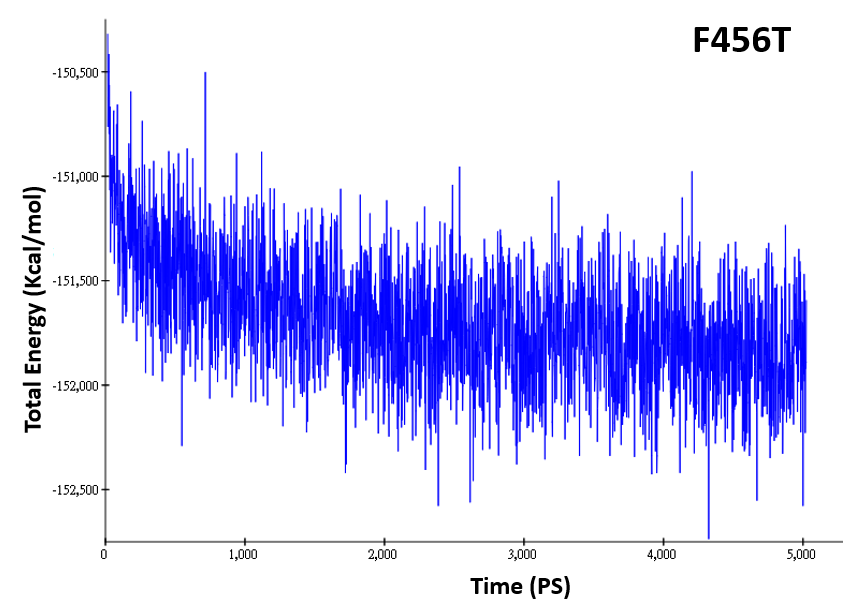

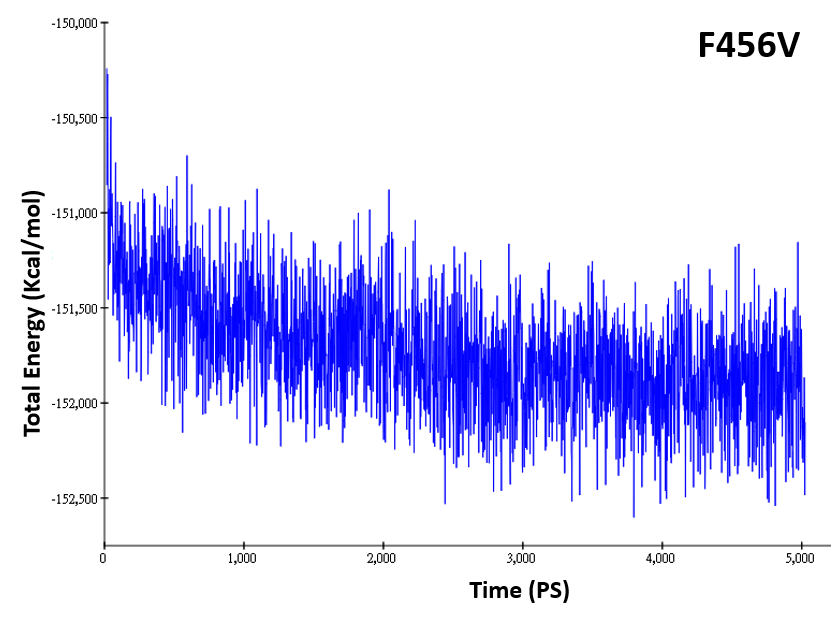

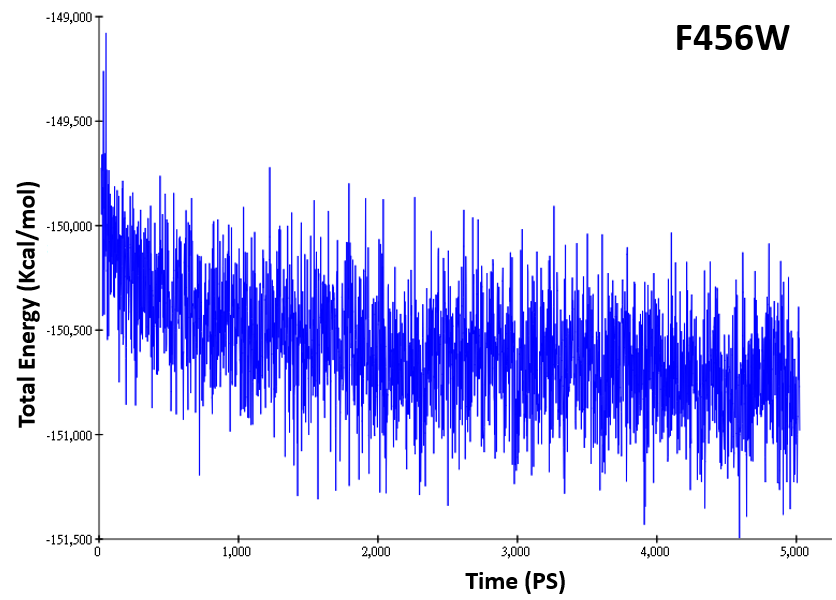


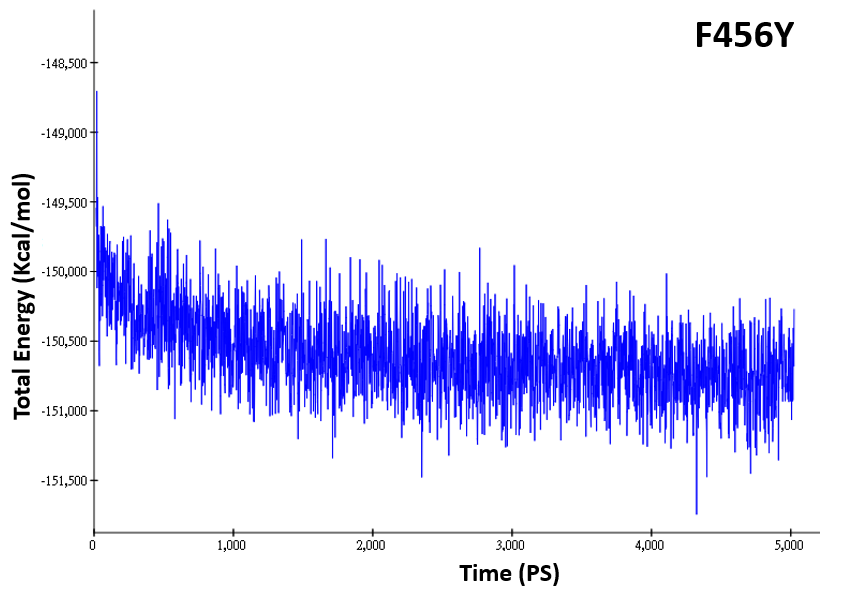


**Figure S8. The molecular dynamics simulations of F456 variants of RBD targeting convalescent antibody B38 (PDB ID: 7BZ5).** The total energies with respect to MD simulation time for 5 ns are presented.


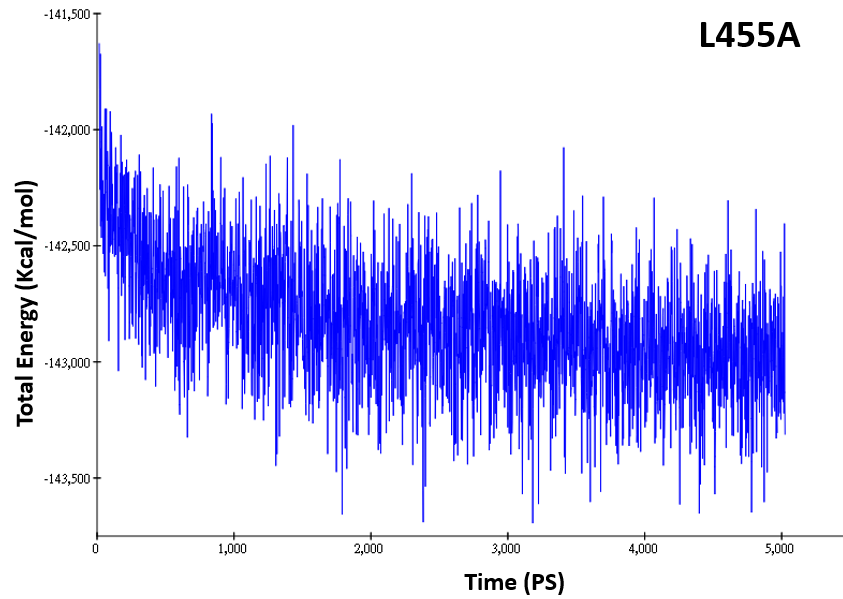

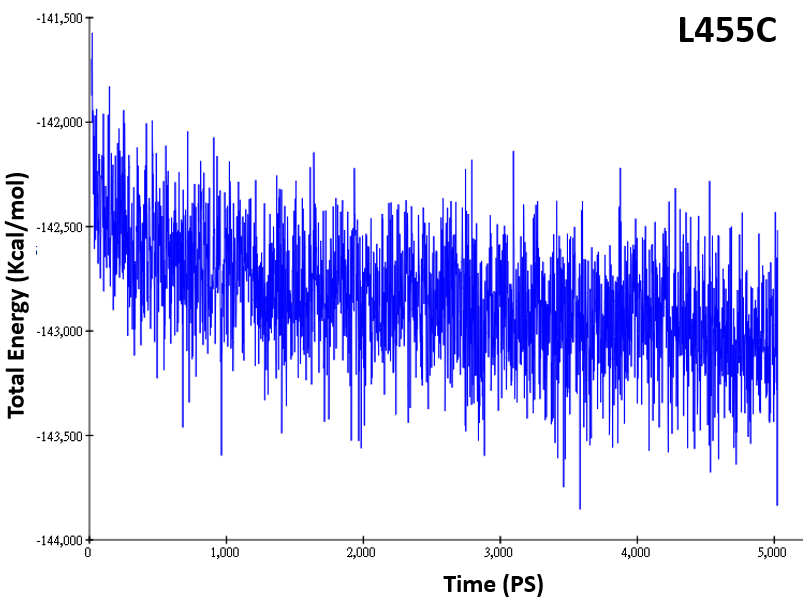

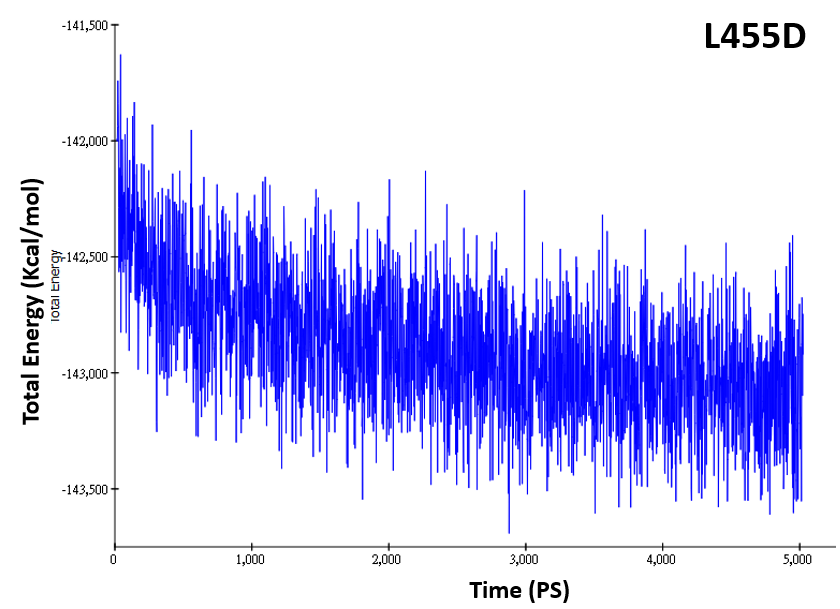


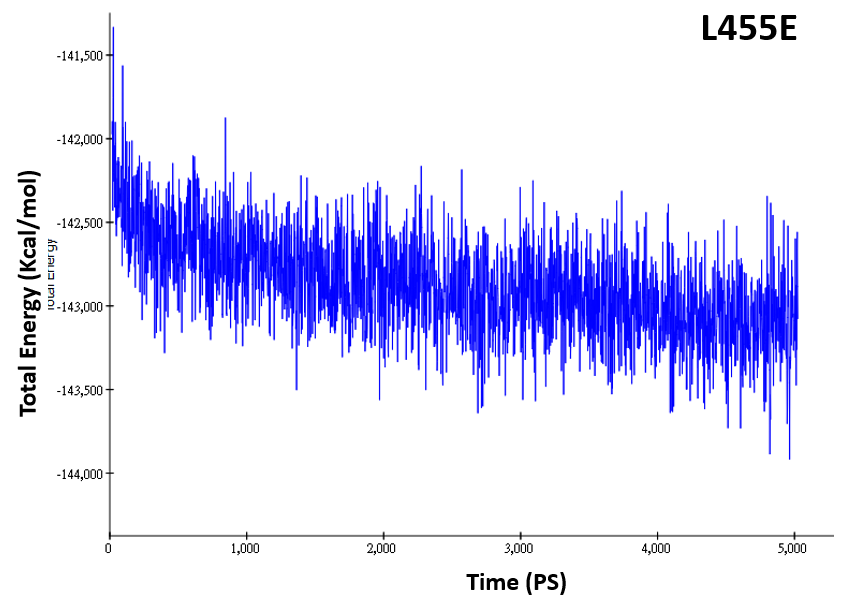

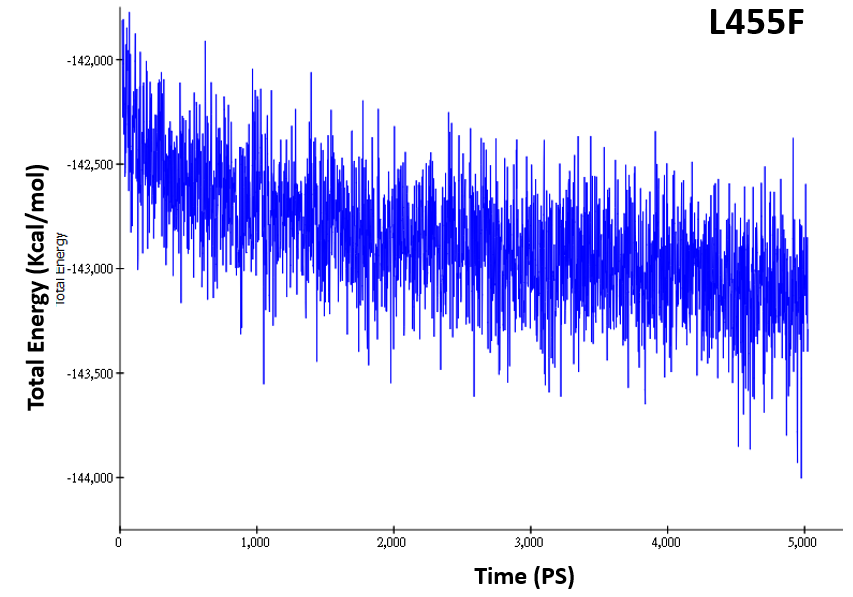

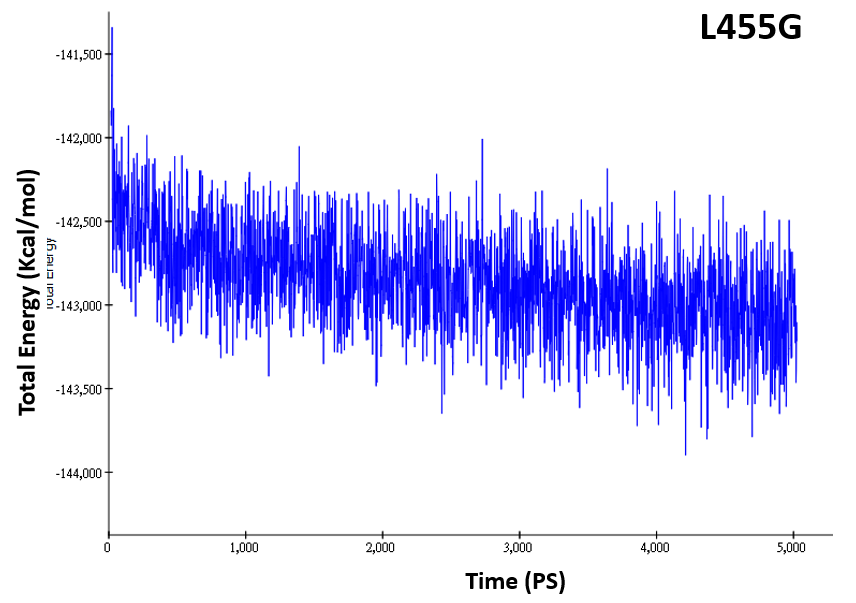


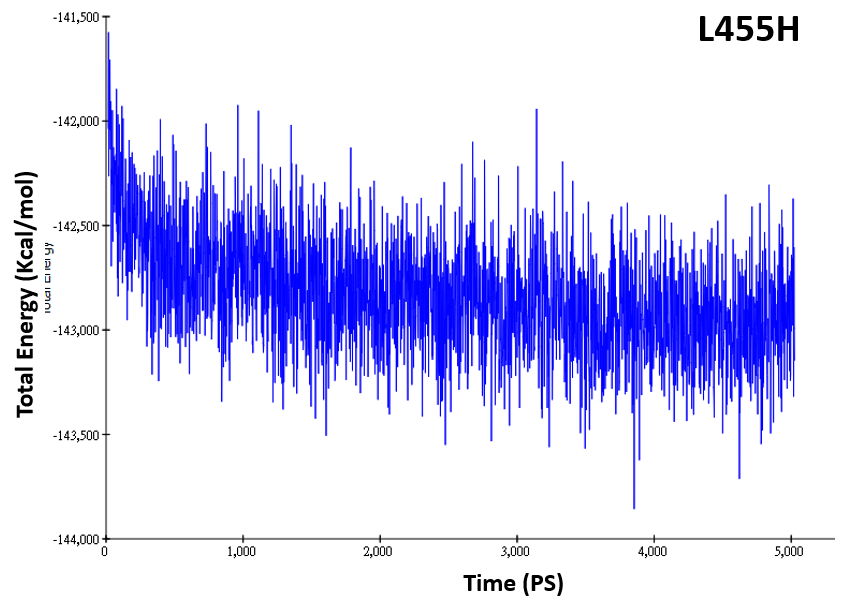

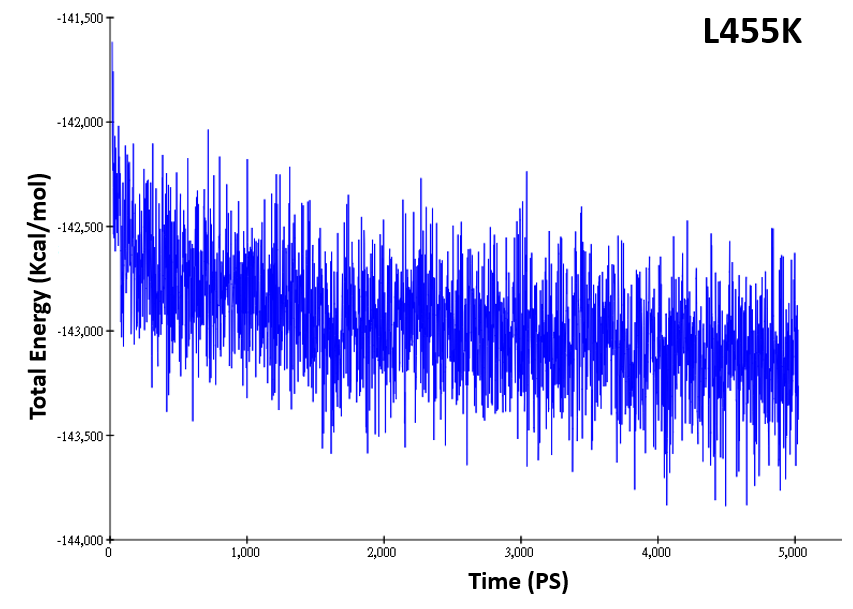

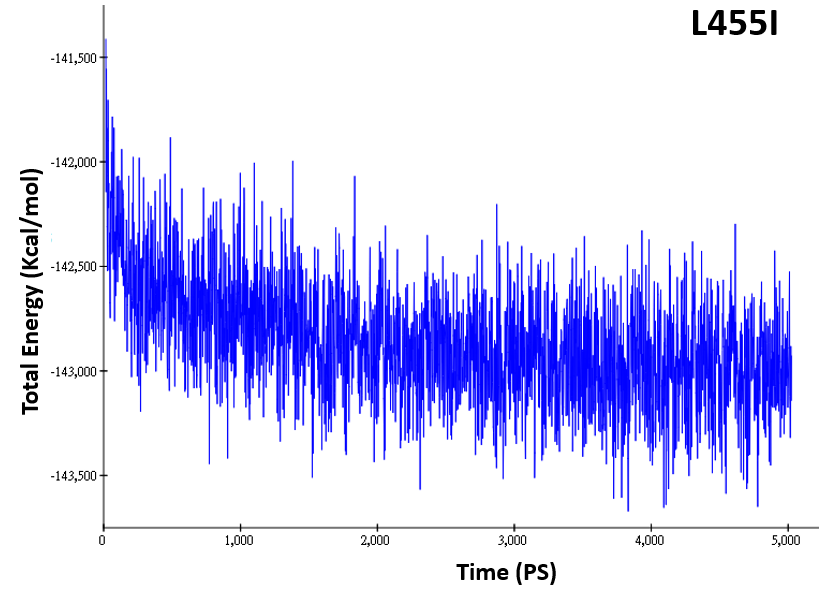


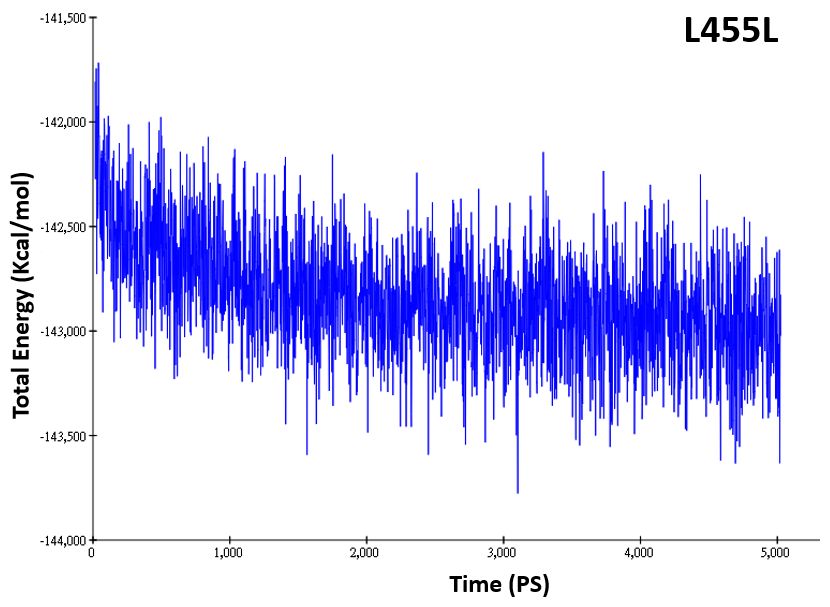


**Figure S9. The molecular dynamics simulations of L455 and its variants of RBD in complex with convalescent antibody CA1-B12 (PDB ID: 7KFV).** The total energies as functions of MD simulation time for 5 ns are shown.


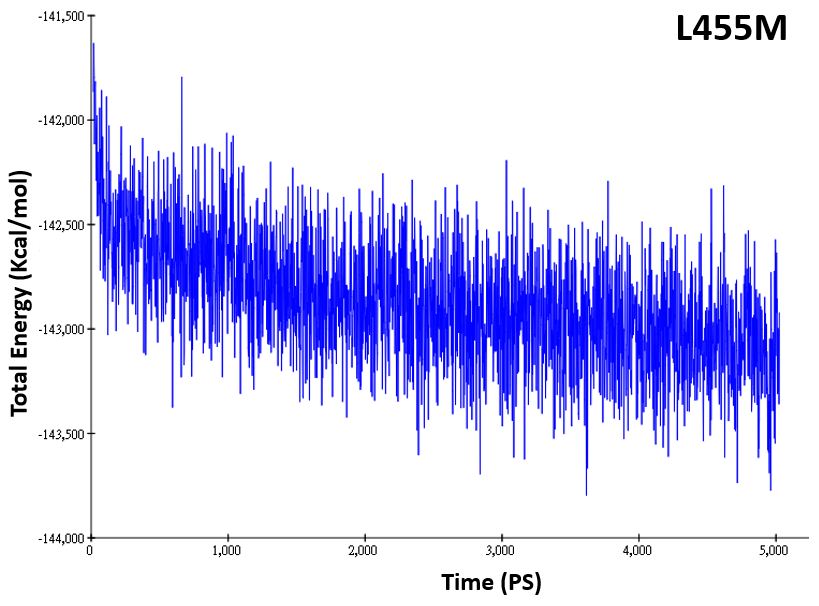

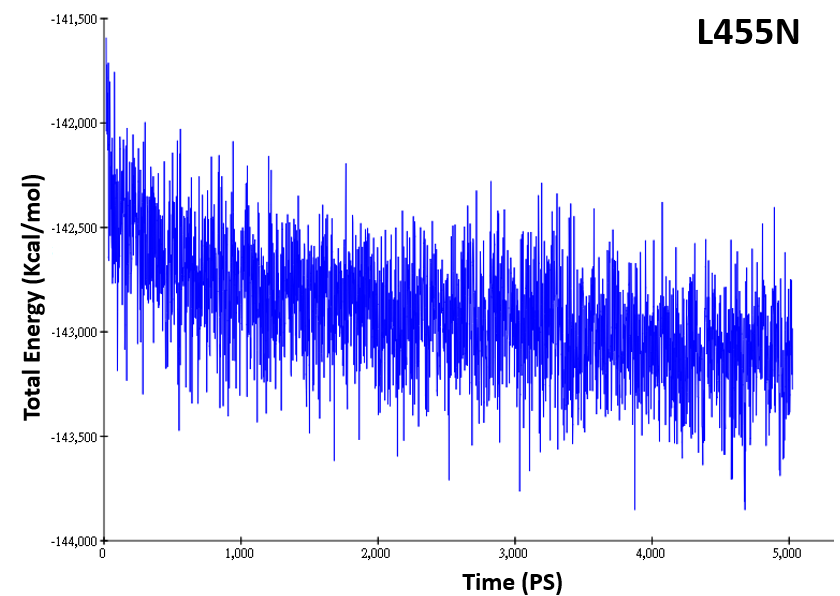

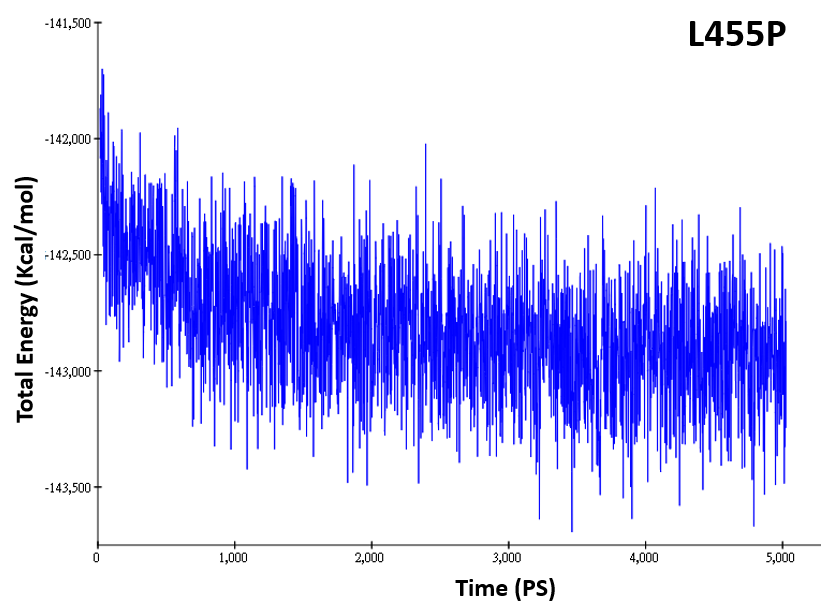


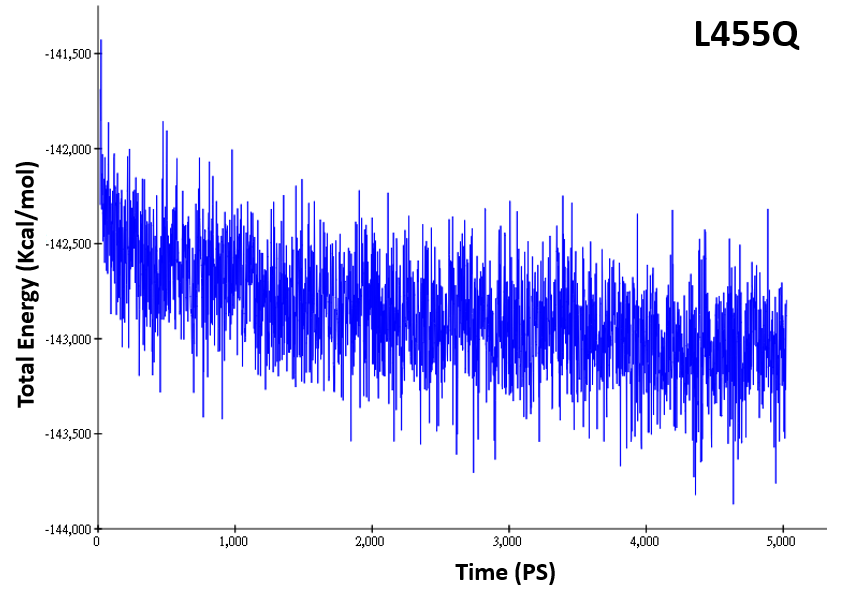

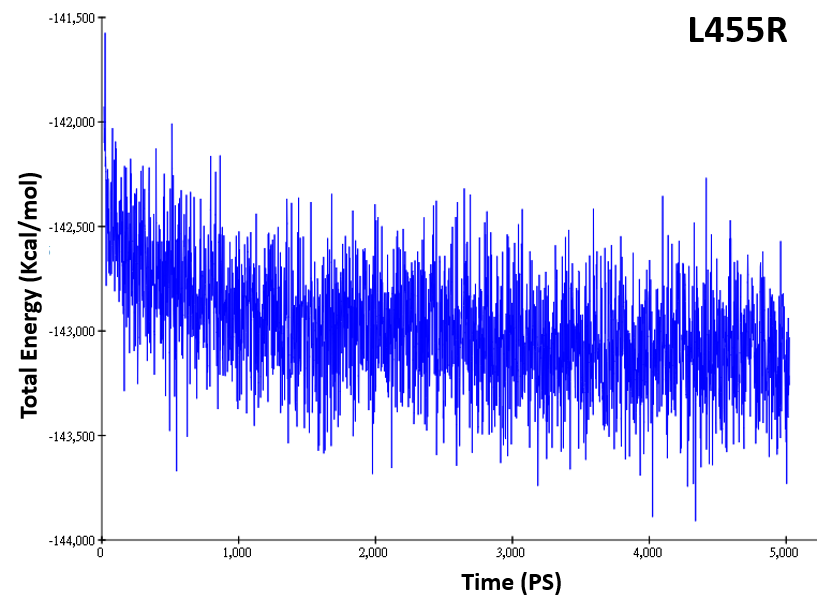

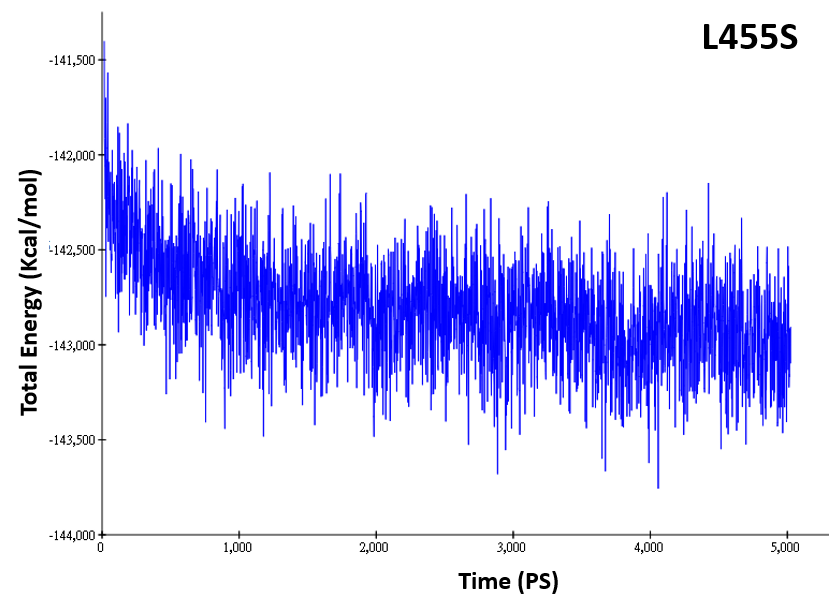


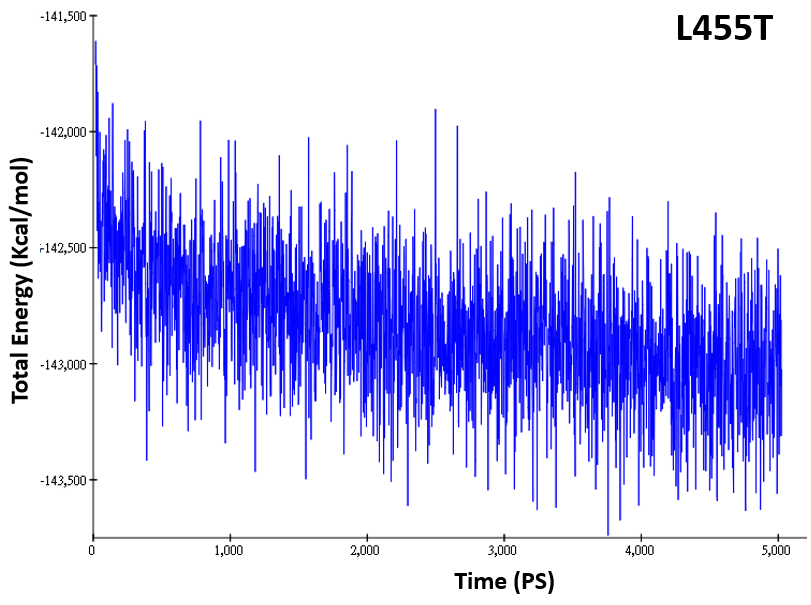

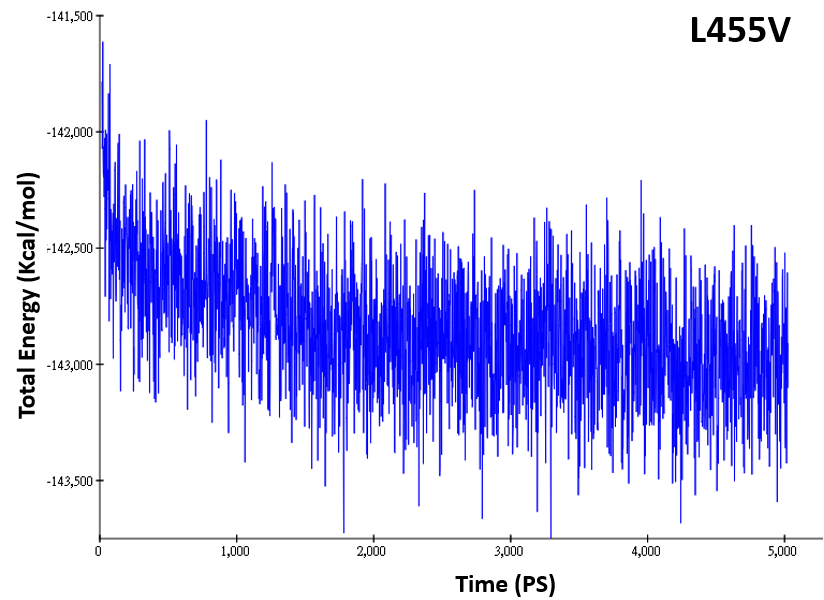

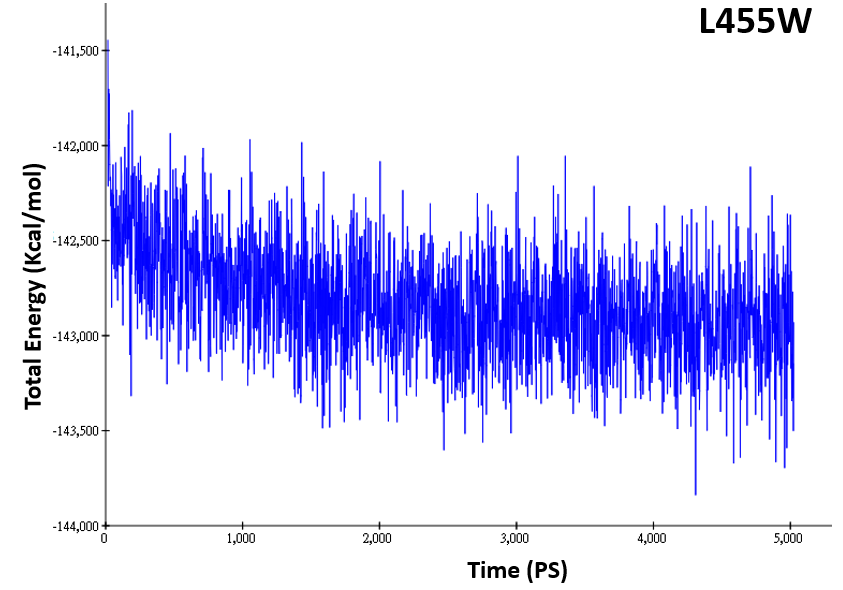


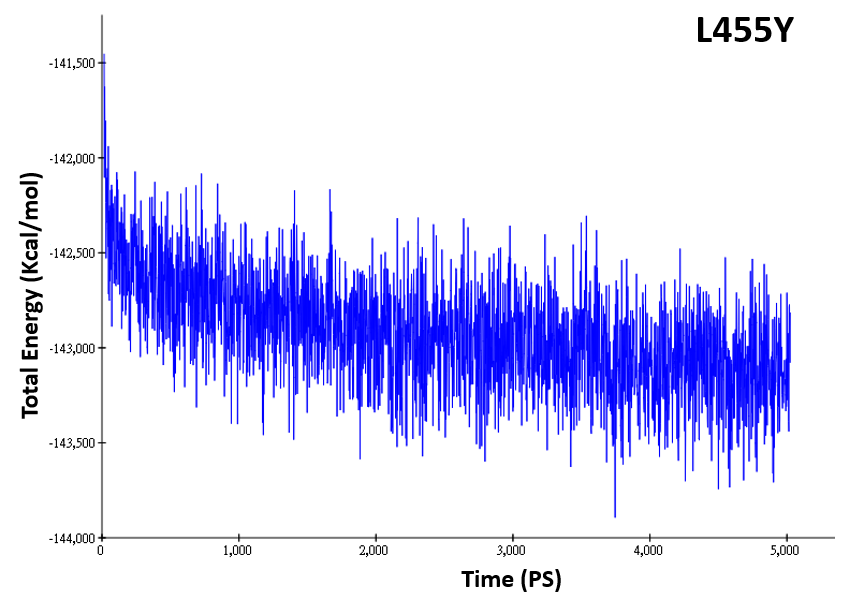


**Figure S10. The molecular dynamics simulations of L455 variants of RBD targeting convalescent antibody CA1-B12 (PDB ID: 7KFV).** The trajectory profiles of total energies during MD simulation time for 5 ns are presented.

**Figure S11. The molecular dynamics simulations of F456 and its variants of RBD in complex with convalescent antibody CA1-B12 (PDB ID: 7KFV).** The total energy changes during the MD simulations for 5 ns are shown.

**Figure S12. The molecular dynamics simulations of F4560 variants of RBD targeting convalescent antibody CA1-B12 (PDB ID: 7KFV) for 5 ns.** The total energy as a function of time of each MD simulation is presented.

**Figure S13. The molecular dynamics simulations of L455 and its variants of RBD in complex with convalescent antibody CA1-B3 (PDB ID: 7KFW) for 5 ns.** The trajectory profiles of total energies of L455 variants in complex with CA1-B3 during MD simulation time for 5 ns are presented.

**Figure S14. The molecular dynamics simulations of L455 variants of RBD targeting convalescent antibody CA1-B3 (PDB ID: 7KFW) for.** The total energies with respect to MD simulation time for 5 ns are shown.

**Figure S15. The molecular dynamics simulations of F465 and its variants of RBD targeting convalescent antibody CA1-B3 (PDB ID: 7KFW).** The total energies as functions of MD simulation time for 5 ns are shown.

**Figure S16. The molecular dynamics simulations of F465 variants of RBD in complex with convalescent antibody CA1-B3 (PDB ID: 7KFW) for 5 ns.** The total energies with respect to MD simulation time for 5 ns are presented.
